# *Trans*-eQTLs Reveal That Independent Genetic Variants Associated with a Complex Phenotype Converge on Intermediate Genes, with a Major Role for the HLA

**DOI:** 10.1371/journal.pgen.1002197

**Published:** 2011-08-04

**Authors:** Rudolf S. N. Fehrmann, Ritsert C. Jansen, Jan H. Veldink, Harm-Jan Westra, Danny Arends, Marc Jan Bonder, Jingyuan Fu, Patrick Deelen, Harry J. M. Groen, Asia Smolonska, Rinse K. Weersma, Robert M. W. Hofstra, Wim A. Buurman, Sander Rensen, Marcel G. M. Wolfs, Mathieu Platteel, Alexandra Zhernakova, Clara C. Elbers, Eleanora M. Festen, Gosia Trynka, Marten H. Hofker, Christiaan G. J. Saris, Roel A. Ophoff, Leonard H. van den Berg, David A. van Heel, Cisca Wijmenga, Gerard J. te Meerman, Lude Franke

**Affiliations:** 1 Department of Genetics, University Medical Center Groningen and University of Groningen, Groningen, The Netherlands; 2 Groningen Bioinformatics Centre, Groningen Biomolecular Sciences and Biotechnology Institute, University of Groningen, Haren, The Netherlands; 3 Department of Neurology, Rudolf Magnus Institute of Neuroscience, University Medical Centre Utrecht, Utrecht, The Netherlands; 4 Department of Pulmonology, University Medical Center Groningen and University of Groningen, Groningen, The Netherlands; 5 Department of Gastroenterology and Hepatology, University Medical Centre Groningen and University of Groningen, Groningen, The Netherlands; 6 NUTRIM School for Nutrition, Toxicology, and Metabolism, Department of General Surgery, Maastricht University Medical Center, Maastricht, The Netherlands; 7 Department of Pathology and Medical Biology, Medical Biology Section, Molecular Genetics, University Medical Center Groningen and University of Groningen, Groningen, The Netherlands; 8 Department of Rheumatology, Leiden University Medical Center, Leiden, The Netherlands; 9 Department of Genetics, University of Pennsylvania, Philadelphia, Pennsylvania, United States of America; 10 Department of Medical Genetics, University Medical Center Utrecht, Utrecht, The Netherlands; 11 Center for Neurobehavioral Genetics, University of California Los Angeles, Los Angeles, California, United States of America; 12 Blizard Institute of Cell and Molecular Science, Barts and The London School of Medicine and Dentistry, Queen Mary University of London, London, United Kingdom; University of Pennsylvania, United States of America

## Abstract

For many complex traits, genetic variants have been found associated. However, it is still mostly unclear through which downstream mechanism these variants cause these phenotypes. Knowledge of these intermediate steps is crucial to understand pathogenesis, while also providing leads for potential pharmacological intervention. Here we relied upon natural human genetic variation to identify effects of these variants on *trans*-gene expression (expression quantitative trait locus mapping, eQTL) in whole peripheral blood from 1,469 unrelated individuals. We looked at 1,167 published trait- or disease-associated SNPs and observed *trans*-eQTL effects on 113 different genes, of which we replicated 46 in monocytes of 1,490 different individuals and 18 in a smaller dataset that comprised subcutaneous adipose, visceral adipose, liver tissue, and muscle tissue. HLA single-nucleotide polymorphisms (SNPs) were 10-fold enriched for *trans*-eQTLs: 48% of the *trans*-acting SNPs map within the HLA, including ulcerative colitis susceptibility variants that affect plausible candidate genes *AOAH* and *TRBV18* in *trans*. We identified 18 pairs of unlinked SNPs associated with the same phenotype and affecting expression of the same *trans-*gene (21 times more than expected, *P*<10^−16^). This was particularly pronounced for mean platelet volume (MPV): Two independent SNPs significantly affect the well-known blood coagulation genes *GP9* and *F13A1* but also *C19orf33, SAMD14, VCL*, and *GNG11*. Several of these SNPs have a substantially higher effect on the downstream *trans*-genes than on the eventual phenotypes, supporting the concept that the effects of these SNPs on expression seems to be much less multifactorial. Therefore, these *trans-*eQTLs could well represent some of the intermediate genes that connect genetic variants with their eventual complex phenotypic outcomes.

## Author Summary

Many genetic variants have been found associated with diseases. However, for many of these genetic variants, it remains unclear how they exert their effect on the eventual phenotype. We investigated genetic variants that are known to be associated with diseases and complex phenotypes and assessed whether these variants were also associated with gene expression levels in a set of 1,469 unrelated whole blood samples. For several diseases, such as type 1 diabetes and ulcerative colitis, we observed that genetic variants affect the expression of genes, not implicated before. For complex traits, such as mean platelet volume and mean corpuscular volume, we observed that independent genetic variants on different chromosomes influence the expression of exactly the same genes. For mean platelet volume, these genes include well-known blood coagulation genes but also genes with still unknown functions. These results indicate that, by systematically correlating genetic variation with gene expression levels, it is possible to identify downstream genes, which provide important avenues for further research.

## Introduction

For many complex traits and diseases, numerous associated single nucleotide polymorphisms (SNPs) have been identified through genome-wide association studies (GWAS)through genome-wide association studies (GWAS) [1]. For many of these identified variants it is still unclear through which mechanism the association between the SNP and the trait or disease phenotype is mediated. A complicating factor is that disease-associated variants might not be the real causal variants, but are in linkage disequilibrium (LD) with the true disease-causing variant, making it difficult to accurately implicate the correct gene for a locus in disease pathogenesis.

Within the major histocompatibility locus (MHC) on 6p, many SNPs have been found to be associated with complex diseases such as celiac disease, inflammatory bowel disease, psoriasis, rheumatoid arthritis, diabetes mellitus, schizophrenia, lung cancer and follicular lymphoma [2]–[10]. An analysis of the Catalog of Published Genome-Wide Association Studies [1] revealed that out of 1,167 unique SNP associations with a reported p<5×10^−7^, 82 (7.0%) were located within the MHC (Fisher's Exact p<10^−30^). Except for celiac disease [11] it remains largely unclear how MHC variants increase disease susceptibility.

However, common variants have been identified that might exert their function by altering gene expression rather than by altering protein structure [2], [12]–[16] (expression quantitative trait loci, eQTLs). Comprehensive eQTL mapping (or genetical genomics [17]) will enable us to assess for every known disease-associated variant if it significantly affects gene expression. Genetic variants that affect expression of genes that map in their vicinity (*cis*-eQTLs) can potentially pinpoint the true disease gene from an associated locus. In addition, genetic variants may also affect expression of genes that reside further away or are on different chromosomes (*trans*-eQTLs) [18]. These *trans*-eQTLs are especially interesting, since they allow us to identify downstream affected disease genes which were not implicated by GWAS studies at all, and thereby potentially having the ability to reveal previously unknown (disease) pathways.

In this study we performed a comprehensive eQTL mapping to explore the downstream effects of SNPs on gene expression by analyzing genotype and expression data of 1,469 unrelated samples. In addition to a genome-wide analysis, we also performed a focused analysis for disease- and trait-associated SNPs and SNPs located within the HLA. We replicated the identified *trans*-eQTLs in a collection of monocyte expression data and expression data from subcutaneous adipose, visceral adipose, muscle and liver tissue. Principal component analysis (PCA) enabled us to remove non-genetic expression variation [19], [20], resulting in increased power to detect eQTLs. A stringent probe-mapping strategy was used to filter out false-positive *cis-*eQTLs due to primer-polymorphisms and false-positive *trans-*eQTLs due to cross-hybridizations. Furthermore, a permutation strategy was utilized that corrects for multiple-testing, while preventing potential confounders such as non-even distribution of SNP markers and expression probe markers across the genome, differences in minor allele frequency (MAF) between SNPs, linkage disequilibrium (LD) within the genotype data, and correlation between expression probes.

## Results

### *Cis*- and *trans*-eQTL mapping

Results of a genome-wide eQTL analysis on 289,044 common SNPs, present on the Illumina HumanHap300 platform in peripheral blood expression data of 1,469 unrelated individuals, are provided in Table 1, Table S1, Table S2, Figure S1 (controlling false discovery rate (FDR) at 0.05 using a permutation strategy).

**Table 1:** Detected eQTLs in 1,469 genetical genomics samples for 289,044 common SNPs and for 1,167 trait-associated SNPs.

eQTL analysis on 289,044 common SNPs			
	*cis*-eQTLs (FDR<0.05)	*trans*-eQTLs (FDR<0.05)	
**Spearman's correlation threshold**	P<1.73×10^−3^	P<3.6×10^−9^	
**Number of tests performed**	2,329,207	13,292,122,142	
**Number of unique eQTL probes**	10,872	244	
**Number of unique eQTL genes**	7,589	202	
**Number of unique eQTL SNPs**	48,717 (16.9% of all tested SNPs)	467 (0.2% of all tested SNPs)	
**Number of unique MHC eQTL SNPs**	1,586 (3.3% of *cis*-eQTL SNPs)	155 (33.2% of *trans*-eQTL SNPs)	

eQTL analysis on 1,167 trait-associated SNPs			
	*cis*-eQTLs (FDR<0.05)	*trans*-eQTLs (FDR<0.05)	*trans*-eQTLs (FDR<0.50)
**Spearman's correlation threshold**	P<3.7×10^−3^	P<2.0×10^−7^	P<1.02×10^−5^
**Number of tests performed**	15,371	53,629,458	53,629,458
**Number of unique eQTL probes**	679	130	726
**Number of unique eQTL genes**	538	113	576
**Number of unique eQTL SNPs**	472 (40.4% of all tested SNPs)	67 (5.7% of all tested SNPs)	462 (39.6% of all tested SNPs)
**Number of unique MHC eQTL SNPs**	65 (13.8% of *cis-*eQTL SNPs)	32 (47.8% of *trans*-eQTL SNPs)	52 (11.3% of *trans*-eQTL SNPs)

For 289,044 SNPs, present on the commonly used Illumina HumanHap300 platform, the false discovery rate (FDR) was controlled at 0.05 for both *cis*- and *trans*-eQTLs. For the analysis of 1,167 successfully imputed SNPs that have been found associated with a quantitative trait or disease the FDR was controlled at 0.05 for the *cis*- and *trans*-eQTLs. We also performed a trans-eQTL analysis for these SNPs while controlling the FDR at 0.50 to generate more hypotheses. The number of unique genes was determined using Ensembl 52 (NCBI 36.3 release).

**Figure 1:**
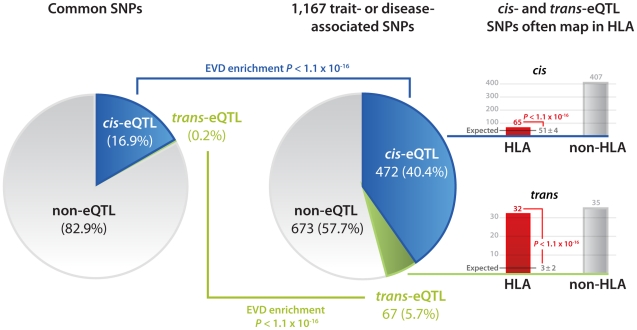
Disease and trait-associated SNPs are enriched for both *cis*- and *trans*-eQTLs. 17% of SNPs, present on common SNP platforms, affect gene expression levels in *cis* or *trans* (at FDR of 0.05). This is substantially different from 1,167 SNPs that have been found associated with traits or disease: 40.4% affect gene expression in cis, while 5.7% of these SNPs affect gene expression in *trans*. These eQTL SNPs significantly more often than expected map within the HLA (13.8% of *cis*-eQTLs, 47.8% of *trans*-eQTLs, extreme value distribution p<1.1×10^−16^).

As reported before [21]–[25] we also observed that eQTLs are strongly enriched for trait-associated SNPs (SNPs associated with a trait or disease phenotype, as reported in the Catalog of Published Genome-Wide Association Studies [1]): We therefore concentrated on these variants and imputed (Impute v2.0 [26]) additional genotype data permitting us to test 1,167 trait-associated SNPs. After removing false-positive eQTLs due to primer-polymorphisms and cross-hybridization 472 (40.4%) of these SNPs were *cis*-eQTLs, affecting the expression of 679 different transcripts, representing 538 genes (Figure 1, Table 1, Figure S2, Table S3). 67 (5.7%) SNPs were *trans*-acting on 130 different transcripts, representing 113 genes (Table S4). Results on the number of detected eQTLs per complex trait are provided in Table S5 and Figure S3. For nearly all significant *trans-*eQTLs the effect was present in each of the seven individual patient and controls cohorts, making up the total dataset (Table S6).

These *trans*-eQTLs provide valuable insight on previously unknown functional downstream consequences trait-associated SNPs have, e.g. rs2395185 is the strongest susceptibility variant for ulcerative colitis [27] (UC) but also the strongest SNP, *trans*-acting on Acyloxyacyl hydrolase (*AOAH*, p = 1.0×10^−36^), an enzyme that modulates host inflammatory responses to gram-negative bacterial invasion. It is known that deficiencies in response mechanisms against bacterial products like lipopolysaccharide, present on gram-negative bacterial cell walls, play an important role in UC disease pathogenesis [28]. Within the peripheral blood we observed that AOAH is significantly co-expressed with colony stimulating factor 1 receptor (*CSF1R*, r = 0.21) and major histocompatibility complex class II DR alpha (*HLA-DRA*, r = 0.19). Hyperstimulation of *CSF1R* has been implicated in UC [29], while *HLA-DRA* is one of the positional UC candidate genes mapping in very close proximity to rs2395185. Another UC HLA variant, rs9268877, was *trans*-acting on T cell receptor beta variable 18 (*TRBV18*), part of the TCR_ß_ locus at 7q34. It is known that TCR_ß_ mutant mice develop chronic colitis [30].

**Figure 2:**
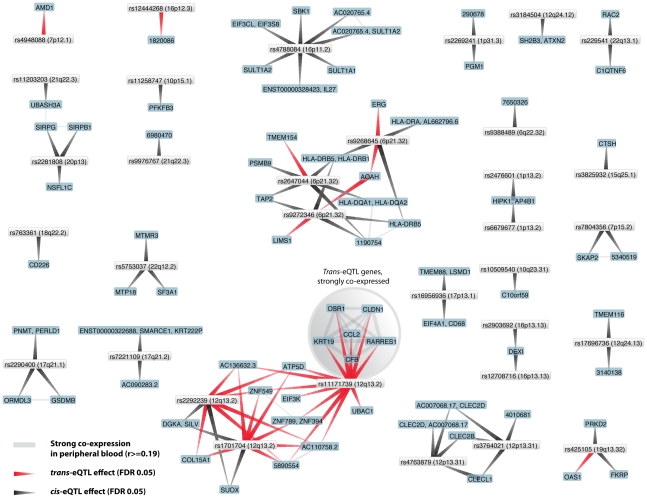
Type 1 diabetes associated SNPs both affect genes in *cis* and in *trans*.

For type 1 diabetes (T1D) we observed that 59% (30/51) of the known and tested T1D associated SNPs are *cis-*acting (on in total 53 unique genes) and 17% (9/50) are *trans*-acting on 22 unique genes (Figure 2). Potentially interesting *trans*-genes include *CCL2*, *CFB*, *CLN1*, *KRT19*, *OSR1* and *RARRES1*, all strongly co-expressed with each other. *CCL2* and *CFB* are known immune response genes and have been implicated in T1D before [31]–[33].

For breast cancer we observed that rs3803662 [34] is *trans*-acting on origin recognition complex subunit 6 (*ORC6L)*. This gene is involved in DNA replication and has been frequently used as part of prognostic profiles for predicting the clinical outcome in breast cancer [35], [36].

**Figure 3:**
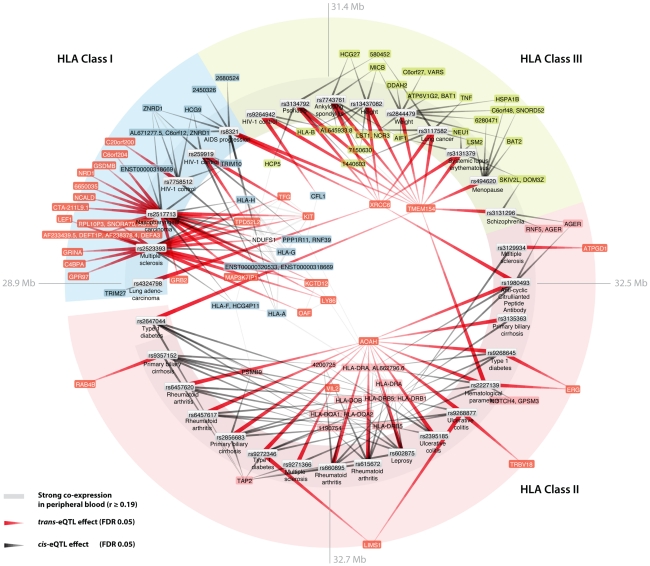
Human leukocyte antigen (HLA) trait-associated SNPs affect gene expression levels in *trans*. Thirty-two trait-associated SNPs that map within the HLA are *trans*-acting on other genes. *Trans*-genes are indicated in red. Peripheral blood co-expression (Pearson correlation coefficient r≥0.19, p<10^−11^) between genes is indicated in light grey. Several trans-genes are co-expressed with HLA genes.

We observed a marked enrichment for SNPs within the MHC among the *cis-* and *trans-*acting trait-associated SNPs: 65 of 472 *cis*-acting SNPs (13.8%, EVD p<1.0×10^−16^) and 32 of 67 *trans*-acting SNPs (47.8%, EVD p<1.0×10^−16^) mapped within the MHC (Figure 3). These SNPs all map to the Human Leukocyte Antigens (HLA) locus: SNPs within the HLA class I region, class II region and class III region affect 20, 7 and 2 different genes in *trans*, respectively.

### Biological convergence of *cis*- and *trans*-eQTLs

While multiple associated SNPs have been identified for many complex diseases, it often remains unclear what the intermediate effects of these variants are that eventually lead to disease. It is reasonable to assume that for a particular phenotype the different associated SNPs eventually converge on the same downstream gene(s) or pathways.

**Figure 4:**
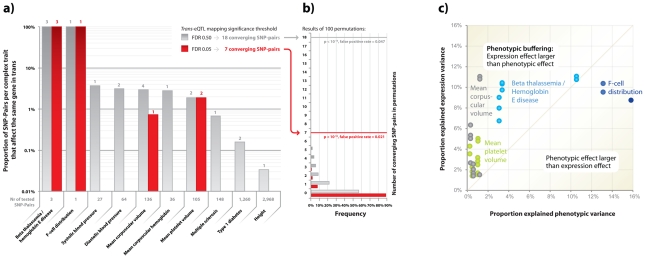
Pairs of SNPs that cause the same phenotype more frequently than expected also affect the same downstream genes. Various pairs of unlinked SNPs cause the same phenotype but also converge on the same downstream genes. a) When using *cis*- and *trans*-eQTLs, identified when controlling FDR at 0.05, 7 unique pairs of SNPs cause the same phenotype but also affect the same downstream gene. When controlling the FDR at 0.50 for the *trans*-eQTLs, 18 unique pairs of SNPs show this convergence. b) This is significantly higher than expected, determined using 100 permutations. c) The SNPs that affect these downstream genes in most instances explain a proportion of the downstream gene expression variation that is substantially higher than what their effect is on the eventual phenotypes.

We identified 7 unique pairs of unlinked SNPs that are associated with the same phenotype and that also affect the same downstream genes in *trans* or *cis* (at FDR 0.05, Table 2, Figure 4a). In order to establish whether this was more than expected by chance, we repeated this analysis, while using a set of trans-eQTLs, equal in size to the set of real *trans*-eQTLs, most significant after having permuted the expression sample identifiers. We performed this procedure 100 times, and observed on average only 0.15 unique pairs of unlinked SNPs (range [0, 3], Figure 4b) that showed this convergence, which indicates that the observed number of converging pairs of SNPs is 47 times more than expected (EVD p<1.0×10^−16^) and implies a false-positive rate of 0.021.

**Table 2:** Trait-associated SNPs converge on the same downstream genes.

Complex Trait	Unlinked SNP-pair		Explained trait variance		SNP-pair convergences	eQTL significance		Explained expression variance	
	SNP 1	SNP 2	SNP 1	SNP 2	on gene (probes)	SNP 1	SNP 2	SNP 1	SNP 2
Beta thalassemia	rs766432	rs2071348	3.3% [40]	3.0% [40]	*HBG2* ^a^ (4010040, 450537, H8v2-6400079)	2.12×10^−29^, 7.67×10^−37^, 6.95×10^−07^	4.22×10^−24^, 6.46×10^−24^, 3.80×10^−06^	9.7%, 10.4%, 10.3%	8.0%, 6.7%, 9.0%
	rs9376092	rs766432	10.5% [40]	3.3% [40]	*HBG2* ^a^ (4010040, 450537)	1.73×10^−32^, 9.49×10^−39^	2.12×10^−29^, 7.67×10^−37^	10.8%, 11.1%	9.7%, 10.4%
	rs9376092	rs2071348	10.5% [40]	3.0% [40]	*HBG2* a (4010040, 450537)	1.73×10^−32^, 9.49×10^−39^	4.22×10^−24^, 6.46×10^−24^	10.8%, 11.1%	8.0%, 6.7%
F-cell distribution	rs1427407	rs9399137	13.1% [38]	15.8% [38]	*HBG2* a (4010040, 450537)	1.18×10^−28^, 1.21×10^−36^	1.70×10^−26^, 1.86×10^−30^	9.51%, 10.35%	8.74%, 8.75%
Systolic blood pressure	rs3184504	rs2681492	N/A	N/A	*LOC338758* (6650035)	1.28×10^−06^	9.17×10^−08^	1.87%	2.27%
Diastolic blood pressure	rs3184504	rs2681472	N/A	N/A	*LOC338758* (6650035)	1.28×10^−06^	2.23×10^−08^	1.87%	2.49%
	rs653178	rs2681472	N/A	N/A	*LOC338758* (6650035)	1.54×10^−06^	2.23×10^−08^	1.85%	2.49%
Mean corpuscular volume	rs12718597	rs643381	0.26% [41]	0.50% [41]	*VWCE* (1450608)	3.39×10^−10^	1.74×10^−06^	2.65%	1.61%
	rs2540917	rs643381	0.24% [41]	0.50% [41]	*ESPN* (3440630)	1.95×10^−15^	6.20×10^−07^	4.99%	1.99%
	rs4895441	rs2540917	1.12% [41]	0.24% [41]	*HBG2* a (4010040, 450537)	2.74×10^−32^, 1.31×10^−38^	2.87×10^−19^, 3.19×10^−18^	10.71%, 10.99%	6.32%, 5.29%
	rs4895441	rs643381	1.12% [41]	0.50% [41]	*RAP1GAP * a (4890181)	2.46×10^−06^	5.57×10^−06^	1.51%	1.41%
					*PDZK1IP1* (3170270)	4.27×10^−06^	7.44×10^−10^	1.45%	2.55%
Mean corpuscular hemoglobin	rs628751	rs7776054	0.34% [41]	1.02% [41]	*PDZK1IP1* (3170270)	7.74×10^−10^	8.97×10^−07^	2.55%	1.65%
Mean platelet volume	rs12485738	rs11602954	0.93% [42]	0.41% [42]	*GP9* b (1050292)	3.62×10^−17^	1.14×10^−07^	4.82%	1.93%
					*GNG11* (1580025)	9.67×10^−12^	2.23×10^−06^	3.22%	1.52%
					*F13A1* (2230241)	5.37×10^−09^	3.13×10^−09^	2.54%	2.38%
					*SAMD14* b (5560280)	4.08×10^−18^	3.10×10^−06^	5.05%	1.47%
					*C19orf33* (630470)	6.26×10^−11^	1.16×10^−08^	2.86%	2.37%
					*VCL* b (70592)	7.49×10^−07^	6.81×10^−06^	1.72%	1.39%
	rs12485738	rs11071720	0.93% [42]	0.18% [42]	*TPM1* (5560246, 610519)	1.47×10^−08^, 1.45×10^−06^	1.38×10^−13^, 4.41×10^−13^	2.58%, 1.60%	4.32%, 3.58%
Multiple sclerosis	rs2523393	rs9271366	N/A	N/A	*TGFBR2* (2340324)	5.15×10^−07^	1.07×10^−06^	2.01%	1.90%
Type 1 diabetes	rs9272346	rs11171739	N/A	N/A	*KRT18* (6580270)	1.87×10^−06^	4.72×10^−06^	2.06%	1.70%
	rs9272346	rs1701704	N/A	N/A	*KRT18* (6580270)	1.87×10^−06^	9.40×10^−06^	2.06%	1.39%
Height	rs910316	rs10946808	N/A	N/A	*BTN3A2* (4610674)	5.42×10^−06^	9.79×10^−10^	1.40%	2.60%

Indicated are 18 pairs of unlinked SNPs that are associated with the same complex phenotype and that also affect the expression levels of the same downstream gene(s) in *cis* (FDR 0.05) or *trans* (FDR 0.50).

**a** Erythrocyte specific gene according to HaemAtlas [43].

**b** Megakaryocyte specific gene according to HaemAtlas [43].

Explained phenotypic variation is shown for traits when reported in the original papers (indicated in superscript) that describe these SNP – phenotype association.

Due to this highly significant enrichment of converging pairs of SNPs and its low estimated false-positive rate, we also ran an analysis where we had relaxed the FDR for *trans*-eQTLs to 0.50 (Table S7). Here we observed 18 pairs of SNPs that converge on the same genes, whereas in the 100 subsequent permutations we observed this only on average for 0.84 SNP-pairs (range [0, 5], 21 times more expected by chance, EVD p<1.0×10^−16^, implying a false-positive rate of 0.047, Table 2, Figure 4b).

**Figure 5:**
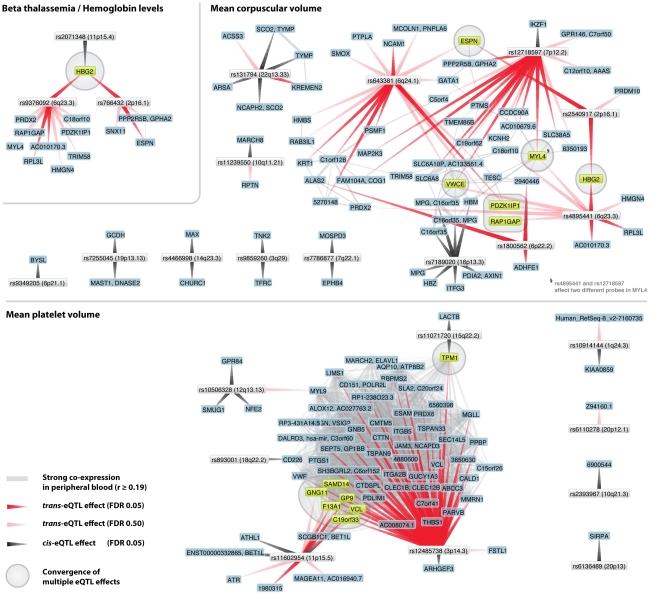
Trait-associated SNPs show convergence on multiple genes. For several traits different and unlinked SNPs affect the same genes in *cis* or *trans*. For beta thalassemia three different loci affect hemoglobin (*HBG2*) gene expression (one in *cis*, indicated with gray arrow, two in *trans* (at FDR 0.05), indicated with red arrows). For mean corpuscular volume (MCV) the same *trans*-effects on HBG2 (at FDR 0.05) exist, but convergence is also apparent on *ESPN*, *VWCE*, *PDKZ1IP1* and *RAP1GAP* (at FDR 0.50). For mean platelet volume (MPV) numerous *trans*-effects on genes involved in blood coagulation were identified. Two MPV loci (rs12485738 on 3p26.3 and rs11602954 on 11p15.5) both affect *GP9*, *F13A1* and *C19orf33* (at FDR 0.05) and *SAMD14*, *GNG11* and *VCL* (at FDR 0.50). Peripheral blood co-expression (Pearson correlation coefficient r≥0.19, p<1.0×10^−11^) between genes is indicated in light grey.

Many of these converging downstream genes make biological sense: three independent loci, associated with hemoglobin protein levels [37]–[39] and ß thalassemia susceptibility [40], significantly affect hemoglobin gamma G (*HBG2*) gene expression levels (each with p<1.0×10^−23^, Figure 5). For mean corpuscular volume (MCV, Figure 5) two unlinked MCV SNPs [41], [42] also affect *HBG2* gene expression levels in *trans* (at FDR 0.05), while other pairs of MCV SNPs converge on *ESPN*, *VWCE*, *PDZK1IP1* and *RAP1GAP.*

For mean platelet volume (MPV) we observed that MPV SNPs rs12485738 on 3p26 and rs11602954 on 11p15 affect several transcripts in *trans*. These two SNPs converge on *GP9*, *F13A1, C19orf33, SAMD14, VCL* and *GNG11*. As *GP9* and *F13A1* are known blood coagulation genes, *C19orf33* is a potential candidate gene, involved in coagulation as well. This is substantiated by strong co-expression between *GP9* and *C19orf33* within peripheral blood (Pearson r = 0.45, p = 7.0×10^−63^) and the fact these SNPs independently also affect various other blood coagulation genes in *trans* (including *CD151, GP1BB, ITGA2B, MMRN1, THBS1* and *VWF*, Figure 4). Many of these are specific to megakaryocytes that are platelet precursor cells [43]. As expected, the Gene Ontology term ‘blood coagulation’ is strongly overrepresented among all these *trans-*genes, Fisher's exact p = 1.0×10^−10^.

We observed that MPV SNP *rs12485738* (on 3p14.3) was also *trans-*acting on tropomyosin 1 (*TPM1*, 15q22.2, p = 9.7×10^−9^), a gene that is also regulated in *cis* by another MPV variant (*rs11071720 on 15q22.2*, p = 1.4×10^−13^). We observed this for two different expression probes that map within different locations of the *TPM1* transcript (probes 5560246 and 610519), and note strong co-expression for these two *TPM1* probes with 46 MPV *trans*-genes (Pearson r>0.19, p<1.0×10^−11^, including five known coagulation genes). Although several genes reside within the rs11071720 MPV locus, these observations strongly implicate *TPM1* as the causal MPV gene.

For both MPV and MCV we observed that the identified *cis*- and *trans-*eQTL probes generally were more strongly co-expressed in peripheral blood than expected (Figure S4, MPV co-expression Wilcoxon P<10^−200^, MCV co-expression Wilcoxon P = 0.009), substantiating the likelihood these genes reflect coherent biological sets. We repeated this co-expression analysis after we had regressed out all *cis*- and *trans*-eQTL effects, and observed that most of this co-expression was independent of the eQTL SNP-effect on the expression of these genes, which further substantiates that these genes are biologically related (MPV co-expression Wilcoxon P<1^−200^, MCV co-expression Wilcoxon P = 0.018).

### Phenotypic buffering

Although the observed convergence provides insight into downstream genes, it is not clear whether the MPV or MCV phenotypes are eventually caused through these *trans*-genes, or whether these *trans*-eQTLs emerged as a result of changes to the volume of the platelets or the erythrocytes.

In order to gain insight into this, we analyzed the effect size of these SNP variants on both the expression levels and the phenotypes. While the effect sizes of these trait-associated SNPs on eventual phenotypes were usually small, their intermediate (molecular) effects was often substantially larger. This supports the notion that the effect on e.g. MPV and MCV is through these *trans*-genes, and suggests the presence of ‘phenotypic buffering’, shown previously in plants [44], in humans (Table 2, Figure 4b): the effects of the 18 converging pairs of SNPs on gene expression levels were often substantially higher than the originally reported effect sizes on the trait-phenotypes. For example, several MPV- and MCV-associated SNPs explain between 1.41% and 10.99% of *trans*-expression variation within the 1,469 unrelated samples, whereas these SNPs only explain between 0.24% and 1.12% of the MPV and MCV phenotype variation (and as such required over 13,000 samples [41], [42] for identification, Figure 4b).

### Replication of *trans*-eQTLs in monocytes and four additional primary tissues

We analyzed peripheral blood which is a mixture of different hematopoetic cell types. In addition, we also assessed whether the identified trait-associated *trans*-eQTLs (detected at FDR 0.05) could be replicated in a single cell-type dataset. This is an important question, as it is potentially possible that the *trans*-acting SNP are able to alter the amount, volume or ratio of certain blood cell types, which might as a consequence result in an indirect net effect on the measured gene expression levels within the mix of the cells that comprise whole blood.

We therefore analyzed monocyte expression data from 1,490 independent samples [45] and did not find evidence that this was a widespread phenomenon as we could replicate 46 out of the 130 different *trans*-eQTLs (each of these with a nominal p<1.0×10^−5^ in the monocyte data, Table S8). These replicated eQTLs include the genes *AOAH, HBG2, GP9, F13A1, SAMD14, CD151, ITGA2B, MMRN1, THBS1, VWF* and *TPM1* mentioned above. Surprisingly we could also replicate the *trans*-eQTL effects on various blood-coagulation genes for mean platelet volume SNP rs12485738: One might argue that rs12485738 primarily increases platelet volume, resulting in a relatively higher volume of platelet-RNA when assessing total peripheral blood RNA. If this were to be the case, a measurable *trans-*effect is expected for platelet-specific (blood coagulation) genes in whole blood. Such an effect would then not actually be an expression-QTL, but rather a ‘cellular-QTL’. However, the *trans-*eQTLs for rs12485738 were also present in single cell-type monocyte datasets, indicating that the above concerns do not apply. Clearly, *trans*-eQTL effects can manifest themselves outside the primary cell-type, in which they are expected to operate.

We also replicated 18 trait-associated *trans*-eQTLs (including *AOAH*, detected at FDR 0.05) in an independent dataset comprising four different non-blood tissues (subcutaneous adipose, visceral adipose, liver and muscle, Figure S5, Table S9 and S10). Since this dataset comprised only 90 samples, it is very encouraging that 18 *trans*-eQTL could be replicated.

## Discussion

Here we investigated gene expression in peripheral blood from 1,469 individuals to identify *cis-* and *trans-*effects of common variants on gene expression levels. When comparing to other genetical genomics studies [12]–[14], [16], [18], [21]–[24], [45], [46] we observe an increasing percentage of genes that are *cis-* or *trans-*regulated (39% of 19,689 unique genes at FDR 0.05). When eQTL studies further increase the sample-sizes and thus increase statistical power, we expect that for the far majority of genes the expression levels are to some extent determined by genetic variation.

GWA studies have identified many loci, but it is still often unclear what the affected gene in each locus is. Here we showed that 39% of trait-associated SNPs affect gene expression in *cis* which is helpful in pinpointing the most likely gene per susceptibility locus. However, GWAS do not immediately provide insight in the *trans-*effects of these susceptibility variants on downstream genes. Here we identified for 2.6% of all trait-associated SNPs *trans*-eQTL effects on in total 113 unique genes. While some of these *trans*-eQTLs are known to be involved in these phenotypes (such as *HBG2* in hemoglobin protein levels and ß-Thallasemia), most of these genes have not been implicated before in these complex traits, and provide additional insight in the downstream mechanisms of these variants. Interestingly, 48% of *trans*-acting trait-associated SNPs map within the HLA, indicating the HLA has a prominent role in regulating peripheral blood gene expression. This might partly explain why the HLA has been found to be associated with so many different diseases.

While we concentrated on peripheral blood, we could replicate 35% of the *trans*-eQTLs in monocytes. Particularly surprising was the observation that for SNPs, known to affect the volume of platelets or erythrocytes the identified *trans*-eQTL effects in whole blood were also present in these monocytes. Among these replicated genes are a considerable number of highly plausible *trans*-genes. For example, for mean platelet volume SNP rs12485738 we detected the same *trans*-eQTL effects on seven well-known blood coagulation genes (*F13A1, GP1BB, GP9, ITGA2B, MMRN1, THBS1* and *VWF*) in both the peripheral blood data and the monocyte data. Interestingly, in both datasets, *trans*-effects for this SNP on another 31 genes were identified as well, which suggests these genes play a role in blood coagulation. It can thus be concluded that *trans-*eQTLs, identified in peripheral blood, generally apply to monocytes as well. We assumed these eQTLs might therefore also be present in other, non-blood tissues, as previously observed for rodents [47]–[49]. Indeed we could replicate some of these *trans*-eQTLs in a smaller dataset of four non-blood tissues. Importantly, as mentioned before [46], the allelic directions were nearly always identical to blood, which implies that *trans-*eQTLs, if also present in another tissue, work in the same way.

Our observation that sets of independent SNPs, associated with the same complex phenotype sometimes also affect exactly the same *trans*-gene, further substantiates the validity of our findings. Based on the reported effect-sizes of these variants on these complex phenotypes, we have shown here that the individual effects of these SNPs on *trans-*gene expression can often be stronger. This suggests that these down-stream gene expression effects do not fully propagate to the eventual phenotype and are somehow buffered. This ‘phenotypic buffering’ has been observed before in plants [44] and suggests that additional compensatory mechanisms exist that control these complex phenotypes. However, we do realize that accurate estimates on this phenomenon requires the availability of both gene-expression and phenotype data for these traits. As we did not have these phenotypes for our samples, we relied upon estimates from literature. Future studies that have collected both genome-wide genotype, expression and phenotype data from the same individuals will permit answering the question what the extent of this phenotypic buffering is. We should emphasize that the number of converging pairs of SNPs that we identified must be a very strong underestimate, and as such the false-negative rate from this analysis is likely to be high: As we observed that on average 40.4% of the trait-associated SNPs affect gene expression levels in *cis*, we expect that many of these SNPs will exert effects on gene expression in *trans*. However, these effects are likely to be small and due to multiple testing issues our current study identified only a relatively small set of *trans*-eQTL effects. Likewise the number of detected converging pairs of SNPs is even smaller. However, as we observed this convergence for various pairs of SNPs, future genetical genomics studies using larger sample sizes will likely reveal many more pairs of converging SNPs, providing better insight in the downstream molecular mechanisms that are affected by these disorders.

The convergence and phenotypic buffering we observed might also help uncover some of the missing heritability in complex disease. As there are probably many SNPs with low marginal phenotypic effects [50], GWAS currently lack power to detect these. However, the effect of these trait-associated SNPs on expression seems to be less multifactorial, leading to larger expression effects. These numerous expression disturbances will eventually converge to a phenotype, explaining the small phenotypic effect of individual trait-associated SNPs.

Therefore, studying expression as intermediate phenotype will be important for disease association studies trying to account for the missing heritability of complex diseases. Disease SNPs, already found to be disease-associated and marked as eQTL, lead to a set of candidate downstream genes. Additional genetic variants that also affect the expression of these genes will therefore be powerful candidates for disease susceptibility.

## Materials and Methods

### Peripheral blood genetical genomics study populations

The peripheral blood genetical genomics study population contained 1,469 unrelated individuals from the United Kingdom and the Netherlands. Some of these are healthy controls while others are patient samples. The 49 ulcerative colitis (UC) cases in this study are part of the inflammatory bowel disease (IBD) cohort of the University Medical Centre Groningen. The 111 celiac disease samples were collected within the Barts and the London NHS Trust and the Oxford Radcliffe Hospitals NHS Trust. The 453 chronic obstructive pulmonary disease (COPD) samples were collected within the NELSON study. The 856 amyotrophic lateral sclerosis (ALS) cases and controls were collected in the University Medical Centre Utrecht. All samples were collected after informed consent and approved by local ethical review boards. Individual sample information is provided in Table S11.

Peripheral blood (2.5 ml) for all samples was collected with the PAXgene system (PreAnalytix GmbH, UK). PAXgene vials were chosen to prevent density gradient centrifugation, immortalization or in vitro cell culture artifacts changing mRNA profiles. PAXgene tubes were mixed gently and incubated at room temperature for two hours. After collection, tubes were frozen at −20°C for at least 24 hours followed by storage at −80°C. RNA was isolated using the PAXgene Blood RNA isolation kit (PreAnalytix GmbH, UK). RNA was quantified using the Nanodrop (Nanodrop Technologies, USA). Total RNA integrity was analyzed using an Agilent Bioanalyzer (Agilent Technologies, USA).

### Peripheral blood SNP genotyping

Peripheral blood samples were either genotyped using the Illumina (Illumina, San Diego, USA) HumanHap300, HumanHap370 or 610 Quad platform. Genotyping was performed according to standard protocols from Illumina. Although the different genotype oligonucleotide arrays differ, they share 294,757 SNPs, to which the analysis was confined. In addition, SNPs with a minor allele frequency of <5%, or a call-rate <95%, or deviating from Hardy-Weinberg equilibrium (exact p-value <0.001) were excluded, resulting in 289,044 SNPs for further analysis. Genotype calling for each SNP was performed by a previously described algorithm [51].

### Peripheral blood Illumina expression profiling

Anti-sense RNA was synthesized, amplified and purified using the Ambion Illumina TotalPrep Amplification Kit (Ambion, USA) following the manufacturers' protocol. Complementary RNA was either hybridized to Illumina HumanRef-8 v2 arrays (229 samples, further referred to as H8v2) or Illumina HumanHT-12 arrays (1,240 samples, further referred to as HT12) and scanned on the Illumina BeadArray Reader. Raw probe intensities were extracted using Illumina's BeadStudio Gene Expression module v3.2 (No background correction was applied, nor did we remove probes with low expression). The raw expression data of the 1,240 HT12 peripheral blood samples were combined with the raw expression data of 296 replication samples (described in detail in paragraph ‘*Trans-eQTL replication dataset*’). Both datasets (H8v2 and HT12) were quantile normalized separately to the median distribution and expression values were subsequently log_2_ transformed. Subsequently, the probes were centered to zero and linearly scaled such that each probe had a standard deviation of one.

### Integration of the Illumina H8V2 and HT12 peripheral blood expression platform identifiers

The HT12 and H8v2 arrays share a considerable number of probes with identical probe sequences. However, in a considerable number of occasions the two platforms use different probe identifiers for the same probe sequences. More importantly, although probe identifiers are often identical, they sometimes represent different probe sequences. In order to permit a meta-analysis incorporating data from both arrays, we decided on the following naming convention: if an H8v2 probe had the same sequence as an HT12 probe, the HT12 ‘ArrayAddressID’ probe identifier was used. If not, the original H8v2 probe identifier was used, but with the prefix “Human_RefSeq-8_v2-” to prevent any potential probe identifier ambiguity. A total of 52,061 unique probes were used for further analysis, representing 19,609 unique genes according to HUGO gene nomenclature.

### Initial genomic mapping of Illumina expression probe sequences

Various mapping strategies were used for the expression probes to get a mapping location that was as unambiguous as possible: if probes have been mapped incorrectly, or cross-hybridize to multiple genomic loci, it might be that an eQTL will be incorrectly deemed a *trans*-eQTL, while in fact it is a *cis*-eQTL or primer polymorphisms. We used Ensembl database version 52 (NCBI 36.3 assembly) to obtain, for each annotated gene, the transcript with the largest number of exons and included this main spliced transcript in our reference set. Second, we added one sequence per intron, extending intron boundaries 40 bp on each side to allow mapping of the 50 bp probe sequences that overlapping exon-intron junctions. Last, a version of the reference DNA genome with masked annotated transcripts was included. Probe sequences were mapped using NOVOALIGN V2.05.12 for all the sequences (main transcript, introns, and non standard exon-exon junctions) originating from the same transcript (parameters −t 150 −v 20 20 200 [>]( [ ^_]*)_). For each probe it was determined whether it was mapping uniquely to one particular genomic locus, or, if multiple hits were present whether all these mappings resided in each other vicinity (<250 kb). Probes that did not map at all, or mapped to multiple different loci were excluded from further analyses. Using this approach, 43,202 of the 48,751 probes on the HT12 and 21,316 of the 22,185 probes on the H8v2 platform were eventually mapped to a single genomic location.

### eQTL mapping

In order to detect *cis*-eQTLs, analysis was confined to those probe-SNP combinations for which the distance from the probe transcript midpoint to SNP genomic location was ≤250 kb. For *trans*-eQTLs, analysis was confined to those probe-SNP combinations for which the distance from probe transcript midpoint to SNP genomic location was ≥5 Mb (to exclude the possibility of accidentally detecting cis-eQTLs due to long ranging linkage disequilibrium). Additionally, for the *trans-*eQTL analysis the effects of the significant *cis-*eQTLs were removed from the expression data by keeping the residual expression after linear regression.

Association for *cis*- and *trans*-eQTL was tested with a non-parametric Spearman's rank correlation. For directly genotyped SNPs we coded genotypes as 0, 1 or 2, while for imputed SNPs we used SNP dosage values, ranging between 0 and 2. When a particular probe-SNP pair was present in both the HT12 and H8v2 datasets, an overall, joint p-value was calculated using a weighted (square root of the dataset sample number) Z-method.

To correct for multiple testing, we controlled the false-discovery rate (FDR) at 0.05: the distribution of observed p-values was used to calculate the FDR, by comparison with the distribution obtained from permuting expression phenotypes relative to genotypes 100 times within the HT12 and H8v2 dataset for both the *cis*- and *trans*- analyses [52].

In order to increase the number of detectable *cis*- and *trans*-eQTLs we applied a principal component analysis (PCA) on the sample correlation matrix. We, among others [19], [20], argue that the dominant PCs, capturing the larger part of the total variation, will primarily capture sample differences in expression that reflect physiological or environmental variation as well as systematic experimental variation (e.g. batch and technical effects). Figure S6 shows for the 1,240 HT12 samples what per individual the PC scores are. It is evident there are, especially among the first PCs, strong batch effects are still present after proper quantile-quantile normalization. By removing the variation captured by these PCs, we expected that the residual expression is more strongly determined by genetic variants and the number of significantly detected *cis*- and *trans*-eQTLs will increase. An aspect to consider is that with the removal of more PCs from the data, the degrees of freedom of the data will decrease. Furthermore, it is not immediately clear which PCs will actually capture physiological, environmental, and systematic variation, which might lead to removal of genetically determined expression variation as well. Therefore a tradeoff has to be made on the number of PCs to subtract from the data. We assessed this systematically, by removing up to 100 PCs from the genetical genomics dataset (in steps of 5).

Figure S7A shows that the number of significantly detected *cis*-eQTL probes increases two-fold when 50 PCs were removed from the expression data. There is a long plateau visible (around PC50), where the number of detected *cis*-eQTLs probes remains approximately constant, irrespective of removing for instance 10 fewer or 10 extra PCs (reported numbers in this figure also include false-positive eQTLs due to potential primer polymorphisms, as we here wanted to solely compare the performance of removing different numbers of PCs). Figure S7B shows that of the initial 5,950 significantly detected *cis*-eQTL probes (no PCs removed), 4,965 (83.5%) were still detected with 50 PCs subtracted. The 985 initially detected *cis*-eQTLs probes, yet no longer detected when 50 PCs had been removed from the expression data, all had a low significance (Figure S8). As we controlled the FDR at 0.05 in all analyses it is therefore likely that a considerable amount of these reflect false-positives. Figure S8C shows that for all the overlapping 4,965 detected *cis*-eQTLs probes between the different analyses, the allelic direction was identical, and effect size on expression correlate well (Pearson r = 0.95) although these were nearly always stronger after having subtracted 50 PCs.

We assessed this for *trans*-eQTLs as well. An important aspect to consider is that *trans*-eQTL SNPs might affect multiple genes. If these effects are substantial (either in effect size or the number of affected genes), it is likely that a certain PC will capture this. Removal of such PCs from the expression data will therefore unintentionally result in the inability to detect these *trans*-eQTLs. In order to avoid such false-negative*s* we first performed a QTL analysis on the first 50 PCs (that had been removed from the expression data for the *cis*-eQTL analysis) to assess whether some of these PCs are under genetic control (genome-wide analysis, controlling FDR at 0.05). We did this for the large HT12 and the smaller H8v2 expression data separately, as PCA had been applied independently to these datasets. We observed that out of the first 25 PCs in the HT12 data three PCs and in the H8v2 two PCs were to some extent genetically determined (r^2^>5%). This was different for PCAs 26–50 in the HT12 data: 11 PCs were under substantial genetic control (Figure S9a).

We therefore assumed that most *trans*-eQTLs could be detected when removing approximately 25 PCs. We quantified this systematically, by removing increasing amounts of PCs from the expression data and conducting a full genome-wide *trans*-eQTL mapping. Indeed, in these analyses at most 244 significant *trans*-eQTLs could be detected (at FDR 0.05, with potential false-positives due to cross-hybridizations removed), when removing 25 PCs (Figure S9b). The overlap with the expression with no PCs removed was substantial: 62 of the 82 *trans*-eQTLs (77%), detected in the original analysis were detected as well in the analysis with 25 PCs removed (Figure S9c), all with identical allelic directions (Figure S9d).

### Identification of false eQTLs due to primer polymorphisms and cross-hybridization

One should be aware that sequence polymorphisms can cause many false *cis*-eQTLs [53]. Such false *cis*-eQTLs do not reflect actual expression differences caused by sequence polymorphisms in *cis*-acting factors that affect mRNA levels. Instead they indicate hybridization differences caused by sequence polymorphisms in the mRNA region that is targeted by the microarray expression probes. Therefore, SNP-probe combinations were excluded from the *cis*-eQTL analysis when the 50 bp long expression probe mapped to a genomic location that contained a known SNP that was showing at least some LD (r^2^>0.1) with the *cis*-SNP. We used SNP data from the 1000 Genomes Projects, as it contains LD information for 9,633,115 SNPs (April 2009 release, based on 57 CEU samples of European descent).

Detected *trans*-eQTLs might also reflect false-positives, although we initially had attempted to map the expression probes as accurately as possible, by using the aforementioned three different mapping strategies: it is still well possible that some of the identified, putative *trans*-eQTLs in fact reflect very subtle cross-hybridization (*e.g.* pertaining to only a small subsequence of the probe). We therefore tried to falsify each of the putative *trans*-eQTLs by attempting to map each *trans*-probe into the vicinity of the SNP probe location, by using a highly relaxed mapping approach. All putative Illumina *trans*-expression probes were mapped using SHRiMP [54], which uses a global alignment approach, to the human reference genome (NCBI 36.3 build). The mapping settings were chosen very loosely to permit the identification of nearly all potential hybridization locations: match score was 10, the mismatch score was 0, the gap open penalty was −250, the gap extension penalty was −100, Smith and Waterman minimum identical alignment threshold was 30.0%, while other SHRiMP parameters were left at default. Using these settings all mappings with a minimum overlap of 15 bases, or with 20 matches with one mismatch, or 30 matches with 2 mismatches, or full-length (50 bp) probe hybridizations with no more than 15 mismatches were accepted. Any *trans*-eQTL was discarded, if the expression probe had a mapping that was within 2 Mb of the SNP that showed the *trans*-eQTL effect. Once these potential false-positive *trans*-eQTLs had been removed from the real, non-permuted data, we repeated the multiple testing correction (again controlling the FDR at 0.05).

Using this strategy we observed several instances where only 20 out the 50 bases of a probe sequence mapped in the vicinity of the *trans-*SNP (data not shown). For these *trans*-eQTLs the Spearman's rank correlation p was often lower than 10^−100^, which would imply these SNPs explain over 25% of the total expression variation of the corresponding *trans*-genes. Given the small amount of *trans*-eQTLs we detected in total, such effect sizes are quite unlikely and therefore provide circumstantial evidence these indeed reflect cross-hybridization artifacts.

We also assessed whether any of the Illumina SNPs that constitute *trans*-eQTLs might map to a different position than what is reported in dbSNP. As such we mapped the 50 bp Illumina SNP probe sequences to the genome assembly, permitting up to four mismatches per 50 bp SNP probe sequence. We did not observe any SNP that could map (with some mismatches) to the same chromosome of the *trans*-probe.

It is still possible that some of the *trans*-eQTLs for which we did not find any evidence of cross-hybridization, still are false positives, e.g. by missing some cross-hybridizations due to imperfections in the NCBI v36 assembly we used. Although we have identified numerous occasions where a SNP affects two different probes within the same gene in *trans*, substantiating the likelihood these *trans*-eQTLs are real, providing unequivocal evidence that all our reported *trans*-eQTLs are real is not straightforward.

### Enrichment analysis of trait-associated SNPs and SNPs located within the HLA region

To assess enrichment of trait-associated SNPs, we used a collection of 1,262 unique SNPs from 'A Catalog of Published Genome-Wide Association Studies' (accessed 09 February 2010, and each having at least one reported association p-value <5.0×10^−7^). We could successfully impute the genotypes for 1,167 of these SNPs and therefore confined all analyses to these SNPs. Of these SNPs 572 had been directly genotyped on the Illumina HumanHap300 platform, with a MAF>0.05, an HWE exact p-value >0.0001 and call-rate >95%.

To ascertain whether these SNPs are more often constituting an eQTL than expected, we used a methodology that is not affected by the following potential confounders: non-even distribution of SNP markers and expression probe markers across the genome, differences in MAF between SNPs and LD structure within the genotype date and correlation between probes in the expression data. Additionally, this methodology is also not confounded by the fact that for certain traits different SNPs in strong LD can have been reported, due to differences in the platforms that were used to identify these loci.

We first determined how many unique eQTL SNPs had been identified in the original eQTL mapping (with an FDR<0.05) and how many of these are trait-associated. Subsequently we permuted the expression phenotypes relative to the genotypes (thus keeping the correlation structure within the genotype data and the correlation structure within the expression data intact, yet assigning the genotypes of a sample to the expression data of a randomly chosen sample) and reran the eQTL mapping, sorting all tested eQTLs on highest significance. We then took an equal number of top associated, but permuted, eQTL SNPs and determined how many of these permuted eQTL SNPs are trait-associated. By performing 100 permutations we obtained an empiric distribution of the number of trait-associated SNPs expected by chance. We subsequently fitted a generalized extreme value distribution (EVD, using the EVD add-on package for R), permitting us to estimate realistic enrichment significance estimates (called EVD p throughout the manuscript).

For the MHC enrichment analysis the followed procedure was identical, with the difference that we looked for enrichment for SNPs within the MHC, defined as SNPs physically mapping between 20 Mb and 40 Mb on chromosome 6 (NCBI 36 assembly).

### *Trans*-eQTL replication datasets

Replication of the detected eQTLs was performed in monocytes from 1,490 different samples [45] and in an independent population of 86 morbidly obese individuals that underwent elective bariatric surgery (Department of general surgery, Maastricht University Medical Centre, the Netherlands). Both these datasets also used the same Illumina HumanHT-12 expression platform.

For the 1,490 monocyte samples eQTL P-Values summary statistics were available for all monocyte *trans*-eQTLs with a nominal p<1.0×10^−5^. We ascertained how many of the *trans*-eQTLs we had found in our peripheral blood data had a nominal eQTL p<1.0×10^−5^ in this monocyte dataset.

We also assessed *trans*-eQTLs in four different tissues from the 86 morbidly obese individuals that underwent bariatric surgery. DNA was extracted from blood samples using the Chemagic Magnetic Separation Module 1 (Chemagen) integrated with a Multiprobe II Pipeting robot (PerkinElmer). All samples were genotyped using both Illumina HumanCytoSNP-12 BeadChips and Illumina HumanOmni1-Quad BeadChips (QC was identical as was applied to the peripheral blood samples). We imputed HapMap 2 genotypes using Impute version 2.0. In addition expression profiling was performed for four different tissues for each of these individuals using the Illumina HumanHT-12 arrays. Wedge biopsies of liver, visceral adipose tissue (VAT, omentum majus), subcutaneous adipose tissue (SAT, abdominal), and muscle (musculus rectus abdominis) were taken during surgery. RNA was isolated using the Qiagen Lipid Tissue Mini Kit (Qiagen, UK, 74804). Assessment of RNA quality and concentration was done with an Agilent Bioanalyzer (Agilent Technologies USA). Starting with 200 ng of RNA, the Ambion Illumina TotalPrep Amplification Kit was used for anti-sense RNA synthesis, amplification, and purification according to the protocol provided by the manufacturer (Ambion, USA). 750 ng of complementary RNA was hybridized to Illumina HumanHT12 BeadChips and scanned on the Illumina BeadArray Reader. Expression data preprocessing was as mentioned before. We first attempted to replicate the trait-associated *trans*-eQTLs per tissue, using an FDR of 0.05 and 100 permutations. Subsequently we conducted a meta-analysis, combining the four tissues. Per *trans*-eQTL we used a weighted Z-method to combine the four individual p-values. However, these four datasets are not independent, as they reflect the same individuals. We resolved this by conducting the permutations in such a way that in every permutation round the samples were permuted in exactly the same way for each of the four tissues. By doing this we retained the correlations that exist between the different tissues per sample, and were able to get a realistic empiric (null-)distribution of expected test-statistics.

### Convergence analysis

Per trait we assessed all the SNPs that have been reported to be associated with that particular trait. We analyzed per trait all possible SNP-pairs. If a pair of SNPs was not in LD (r^2^<0.001) we assessed whether they affected the same gene in *cis* or *trans*. When using the trait-associated *cis*- and *trans*-eQTLs that had been identified when controlling the FDR at 0.05, we identified 7 unique pairs of SNPs that caused both the same phenotype and also affected the same gene(s). When using a somewhat more relaxed set of *trans*-eQTLs, identified when controlling the FDR at 0.5, we identified 18 unique pairs of SNPs that affect the same downstream gene.

We assessed whether these numbers were significantly higher than expected, by using the same strategy that we had used to assess the enrichment of trait-associated SNPs and the HLA; we ran 100 permutations. We kept per permutation the *cis*-eQTL list as it was, but generated a permuted set of *trans*-eQTLs, equal in size to the original set of non-permuted *trans*-eQTLs. This enabled us to determine per permutation round how many unique pairs of SNPs converge on the same gene(s). We subsequently fitted a generalized extreme value distribution, permitting us to estimate realistic enrichment significance estimates.

### Co-expression between genes, based on HT12 peripheral blood co-expression

If a particular SNP is *cis*- or *trans*-acting on multiple genes, it is plausible that those genes are biologically related. Co-expression between these genes provides circumstantial evidence this is the case, strengthening the likelihood such *cis*- and *trans*-eQTLs are real. We assessed this in the peripheral blood data, by using the expression data of the 1,240 samples, run on the comprehensive HT12 expression platform. As we had removed 25 PCs (to remove physiological, environmental variation, and systematic experimental variation) for the *trans*-eQTL analyses, we decided to confine co-expression analyses to this expression dataset. As there are 43,202 HT12 probes that we mapped to a known genomic location, 43,202×43,201/2 = 933,184,801 probe-pairs exist. Given 1,240 samples, a Pearson correlation coefficient r≥0.19 corresponds to a p<0.05 when applying stringent Bonferroni correction for these number of probe-pairs.

### Accession numbers

Expression data for both the peripheral blood and the four non-blood datasets have been deposited in GEO with accession numbers GSE20142 (1,240 peripheral blood samples, hybridized to HT12 arrays), GSE20332 (229 peripheral blood samples, hybridized to H8v2 arrays) and GSE22070 (subcutaneous adipose, visceral adipose, muscle and liver samples).

## Supporting Information

Figure S1 Detected *cis*- and *trans*-eQTLs in genome-wide analysis.
(TIF)

Figure S2 Detected *cis*- and *trans*-eQTLs for 1,167 trait-associated SNPs.
(TIF)

Figure S3 Detected *cis*- and *trans*-eQTLs per complex trait. Immune-related and hematological associated SNPs often affect gene expression in *cis* or *trans*.
(TIF)

Figure S4 Co-expression distribution between eQTL genes for mean platelet volume and mean corpuscular volume.
(PDF)

Figure S5 Replication of *trans*-eQTLs in four non-blood tissues.
(TIF)

Figure S6 Principal components used as covariates in analyses.
(PDF)

Figure S7 Effect of removing principal components from expression data on detect ability of *cis*-eQTLs.
(TIF)

Figure S8 Significance of detected *cis*-eQTLs before and after removal of principal components from expression data.
(TIF)

Figure S9 Effect of removing principal components from expression data on detect ability of *trans*-eQTLs.
(TIF)

Table S1 Detected *cis*-eQTLs (FDR 0.05) for all common SNPs.
(XLSX)

Table S2 Detected *trans*-eQTLs (FDR 0.05) for all common SNPs.
(XLS)

Table S3 Detected *cis*-eQTLs (FDR 0.05) for 1,167 trait-associated SNPs.
(XLS)

Table S4 Detected *trans*-eQTLs (FDR 0.05) for 1,167 trait-associated SNPs.
(XLS)

Table S5 Detected *cis-* and *trans*-eQTLs (FDR 0.05) per complex trait.
(XLS)

Table S6 Plots of detected *trans*-eQTLs for 1,167 trait-associated SNPs for each of the seven individual cohorts of samples that make up the total of 1,469 peripheral blood samples.
(PDF)

Table S7 Detected *trans*-eQTLs (FDR 0.50) for 1,167 trait-associated SNPs.
(XLS)

Table S8 Replicated *trans*-eQTLs in monocyte eQTL dataset.
(XLS)

Table S9 Characteristics of subcutaneous adipose, visceral adipose, muscle and liver datasets.
(XLS)

Table S10 Replicated *trans*-eQTLs in subcutaneous adipose, visceral adipose, muscle and liver datasets.
(XLS)

Table S11 Characteristics of peripheral blood expression data.
(XLS)
