# Supplementary figures and images for: Trans-eQTLs Reveal That Independent Genetic Variants Associated with a Complex Phenotype Converge on Intermediate Genes, with a Major Role for the HLA
Source: PLoS Genet. 2011 Aug 4;7(8):e1002197. doi: 10.1371/journal.pgen.1002197 (PMC3150446; doi:10.1371/journal.pgen.1002197)

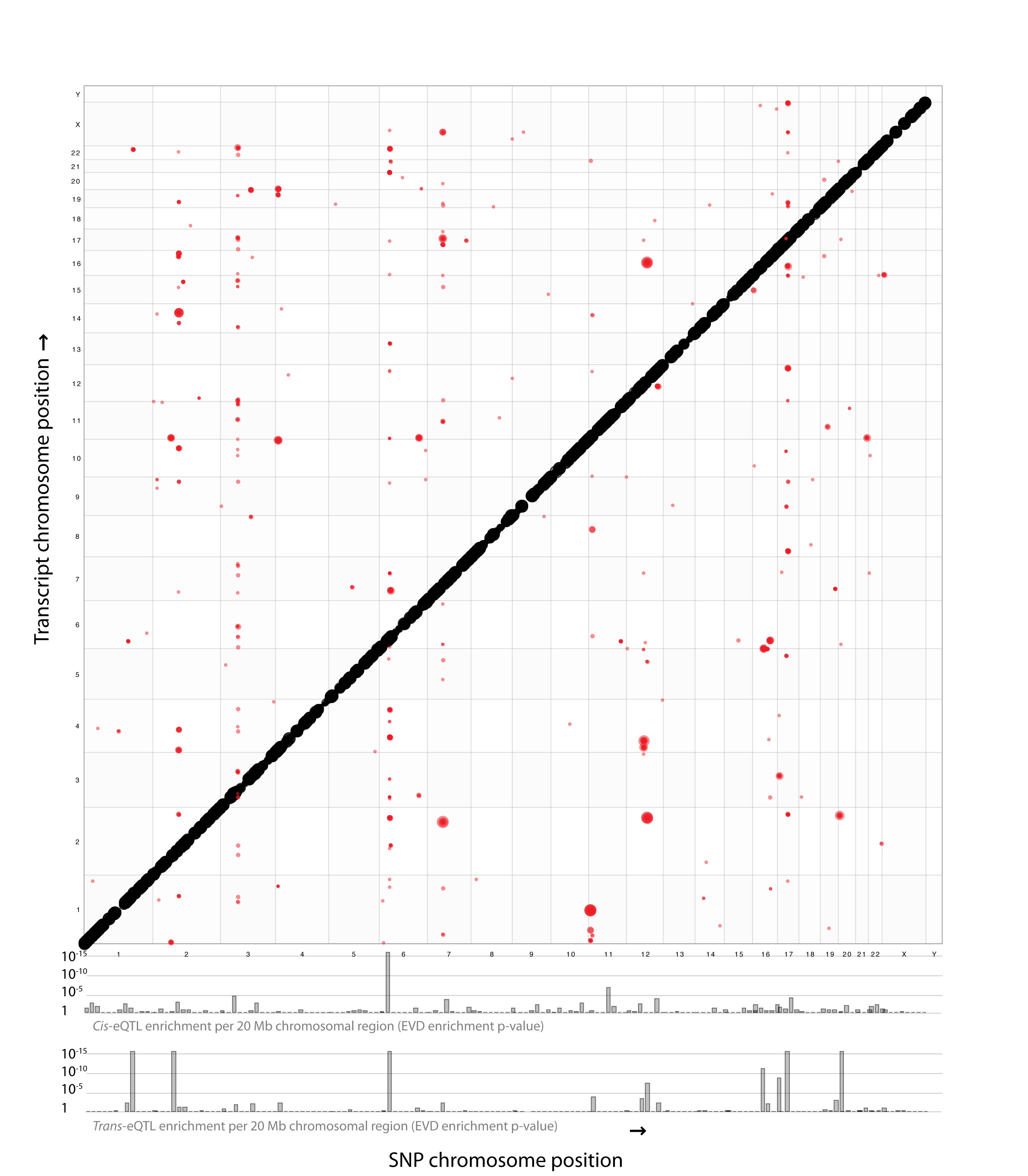

Supplement: Figure S1 — Detected cis- and trans-eQTLs in genome-wide analysis. (TIF) [file pgen.1002197.s001.tif]

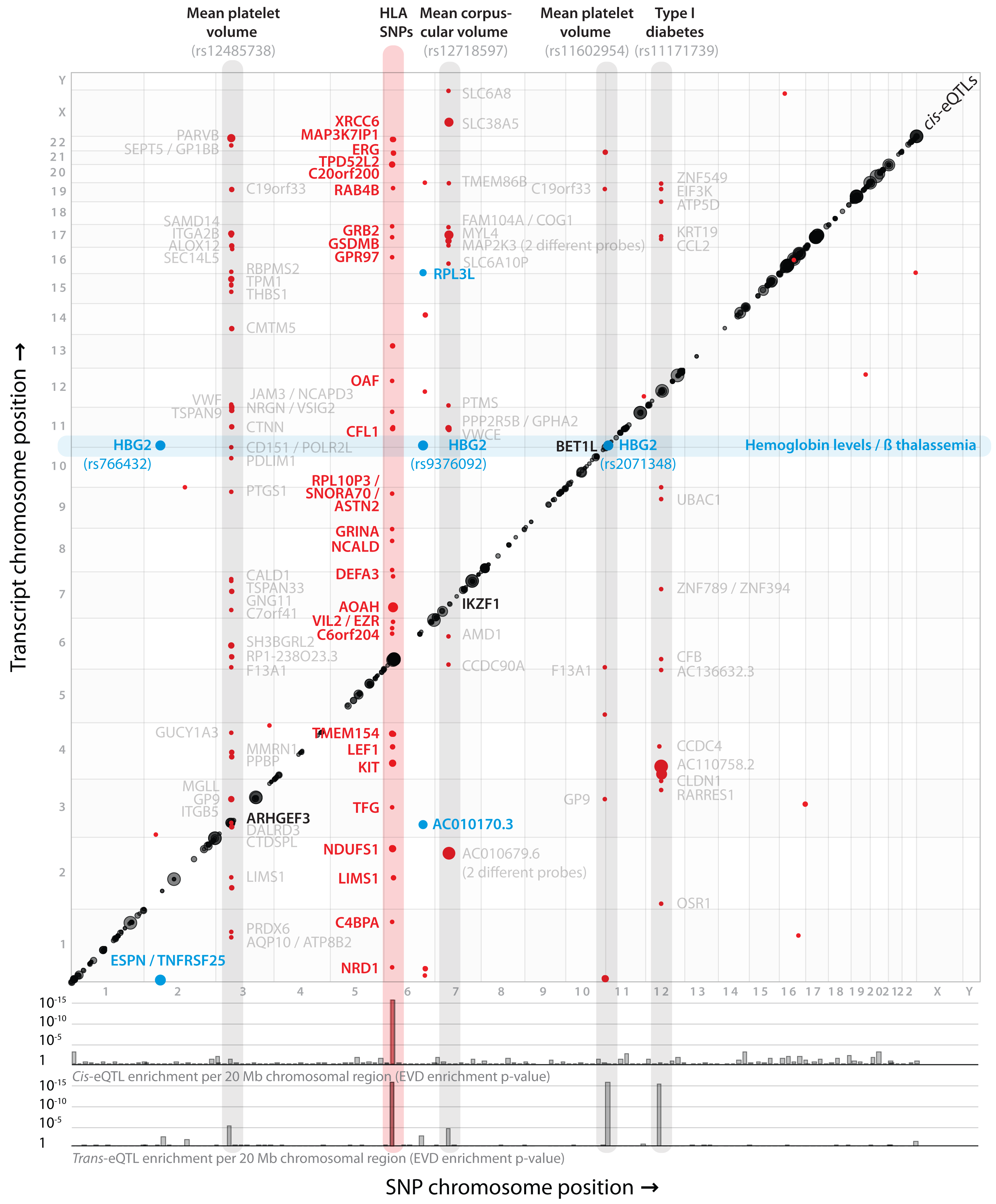

Supplement: Figure S2 — Detected cis- and trans-eQTLs for 1,167 trait-associated SNPs. (TIF) [file pgen.1002197.s002.tif]

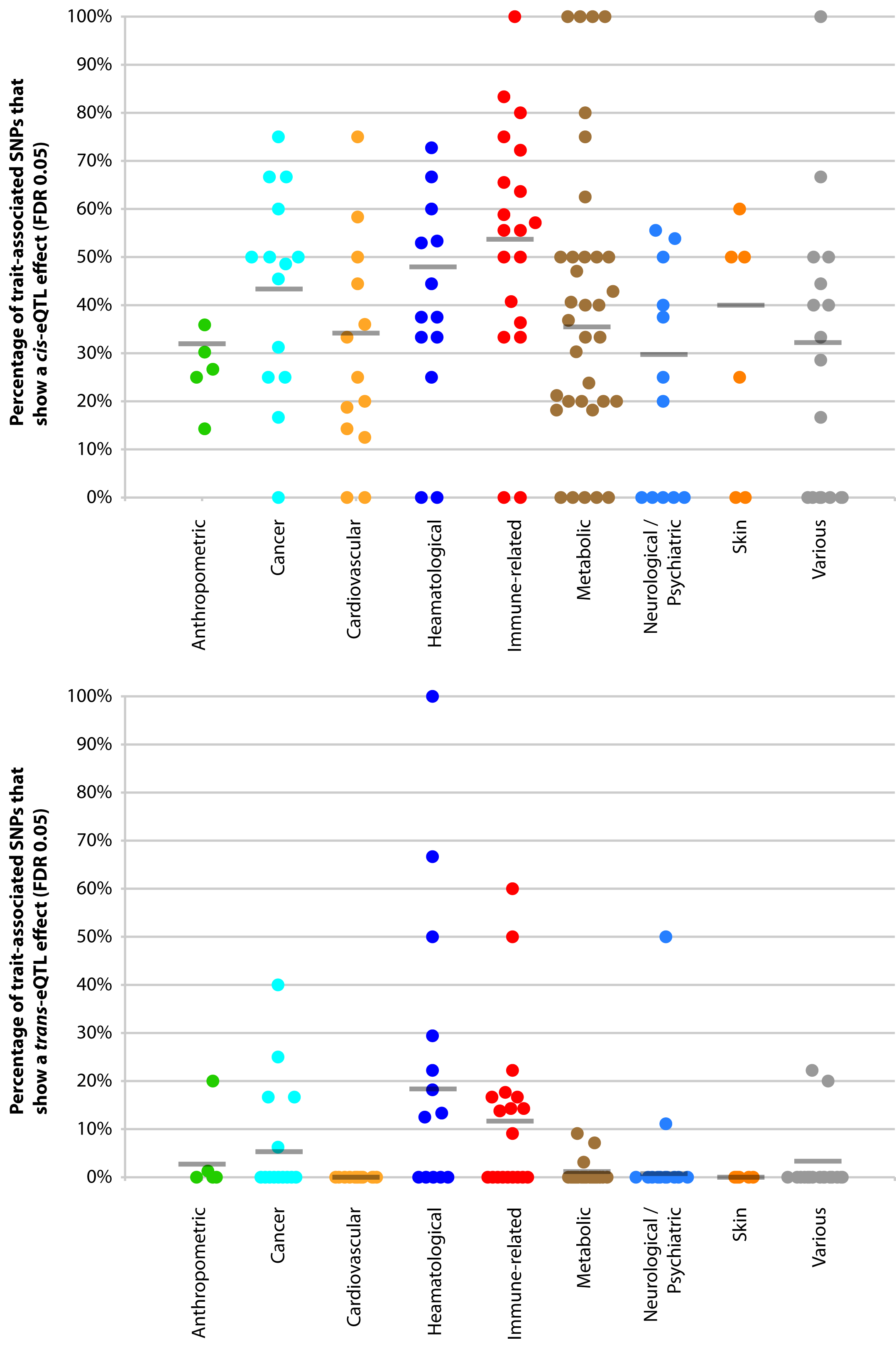

Supplement: Figure S3 — Detected cis- and trans-eQTLs per complex trait. Immune-related and hematological associated SNPs often affect gene expression in cis or trans. (TIF) [file pgen.1002197.s003.tif]

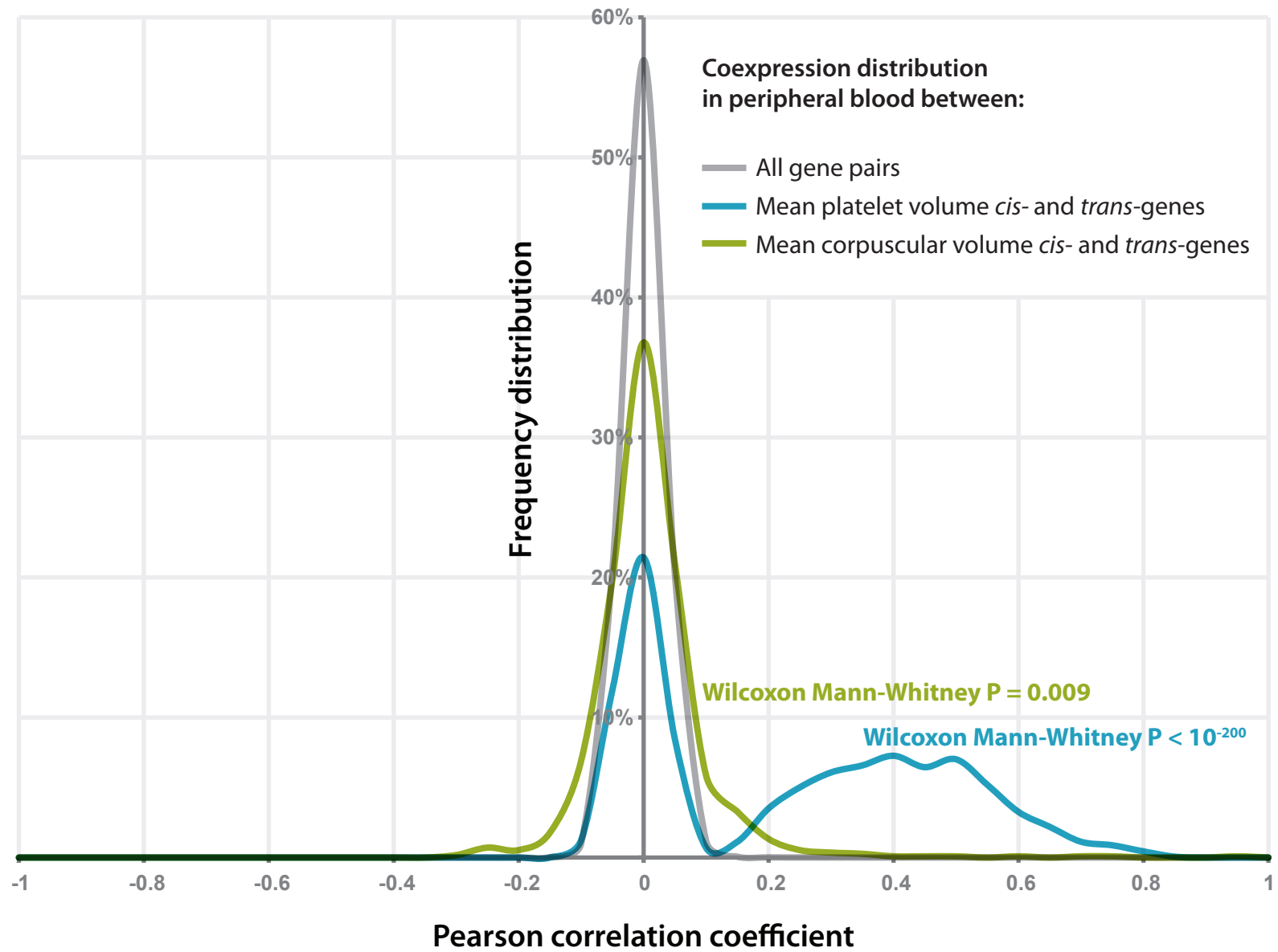

Supplement: Figure S4 — Co-expression distribution between eQTL genes for mean platelet volume and mean corpuscular volume. (PDF) [file pgen.1002197.s004.pdf]

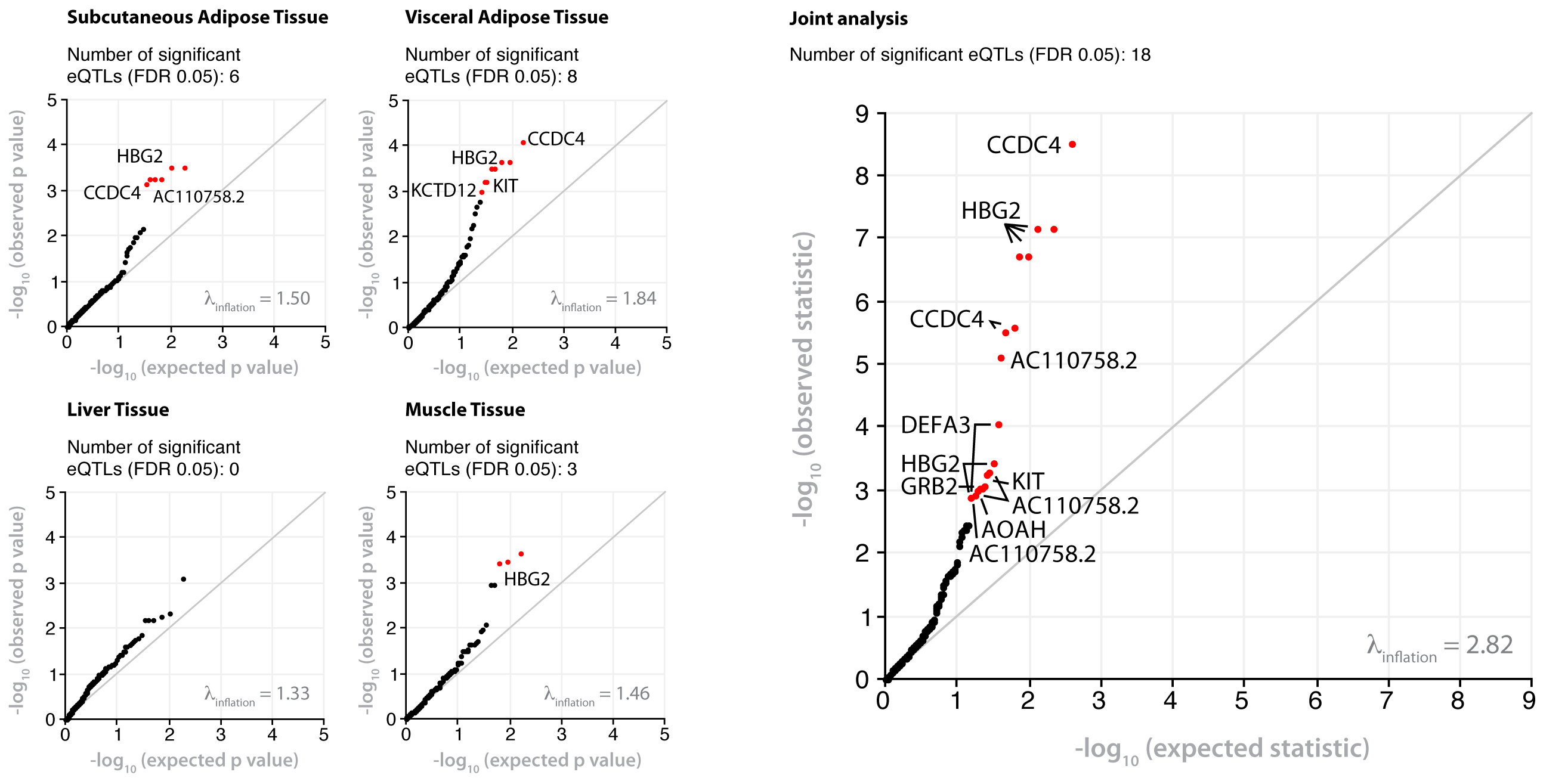

Supplement: Figure S5 — Replication of trans-eQTLs in four non-blood tissues. (TIF) [file pgen.1002197.s005.tif]

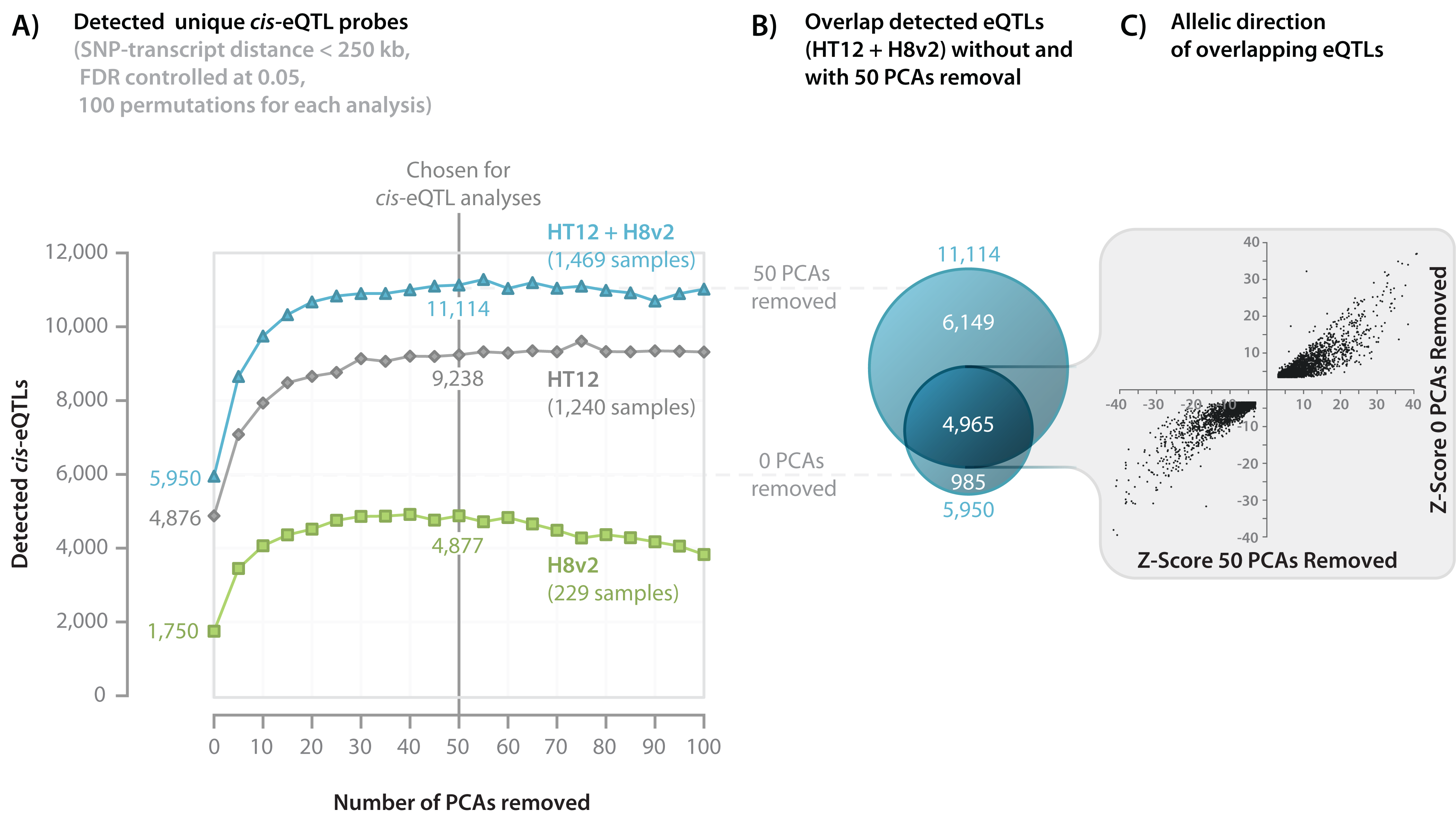

Supplement: Figure S7 — Effect of removing principal components from expression data on detect ability of cis-eQTLs. (TIF) [file pgen.1002197.s007.tif]

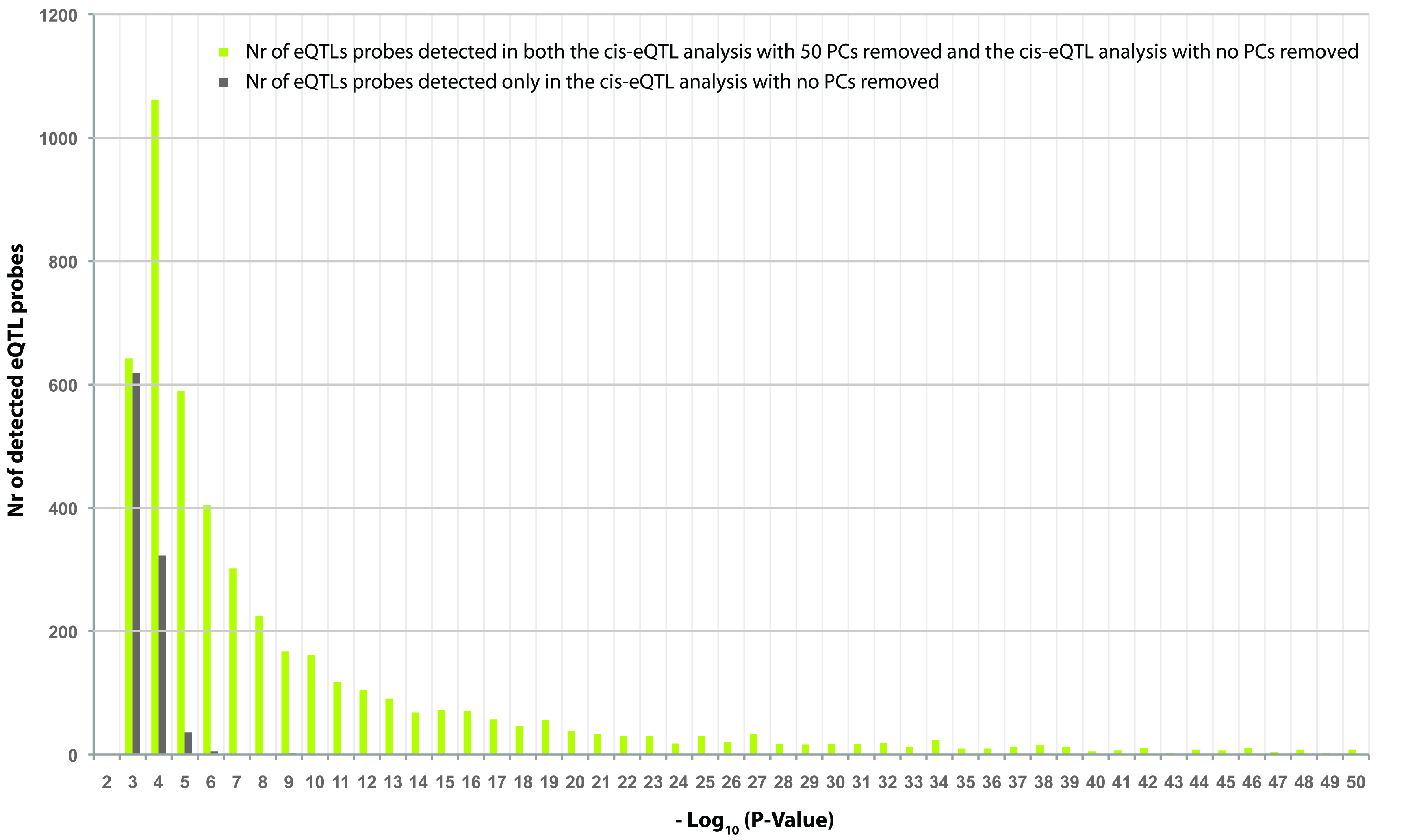

Supplement: Figure S8 — Significance of detected cis-eQTLs before and after removal of principal components from expression data. (TIF) [file pgen.1002197.s008.tif]

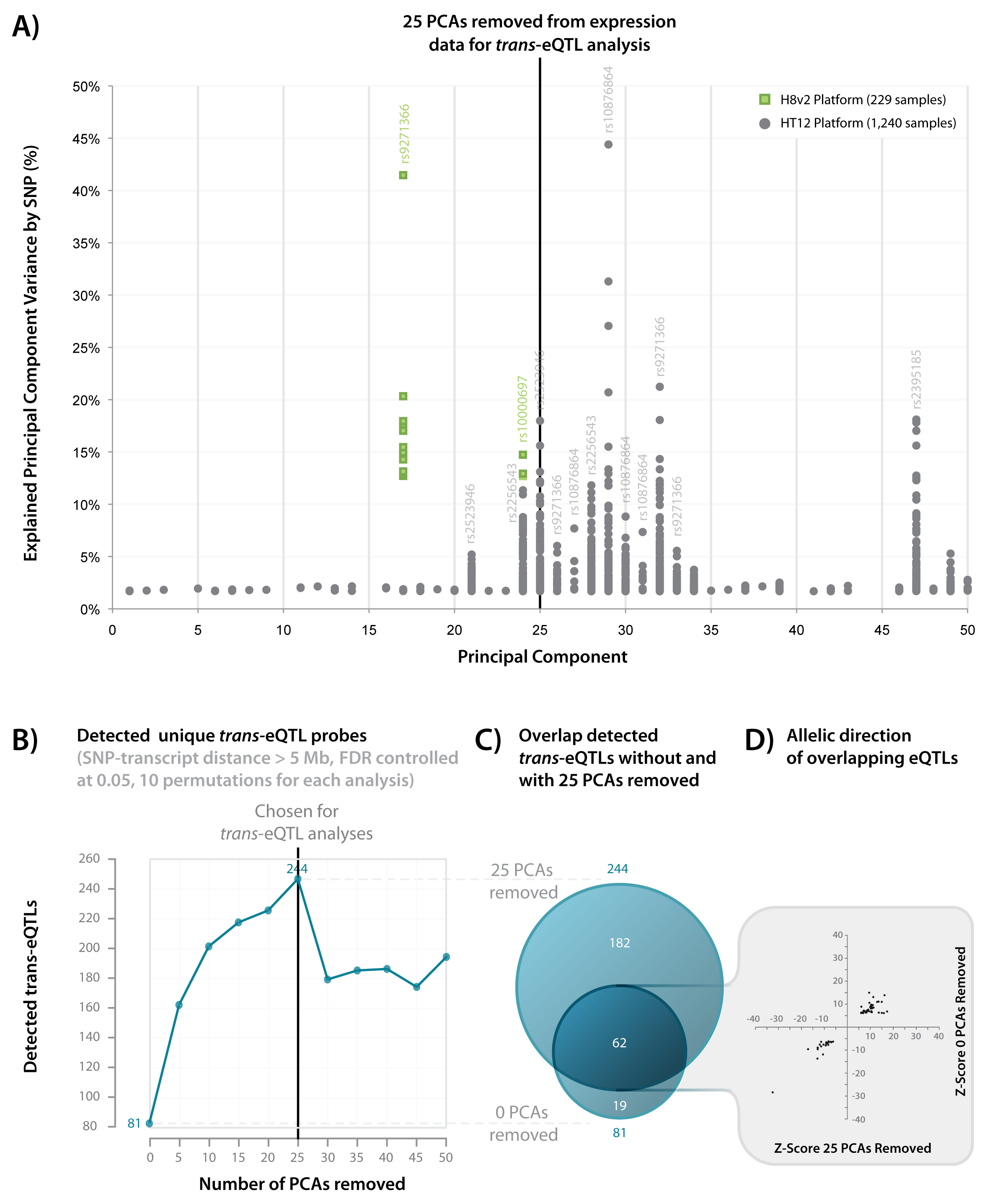

Supplement: Figure S9 — Effect of removing principal components from expression data on detect ability of trans-eQTLs. (TIF) [file pgen.1002197.s009.tif]
