# Supplementary material for: Trans-eQTLs Reveal That Independent Genetic Variants Associated with a Complex Phenotype Converge on Intermediate Genes, with a Major Role for the HLA
Source: PLoS Genet. 2011 Aug 4;7(8):e1002197. doi: 10.1371/journal.pgen.1002197 (PMC3150446; doi:10.1371/journal.pgen.1002197)
Supplement: Figure S6 — Principal components used as covariates in analyses. (PDF) [file pgen.1002197.s006.pdf]

Principal component 1

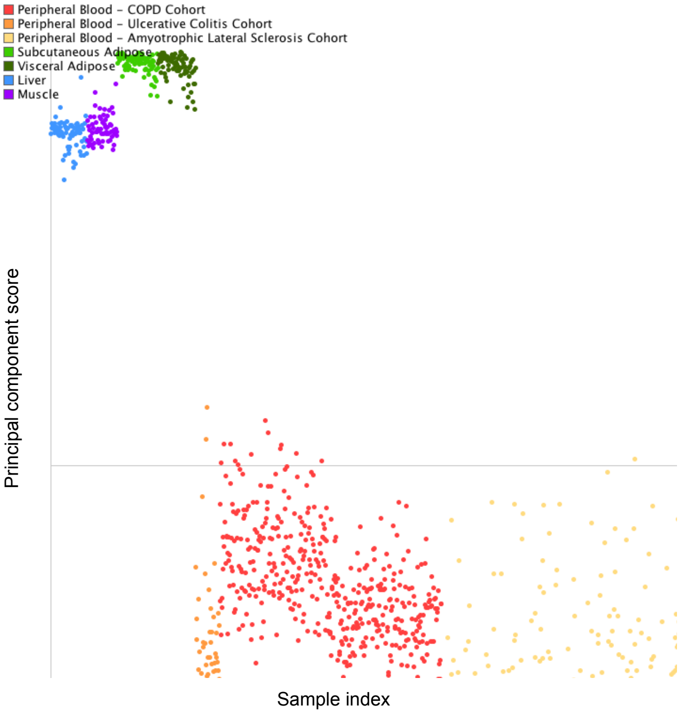

Principal component 2

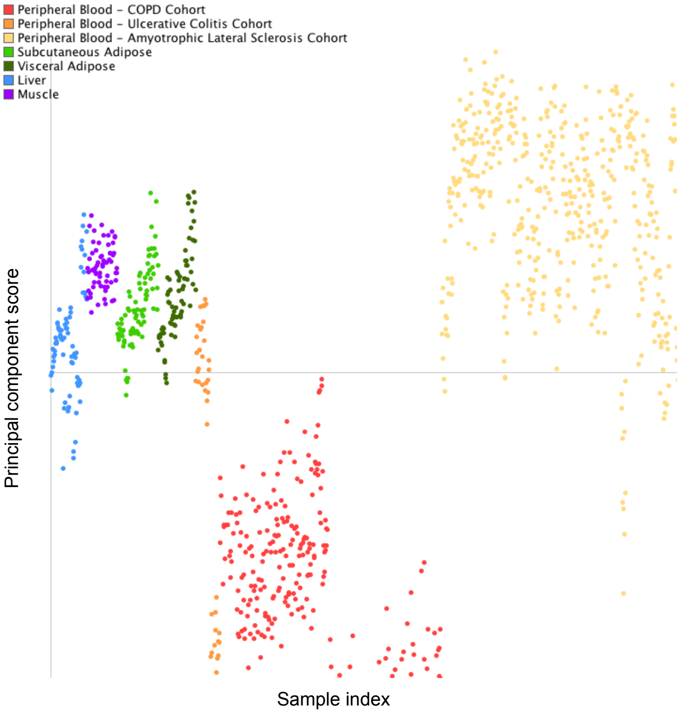

Principal component 3

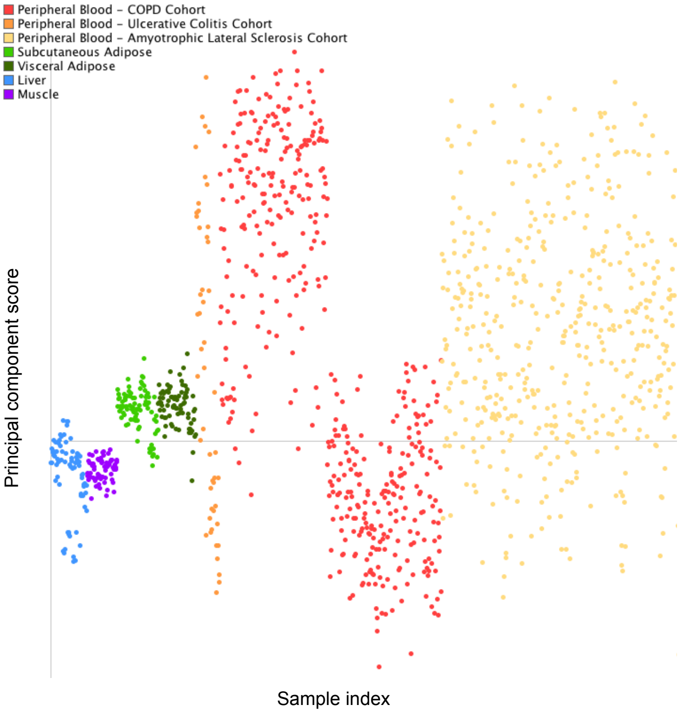

Principal component 4

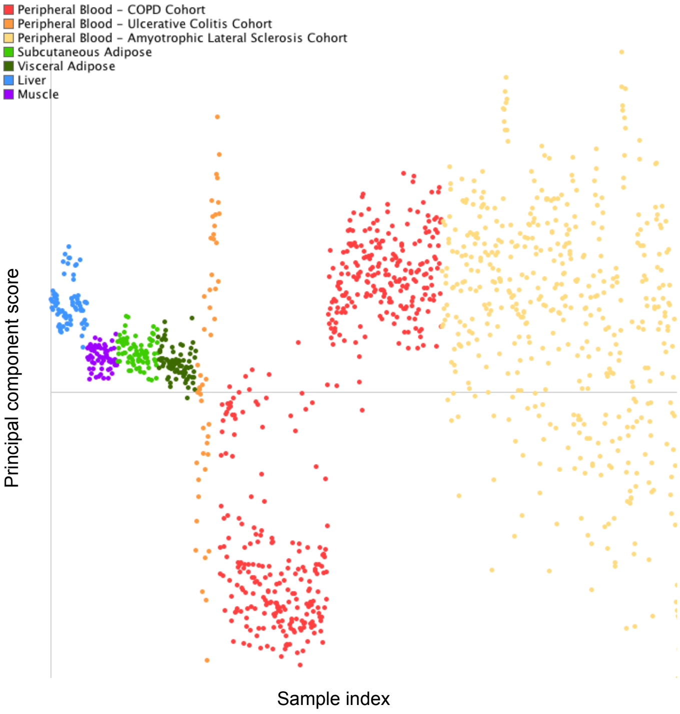

Principal component 5

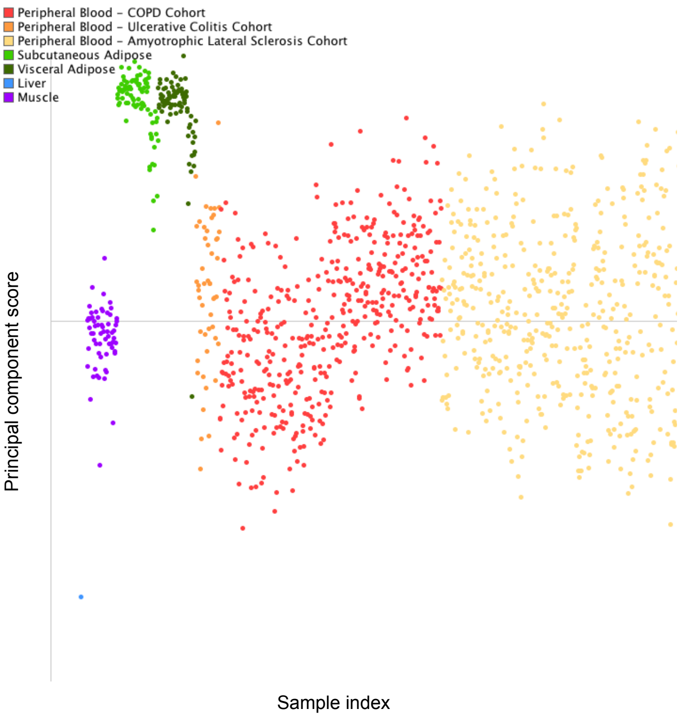

Principal component 6

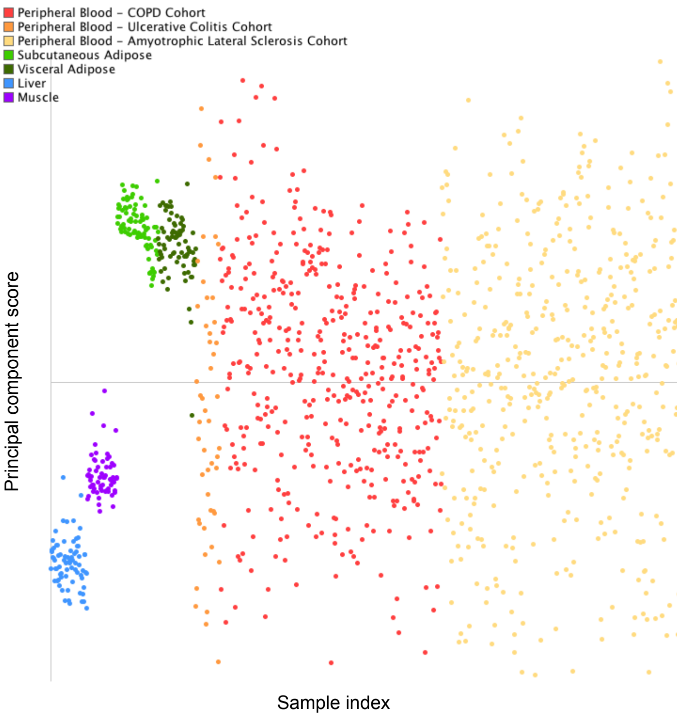

Principal component 7

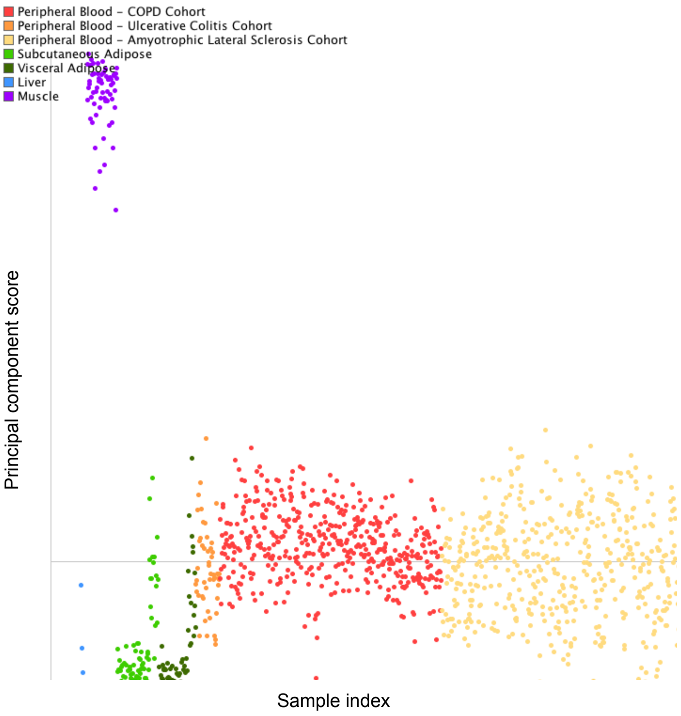

Principal component 8

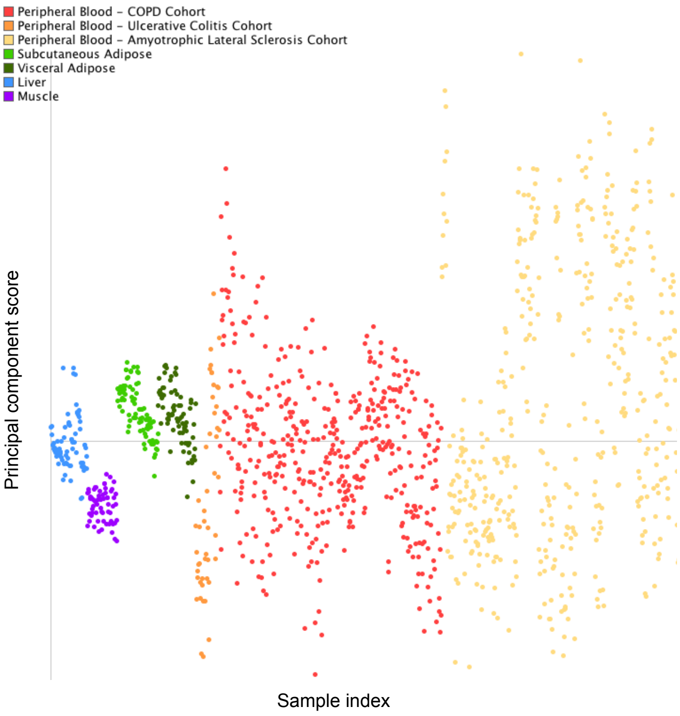

Principal component 9

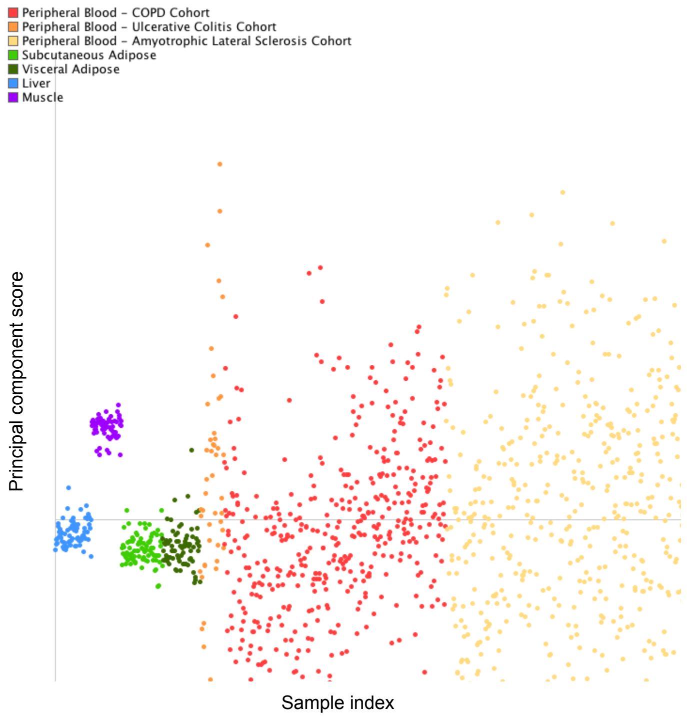

Principal component 10

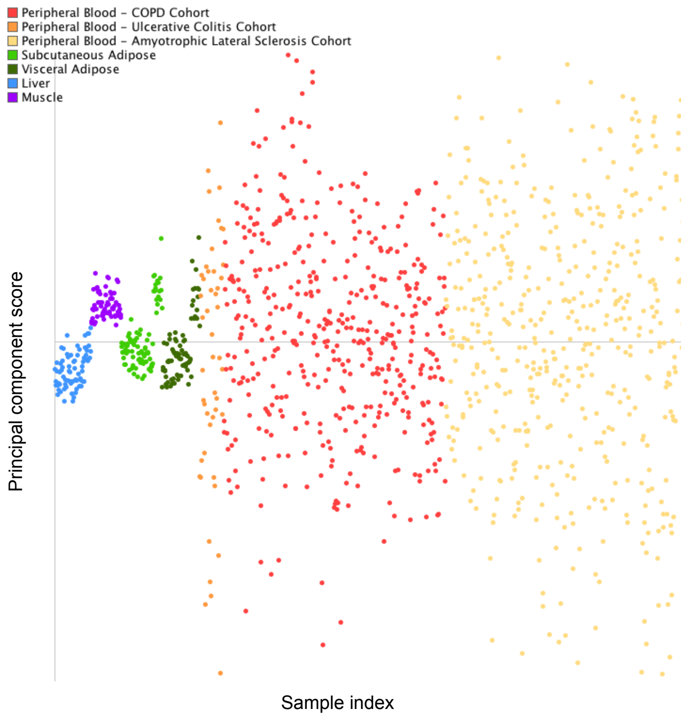

Principal component 11

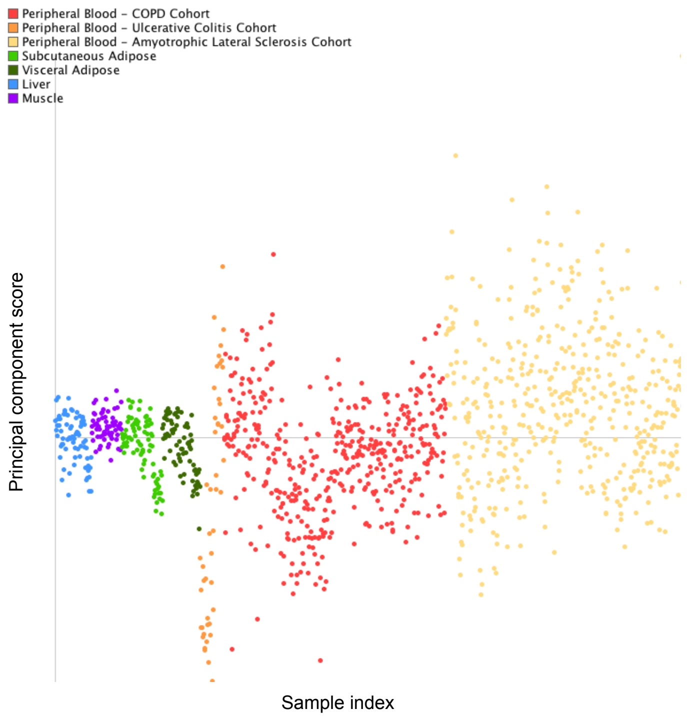

Principal component 12

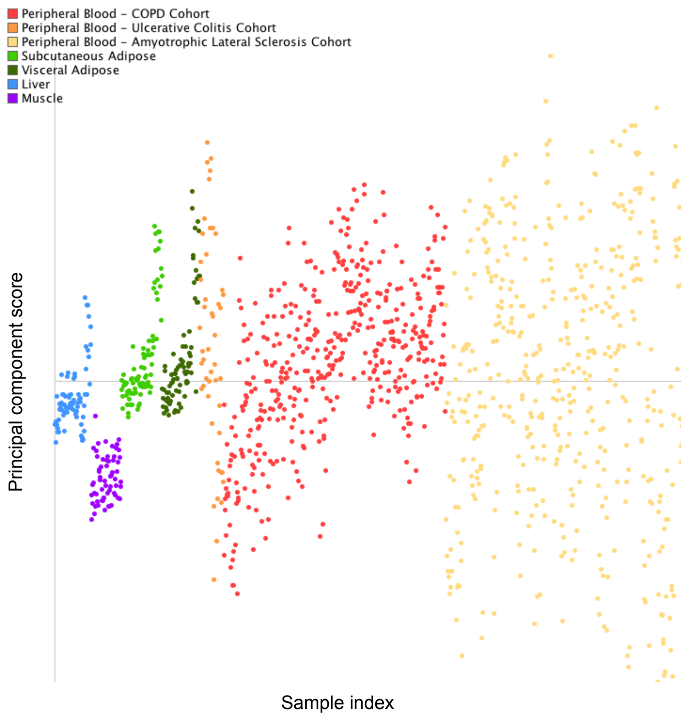

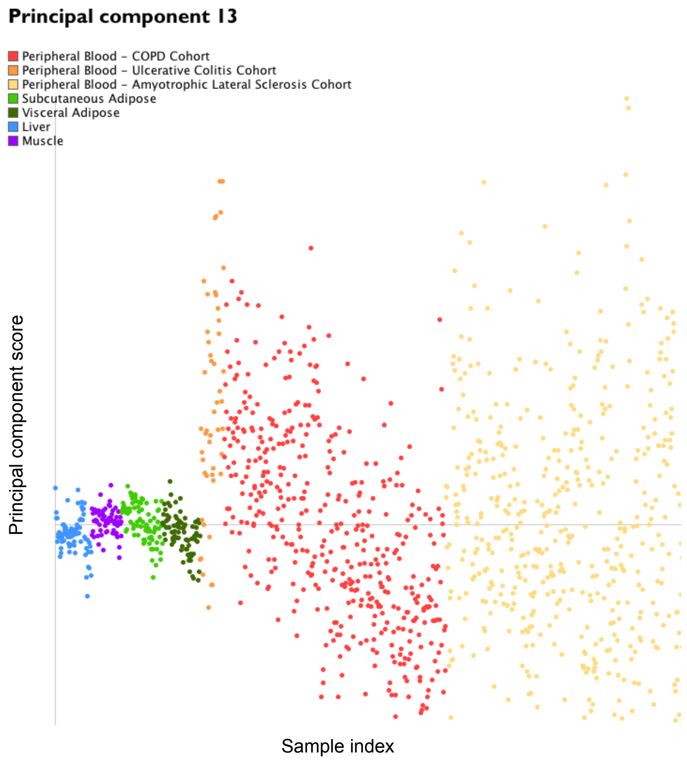
Principal component 13
Peripheral Blood - COPD Cohort
Peripheral Blood - Ulcerative Colitis Cohort
Peripheral Blood - Amyotrophic Lateral Sclerosis Cohort
Subcutaneous Adipose
Visceral Adipose
Liver
Muscle
Principal component score
Sample index

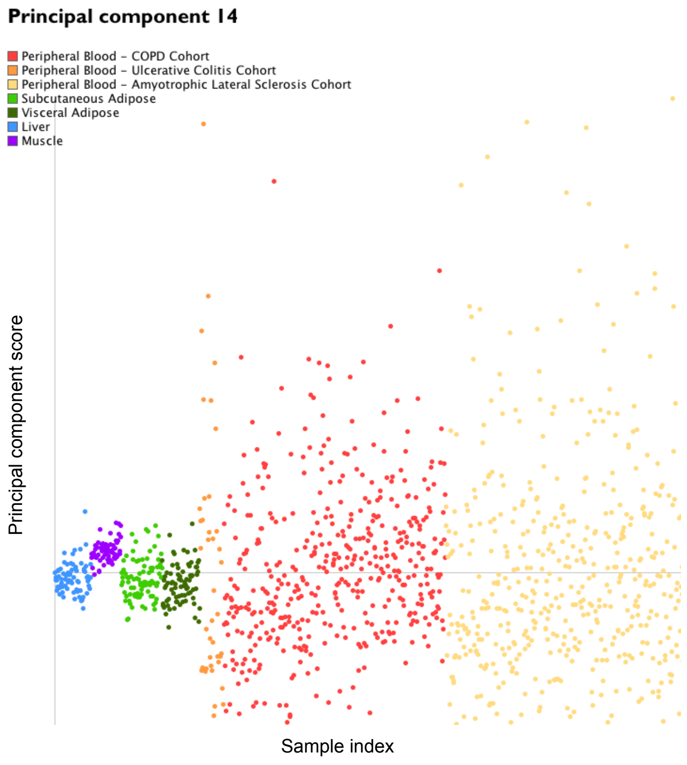
Principal component 14
Peripheral Blood - COPD Cohort
Peripheral Blood - Ulcerative Colitis Cohort
Peripheral Blood - Amyotrophic Lateral Sclerosis Cohort
Subcutaneous Adipose
Visceral Adipose
Liver
Muscle
Principal component score
Sample index

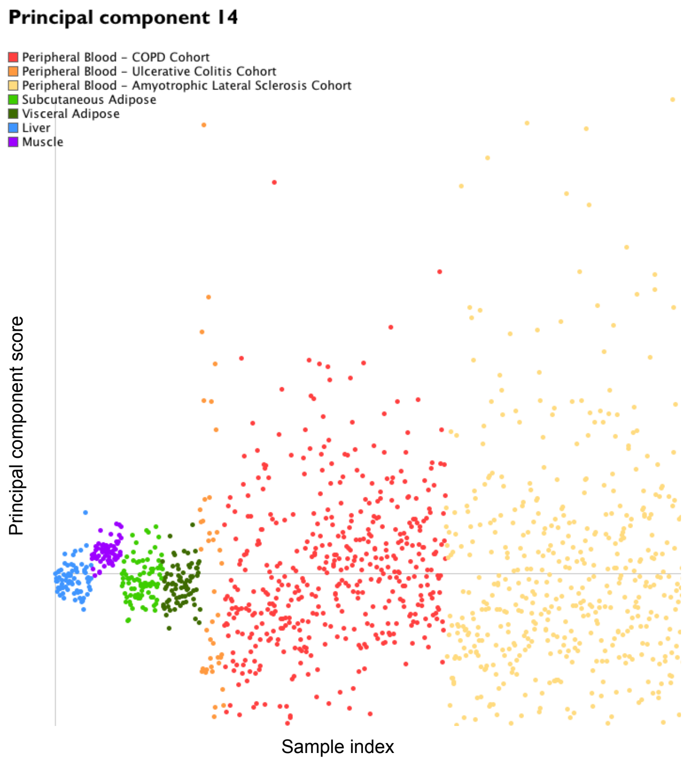
Principal component 14
Peripheral Blood - COPD Cohort
Peripheral Blood - Ulcerative Colitis Cohort
Peripheral Blood - Amyotrophic Lateral Sclerosis Cohort
Subcutaneous Adipose
Visceral Adipose
Liver
Muscle
Principal component score
Sample index

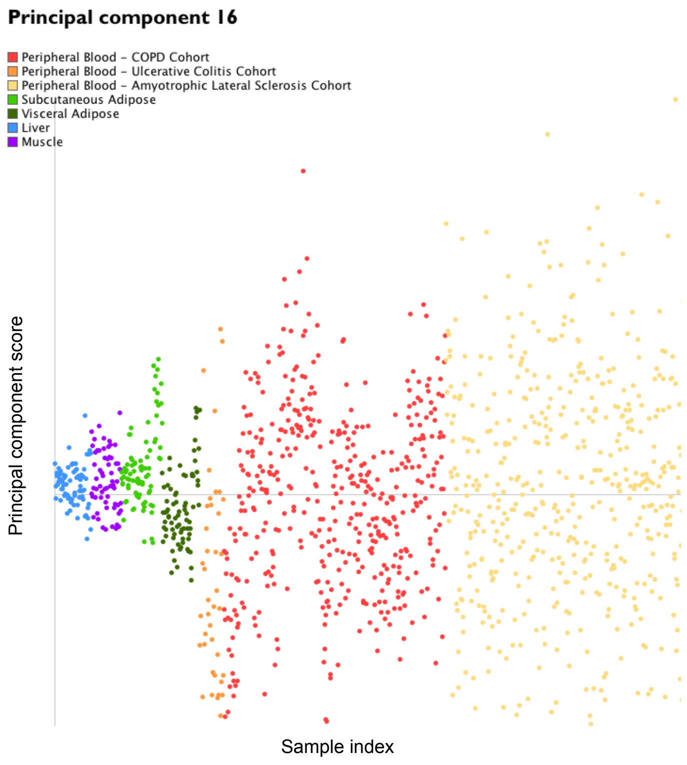
Principal component 16
Peripheral Blood - COPD Cohort
Peripheral Blood - Ulcerative Colitis Cohort
Peripheral Blood - Amyotrophic Lateral Sclerosis Cohort
Subcutaneous Adipose
Visceral Adipose
Liver
Muscle
Principal component score
Sample index

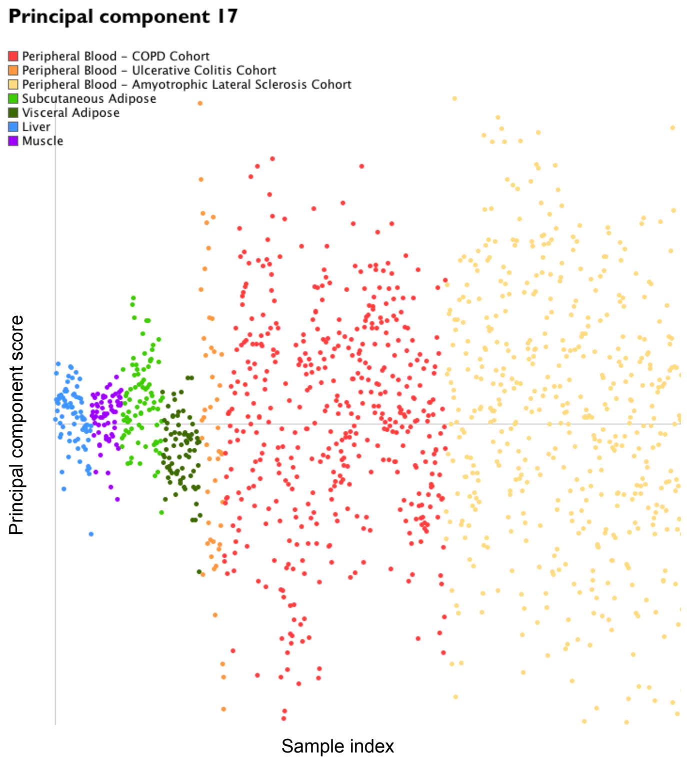
Principal component 17
Peripheral Blood - COPD Cohort
Peripheral Blood - Ulcerative Colitis Cohort
Peripheral Blood - Amyotrophic Lateral Sclerosis Cohort
Subcutaneous Adipose
Visceral Adipose
Liver
Muscle
Principal component score
Sample index

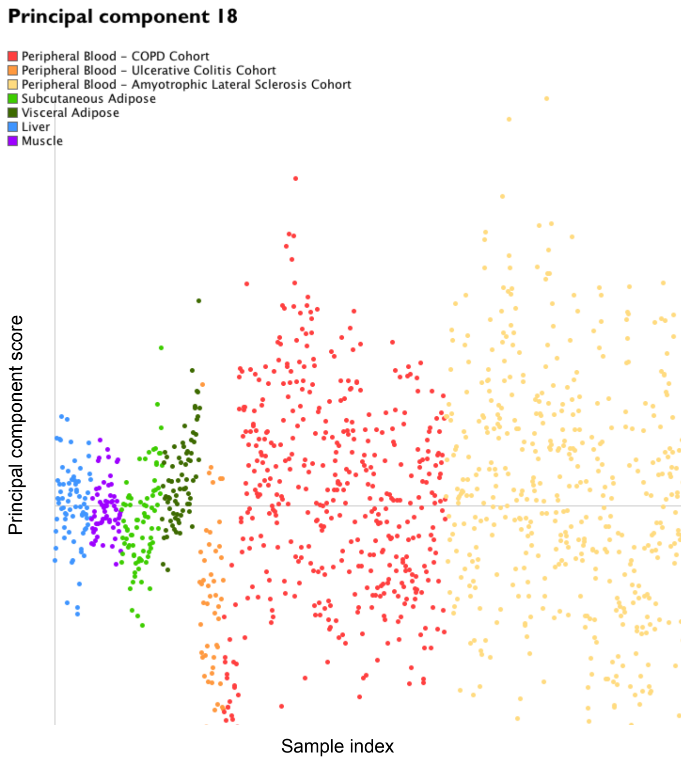
Principal component 18
Peripheral Blood - COPD Cohort
Peripheral Blood - Ulcerative Colitis Cohort
Peripheral Blood - Amyotrophic Lateral Sclerosis Cohort
Subcutaneous Adipose
Visceral Adipose
Liver
Muscle
Principal component score
Sample index

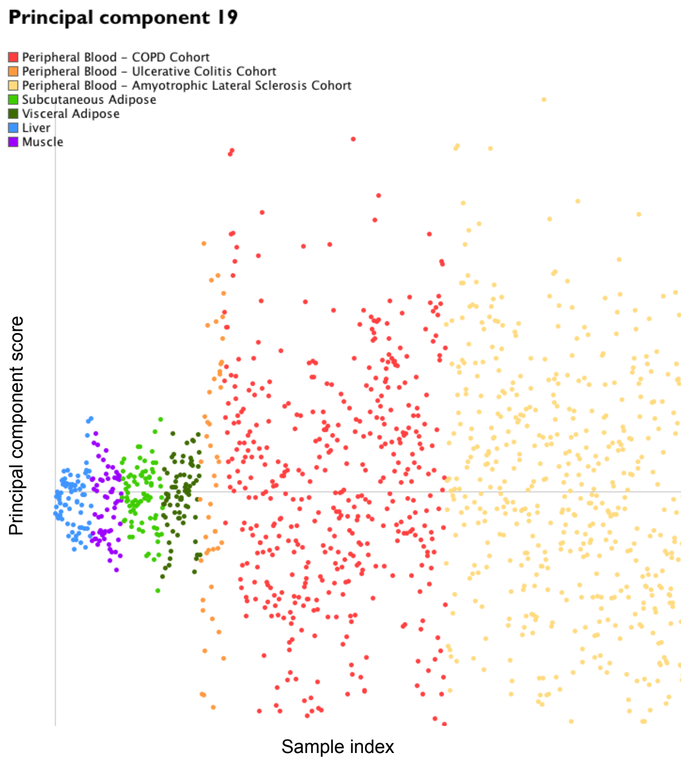
Principal component 19
Peripheral Blood - COPD Cohort
Peripheral Blood - Ulcerative Colitis Cohort
Peripheral Blood - Amyotrophic Lateral Sclerosis Cohort
Subcutaneous Adipose
Visceral Adipose
Liver
Muscle
Principal component score
Sample index

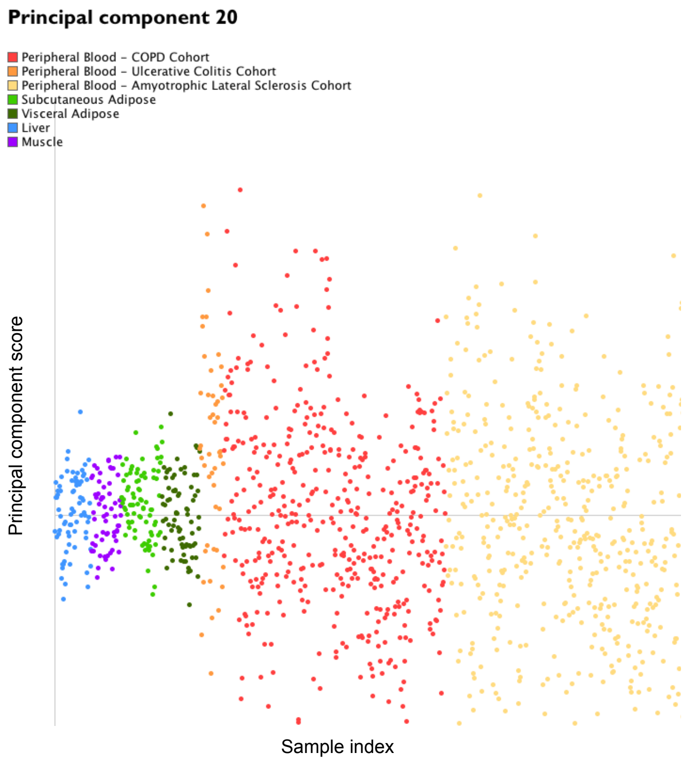
Principal component 20
Peripheral Blood - COPD Cohort
Peripheral Blood - Ulcerative Colitis Cohort
Peripheral Blood - Amyotrophic Lateral Sclerosis Cohort
Subcutaneous Adipose
Visceral Adipose
Liver
Muscle
Principal component score
Sample index

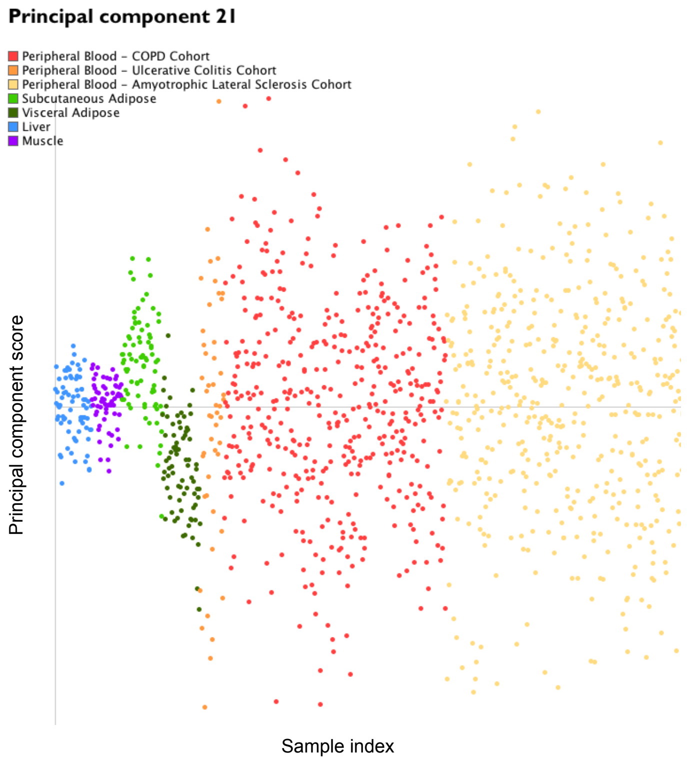
Principal component 21
Peripheral Blood – COPD Cohort
Peripheral Blood – Ulcerative Colitis Cohort
Peripheral Blood – Amyotrophic Lateral Sclerosis Cohort
Subcutaneous Adipose
Visceral Adipose
Liver
Muscle
Principal component score
Sample index

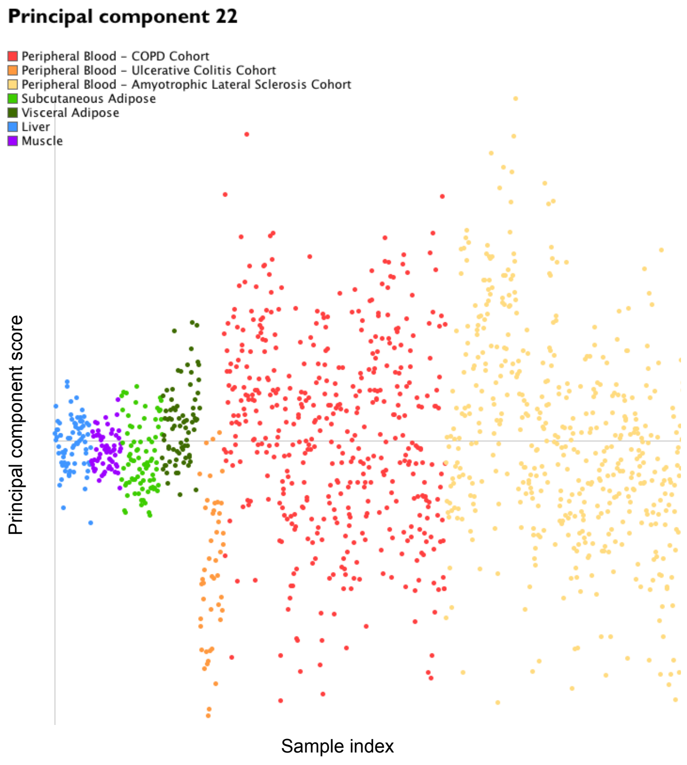
Principal component 22
Peripheral Blood – COPD Cohort
Peripheral Blood – Ulcerative Colitis Cohort
Peripheral Blood – Amyotrophic Lateral Sclerosis Cohort
Subcutaneous Adipose
Visceral Adipose
Liver
Muscle
Principal component score
Sample index

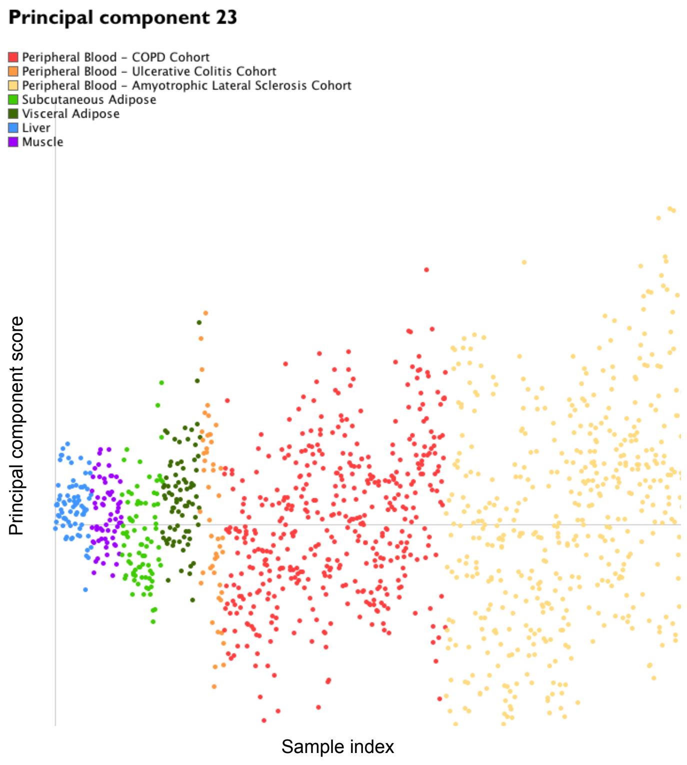
Principal component 23
Peripheral Blood – COPD Cohort
Peripheral Blood – Ulcerative Colitis Cohort
Peripheral Blood – Amyotrophic Lateral Sclerosis Cohort
Subcutaneous Adipose
Visceral Adipose
Liver
Muscle
Principal component score
Sample index

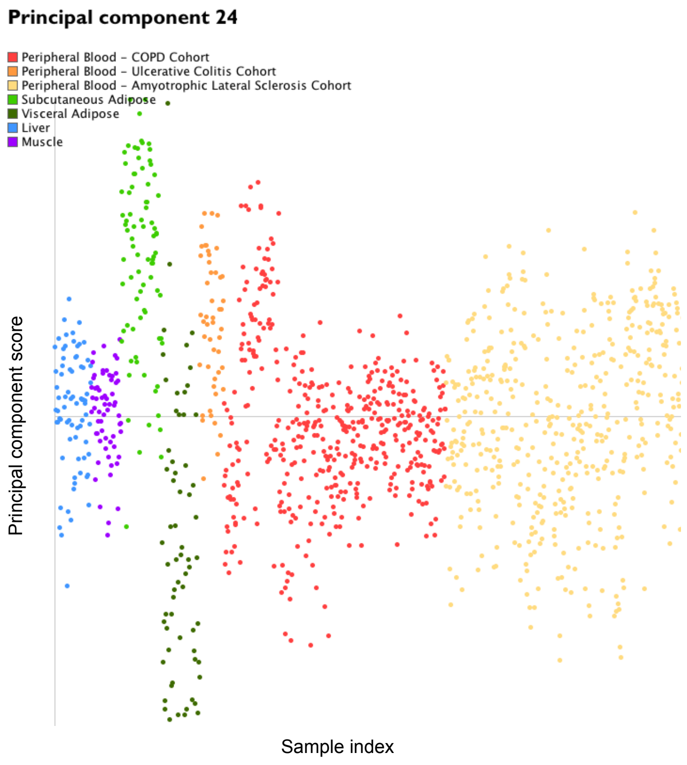
Principal component 24
Peripheral Blood – COPD Cohort
Peripheral Blood – Ulcerative Colitis Cohort
Peripheral Blood – Amyotrophic Lateral Sclerosis Cohort
Subcutaneous Adipose
Visceral Adipose
Liver
Muscle
Principal component score
Sample index

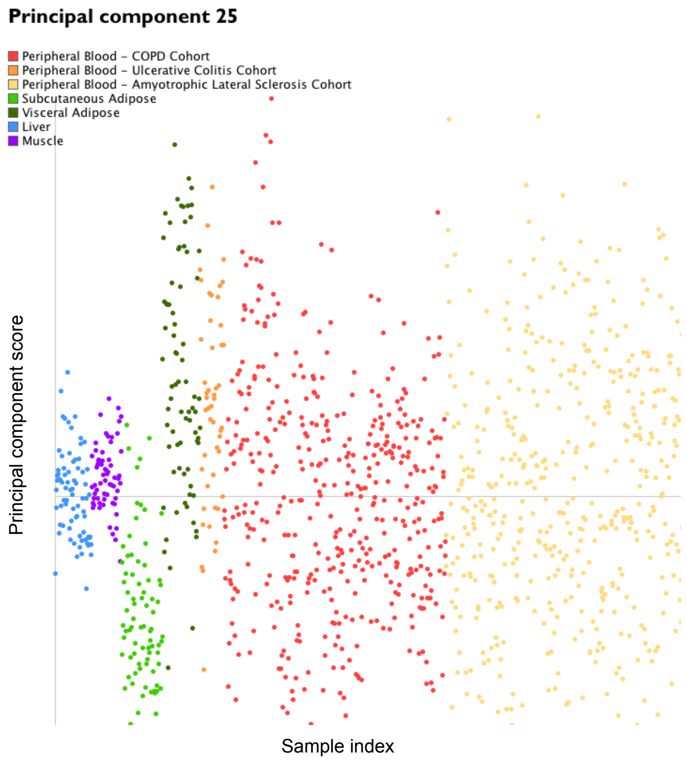
Principal component 25
Peripheral Blood – COPD Cohort
Peripheral Blood – Ulcerative Colitis Cohort
Peripheral Blood – Amyotrophic Lateral Sclerosis Cohort
Subcutaneous Adipose
Visceral Adipose
Liver
Muscle
Principal component score
Sample index

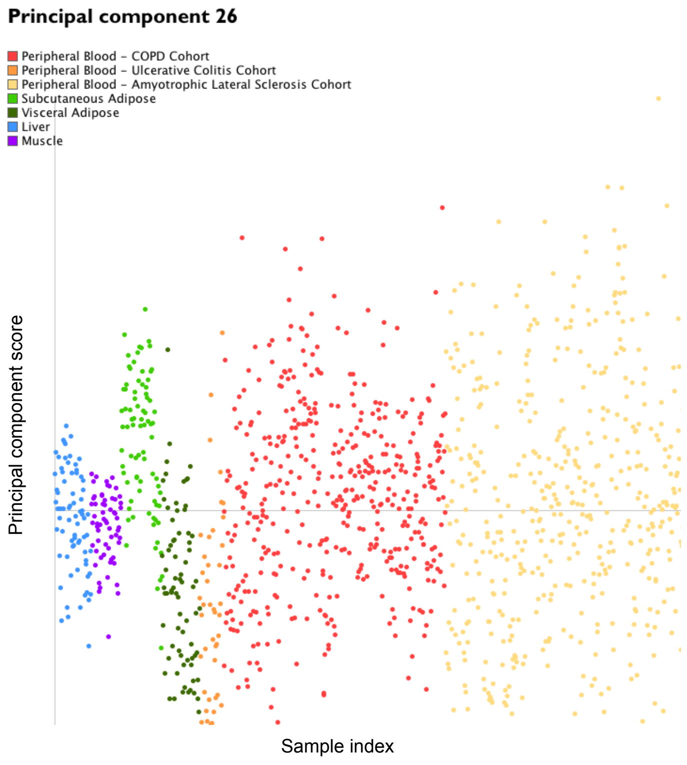
Principal component 26
Peripheral Blood – COPD Cohort
Peripheral Blood – Ulcerative Colitis Cohort
Peripheral Blood – Amyotrophic Lateral Sclerosis Cohort
Subcutaneous Adipose
Visceral Adipose
Liver
Muscle
Principal component score
Sample index

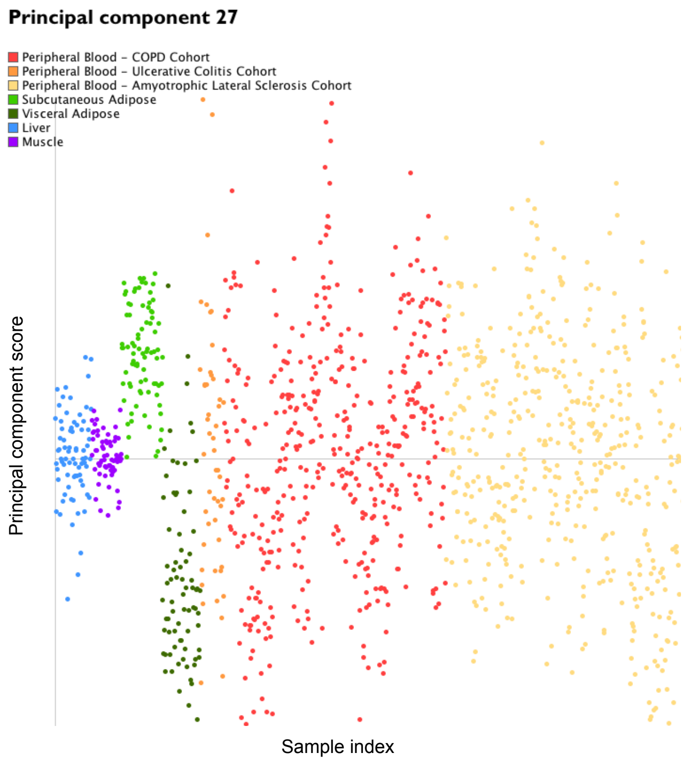
Principal component 27
Peripheral Blood – COPD Cohort
Peripheral Blood – Ulcerative Colitis Cohort
Peripheral Blood – Amyotrophic Lateral Sclerosis Cohort
Subcutaneous Adipose
Visceral Adipose
Liver
Muscle
Principal component score
Sample index

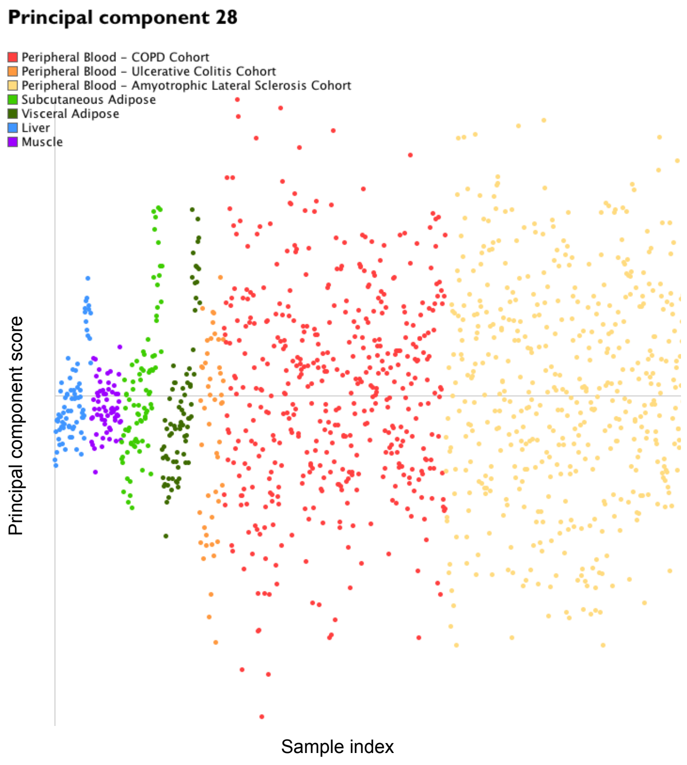
Principal component 28
Peripheral Blood – COPD Cohort
Peripheral Blood – Ulcerative Colitis Cohort
Peripheral Blood – Amyotrophic Lateral Sclerosis Cohort
Subcutaneous Adipose
Visceral Adipose
Liver
Muscle
Principal component score
Sample index

Principal component 29

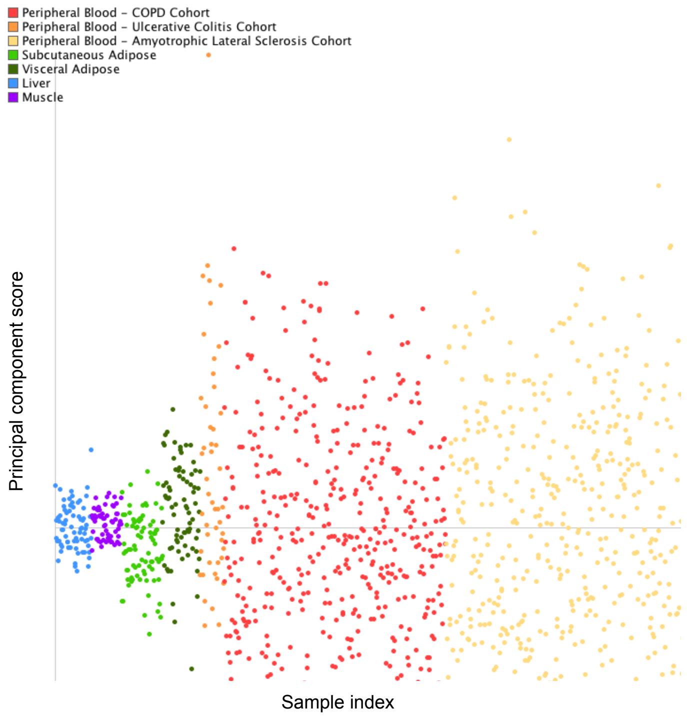

Principal component 30

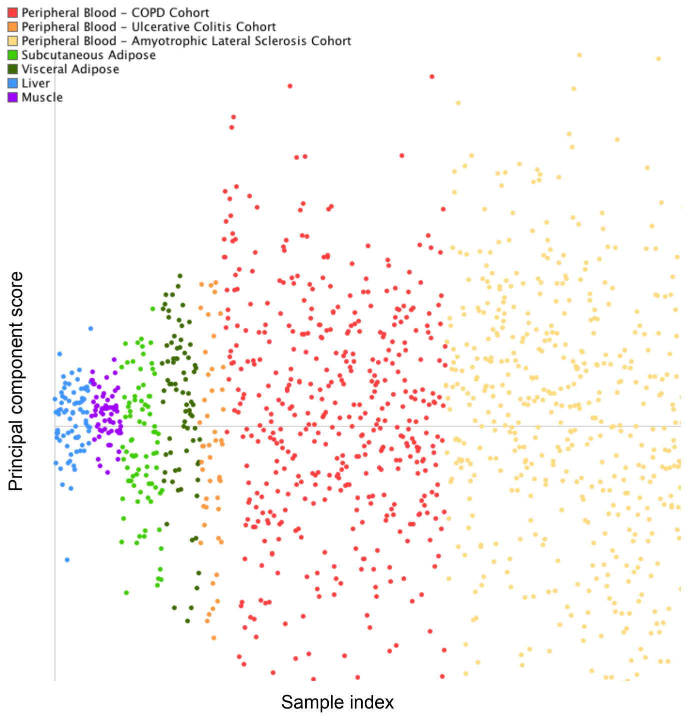

Principal component 31

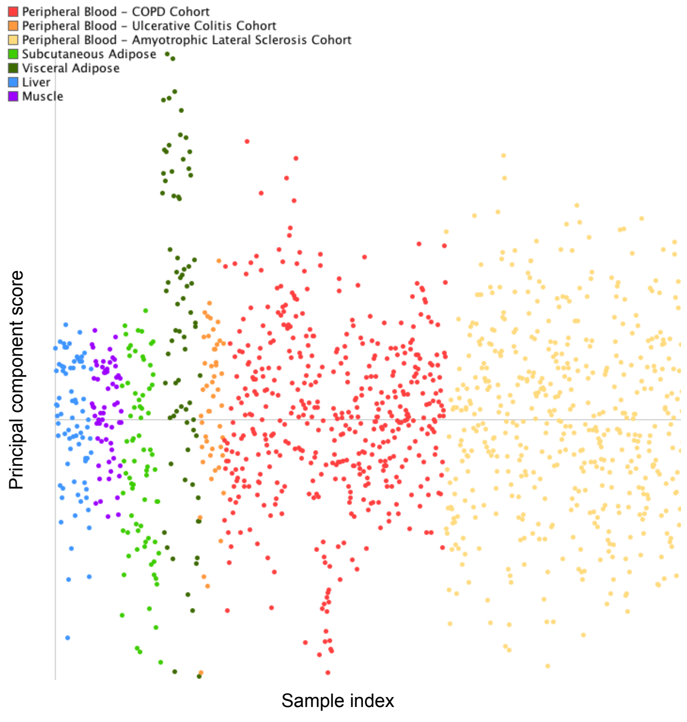

Principal component 32

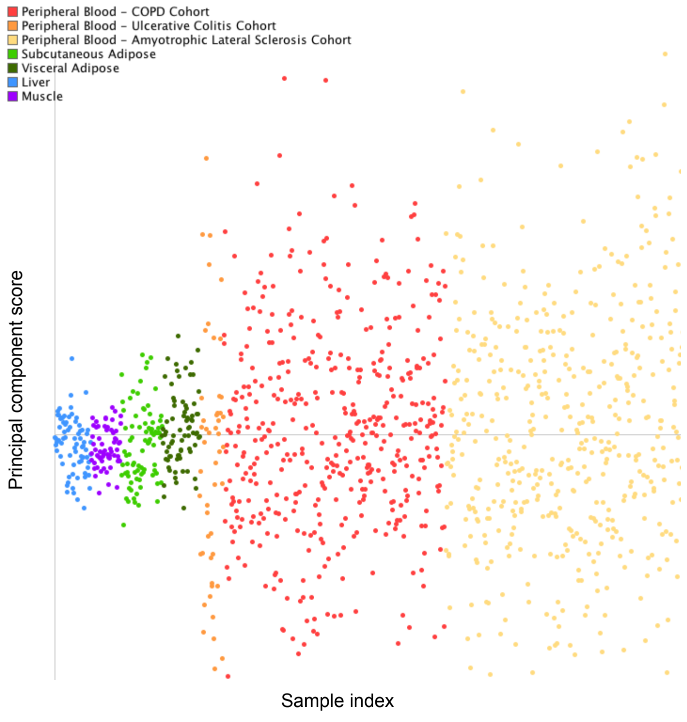

Principal component 33

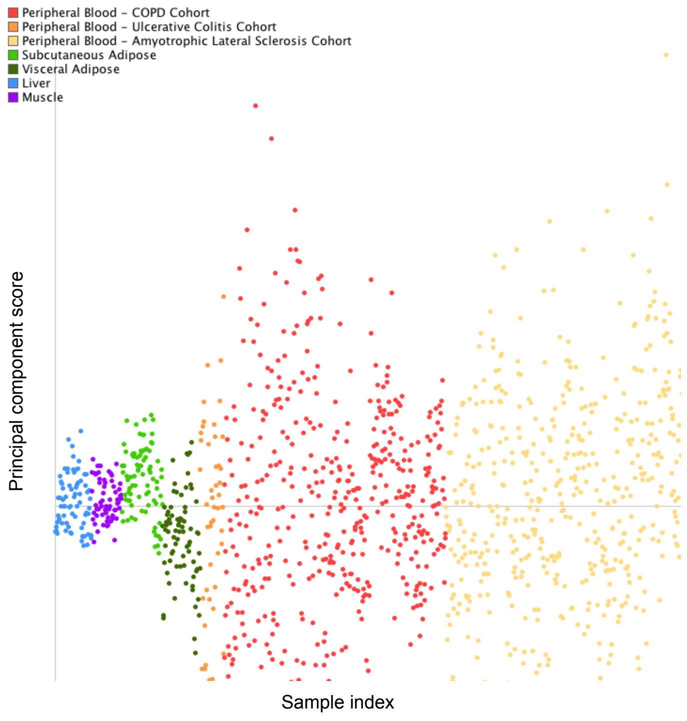

Principal component 34

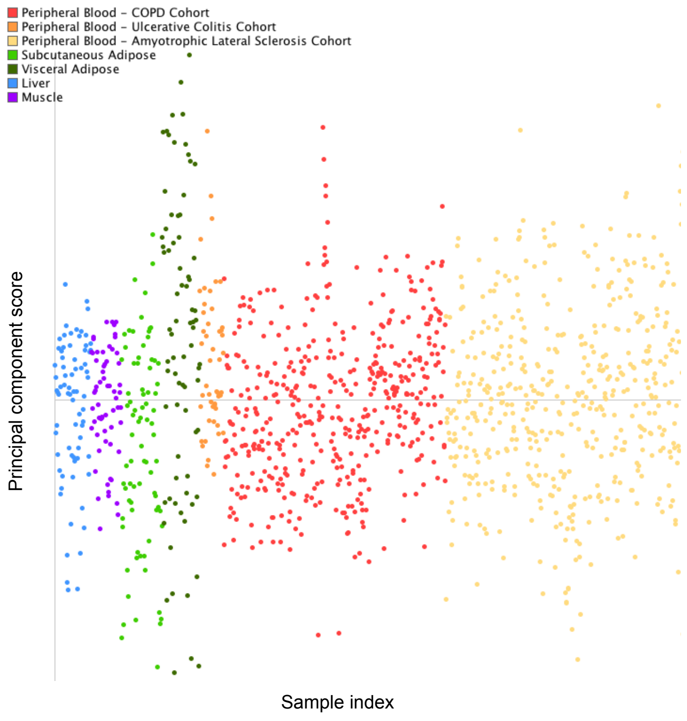

Principal component 35

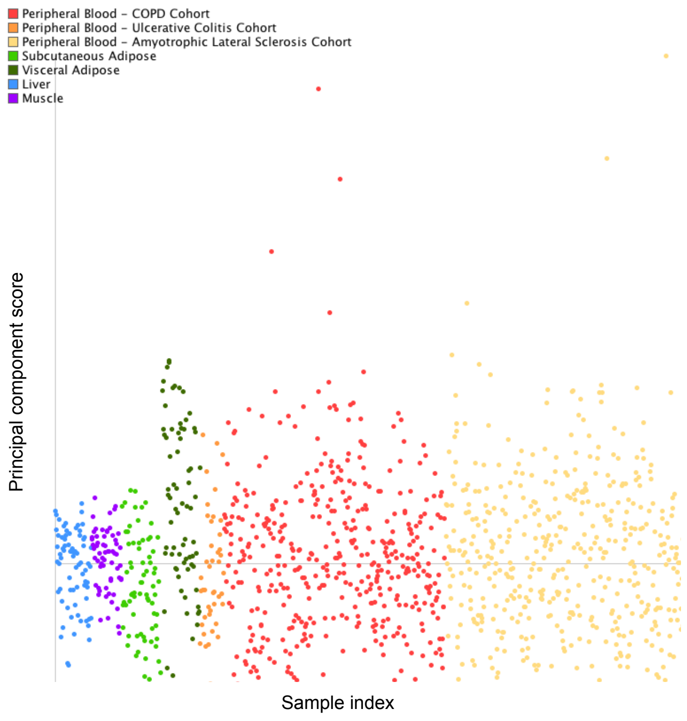

Principal component 36

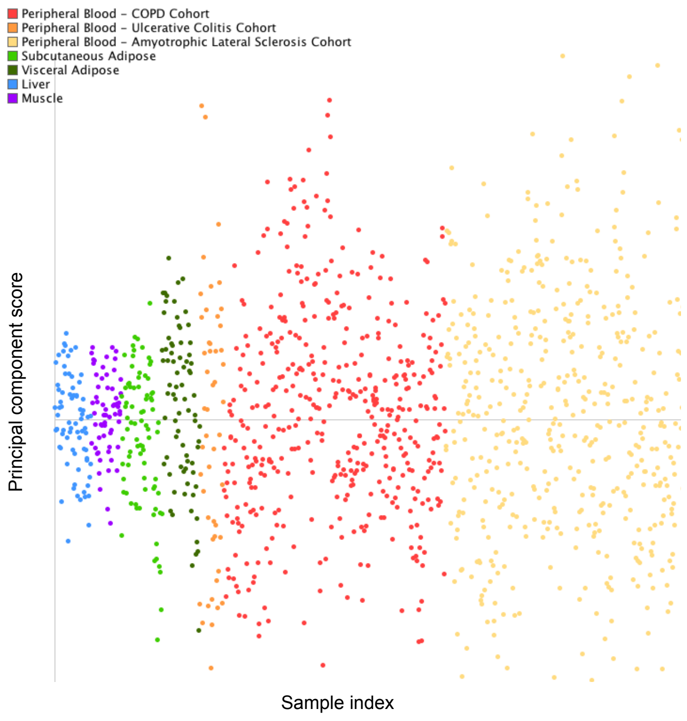

Principal component 37

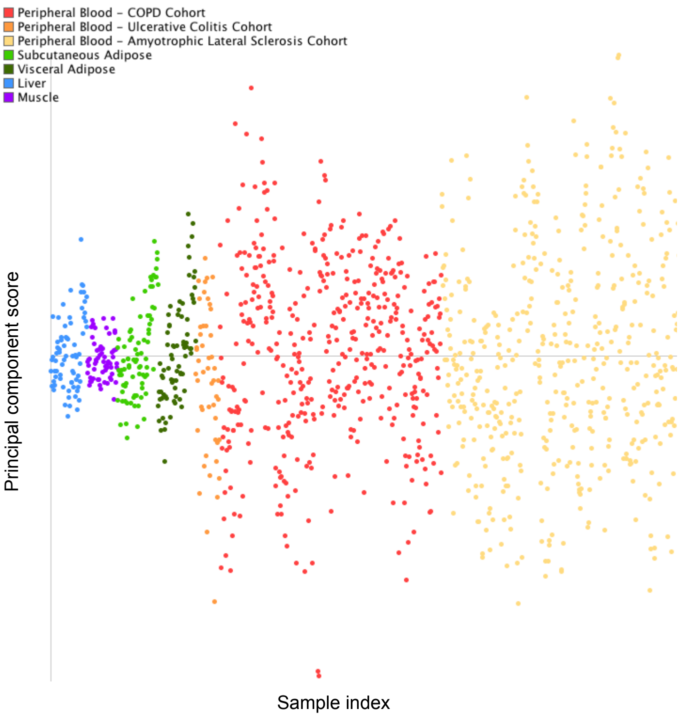

Principal component 38

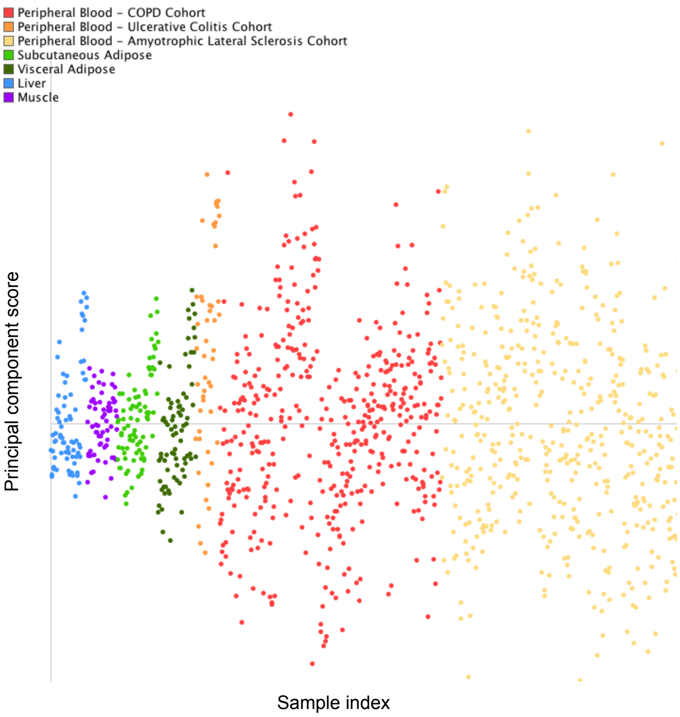

Principal component 39

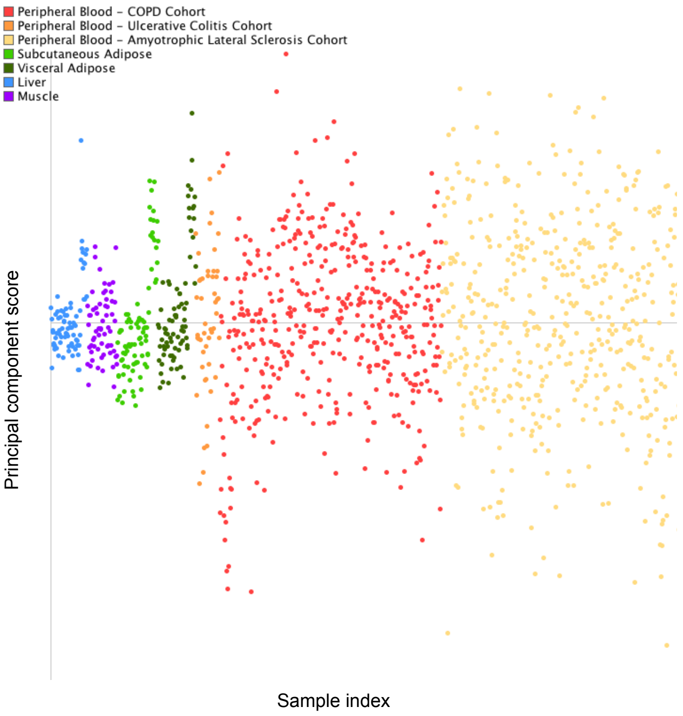

Principal component 40

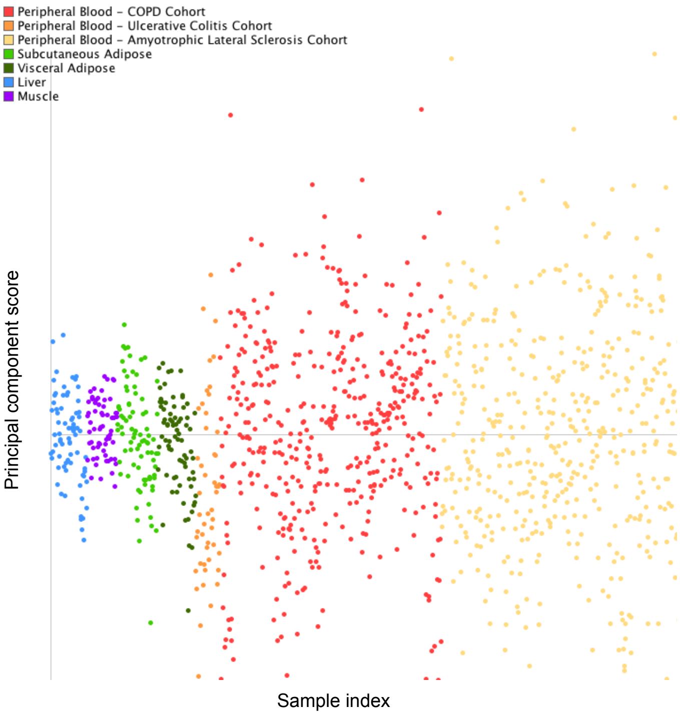

Principal component 41

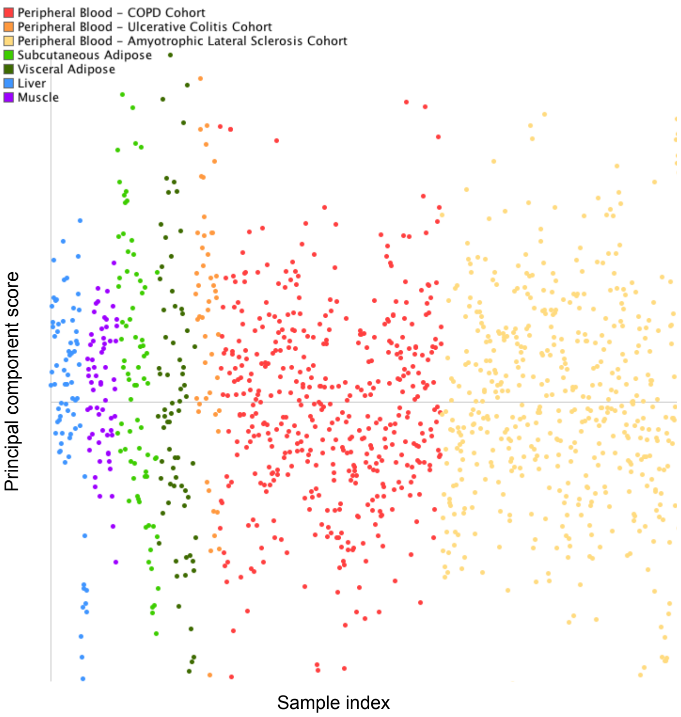

Principal component 42

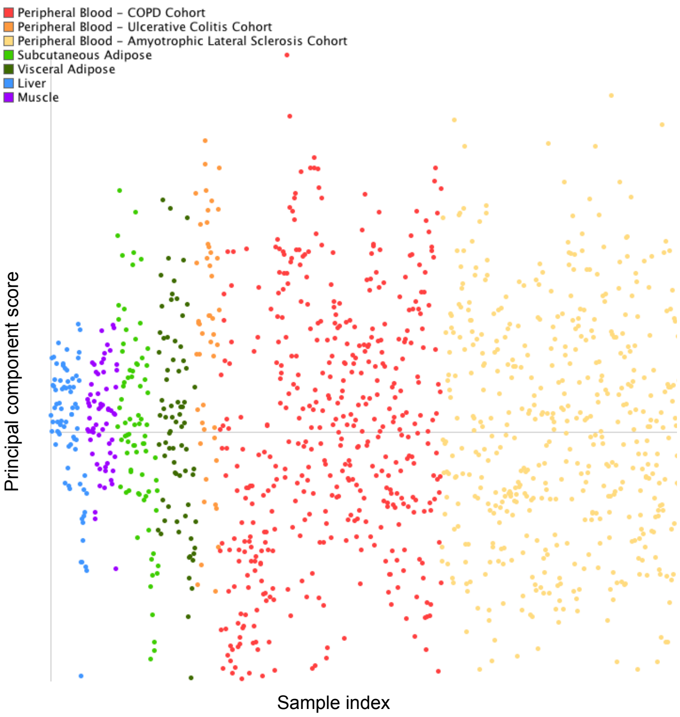

Principal component 43

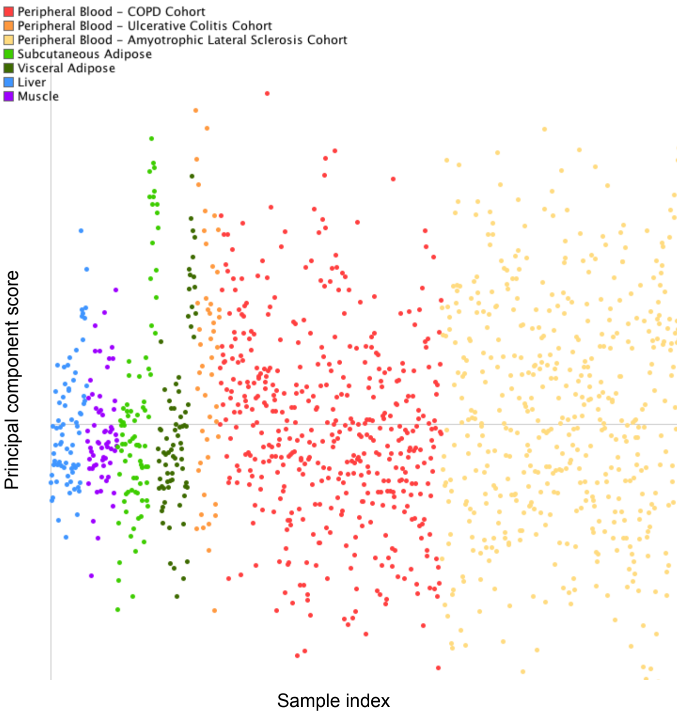

Principal component 44

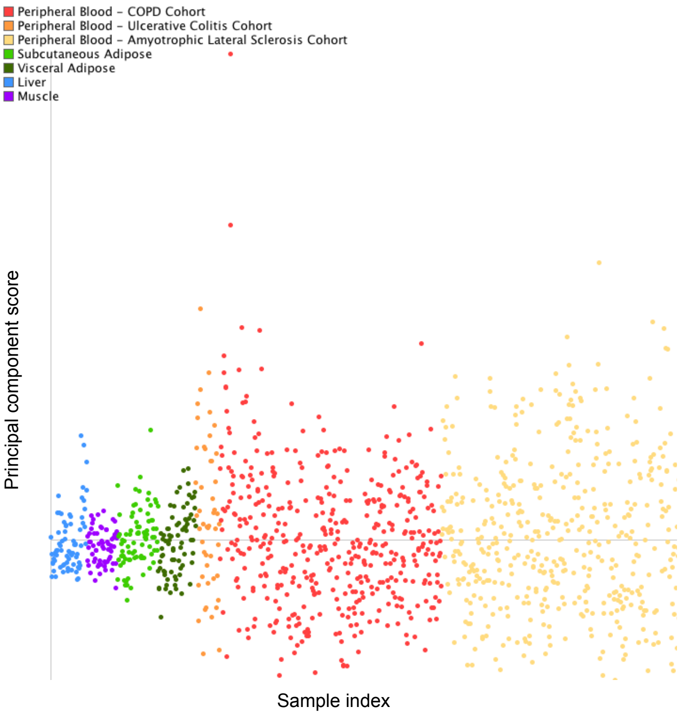

Principal component 45

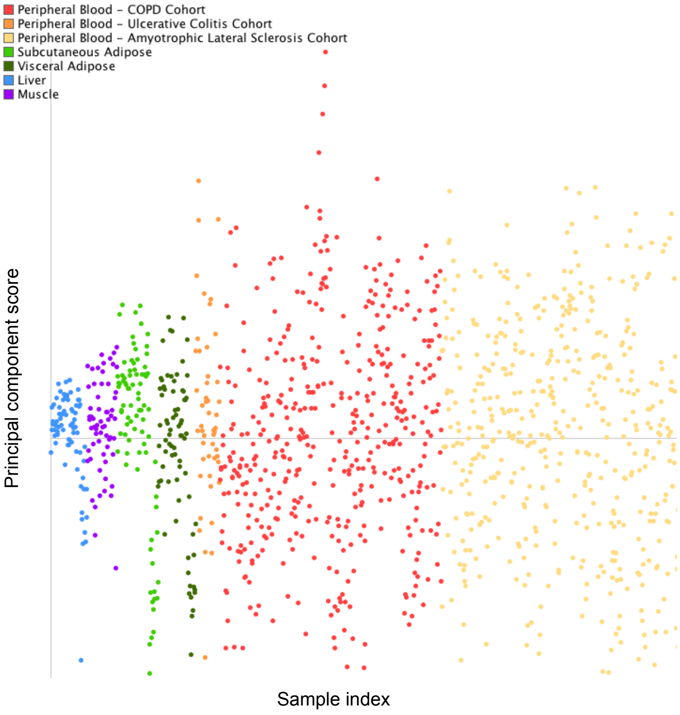

Principal component 46

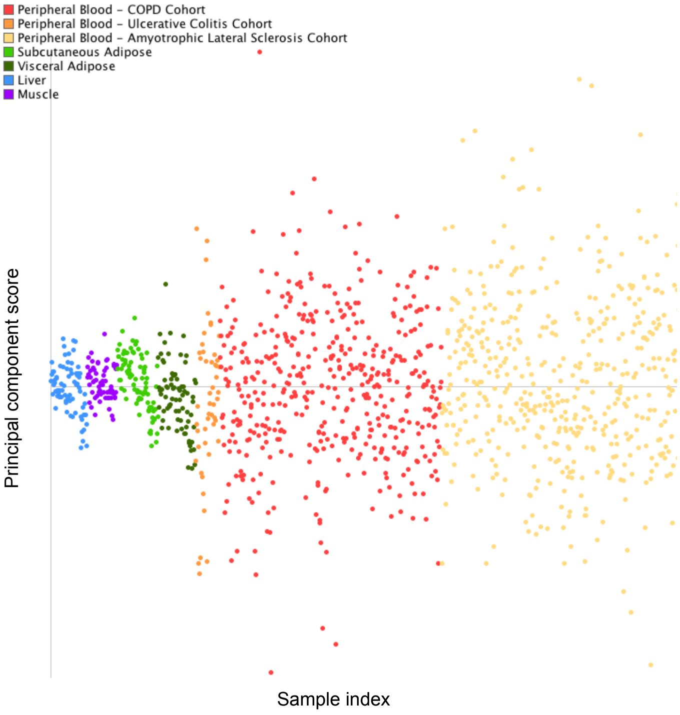

Principal component 47

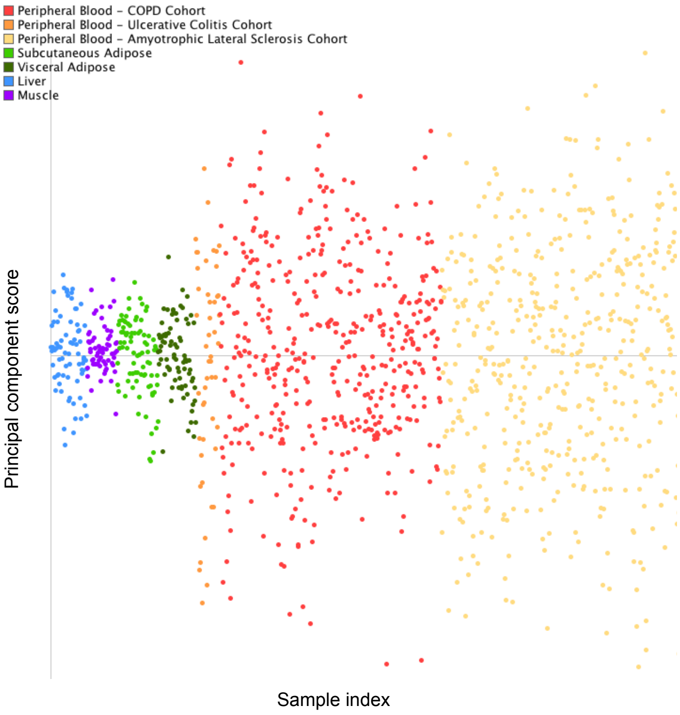

Principal component 48

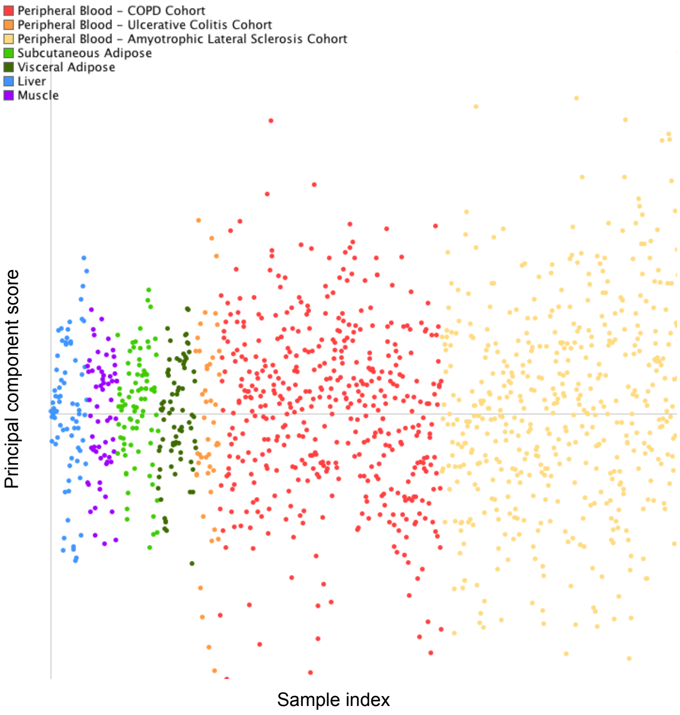

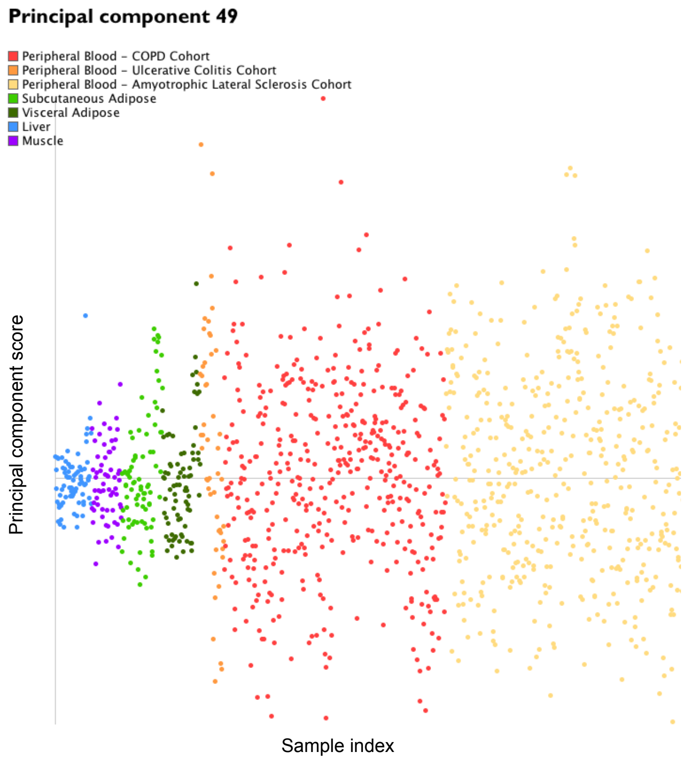

Principal component 49
Peripheral Blood - COPD Cohort
Peripheral Blood - Ulcerative Colitis Cohort
Peripheral Blood - Amyotrophic Lateral Sclerosis Cohort
Subcutaneous Adipose
Visceral Adipose
Liver
Muscle
Principal component score
Sample index

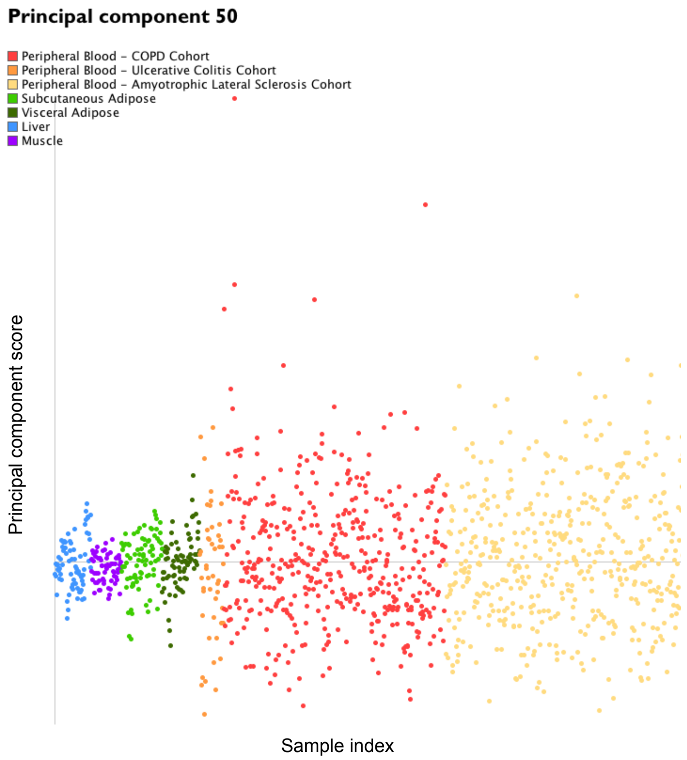

Principal component 50
Peripheral Blood - COPD Cohort
Peripheral Blood - Ulcerative Colitis Cohort
Peripheral Blood - Amyotrophic Lateral Sclerosis Cohort
Subcutaneous Adipose
Visceral Adipose
Liver
Muscle
Principal component score
Sample index
