# Supplementary material for: Trans-eQTLs Reveal That Independent Genetic Variants Associated with a Complex Phenotype Converge on Intermediate Genes, with a Major Role for the HLA
Source: PLoS Genet. 2011 Aug 4;7(8):e1002197. doi: 10.1371/journal.pgen.1002197 (PMC3150446; doi:10.1371/journal.pgen.1002197)

SNP rs4948088 (chr. 7, 50994688 bp)
Probe 3870619 (chr. 6, 111301680 - 111324609 bp), AMD1
P-Value 7.98E-6

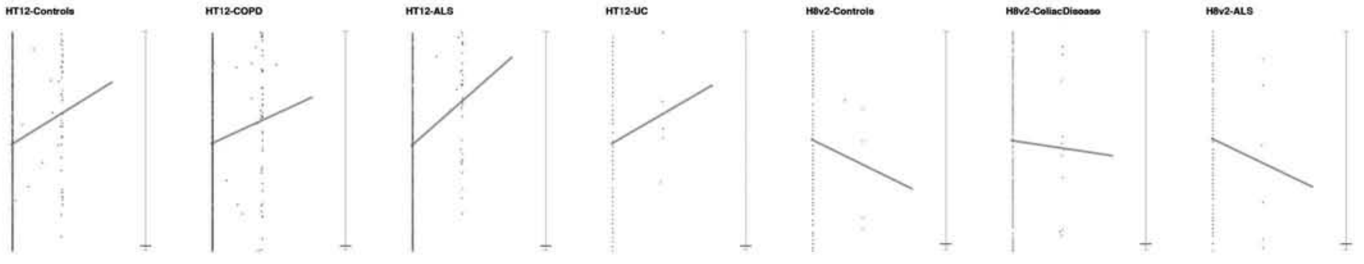

	HT12-Controls	HT12-COPD	HT12-ALS	HT12-UC	H8v2-Controls	H8v2-CeliacDisease	H8v2-ALS
Genotypes	CC (369) CA (45) AA (0)	CC (405) CA (48) AA (0)	CC (290) CA (34) AA (0)	CC (44) CA (5) AA (0)	CC (54) CA (5) AA (0)	CC (100) CA (11) AA (0)	CC (53) CA (6) AA (0)
Corr:	0.15	0.11	0.211	0.136	-0.101	-0.036	-0.113
R2:	0.022	0.012	0.044	0.019	0.01	0.001	0.013
Z-Score:	3.055	2.342	3.823	0.933	-0.759	-0.371	-0.851
P-Value:	0.002	0.019	1.32E-4	0.351	0.448	0.711	0.395

SNP rs2517713 (chr. 6, 30026078 bp)
Probe 6840408 (chr. 6, 6532340 - 6601215 bp), LY86
P-Value 5.06E-7

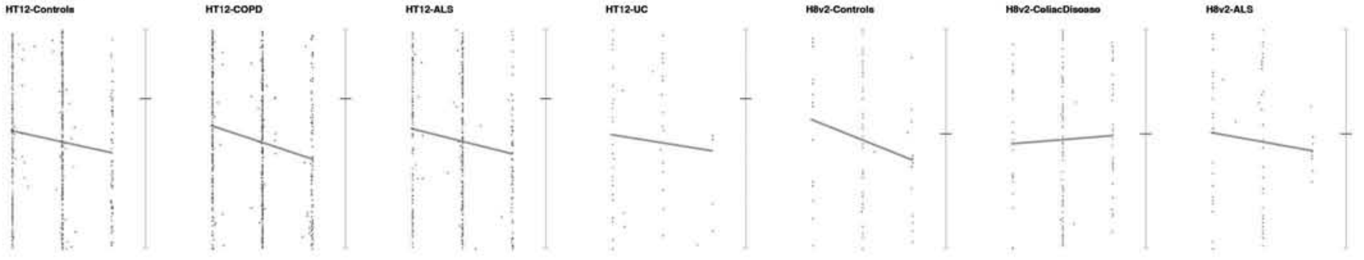

	HT12-Controls	HT12-COPD	HT12-ALS	HT12-UC	H8v2-Controls	H8v2-CeliacDisease	H8v2-ALS
Genotypes	TT (150) TG (205) GG (59)	TT (162) TG (209) GG (82)	TT (98) TG (170) GG (56)	TT (27) TG (17) GG (5)	TT (14) TG (32) GG (13)	TT (20) TG (62) GG (29)	TT (23) TG (28) GG (8)
Corr:	-0.118	-0.183	-0.134	-0.08	-0.213	0.043	-0.092
R2:	0.014	0.034	0.018	0.006	0.045	0.002	0.009
Z-Score:	-2.404	-3.921	-2.405	-0.544	-1.617	0.444	-0.696
P-Value:	0.016	8.82E-5	0.016	0.587	0.106	0.657	0.486

SNP rs3803662 (chr. 16, 51143842 bp)
Probe 4830343 (chr. 16, 45280059 - 45290806 bp), ORC6L
P-Value 4.12E-7

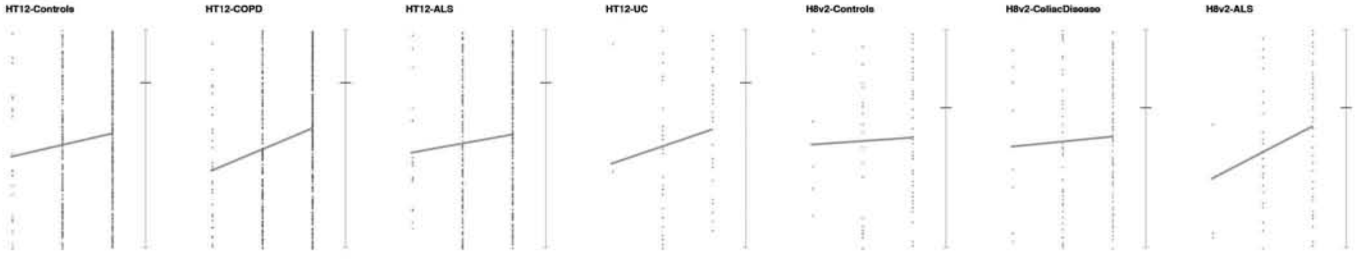

	HT12-Controls	HT12-COPD	HT12-ALS	HT12-UC	H8v2-Controls	H8v2-CeliacDisease	H8v2-ALS
Genotypes	AA (20) AG (157) GG (237)	AA (26) AG (176) GG (249)	AA (17) AG (130) GG (177)	AA (2) AG (23) GG (24)	AA (6) AG (18) GG (35)	AA (8) AG (37) GG (66)	AA (3) AG (22) GG (34)
Corr:	0.106	0.208	0.082	0.148	0.038	0.05	0.237
R2:	0.011	0.043	0.007	0.022	0.001	0.002	0.056
Z-Score:	2.158	4.453	1.478	1.018	0.289	0.518	1.806
P-Value:	0.031	8.48E-6	0.139	0.309	0.773	0.604	0.071

SNP rs2292239 (chr. 12, 54768447 bp)
Probe 3190671 (chr. 19, 1191749 - 1196823 bp), ATP5D
P-Value 4.11E-7

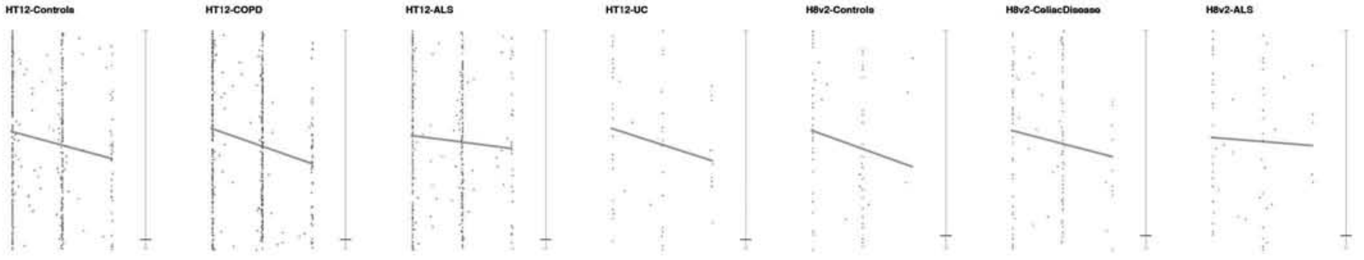

	HT12-Controls	HT12-COPD	HT12-ALS	HT12-UC	H8v2-Controls	H8v2-CeliacDisease	H8v2-ALS
Genotypes	GG (196) GT (180) TT (38)	GG (198) GT (206) TT (49)	GG (144) GT (147) TT (33)	GG (23) GT (17) TT (9)	GG (30) GT (26) TT (3)	GG (44) GT (55) TT (12)	GG (28) GT (26) TT (5)
Corr:	-0.134	-0.181	-0.066	-0.182	-0.162	-0.129	-0.041
R2:	0.018	0.033	0.004	0.033	0.026	0.017	0.002
Z-Score:	-2.726	-3.885	-1.178	-1.254	-1.227	-1.352	-0.31
P-Value:	0.006	1.03E-4	0.239	0.21	0.22	0.176	0.756

SNP rs9271366 (chr. 6, 32694832 bp)
Probe 2640369 (chr. 6, 159105766 - 159161432 bp), VIL2
P-Value 4.06E-7

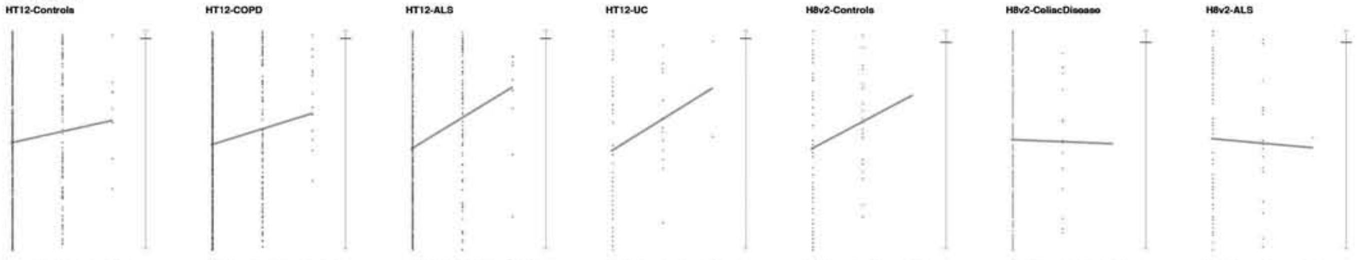

	HT12-Controls	HT12-COPD	HT12-ALS	HT12-UC	H8v2-Controls	H8v2-CeliacDisease	H8v2-ALS
Genotypes	AA (316) AG (90) GG (8)	AA (342) AG (98) GG (13)	AA (239) AG (75) GG (10)	AA (34) AG (13) GG (2)	AA (40) AG (19) GG (0)	AA (97) AG (14) GG (0)	AA (42) AG (16) GG (1)
Corr:	0.085	0.122	0.251	0.27	0.192	-0.01	-0.032
R2:	0.007	0.015	0.063	0.073	0.037	0	0.001
Z-Score:	1.726	2.6	4.582	1.879	1.455	-0.106	-0.241
P-Value:	0.084	0.009	4.6E-6	0.06	0.146	0.916	0.809

SNP rs12485738 (chr. 3, 56840816 bp)
Probe 5870138 (chr. 12, 5927301 - 6105097 bp), VWF
P-Value 3.86E-7

HT12-Controls	HT12-COPD	HT12-ALS	HT12-UC	H8v2-Controls	H8v2-CeliacDisease	H8v2-ALS

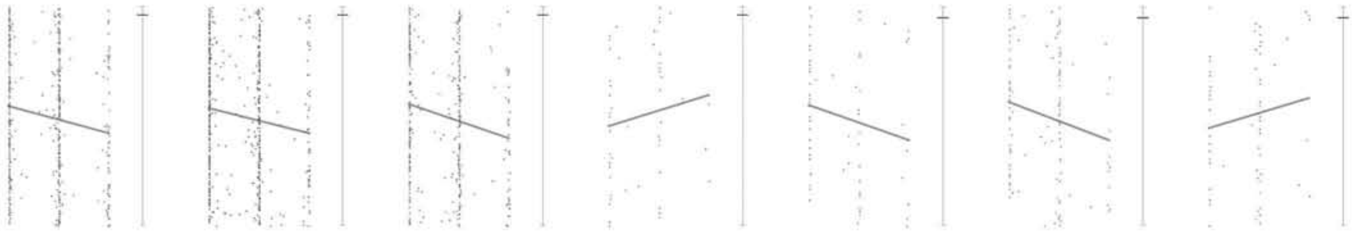

HT12-Controls	HT12-COPD	HT12-ALS	HT12-UC	H8v2-Controls	H8v2-CeliacDisease	H8v2-ALS
GG (153) GA (194) AA (67)	GG (202) GA (200) AA (51)	GG (132) GA (148) AA (44)	GG (24) GA (22) AA (3)	GG (28) GA (21) AA (10)	GG (41) GA (57) AA (13)	GG (20) GA (32) AA (7)
Corr: -0.146 R2: 0.021	Corr: -0.13 R2: 0.017	Corr: -0.178 R2: 0.032	Corr: 0.142 R2: 0.02	Corr: -0.195 R2: 0.038	Corr: -0.188 R2: 0.035	Corr: 0.143 R2: 0.02
Z-Score:-2.975 P-Value:0.003	Z-Score:-2.765 P-Value:0.006	Z-Score:-3.222 P-Value:0.001	Z-Score:0.971 P-Value:0.331	Z-Score:-1.478 P-Value:0.139	Z-Score:-1.972 P-Value:0.049	Z-Score:1.077 P-Value:0.281

SNP rs12485738 (chr. 3, 56840816 bp)
Probe 1070450 (chr. 9, 124172050 - 124198802 bp), PTGS1
P-Value 3.29E-7

HT12-Controls	HT12-COPD	HT12-ALS	HT12-UC	H8v2-Controls	H8v2-CeliacDisease	H8v2-ALS
				**eQTL not available**	**eQTL not available**	**eQTL not available**
				Probe not present.	Probe not present.	Probe not present.

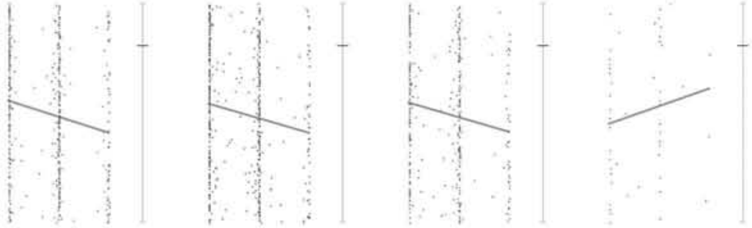

HT12-Controls	HT12-COPD	HT12-ALS	HT12-UC
GG (153) GA (194) AA (67)	GG (202) GA (200) AA (51)	GG (132) GA (148) AA (44)	GG (24) GA (22) AA (3)
Corr: -0.169 R2: 0.029	Corr: -0.152 R2: 0.023	Corr: -0.149 R2: 0.022	Corr: 0.164 R2: 0.027
Z-Score:-3.457 P-Value:5.46E-4	Z-Score:-3.246 P-Value:0.001	Z-Score:-2.678 P-Value:0.007	Z-Score:1.125 P-Value:0.261

SNP rs11887534 (chr. 2, 43919751 bp)
Probe 3120368 (chr. 3, 9910358 - 9934079 bp), IL17RE
P-Value 2.79E-7

HT12-Controls	HT12-COPD	HT12-ALS	HT12-UC	H8v2-Controls	H8v2-CeliacDisease	H8v2-ALS
				**eQTL not available**	**eQTL not available**	**eQTL not available**
				Probe not present.	Probe not present.	Probe not present.

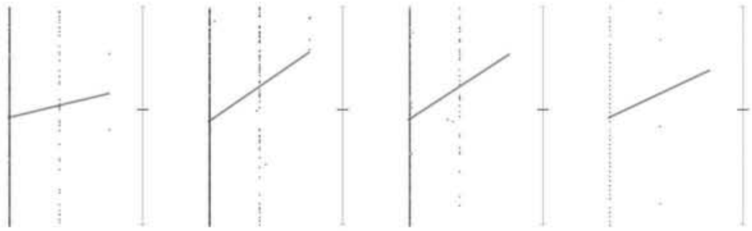

HT12-Controls	HT12-COPD	HT12-ALS	HT12-UC
GG (373) GC (39) CC (2)	GG (381) GC (68) CC (4)	GG (289) GC (35) CC (0)	GG (45) GC (4) CC (0)
Corr: 0.06 R2: 0.004	Corr: 0.218 R2: 0.047	Corr: 0.159 R2: 0.025	Corr: 0.1 R2: 0.01
Z-Score:1.214 P-Value:0.225	Z-Score:4.674 P-Value:2.95E-6	Z-Score:2.874 P-Value:0.004	Z-Score:0.685 P-Value:0.494

SNP rs2523393 (chr. 6, 29813638 bp)
Probe 2000128 (chr. 1, 205343230 - 205385929 bp), C4BPA
P-Value 2.44E-7

HT12-Controls	HT12-COPD	HT12-ALS	HT12-UC	H8v2-Controls	H8v2-CeliacDisease	H8v2-ALS

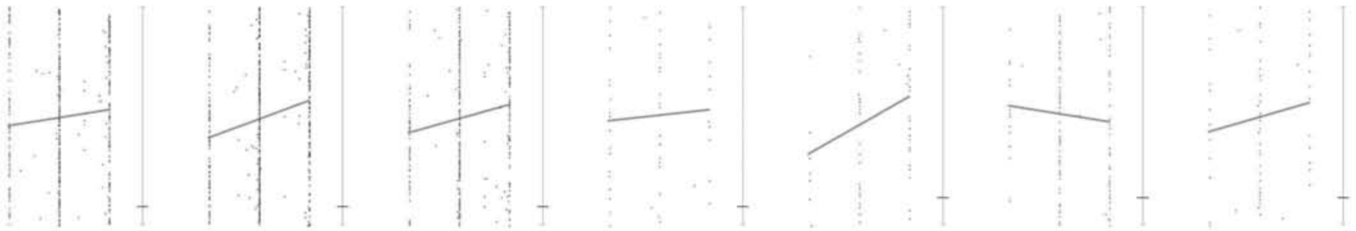

HT12-Controls	HT12-COPD	HT12-ALS	HT12-UC	H8v2-Controls	H8v2-CeliacDisease	H8v2-ALS
GG (72) GA (196) AA (148)	GG (75) GA (221) AA (157)	GG (53) GA (158) AA (113)	GG (18) GA (20) AA (11)	GG (7) GA (25) AA (27)	GG (14) GA (54) AA (43)	GG (12) GA (32) AA (15)
Corr: 0.087 R2: 0.008	Corr: 0.203 R2: 0.041	Corr: 0.15 R2: 0.023	Corr: 0.065 R2: 0.004	Corr: 0.298 R2: 0.089	Corr: -0.083 R2: 0.007	Corr: 0.154 R2: 0.024
Z-Score:1.767 P-Value:0.077	Z-Score:4.361 P-Value:1.29E-5	Z-Score:2.709 P-Value:0.007	Z-Score:0.442 P-Value:0.659	Z-Score:2.292 P-Value:0.022	Z-Score:-0.863 P-Value:0.388	Z-Score:1.162 P-Value:0.245

SNP rs2517713 (chr. 6, 30026078 bp)
Probe 1500148 (chr. 22, 38124692 - 38164078 bp), MAP3K7IP1
P-Value 2.07E-7

HT12-Controls	HT12-COPD	HT12-ALS	HT12-UC	H8v2-Controls	H8v2-CeliacDisease	H8v2-ALS

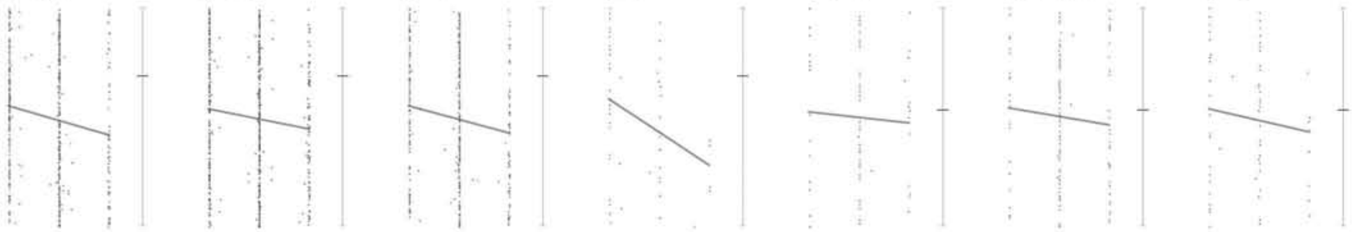

HT12-Controls	HT12-COPD	HT12-ALS	HT12-UC	H8v2-Controls	H8v2-CeliacDisease	H8v2-ALS
TT (150) TG (205) GG (59)	TT (162) TG (209) GG (82)	TT (98) TG (170) GG (56)	TT (27) TG (17) GG (5)	TT (14) TG (32) GG (13)	TT (20) TG (62) GG (29)	TT (23) TG (28) GG (8)
Corr: -0.156 R2: 0.024	Corr: -0.114 R2: 0.013	Corr: -0.143 R2: 0.02	Corr: -0.333 R2: 0.111	Corr: -0.058 R2: 0.003	Corr: -0.087 R2: 0.007	Corr: -0.119 R2: 0.014
Z-Score:-3.187 P-Value:0.001	Z-Score:-2.427 P-Value:0.015	Z-Score:-2.569 P-Value:0.01	Z-Score:-2.341 P-Value:0.019	Z-Score:-0.435 P-Value:0.663	Z-Score:-0.903 P-Value:0.367	Z-Score:-0.897 P-Value:0.37

SNP rs3135363 (chr. 6, 32497626 bp)
Probe 2940424 (chr. 7, 36518135 - 36731595 bp), AOAH
P-Value 2.08E-7

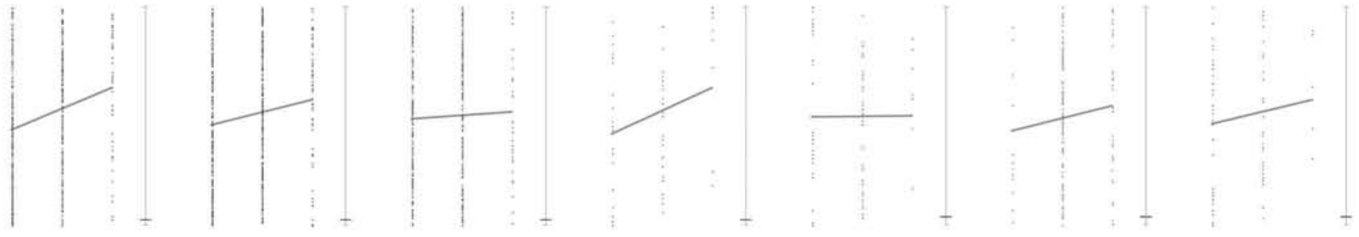

	HT12-Controls	HT12-COPD	HT12-ALS	HT12-UC	H8v2-Controls	H8v2-CeliacDisease	H8v2-ALS
Genotypes	AA (195) AG (173) GG (46)	AA (195) AG (201) GG (57)	AA (145) AG (151) GG (28)	AA (19) AG (22) GG (8)	AA (24) AG (28) GG (7)	AA (12) AG (69) GG (30)	AA (28) AG (26) GG (5)
Corr:	0.223	0.139	0.032	0.253	0.004	0.116	0.122
R2:	0.05	0.019	0.001	0.064	0	0.013	0.015
Z-Score:	4.563	2.957	0.566	1.753	0.034	1.208	0.918
P-Value:	4.58E-6	0.003	0.571	0.08	0.973	0.227	0.359

SNP rs1701704 (chr. 12, 54698754 bp)
Probe 3190671 (chr. 19, 1191749 - 1196823 bp), ATP5D
P-Value 2.05E-7

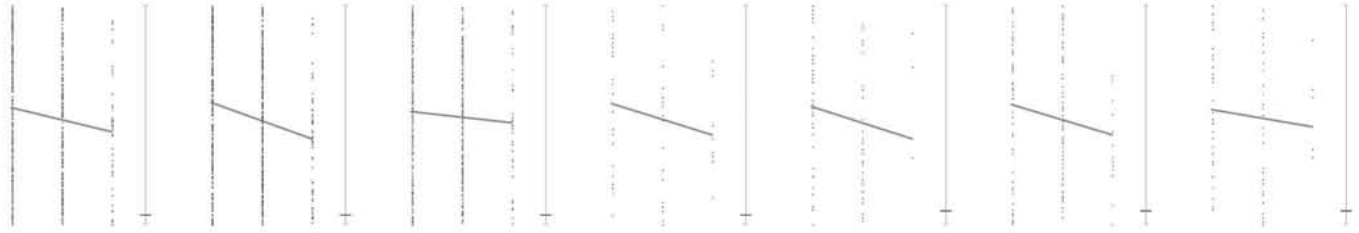

	HT12-Controls	HT12-COPD	HT12-ALS	HT12-UC	H8v2-Controls	H8v2-CeliacDisease	H8v2-ALS
Genotypes	TT (193) TG (178) GG (43)	TT (196) TG (206) GG (51)	TT (145) TG (146) GG (33)	TT (22) TG (18) GG (9)	TT (31) TG (25) GG (3)	TT (44) TG (54) GG (13)	TT (27) TG (27) GG (5)
Corr:	-0.128	-0.191	-0.061	-0.181	-0.146	-0.158	-0.08
R2:	0.016	0.036	0.004	0.033	0.021	0.025	0.006
Z-Score:	-2.61	-4.093	-1.101	-1.244	-1.104	-1.652	-0.602
P-Value:	0.009	4.26E-5	0.271	0.214	0.269	0.098	0.547

SNP rs2523393 (chr. 6, 29813638 bp)
Probe 6840408 (chr. 6, 6532340 - 6601215 bp), LY86
P-Value 2.06E-7

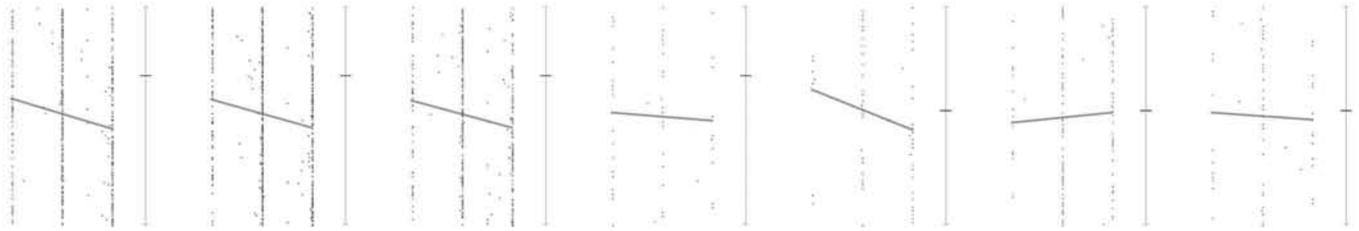

	HT12-Controls	HT12-COPD	HT12-ALS	HT12-UC	H8v2-Controls	H8v2-CeliacDisease	H8v2-ALS
Genotypes	GG (72) GA (196) AA (146)	GG (75) GA (221) AA (157)	GG (53) GA (158) AA (113)	GG (18) GA (20) AA (11)	GG (7) GA (25) AA (27)	GG (14) GA (54) AA (43)	GG (12) GA (32) AA (15)
Corr:	-0.169	-0.157	-0.145	-0.044	-0.209	0.054	-0.035
R2:	0.028	0.025	0.021	0.002	0.044	0.003	0.001
Z-Score:	-3.444	-3.347	-2.617	-0.298	-1.587	0.562	-0.26
P-Value:	5.74E-4	8.18E-4	0.009	0.766	0.112	0.574	0.795

SNP rs4397868 (chr. 11, 133072195 bp)
Probe Human_RefSeq-8_v2-4730132 (chr. 12, 38434090 - 38787158 bp), SLC2A13
P-Value 2.01E-7

HT12-Controls
eQTL not available
Probe not present.

HT12-COPD
eQTL not available
Probe not present.

HT12-ALS
eQTL not available
Probe not present.

HT12-UC
eQTL not available
Probe not present.

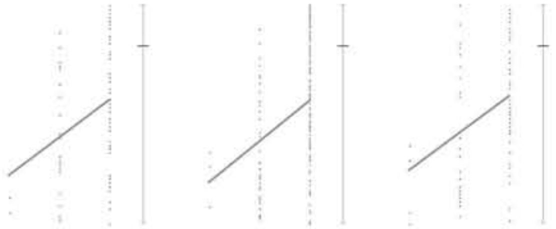

	H8v2-Controls	H8v2-CeliacDisease	H8v2-ALS
Genotypes	CC (2) CT (19) TT (38)	CC (3) CT (31) TT (77)	CC (4) CT (21) TT (34)
Corr:	0.324	0.336	0.359
R2:	0.105	0.113	0.129
Z-Score:	2.504	3.611	2.793
P-Value:	0.012	3.05E-4	0.005

SNP rs2647044 (chr. 6, 32775888 bp)
Probe 1110300 (chr. 4, 153765727 - 153821641 bp), TMEM154
P-Value 1.75E-7

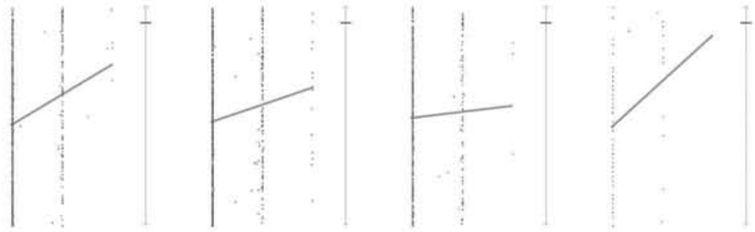

	HT12-Controls	HT12-COPD	HT12-ALS	HT12-UC
Genotypes	GG (307) GA (101) AA (6)	GG (325) GA (114) AA (14)	GG (242) GA (79) AA (3)	GG (38) GA (11) AA (0)
Corr:	0.22	0.14	0.044	0.292
R2:	0.049	0.02	0.002	0.086
Z-Score:	4.528	2.988	0.79	2.039
P-Value:	5.94E-6	0.003	0.43	0.041

H8v2-Controls
eQTL not available
Probe not present.

H8v2-CeliacDisease
eQTL not available
Probe not present.

H8v2-ALS
eQTL not available
Probe not present.

SNP rs12485738 (chr. 3, 56840816 bp)
Probe 5810685 (chr. 15, 37659572 - 37682845 bp), THBS1
P-Value 1.7E-7

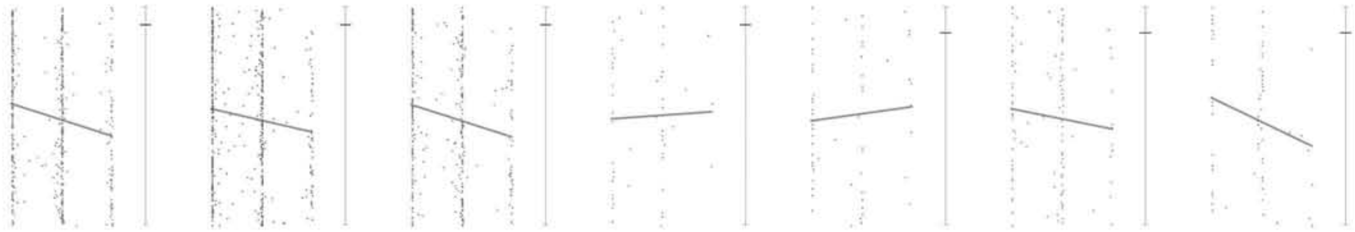

HT12-Controls
GG (153) GA (194) AA (67)
Corr: -0.174 **R2:** 0.03
Z-Score: -3.564 **P-Value:** 3.65E-4

HT12-COPD
GG (202) GA (200) AA (51)
Corr: -0.121 **R2:** 0.015
Z-Score: -2.585 **P-Value:** 0.01

HT12-ALS
GG (132) GA (148) AA (44)
Corr: -0.168 **R2:** 0.028
Z-Score: -3.029 **P-Value:** 0.002

HT12-UC
GG (24) GA (22) AA (3)
Corr: 0.031 **R2:** 0.001
Z-Score: 0.214 **P-Value:** 0.831

H8v2-Controls
GG (28) GA (21) AA (10)
Corr: 0.075 **R2:** 0.006
Z-Score: 0.568 **P-Value:** 0.57

H8v2-CeliacDisease
GG (41) GA (57) AA (13)
Corr: -0.099 **R2:** 0.01
Z-Score: -1.039 **P-Value:** 0.299

H8v2-ALS
GG (20) GA (32) AA (7)
Corr: -0.231 **R2:** 0.053
Z-Score: -1.762 **P-Value:** 0.078

SNP rs11602954 (chr. 11, 192856 bp)
Probe 1050292 (chr. 3, 130261335 - 130264935 bp), GP9
P-Value 1.64E-7

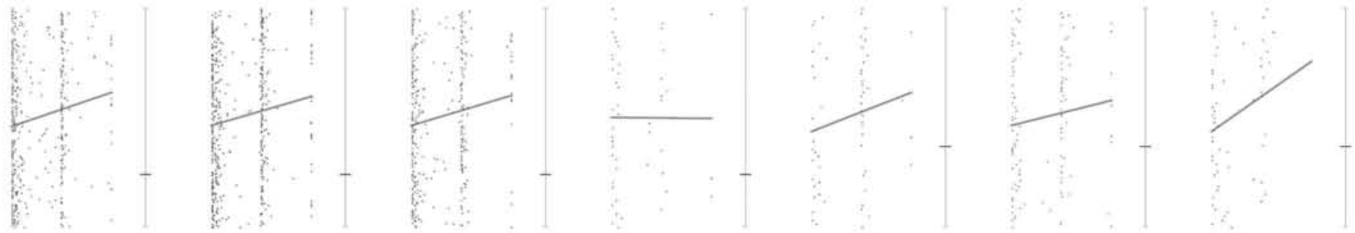

HT12-Controls
GG (248) GA (143) AA (23)
Corr: 0.144 **R2:** 0.021
Z-Score: 2.934 **P-Value:** 0.003

HT12-COPD
GG (261) GA (165) AA (27)
Corr: 0.134 **R2:** 0.018
Z-Score: 2.853 **P-Value:** 0.004

HT12-ALS
GG (190) GA (116) AA (18)
Corr: 0.134 **R2:** 0.018
Z-Score: 2.419 **P-Value:** 0.016

HT12-UC
GG (31) GA (15) AA (3)
Corr: -0.002 **R2:** 0
Z-Score: -0.012 **P-Value:** 0.99

H8v2-Controls
GG (26) GA (29) AA (4)
Corr: 0.174 **R2:** 0.03
Z-Score: 1.317 **P-Value:** 0.188

H8v2-CeliacDisease
GG (56) GA (47) AA (8)
Corr: 0.115 **R2:** 0.013
Z-Score: 1.197 **P-Value:** 0.231

H8v2-ALS
GG (40) GA (19) AA (0)
Corr: 0.239 **R2:** 0.057
Z-Score: 1.821 **P-Value:** 0.069

SNP rs12485738 (chr. 3, 56840816 bp)
Probe 2340577 (chr. 1, 152561435 - 152578414 bp), AQP10///ATP8B2
P-Value 1.61E-7

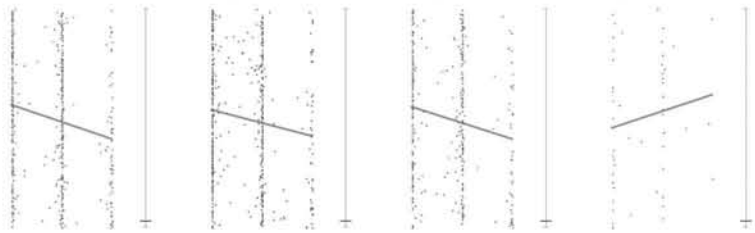

HT12-Controls
GG (153) GA (194) AA (67)
Corr: -0.187 **R2:** 0.035
Z-Score: -3.818 **P-Value:** 1.35E-4

HT12-COPD
GG (202) GA (200) AA (51)
Corr: -0.133 **R2:** 0.018
Z-Score: -2.839 **P-Value:** 0.005

HT12-ALS
GG (132) GA (148) AA (44)
Corr: -0.165 **R2:** 0.027
Z-Score: -2.984 **P-Value:** 0.003

HT12-UC
GG (24) GA (22) AA (3)
Corr: 0.153 **R2:** 0.023
Z-Score: 1.048 **P-Value:** 0.295

H8v2-Controls
eQTL not available
Probe not present.

H8v2-CeliacDisease
eQTL not available
Probe not present.

H8v2-ALS
eQTL not available
Probe not present.

SNP rs425105 (chr. 19, 51900321 bp)
Probe 1090390 (chr. 12, 111828122 - 111855177 bp), OAS1
P-Value 1.58E-7

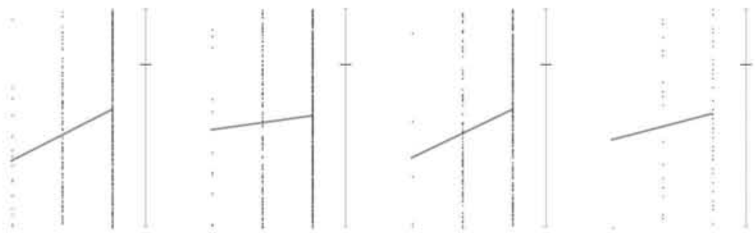

HT12-Controls
CC (15) CT (109) TT (290)
Corr: 0.22 **R2:** 0.048
Z-Score: 4.509 **P-Value:** 6.5E-6

HT12-COPD
CC (11) CT (119) TT (323)
Corr: 0.056 **R2:** 0.003
Z-Score: 1.18 **P-Value:** 0.238

HT12-ALS
CC (5) CT (99) TT (220)
Corr: 0.191 **R2:** 0.036
Z-Score: 3.455 **P-Value:** 5.5E-4

HT12-UC
CC (1) CT (16) TT (32)
Corr: 0.108 **R2:** 0.012
Z-Score: 0.736 **P-Value:** 0.461

H8v2-Controls
eQTL not available
Probe not present.

H8v2-CeliacDisease
eQTL not available
Probe not present.

H8v2-ALS
eQTL not available
Probe not present.

SNP rs12718597 (chr. 7, 50395922 bp)
Probe 3290161 (chr. 19, 60428928 - 60433444 bp), TMEM86B
P-Value 1.43E-7

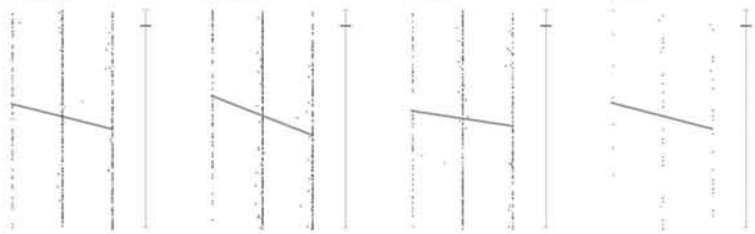

HT12-Controls
AA (64) AC (197) CC (153)
Corr: -0.138 **R2:** 0.019
Z-Score: -2.83 **P-Value:** 0.005

HT12-COPD
AA (68) AC (226) CC (159)
Corr: -0.209 **R2:** 0.044
Z-Score: -4.492 **P-Value:** 7.05E-6

HT12-ALS
AA (56) AC (163) CC (105)
Corr: -0.079 **R2:** 0.006
Z-Score: -1.42 **P-Value:** 0.156

HT12-UC
AA (7) AC (23) CC (19)
Corr: -0.138 **R2:** 0.019
Z-Score: -0.946 **P-Value:** 0.344

H8v2-Controls
eQTL not available
Probe not present.

H8v2-CeliacDisease
eQTL not available
Probe not present.

H8v2-ALS
eQTL not available
Probe not present.

SNP rs11171739 (chr. 12, 54756892 bp)
Probe 3450750 (chr. 19, 62729505 - 62744936 bp), ZNF549
P-Value 1.3E-7

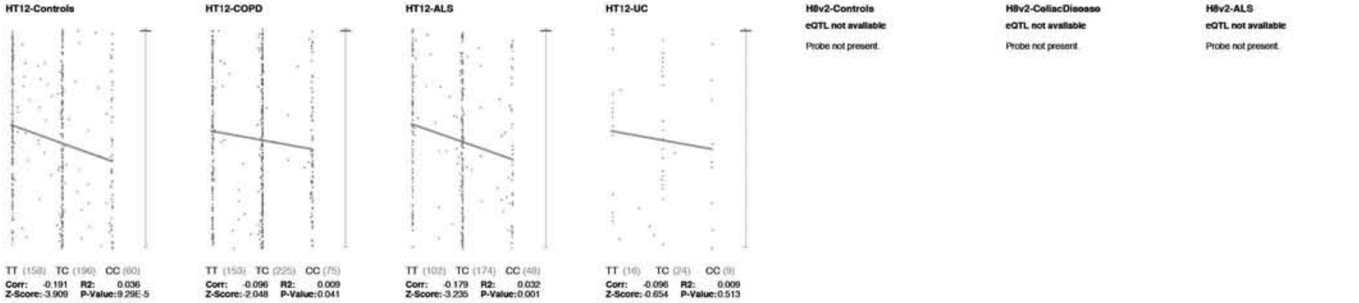

SNP rs2292239 (chr. 12, 54768447 bp)
Probe 5090026 (chr. 9, 100744959 - 100873889 bp), COL15A1
P-Value 1.26E-7

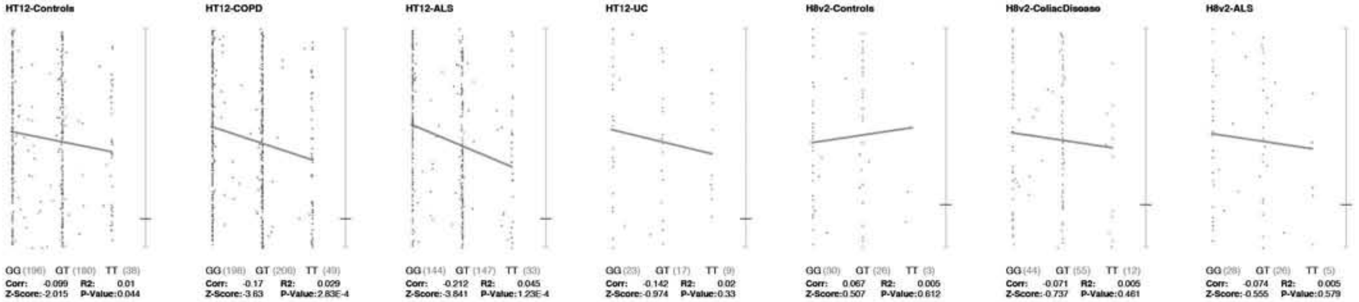

SNP rs9357152 (chr. 6, 32772938 bp)
Probe 7610167 (chr. 19, 45975011 - 45995687 bp), RAB4B
P-Value 1.21E-7

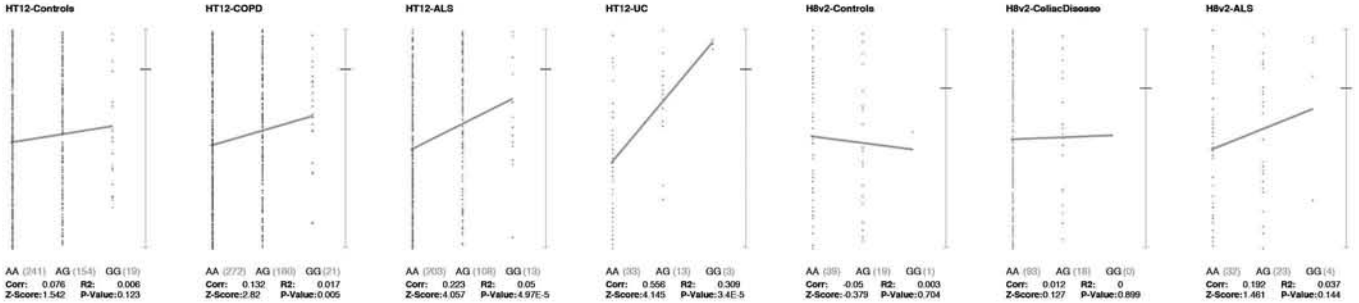

SNP rs11602954 (chr. 11, 192856 bp)
Probe 1980315 (chr. 5, 24871568 - 24871568 bp), -
P-Value 1.12E-7

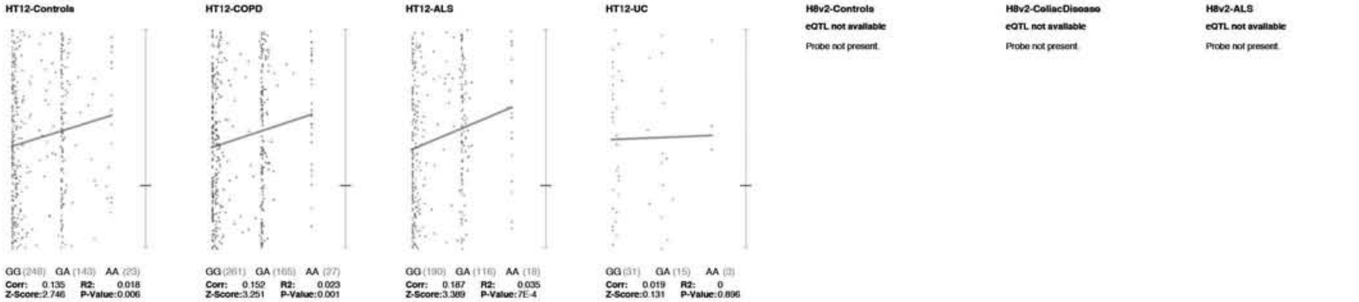

SNP rs8321 (chr. 6, 30140501 bp)
Probe 1110300 (chr. 4, 153765727 - 153821641 bp), TMEM154
P-Value 1.05E-7

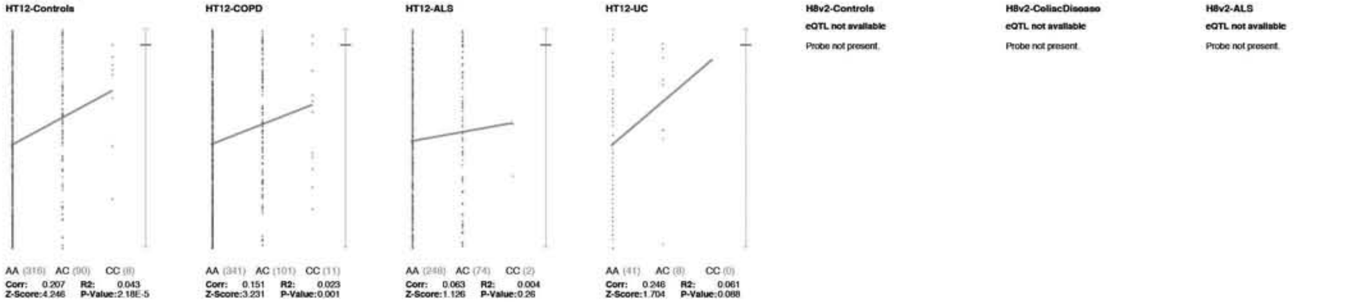

SNP rs1427407 (chr. 2, 60571547 bp)
Probe Human_RefSeq-8_v2-6400079 (chr. 11, 5229996 - 5429091 bp), HBG2///ENST00000399563
P-Value 1.05E-7

HT12-Controls
eQTL not available
Probe not present.

HT12-COPD
eQTL not available
Probe not present.

HT12-ALS
eQTL not available
Probe not present.

HT12-UC
eQTL not available
Probe not present.

H8v2-Controls | **H8v2-CeliacDisease** | **H8v2-ALS**

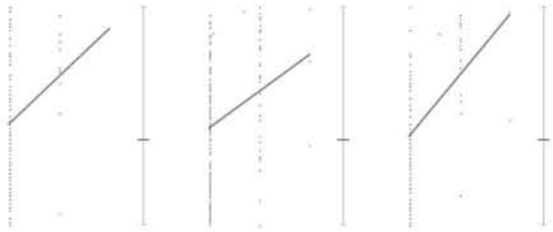

H8v2-Controls: GG (50) GT (9) TT (0)
Corr: 0.266 R2: 0.071
Z-Score:2.039 P-Value:0.041

H8v2-CeliacDisease: GG (79) GT (30) TT (3)
Corr: 0.302 R2: 0.091
Z-Score:3.221 P-Value:0.001

H8v2-ALS: GG (42) GT (15) TT (2)
Corr: 0.499 R2: 0.249
Z-Score:4.021 P-Value:5.79E-5

SNP rs1701704 (chr. 12, 54698754 bp)
Probe 5090026 (chr. 9, 100744959 - 100873889 bp), COL15A1
P-Value 1.03E-7

HT12-Controls | **HT12-COPD** | **HT12-ALS** | **HT12-UC** | **H8v2-Controls** | **H8v2-CeliacDisease** | **H8v2-ALS**

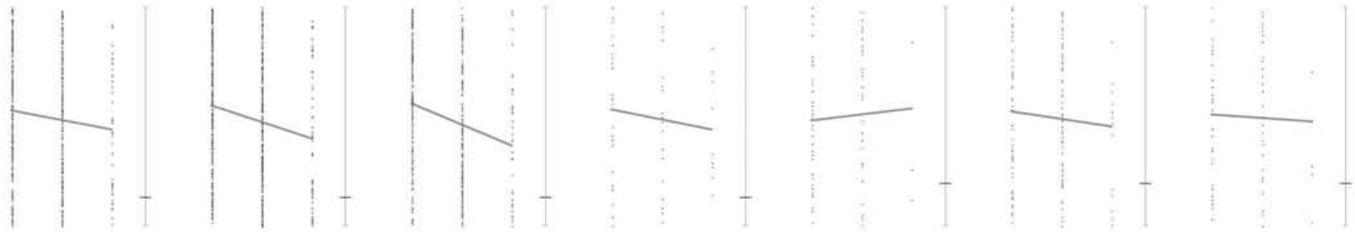

HT12-Controls: TT (193) TG (178) GG (43)
Corr: -0.1 R2: 0.01
Z-Score:-2.034 P-Value:0.042

HT12-COPD: TT (196) TG (206) GG (51)
Corr: -0.171 R2: 0.029
Z-Score:-3.654 P-Value:2.58E-4

HT12-ALS: TT (145) TG (146) GG (33)
Corr: -0.22 R2: 0.048
Z-Score:-3.991 P-Value:6.59E-5

HT12-UC: TT (22) TG (18) GG (9)
Corr: -0.119 R2: 0.014
Z-Score:-0.817 P-Value:0.414

H8v2-Controls: TT (31) TG (25) GG (3)
Corr: 0.055 R2: 0.003
Z-Score:0.417 P-Value:0.677

H8v2-CeliacDisease: TT (44) TG (54) GG (13)
Corr: -0.075 R2: 0.006
Z-Score:-0.782 P-Value:0.434

H8v2-ALS: TT (27) TG (27) GG (5)
Corr: -0.036 R2: 0.001
Z-Score:-0.271 P-Value:0.786

SNP rs9268645 (chr. 6, 32516505 bp)
Probe 2570703 (chr. 21, 38672819 - 38956488 bp), ERG
P-Value 9.6E-8

HT12-Controls | **HT12-COPD** | **HT12-ALS** | **HT12-UC** | **H8v2-Controls** | **H8v2-CeliacDisease** | **H8v2-ALS**

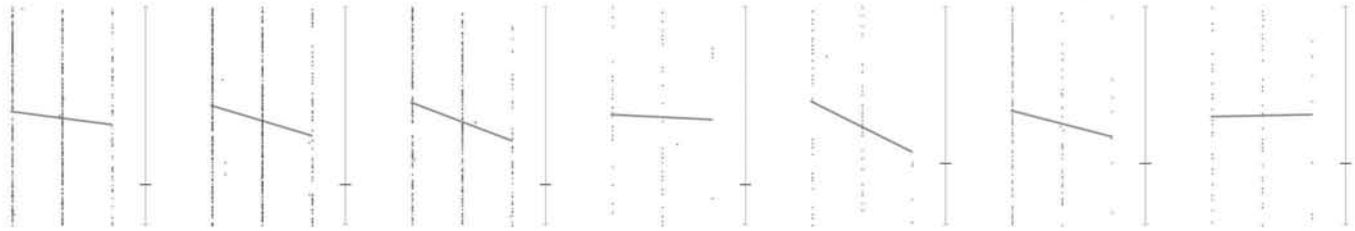

HT12-Controls: CC (172) CG (187) GG (55)
Corr: -0.074 R2: 0.005
Z-Score:-1.502 P-Value:0.133

HT12-COPD: CC (194) CG (197) GG (62)
Corr: -0.168 R2: 0.028
Z-Score:-3.582 P-Value:3.41E-4

HT12-ALS: CC (139) CG (140) GG (45)
Corr: -0.213 R2: 0.045
Z-Score:-3.857 P-Value:1.15E-4

HT12-UC: CC (20) CG (25) GG (4)
Corr: -0.025 R2: 0.001
Z-Score:-0.17 P-Value:0.865

H8v2-Controls: CC (29) CG (26) GG (4)
Corr: -0.238 R2: 0.057
Z-Score:-1.815 P-Value:0.07

H8v2-CeliacDisease: CC (73) CG (31) GG (7)
Corr: -0.122 R2: 0.015
Z-Score:-1.272 P-Value:0.203

H8v2-ALS: CC (24) CG (28) GG (7)
Corr: 0.012 R2: 0
Z-Score:0.087 P-Value:0.93

SNP rs9399137 (chr. 6, 135460711 bp)
Probe 6960615 (chr. 16, 1932977 - 1945672 bp), RPL3L
P-Value 9.59E-8

HT12-Controls | **HT12-COPD** | **HT12-ALS** | **HT12-UC** | **H8v2-Controls** | **H8v2-CeliacDisease** | **H8v2-ALS**

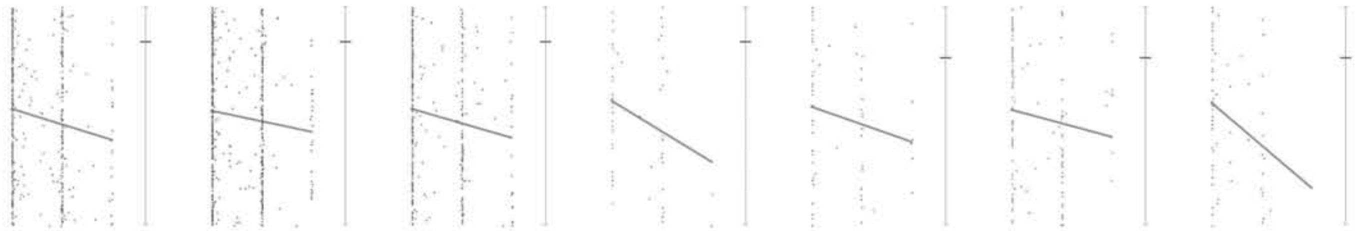

HT12-Controls: TT (233) TC (158) CC (23)
Corr: -0.144 R2: 0.021
Z-Score:-2.935 P-Value:0.003

HT12-COPD: TT (255) TC (173) CC (25)
Corr: -0.098 R2: 0.01
Z-Score:-2.08 P-Value:0.038

HT12-ALS: TT (188) TC (114) CC (22)
Corr: -0.14 R2: 0.02
Z-Score:-2.522 P-Value:0.012

HT12-UC: TT (25) TC (22) CC (2)
Corr: -0.266 R2: 0.071
Z-Score:-1.847 P-Value:0.065

H8v2-Controls: TT (34) TC (19) CC (6)
Corr: -0.181 R2: 0.033
Z-Score:-1.374 P-Value:0.17

H8v2-CeliacDisease: TT (65) TC (37) CC (9)
Corr: -0.134 R2: 0.018
Z-Score:-1.407 P-Value:0.159

H8v2-ALS: TT (42) TC (17) CC (0)
Corr: -0.283 R2: 0.08
Z-Score:-2.17 P-Value:0.03

SNP rs7776054 (chr. 6, 135460609 bp)
Probe 6960615 (chr. 16, 1932977 - 1945672 bp), RPL3L
P-Value 9.43E-8

HT12-Controls | **HT12-COPD** | **HT12-ALS** | **HT12-UC** | **H8v2-Controls** | **H8v2-CeliacDisease** | **H8v2-ALS**

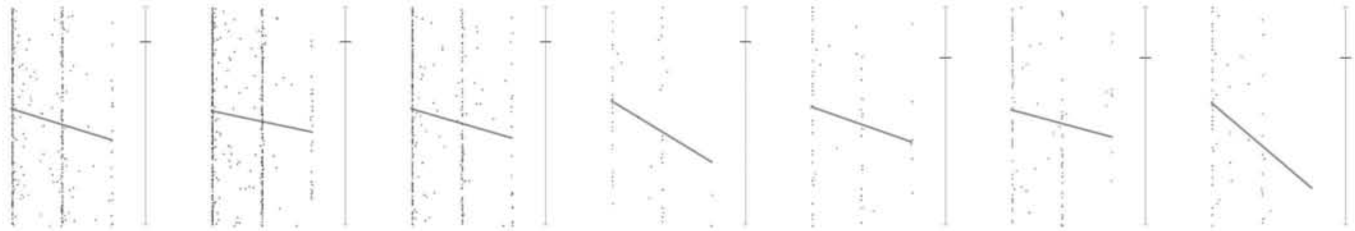

HT12-Controls: AA (233) AG (158) GG (23)
Corr: -0.144 R2: 0.021
Z-Score:-2.933 P-Value:0.003

HT12-COPD: AA (255) AG (173) GG (25)
Corr: -0.098 R2: 0.01
Z-Score:-2.081 P-Value:0.037

HT12-ALS: AA (188) AG (114) GG (22)
Corr: -0.14 R2: 0.02
Z-Score:-2.52 P-Value:0.012

HT12-UC: AA (25) AG (22) GG (2)
Corr: -0.266 R2: 0.071
Z-Score:-1.844 P-Value:0.065

H8v2-Controls: AA (34) AG (19) GG (6)
Corr: -0.181 R2: 0.033
Z-Score:-1.374 P-Value:0.17

H8v2-CeliacDisease: AA (65) AG (37) GG (9)
Corr: -0.135 R2: 0.018
Z-Score:-1.408 P-Value:0.159

H8v2-ALS: AA (42) AG (17) GG (0)
Corr: -0.283 R2: 0.08
Z-Score:-2.174 P-Value:0.03

SNP rs6756629 (chr. 2, 43918594 bp)
Probe 3120368 (chr. 3, 9910358 - 9934079 bp), IL17RE
P-Value 8.69E-8

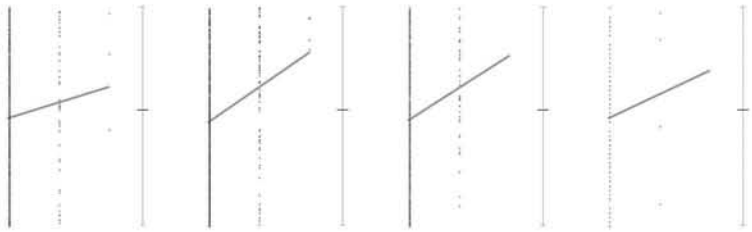

Dataset	Genotypes	Corr	R2	Z-Score	P-Value
HT12-Controls	GG (372) GA (39) AA (3)	0.081	0.007	1.65	0.099
HT12-COPD	GG (381) GA (68) AA (4)	0.217	0.047	4.669	3.03E-6
HT12-ALS	GG (289) GA (35) AA (0)	0.157	0.025	2.83	0.005
HT12-UC	GG (45) GA (4) AA (0)	0.1	0.01	0.685	0.494

H8v2-Controls
eQTL not available
Probe not present.

H8v2-CeliacDisease
eQTL not available
Probe not present.

H8v2-ALS
eQTL not available
Probe not present.

SNP rs2523393 (chr. 6, 29813638 bp)
Probe 4200025 (chr. 11, 119585957 - 119606859 bp), OAF
P-Value 8.57E-8

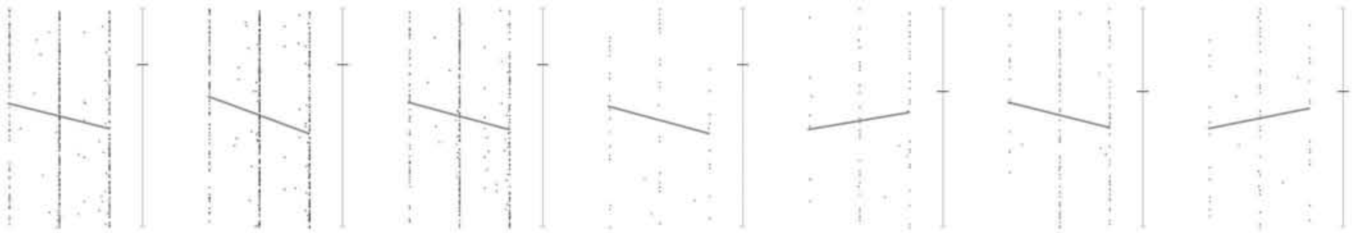

Dataset	Genotypes	Corr	R2	Z-Score	P-Value
HT12-Controls	GG (72) GA (196) AA (146)	-0.14	0.02	-2.862	0.004
HT12-COPD	GG (75) GA (221) AA (157)	-0.197	0.039	-4.219	2.45E-5
HT12-ALS	GG (53) GA (158) AA (113)	-0.144	0.021	-2.604	0.009
HT12-UC	GG (18) GA (20) AA (11)	-0.153	0.023	-1.051	0.293
H8v2-Controls	GG (7) GA (25) AA (27)	0.087	0.008	0.654	0.513
H8v2-CeliacDisease	GG (14) GA (54) AA (43)	-0.129	0.017	-1.347	0.178
H8v2-ALS	GG (12) GA (32) AA (15)	0.103	0.011	0.773	0.44

SNP rs7758512 (chr. 6, 30078568 bp)
Probe 4290600 (chr. 20, 60551199 - 60560176 bp), C20orf200
P-Value 8.35E-8

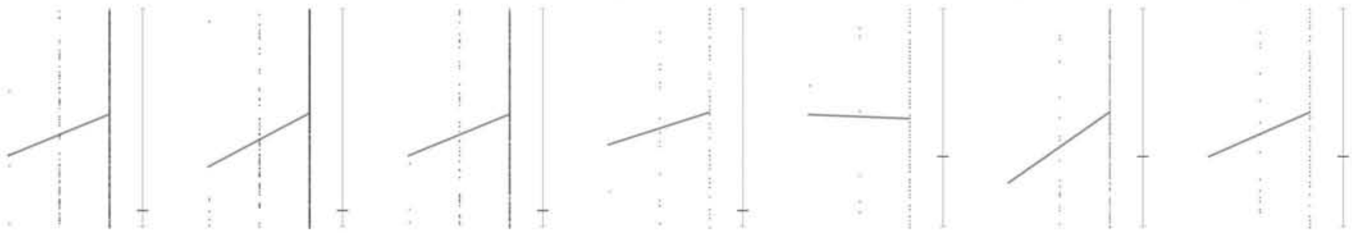

Dataset	Genotypes	Corr	R2	Z-Score	P-Value
HT12-Controls	GG (4) GT (64) TT (346)	0.13	0.017	2.655	0.008
HT12-COPD	GG (6) GT (58) TT (389)	0.167	0.028	3.561	3.7E-4
HT12-ALS	GG (3) GT (44) TT (277)	0.125	0.016	2.257	0.024
HT12-UC	GG (1) GT (14) TT (34)	0.124	0.015	0.851	0.395
H8v2-Controls	GG (1) GT (6) TT (52)	-0.01	0	-0.077	0.939
H8v2-CeliacDisease	GG (0) GT (16) TT (95)	0.192	0.037	2.02	0.043
H8v2-ALS	GG (0) GT (14) TT (45)	0.145	0.021	1.096	0.273

SNP rs3129934 (chr. 6, 32444165 bp)
Probe 3180504 (chr. 11, 66938721 - 66950652 bp), ATPGD1
P-Value 7.8E-8

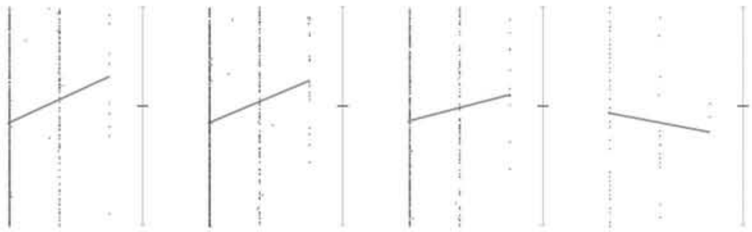

Dataset	Genotypes	Corr	R2	Z-Score	P-Value
HT12-Controls	CC (303) CT (102) TT (9)	0.183	0.034	3.745	1.8E-4
HT12-COPD	CC (331) CT (104) TT (18)	0.178	0.032	3.813	1.37E-4
HT12-ALS	CC (229) CT (84) TT (11)	0.111	0.012	1.993	0.046
HT12-UC	CC (34) CT (13) TT (2)	-0.084	0.007	-0.57	0.568

H8v2-Controls
eQTL not available
Probe not present.

H8v2-CeliacDisease
eQTL not available
Probe not present.

H8v2-ALS
eQTL not available
Probe not present.

SNP rs12718597 (chr. 7, 50395922 bp)
Probe 830309 (chr. 17, 68714519 - 68728596 bp), FAM104A///COG1
P-Value 7.32E-8

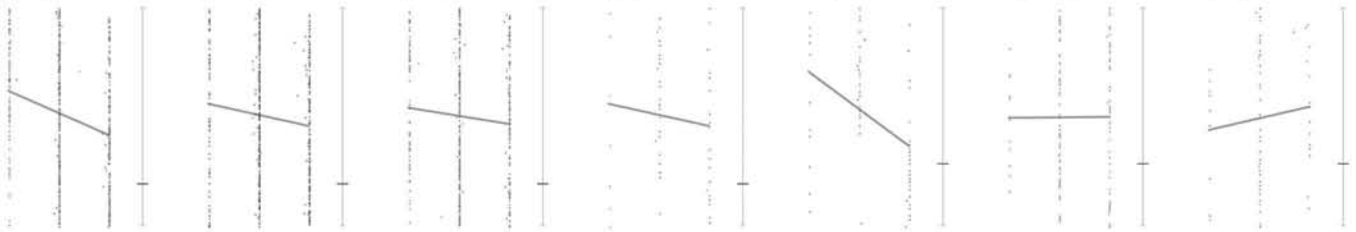

Dataset	Genotypes	Corr	R2	Z-Score	P-Value
HT12-Controls	AA (64) AC (197) CC (153)	-0.238	0.057	-4.906	9.31E-7
HT12-COPD	AA (68) AC (226) CC (159)	-0.118	0.014	-2.503	0.012
HT12-ALS	AA (56) AC (163) CC (105)	-0.089	0.008	-1.59	0.112
HT12-UC	AA (7) AC (23) CC (19)	-0.115	0.013	-0.787	0.431
H8v2-Controls	AA (11) AC (23) CC (25)	-0.428	0.183	-3.382	7.19E-4
H8v2-CeliacDisease	AA (11) AC (53) CC (47)	0.005	0	0.051	0.96
H8v2-ALS	AA (9) AC (35) CC (15)	0.113	0.013	0.849	0.396

SNP rs2517713 (chr. 6, 30026078 bp)
Probe 60093 (chr. 8, 102766947 - 103207311 bp), NCALD
P-Value 6.81E-8

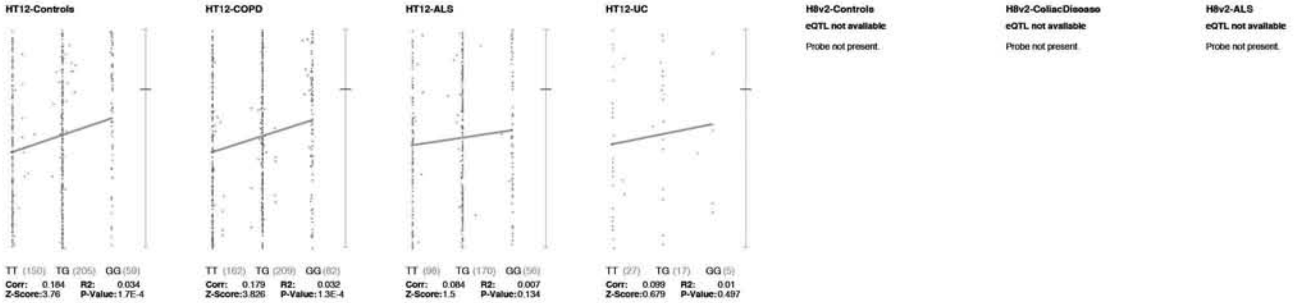

SNP rs11171739 (chr. 12, 54756892 bp)
Probe 3190671 (chr. 19, 1191749 - 1196823 bp), ATP5D
P-Value 5.99E-8

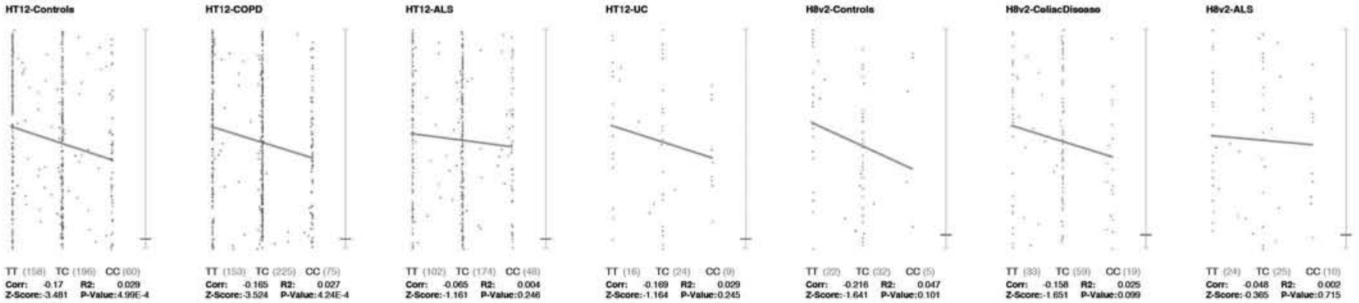

SNP rs12485738 (chr. 3, 56840816 bp)
Probe 4860681 (chr. 4, 156806328 - 156873926 bp), GUCY1A3
P-Value 6.05E-8

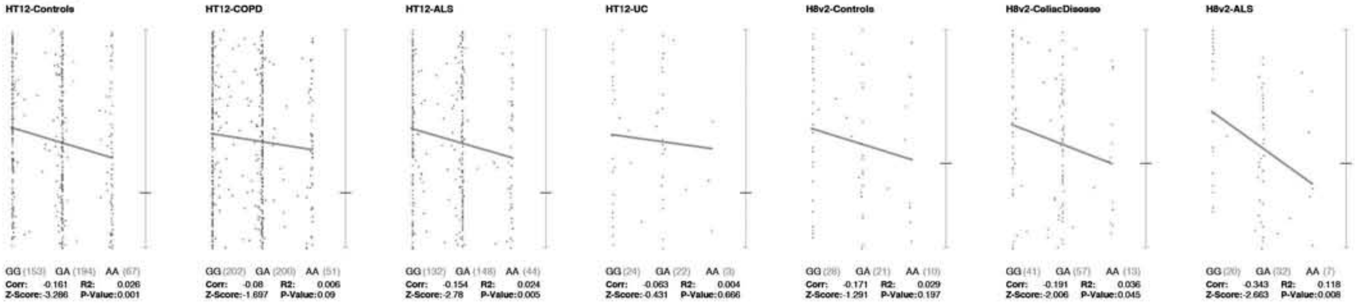

SNP rs3131296 (chr. 6, 32280971 bp)
Probe 1110300 (chr. 4, 153765727 - 153821641 bp), TMEM154
P-Value 5.66E-8

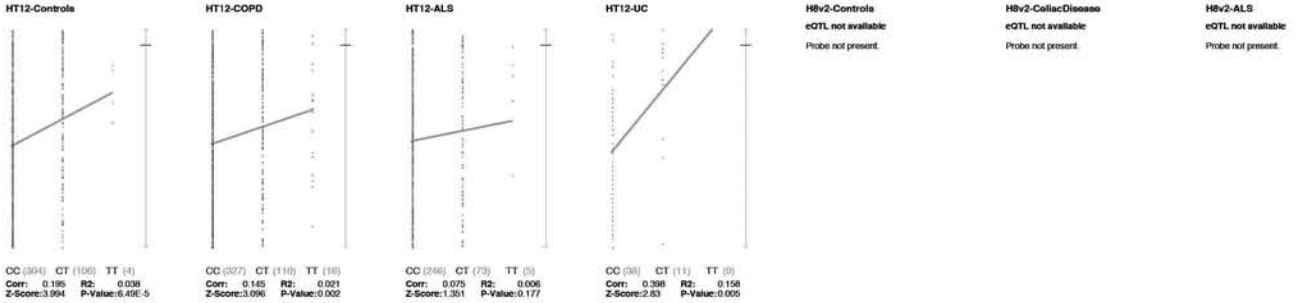

SNP rs7743761 (chr. 6, 31444079 bp)
Probe 6380347 (chr. 22, 40346241 - 40390998 bp), XRCC6
P-Value 5.68E-8

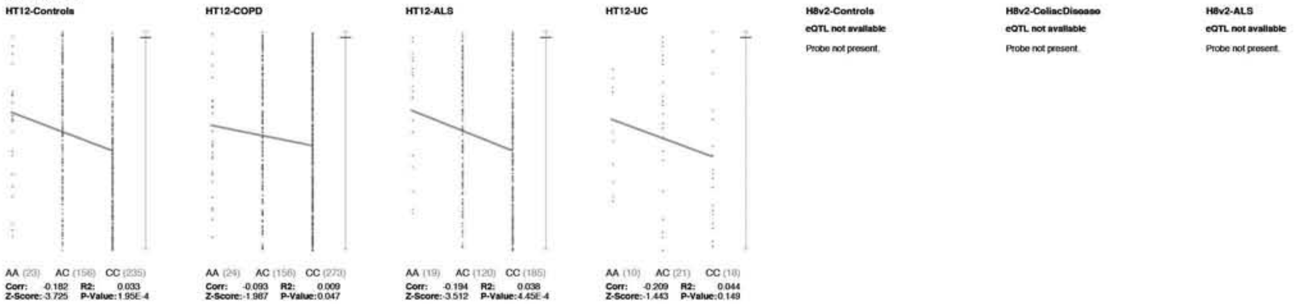

SNP rs494620 (chr. 6, 31946692 bp)
Probe 1110300 (chr. 4, 153765727 - 153821641 bp), TMEM154
P-Value 5.01E-8

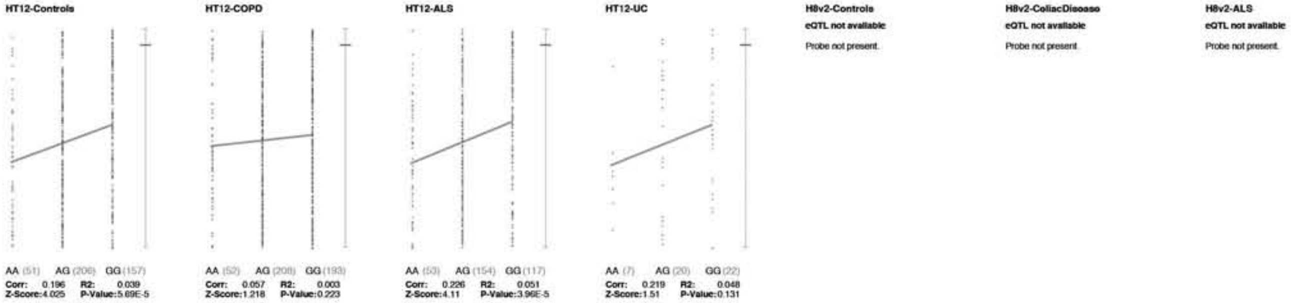

SNP rs2292239 (chr. 12, 54768447 bp)
Probe 3450750 (chr. 19, 62729505 - 62744936 bp), ZNF549
P-Value 4.89E-8

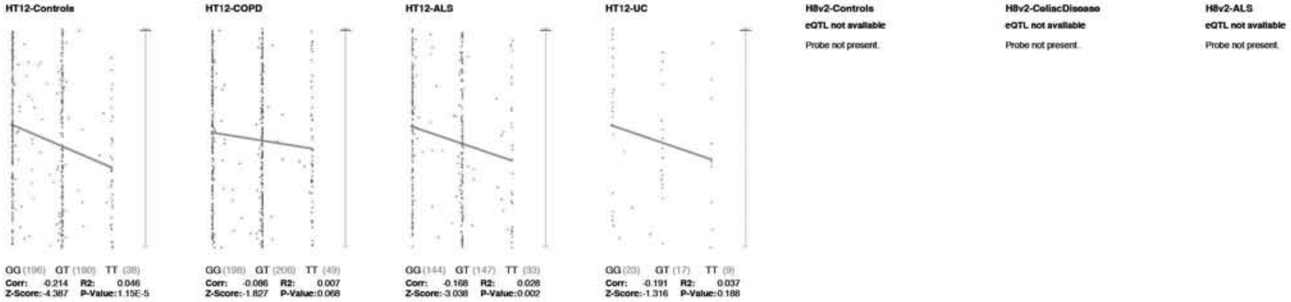

SNP rs6445975 (chr. 3, 58345217 bp)
Probe 450370 (chr. 16, 83103029 - 83103029 bp), -
P-Value 4.87E-8

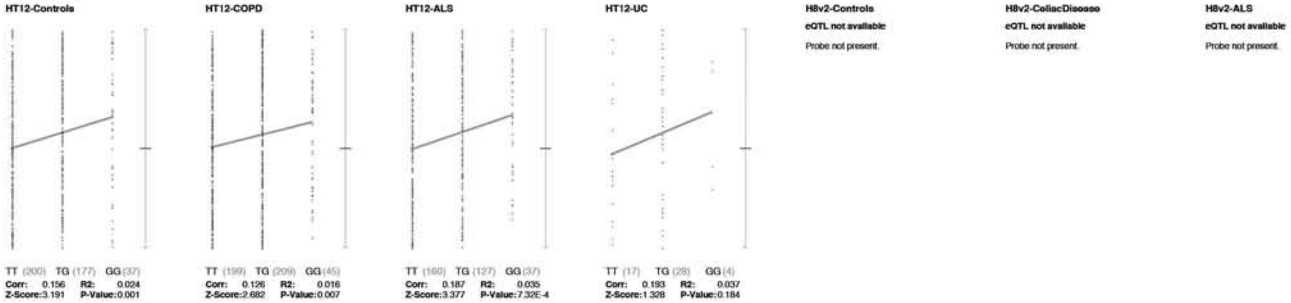

SNP rs11171739 (chr. 12, 54756892 bp)
Probe 1030333 (chr. 17, 29605409 - 29609329 bp), CCL2
P-Value 4.93E-8

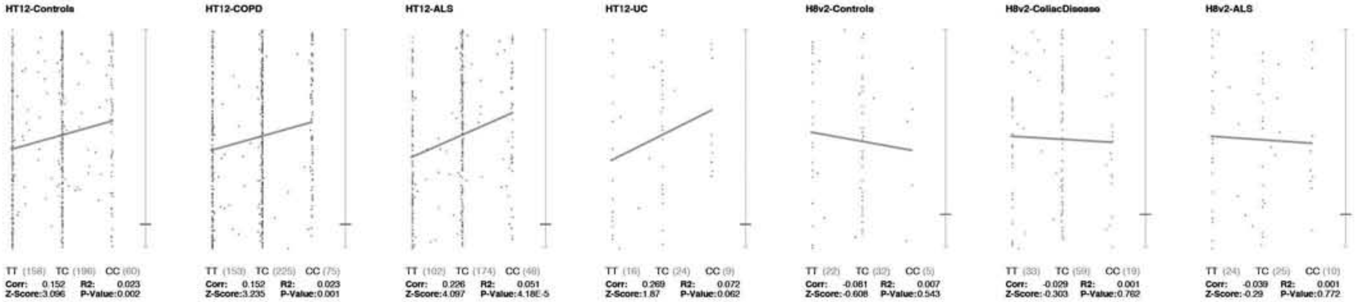

SNP rs11171739 (chr. 12, 54756892 bp)
Probe 2850112 (chr. 9, 137963637 - 137994047 bp), UBAC1
P-Value 4.6E-8

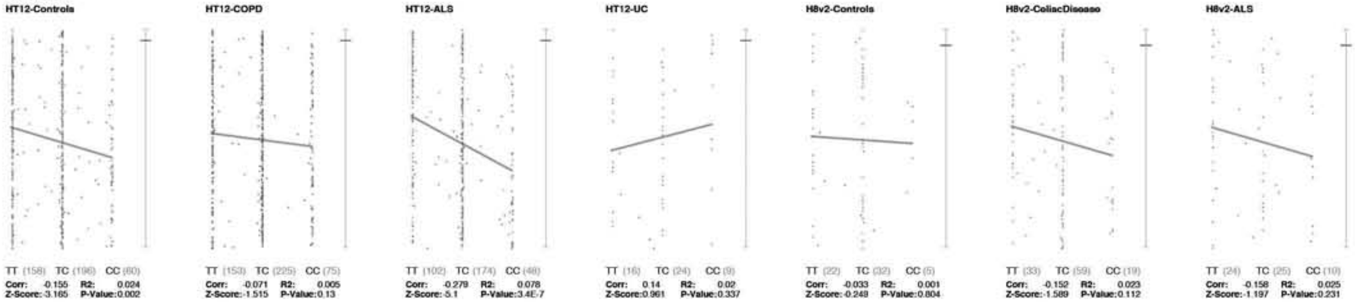

SNP rs643381 (chr. 6, 139881116 bp)
Probe 3290369 (chr. 20, 1040906 - 1098022 bp), PSMF1
P-Value 4.43E-8

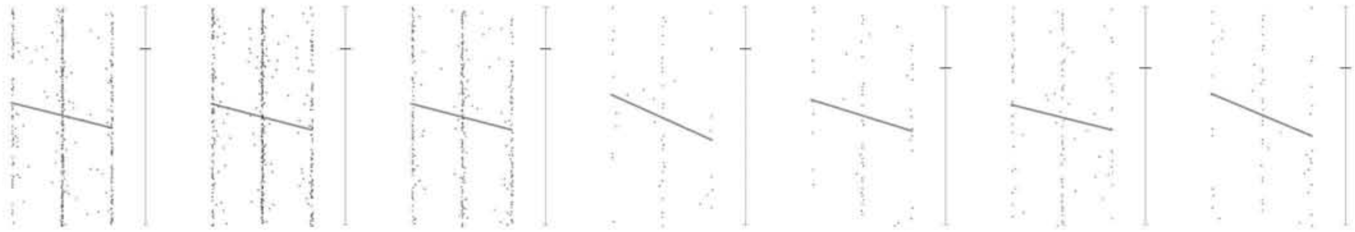

HT12-Controls
AA (82) AC (216) CC (116)
Corr: -0.129 **R2:** 0.017
Z-Score:-2.627 **P-Value:**0.009

HT12-COPD
AA (116) AC (238) CC (101)
Corr: -0.133 **R2:** 0.018
Z-Score:-2.842 **P-Value:**0.004

HT12-ALS
AA (95) AC (149) CC (80)
Corr: -0.151 **R2:** 0.023
Z-Score:-2.715 **P-Value:**0.007

HT12-UC
AA (11) AC (29) CC (9)
Corr: -0.211 **R2:** 0.044
Z-Score:-1.452 **P-Value:**0.147

H8v2-Controls
AA (11) AC (31) CC (17)
Corr: -0.161 **R2:** 0.026
Z-Score:-1.218 **P-Value:**0.223

H8v2-ColiacDisease
AA (30) AC (57) CC (24)
Corr: -0.128 **R2:** 0.016
Z-Score:-1.339 **P-Value:**0.18

H8v2-ALS
AA (14) AC (28) CC (17)
Corr: -0.227 **R2:** 0.051
Z-Score:-1.725 **P-Value:**0.085

SNP rs628751 (chr. 6, 139880112 bp)
Probe 3290369 (chr. 20, 1040906 - 1098022 bp), PSMF1
P-Value 4.4E-8

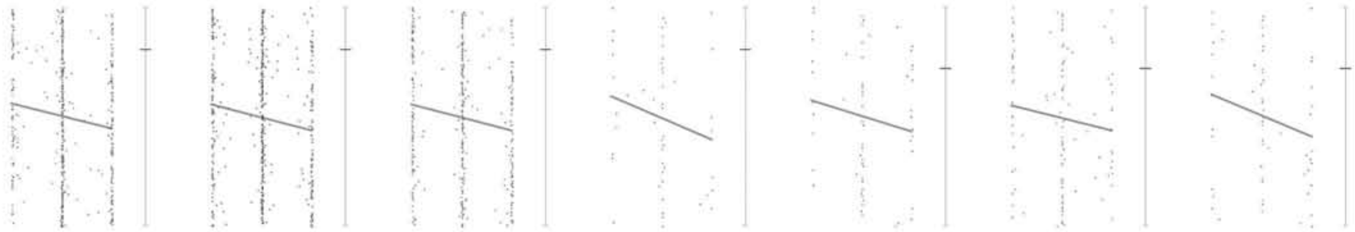

HT12-Controls
AA (82) AC (216) CC (116)
Corr: -0.129 **R2:** 0.017
Z-Score:-2.63 **P-Value:**0.009

HT12-COPD
AA (116) AC (236) CC (101)
Corr: -0.134 **R2:** 0.018
Z-Score:-2.852 **P-Value:**0.004

HT12-ALS
AA (95) AC (149) CC (80)
Corr: -0.15 **R2:** 0.022
Z-Score:-2.705 **P-Value:**0.007

HT12-UC
AA (11) AC (29) CC (9)
Corr: -0.208 **R2:** 0.043
Z-Score:-1.438 **P-Value:**0.151

H8v2-Controls
AA (11) AC (31) CC (17)
Corr: -0.162 **R2:** 0.026
Z-Score:-1.225 **P-Value:**0.22

H8v2-ColiacDisease
AA (30) AC (57) CC (24)
Corr: -0.128 **R2:** 0.016
Z-Score:-1.336 **P-Value:**0.181

H8v2-ALS
AA (14) AC (28) CC (17)
Corr: -0.227 **R2:** 0.051
Z-Score:-1.727 **P-Value:**0.084

SNP rs12718597 (chr. 7, 50395922 bp)
Probe 1780538 (chr. 23, 152605586 - 152616234 bp), SLC6A8
P-Value 4.33E-8

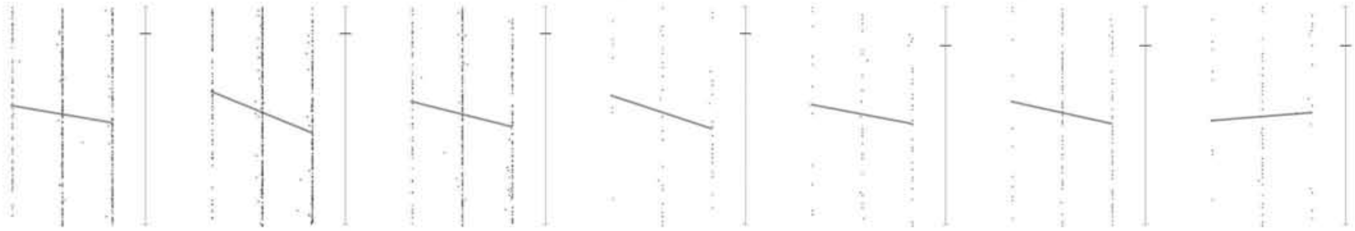

HT12-Controls
AA (64) AC (197) CC (153)
Corr: -0.095 **R2:** 0.009
Z-Score:-1.924 **P-Value:**0.054

HT12-COPD
AA (68) AC (226) CC (159)
Corr: -0.222 **R2:** 0.049
Z-Score:-4.779 **P-Value:**1.76E-6

HT12-ALS
AA (56) AC (163) CC (105)
Corr: -0.137 **R2:** 0.019
Z-Score:-2.47 **P-Value:**0.014

HT12-UC
AA (7) AC (23) CC (19)
Corr: -0.175 **R2:** 0.031
Z-Score:-1.203 **P-Value:**0.229

H8v2-Controls
AA (11) AC (23) CC (25)
Corr: -0.107 **R2:** 0.012
Z-Score:-0.808 **P-Value:**0.419

H8v2-ColiacDisease
AA (11) AC (53) CC (47)
Corr: -0.113 **R2:** 0.013
Z-Score:-1.176 **P-Value:**0.239

H8v2-ALS
AA (9) AC (35) CC (15)
Corr: 0.042 **R2:** 0.002
Z-Score:0.313 **P-Value:**0.754

SNP rs13437082 (chr. 6, 31462539 bp)
Probe 6380347 (chr. 22, 40346241 - 40390998 bp), XRCC6
P-Value 4.14E-8

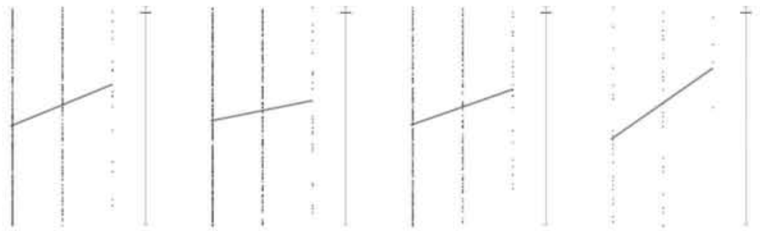

HT12-Controls
CC (248) CT (149) TT (17)
Corr: 0.185 **R2:** 0.034
Z-Score:3.793 **P-Value:**1.49E-4

HT12-COPD
CC (260) CT (169) TT (24)
Corr: 0.095 **R2:** 0.009
Z-Score:2.03 **P-Value:**0.042

HT12-ALS
CC (188) CT (113) TT (23)
Corr: 0.173 **R2:** 0.03
Z-Score:3.134 **P-Value:**0.002

HT12-UC
CC (22) CT (23) TT (4)
Corr: 0.342 **R2:** 0.117
Z-Score:2.404 **P-Value:**0.016

H8v2-Controls
eQTL not available
Probe not present.

H8v2-ColiacDisease
eQTL not available
Probe not present.

H8v2-ALS
eQTL not available
Probe not present.

SNP rs5770917 (chr. 22, 49364219 bp)
Probe 2940673 (chr. 16, 2957408 - 2957567 bp), KREMEN2
P-Value 3.81E-8

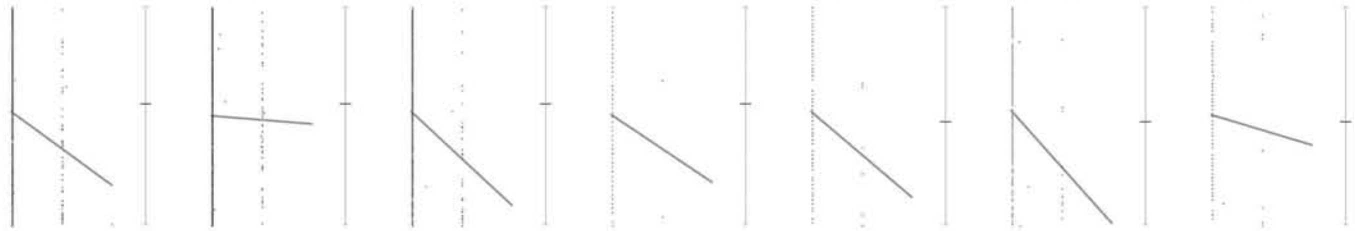

HT12-Controls
TT (365) TC (48) CC (1)
Corr: -0.192 **R2:** 0.037
Z-Score:-3.939 **P-Value:**8.17E-5

HT12-COPD
TT (411) TC (42) CC (0)
Corr: -0.018 **R2:** 0
Z-Score:-0.384 **P-Value:**0.701

HT12-ALS
TT (292) TC (32) CC (0)
Corr: -0.22 **R2:** 0.048
Z-Score:-3.989 **P-Value:**6.64E-5

HT12-UC
TT (47) TC (2) CC (0)
Corr: -0.103 **R2:** 0.011
Z-Score:-0.705 **P-Value:**0.481

H8v2-Controls
TT (53) TC (6) CC (0)
Corr: -0.201 **R2:** 0.04
Z-Score:-1.526 **P-Value:**0.127

H8v2-ColiacDisease
TT (100) TC (11) CC (0)
Corr: -0.266 **R2:** 0.071
Z-Score:-2.817 **P-Value:**0.005

H8v2-ALS
TT (52) TC (7) CC (0)
Corr: -0.076 **R2:** 0.006
Z-Score:-0.573 **P-Value:**0.566

SNP rs12485738 (chr. 3, 56840816 bp)
Probe 1090739 (chr. 2, 108570199 - 108671134 bp), LIMS1
P-Value 3.82E-8

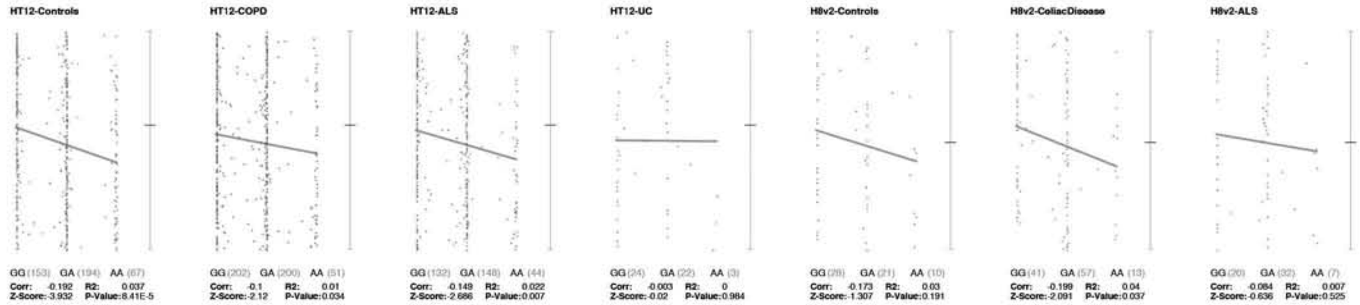

SNP rs12474609 (chr. 2, 141437612 bp)
Probe 5490717 (chr. 9, 139442152 - 139453200 bp), NOXA1///ENTPD8
P-Value 3.3E-8

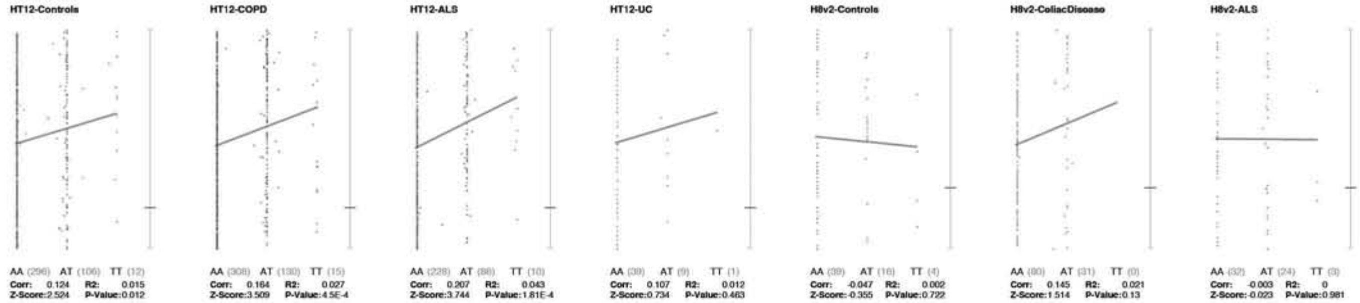

SNP rs11171739 (chr. 12, 54756892 bp)
Probe 2340681 (chr. 3, 158896597 - 159933969 bp), RARRES1
P-Value 2.99E-8

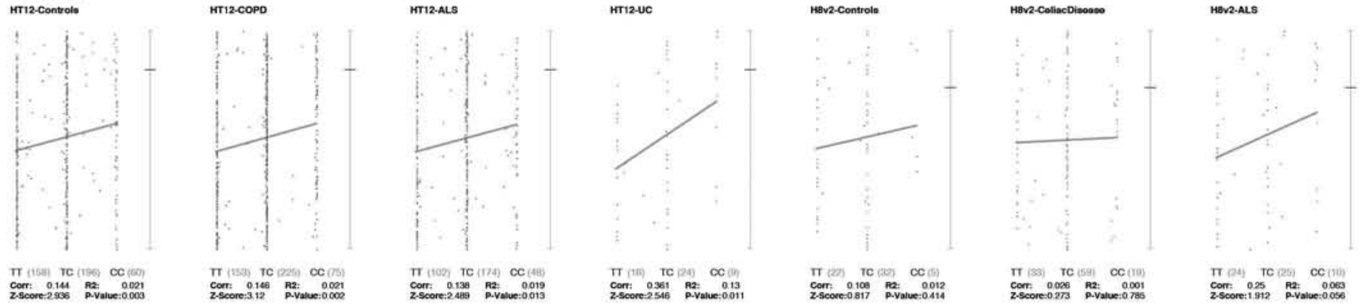

SNP rs1980493 (chr. 6, 32471193 bp)
Probe 2940424 (chr. 7, 36518135 - 36731595 bp), AOAH
P-Value 2.88E-8

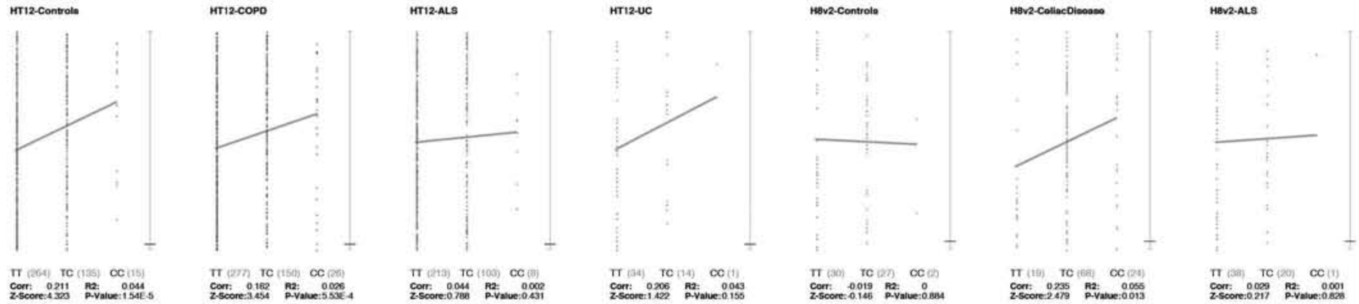

SNP rs259919 (chr. 6, 30133482 bp)
Probe 5080576 (chr. 4, 55217852 - 55302636 bp), KIT
P-Value 2.56E-8

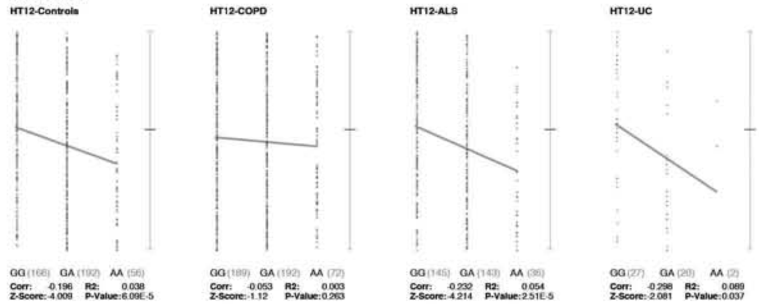

H8v2-Controls
eQTL not available
Probe not present.

H8v2-CeliacDisease
eQTL not available
Probe not present.

H8v2-ALS
eQTL not available
Probe not present.

SNP rs1701704 (chr. 12, 54698754 bp)
Probe 3310592 (chr. 7, 98917628 - 98936260 bp), ZNF789///ZNF394
P-Value 2.55E-8

HT12-Controls | **HT12-COPD** | **HT12-ALS** | **HT12-UC** | **H8v2-Controls** | **H8v2-CeliacDisease** | **H8v2-ALS**

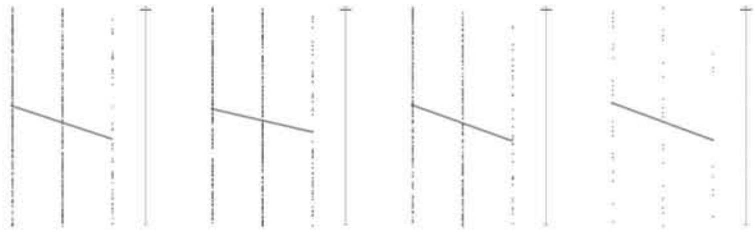

HT12-Controls
TT (193) TG (178) GG (43)
Corr: -0.17 R2: 0.029
Z-Score: -3.474 P-Value: 5.13E-4

HT12-COPD
TT (196) TG (206) GG (51)
Corr: -0.12 R2: 0.014
Z-Score: -2.56 P-Value: 0.01

HT12-ALS
TT (145) TG (146) GG (33)
Corr: -0.186 R2: 0.035
Z-Score: -3.36 P-Value: 7.79E-4

HT12-UC
TT (22) TG (18) GG (9)
Corr: -0.217 R2: 0.047
Z-Score: -1.501 P-Value: 0.133

H8v2-Controls
eQTL not available
Probe not present.

H8v2-CeliacDisease
eQTL not available
Probe not present.

H8v2-ALS
eQTL not available
Probe not present.

SNP rs11602954 (chr. 11, 192856 bp)
Probe 630470 (chr. 19, 43485644 - 43488486 bp), C19orf33
P-Value 2.56E-8

HT12-Controls | **HT12-COPD** | **HT12-ALS** | **HT12-UC** | **H8v2-Controls** | **H8v2-CeliacDisease** | **H8v2-ALS**

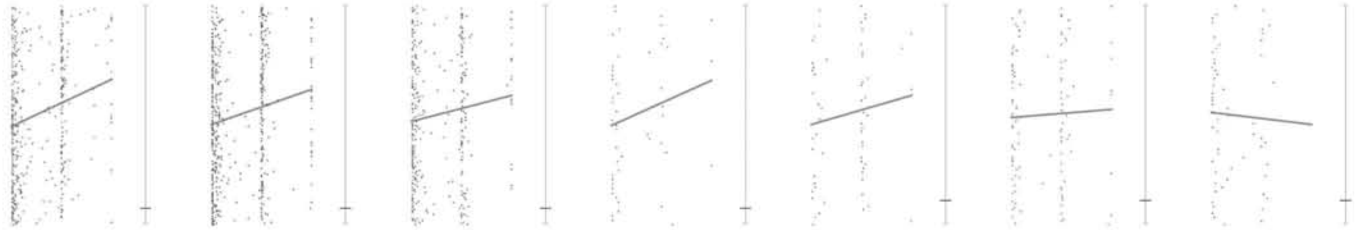

HT12-Controls
GG (248) GA (143) AA (23)
Corr: 0.203 R2: 0.041
Z-Score: 4.164 P-Value: 3.13E-5

HT12-COPD
GG (261) GA (165) AA (27)
Corr: 0.157 R2: 0.025
Z-Score: 3.348 P-Value: 8.13E-4

HT12-ALS
GG (190) GA (116) AA (18)
Corr: 0.119 R2: 0.014
Z-Score: 2.147 P-Value: 0.032

HT12-UC
GG (31) GA (15) AA (3)
Corr: 0.201 R2: 0.04
Z-Score: 1.382 P-Value: 0.167

H8v2-Controls
GG (26) GA (29) AA (4)
Corr: 0.129 R2: 0.017
Z-Score: 0.976 P-Value: 0.329

H8v2-CeliacDisease
GG (56) GA (47) AA (8)
Corr: 0.037 R2: 0.001
Z-Score: 0.38 P-Value: 0.704

H8v2-ALS
GG (40) GA (19) AA (0)
Corr: -0.041 R2: 0.002
Z-Score: -0.307 P-Value: 0.759

SNP rs12485738 (chr. 3, 56840816 bp)
Probe 1740458 (chr. 7, 30140077 - 30169906 bp), C7orf41
P-Value 2.41E-8

HT12-Controls | **HT12-COPD** | **HT12-ALS** | **HT12-UC** | **H8v2-Controls** | **H8v2-CeliacDisease** | **H8v2-ALS**

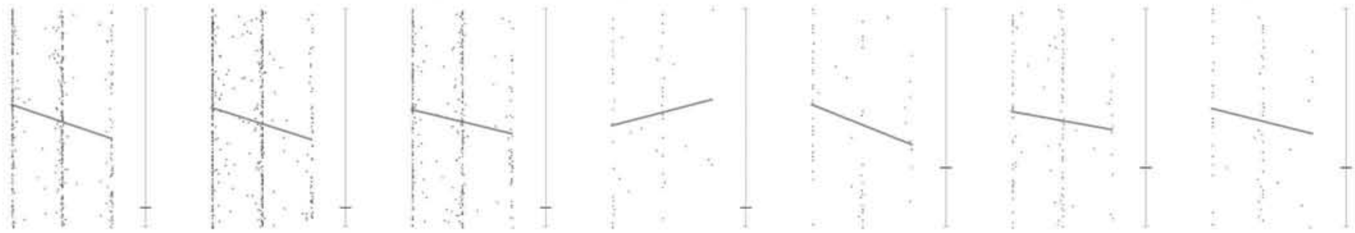

HT12-Controls
GG (153) GA (194) AA (67)
Corr: -0.181 R2: 0.033
Z-Score: -3.697 P-Value: 2.19E-4

HT12-COPD
GG (202) GA (200) AA (51)
Corr: -0.164 R2: 0.027
Z-Score: -3.495 P-Value: 4.74E-4

HT12-ALS
GG (132) GA (148) AA (44)
Corr: -0.125 R2: 0.016
Z-Score: -2.257 P-Value: 0.024

HT12-UC
GG (24) GA (22) AA (3)
Corr: 0.119 R2: 0.014
Z-Score: 0.814 P-Value: 0.416

H8v2-Controls
GG (28) GA (21) AA (10)
Corr: -0.217 R2: 0.047
Z-Score: -1.648 P-Value: 0.099

H8v2-CeliacDisease
GG (41) GA (57) AA (13)
Corr: -0.088 R2: 0.008
Z-Score: -0.919 P-Value: 0.358

H8v2-ALS
GG (20) GA (32) AA (7)
Corr: -0.117 R2: 0.014
Z-Score: -0.882 P-Value: 0.378

SNP rs12485738 (chr. 3, 56840816 bp)
Probe 20075 (chr. 3, 49030497 - 49034803 bp), DALRD3///hsa-mir///C3orf60
P-Value 2.26E-8

HT12-Controls | **HT12-COPD** | **HT12-ALS** | **HT12-UC** | **H8v2-Controls** | **H8v2-CeliacDisease** | **H8v2-ALS**

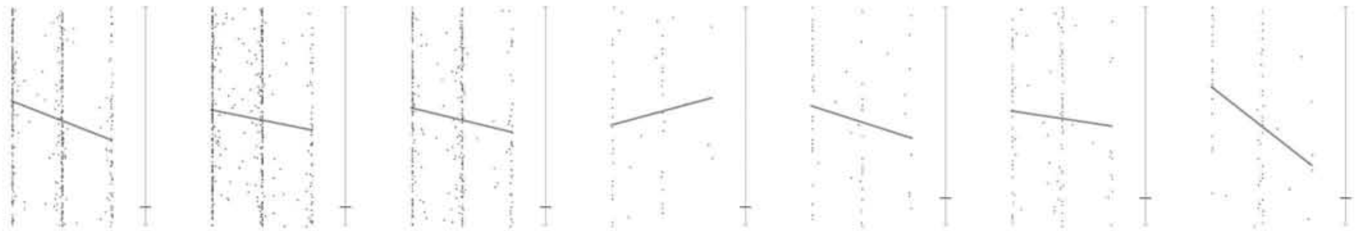

HT12-Controls
GG (153) GA (194) AA (67)
Corr: -0.212 R2: 0.045
Z-Score: -4.342 P-Value: 1.41E-5

HT12-COPD
GG (202) GA (200) AA (51)
Corr: -0.107 R2: 0.012
Z-Score: -2.286 P-Value: 0.022

HT12-ALS
GG (132) GA (148) AA (44)
Corr: -0.13 R2: 0.017
Z-Score: -2.335 P-Value: 0.02

HT12-UC
GG (24) GA (22) AA (3)
Corr: 0.124 R2: 0.015
Z-Score: 0.849 P-Value: 0.396

H8v2-Controls
GG (28) GA (21) AA (10)
Corr: -0.179 R2: 0.032
Z-Score: -1.353 P-Value: 0.176

H8v2-CeliacDisease
GG (41) GA (57) AA (13)
Corr: -0.077 R2: 0.006
Z-Score: -0.8 P-Value: 0.424

H8v2-ALS
GG (20) GA (32) AA (7)
Corr: -0.371 R2: 0.138
Z-Score: -2.895 P-Value: 0.004

SNP rs12718597 (chr. 7, 50395922 bp)
Probe 7610615 (chr. 16, 33240034 - 33250316 bp), SLC6A10P///AC133561.4
P-Value 2.08E-8

HT12-Controls | **HT12-COPD** | **HT12-ALS** | **HT12-UC** | **H8v2-Controls** | **H8v2-CeliacDisease** | **H8v2-ALS**

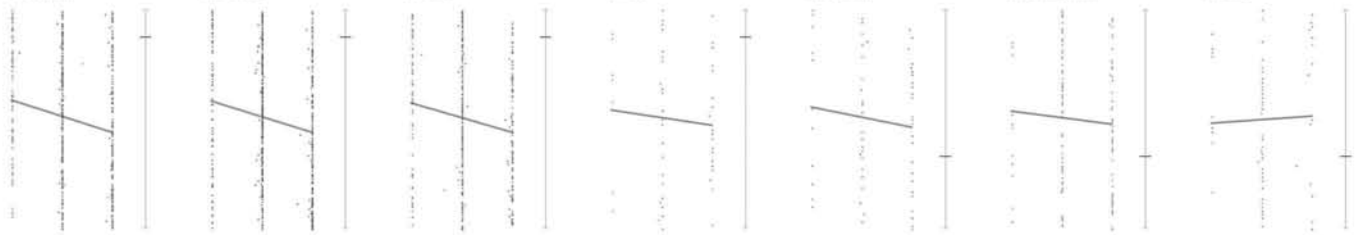

HT12-Controls
AA (64) AC (197) CC (153)
Corr: -0.177 R2: 0.031
Z-Score: -3.628 P-Value: 2.86E-4

HT12-COPD
AA (68) AC (226) CC (159)
Corr: -0.165 R2: 0.027
Z-Score: -3.522 P-Value: 4.28E-4

HT12-ALS
AA (56) AC (163) CC (105)
Corr: -0.154 R2: 0.024
Z-Score: -2.776 P-Value: 0.005

HT12-UC
AA (7) AC (23) CC (19)
Corr: -0.078 R2: 0.006
Z-Score: -0.53 P-Value: 0.596

H8v2-Controls
AA (11) AC (23) CC (25)
Corr: -0.121 R2: 0.015
Z-Score: -0.911 P-Value: 0.362

H8v2-CeliacDisease
AA (11) AC (53) CC (47)
Corr: -0.067 R2: 0.004
Z-Score: -0.694 P-Value: 0.488

H8v2-ALS
AA (9) AC (35) CC (15)
Corr: 0.034 R2: 0.001
Z-Score: 0.252 P-Value: 0.801

SNP rs11171739 (chr. 12, 54756892 bp)
Probe 3370349 (chr. 6, 32020406 - 32028840 bp), CFB
P-Value 1.98E-8

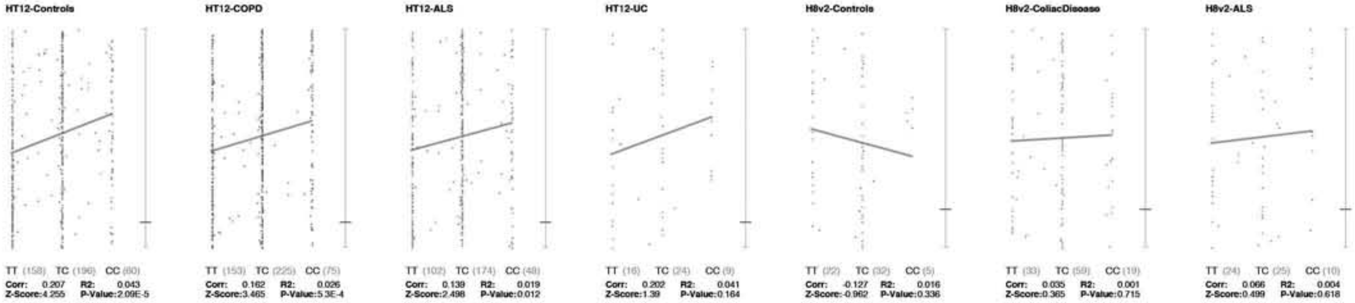

SNP rs2523393 (chr. 6, 29813638 bp)
Probe 3800392 (chr. 8, 145135214 - 145140571 bp), GRINA
P-Value 1.9E-8

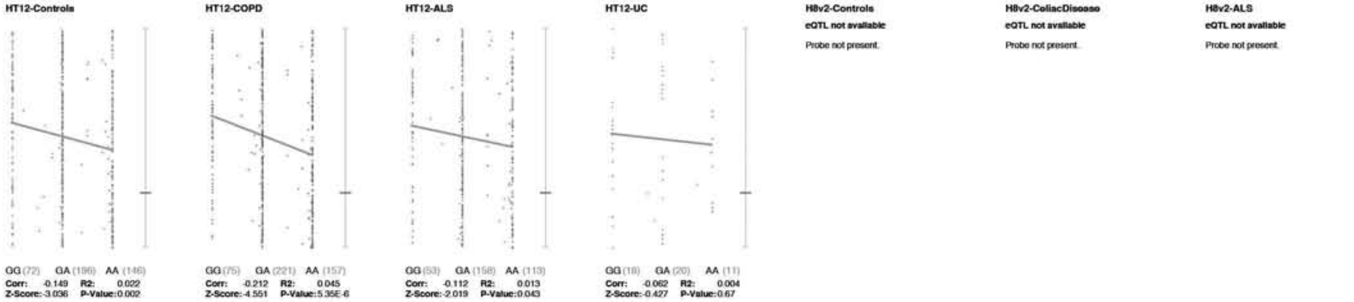

SNP rs2517713 (chr. 6, 30026078 bp)
Probe 7000274 (chr. 9, 118968171 - 118994970 bp), RPL10P3///SNORA70///ASTN2
P-Value 1.69E-8

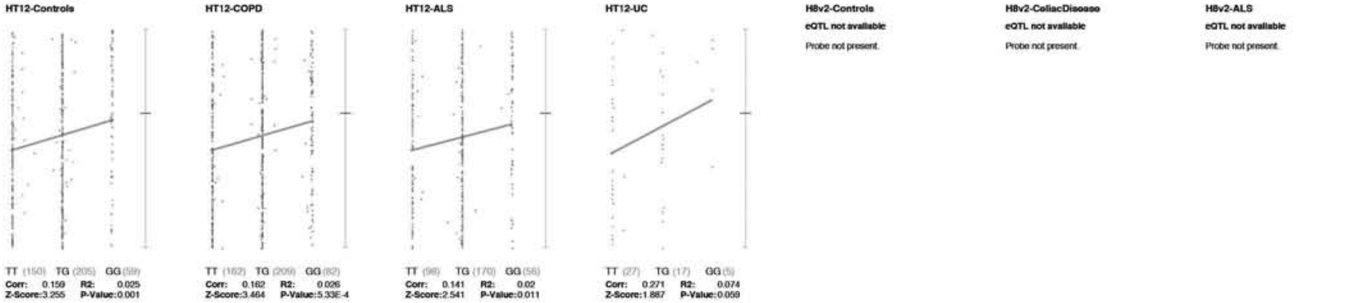

SNP rs2517713 (chr. 6, 30026078 bp)
Probe 7380725 (chr. 17, 70824752 - 70914385 bp), GRB2
P-Value 1.59E-8

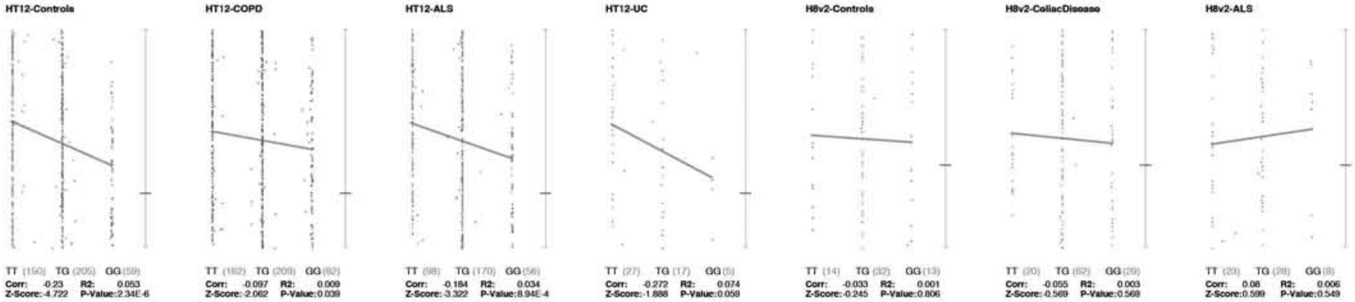

SNP rs12485738 (chr. 3, 56840816 bp)
Probe 5560246 (chr. 15, 61120891 - 61152164 bp), TPM1
P-Value 1.57E-8

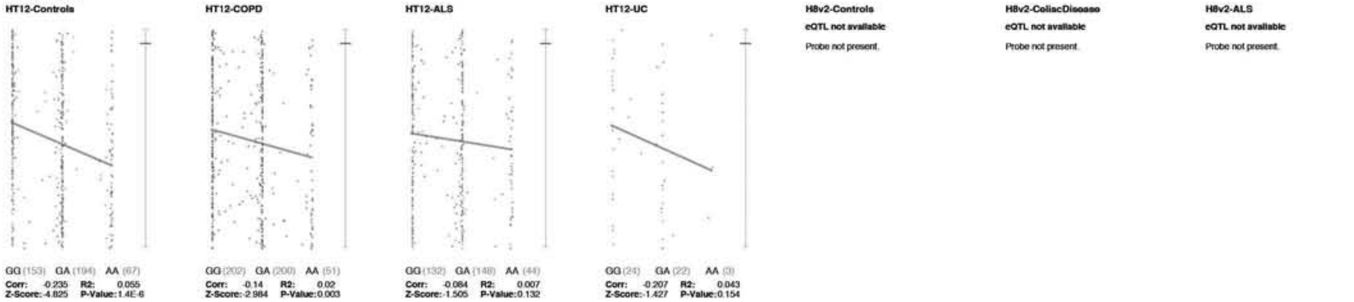

SNP rs12485738 (chr. 3, 56840816 bp)
Probe 2230241 (chr. 6, 6088310 - 6266923 bp), F13A1
P-Value 1.56E-8

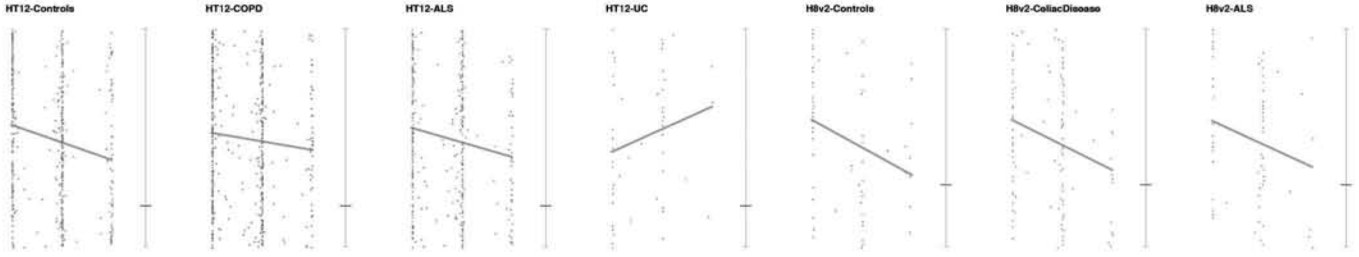

Dataset	Genotypes	Corr	R2	Z-Score	P-Value
HT12-Controls	GG (153) GA (194) AA (67)	-0.193	0.037	-3.947	7.9E-5
HT12-COPD	GG (202) GA (200) AA (51)	-0.09	0.008	-1.91	0.056
HT12-ALS	GG (132) GA (148) AA (44)	-0.147	0.022	-2.659	0.008
HT12-UC	GG (24) GA (22) AA (3)	0.208	0.043	1.435	0.151
H8v2-Controls	GG (28) GA (21) AA (10)	-0.305	0.093	-2.346	0.019
H8v2-CeliacDisease	GG (41) GA (57) AA (13)	-0.244	0.059	-2.578	0.01
H8v2-ALS	GG (20) GA (32) AA (7)	-0.217	0.047	-1.649	0.099

SNP rs4917014 (chr. 7, 50276409 bp)
Probe 780215 (chr. 17, 6917581 - 6925324 bp), CLEC10A
P-Value 1.51E-8

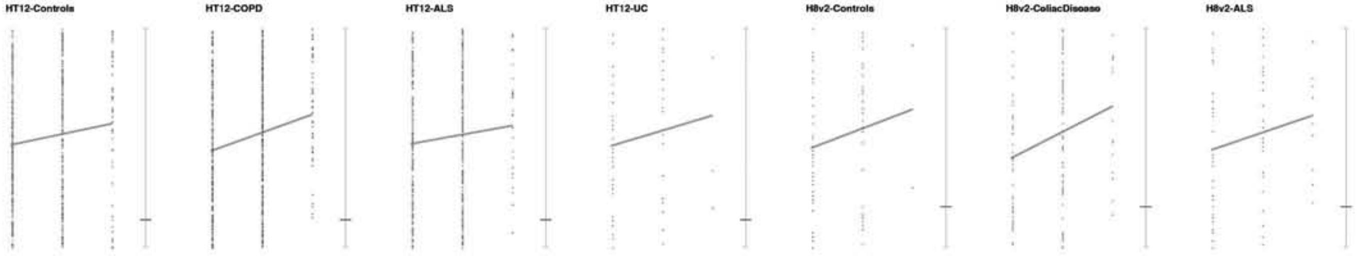

Dataset	Genotypes	Corr	R2	Z-Score	P-Value
HT12-Controls	TT (197) TG (168) GG (49)	0.115	0.013	2.341	0.019
HT12-COPD	TT (185) TG (222) GG (46)	0.182	0.033	3.894	9.87E-5
HT12-ALS	TT (142) TG (155) GG (27)	0.092	0.008	1.646	0.1
HT12-UC	TT (27) TG (19) GG (3)	0.144	0.021	0.989	0.323
H8v2-Controls	TT (32) TG (25) GG (2)	0.166	0.028	1.256	0.209
H8v2-CeliacDisease	TT (39) TG (58) GG (14)	0.26	0.068	2.76	0.006
H8v2-ALS	TT (27) TG (23) GG (9)	0.19	0.036	1.438	0.15

SNP rs131794 (chr. 22, 49318618 bp)
Probe 2940673 (chr. 16, 2957408 - 2957567 bp), KREMEN2
P-Value 1.46E-8

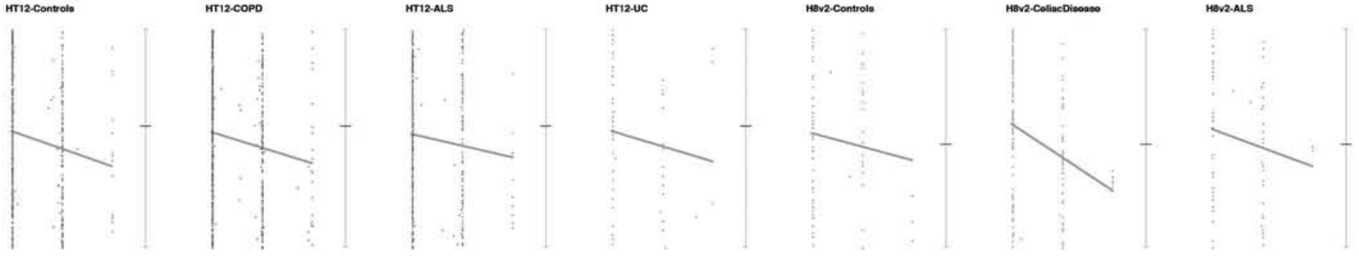

Dataset	Genotypes	Corr	R2	Z-Score	P-Value
HT12-Controls	CC (259) CA (142) AA (13)	-0.152	0.023	-3.109	0.002
HT12-COPD	CC (282) CA (149) AA (22)	-0.141	0.02	-3.012	0.003
HT12-ALS	CC (220) CA (94) AA (10)	-0.097	0.009	-1.738	0.082
HT12-UC	CC (30) CA (14) AA (5)	-0.153	0.023	-1.048	0.295
H8v2-Controls	CC (37) CA (19) AA (3)	-0.122	0.015	-0.92	0.358
H8v2-CeliacDisease	CC (69) CA (36) AA (6)	-0.309	0.095	-3.297	9.79E-4
H8v2-ALS	CC (31) CA (26) AA (2)	-0.163	0.027	-1.233	0.218

SNP rs12485738 (chr. 3, 56840816 bp)
Probe 3830327 (chr. 3, 128889601 - 129041709 bp), MGLL
P-Value 1.45E-8

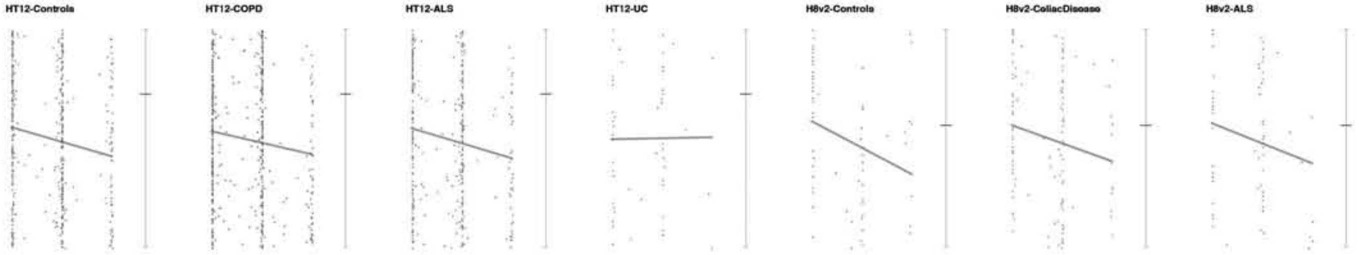

Dataset	Genotypes	Corr	R2	Z-Score	P-Value
HT12-Controls	GG (153) GA (194) AA (67)	-0.158	0.025	-3.216	0.001
HT12-COPD	GG (202) GA (200) AA (51)	-0.119	0.014	-2.536	0.011
HT12-ALS	GG (132) GA (148) AA (44)	-0.155	0.024	-2.794	0.005
HT12-UC	GG (24) GA (22) AA (3)	0.01	0	0.071	0.944
H8v2-Controls	GG (28) GA (21) AA (10)	-0.293	0.086	-2.252	0.024
H8v2-CeliacDisease	GG (41) GA (57) AA (13)	-0.176	0.031	-1.843	0.065
H8v2-ALS	GG (20) GA (32) AA (7)	-0.189	0.036	-1.432	0.152

SNP rs2517713 (chr. 6, 30026078 bp)
Probe 5390608 (chr. 17, 35313374 - 35329429 bp), GSDMB
P-Value 1.4E-8

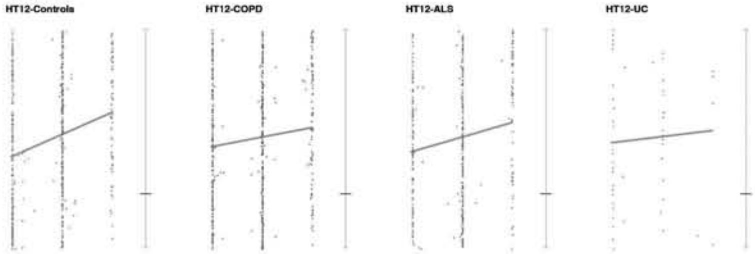

Dataset	Genotypes	Corr	R2	Z-Score	P-Value
HT12-Controls	TT (150) TG (205) GG (59)	0.231	0.053	4.75	2.04E-6
HT12-COPD	TT (162) TG (209) GG (82)	0.108	0.012	2.299	0.021
HT12-ALS	TT (98) TG (170) GG (56)	0.158	0.025	2.848	0.004
HT12-UC	TT (27) TG (17) GG (5)	0.06	0.004	0.408	0.683

H8v2-Controls
eQTL not available
Probe not present.

H8v2-CeliacDisease
eQTL not available
Probe not present.

H8v2-ALS
eQTL not available
Probe not present.

SNP rs4324798 (chr. 6, 28884096 bp)
Probe 6380347 (chr. 22, 40346241 - 40390998 bp), XRCC6
P-Value 1.43E-8

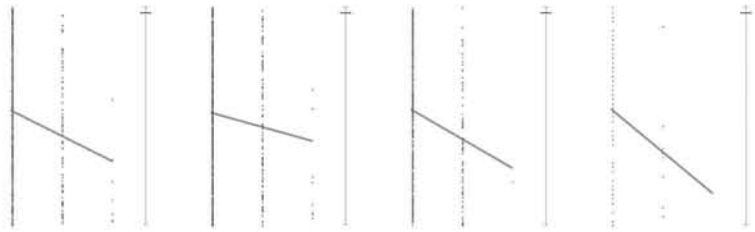

HT12-Controls
GG (324) GA (83) AA (7)
Corr: -0.185 **R2:** 0.034
Z-Score: -3.788 **P-Value:** 1.52E-4

HT12-COPD
GG (348) GA (97) AA (8)
Corr: -0.105 **R2:** 0.011
Z-Score: -2.238 **P-Value:** 0.025

HT12-ALS
GG (253) GA (70) AA (1)
Corr: -0.195 **R2:** 0.038
Z-Score: -3.537 **P-Value:** 4.04E-4

HT12-UC
GG (41) GA (8) AA (0)
Corr: -0.238 **R2:** 0.057
Z-Score: -1.648 **P-Value:** 0.099

H8v2-Controls
eQTL not available
Probe not present.

H8v2-CeliacDisease
eQTL not available
Probe not present.

H8v2-ALS
eQTL not available
Probe not present.

SNP rs9268877 (chr. 6, 32539125 bp)
Probe 2940424 (chr. 7, 36518135 - 36731595 bp), AOAH
P-Value 1.36E-8

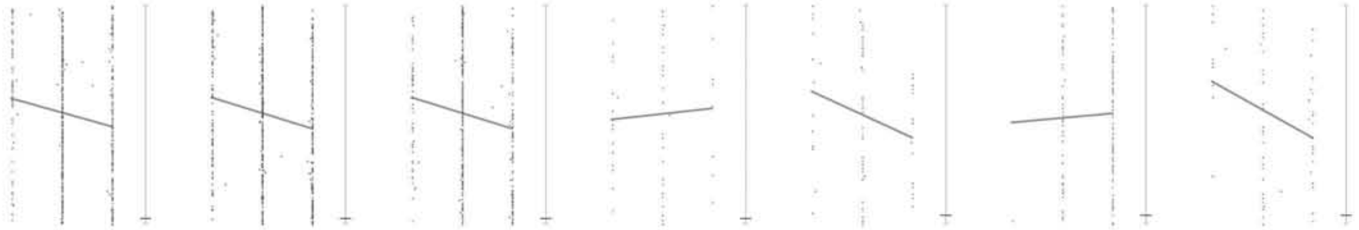

HT12-Controls
AA (61) AG (212) GG (141)
Corr: -0.15 **R2:** 0.023
Z-Score: -3.068 **P-Value:** 0.002

HT12-COPD
AA (91) AG (214) GG (148)
Corr: -0.172 **R2:** 0.03
Z-Score: -3.682 **P-Value:** 2.32E-4

HT12-ALS
AA (50) AG (179) GG (95)
Corr: -0.16 **R2:** 0.026
Z-Score: -2.888 **P-Value:** 0.004

HT12-UC
AA (16) AG (25) GG (8)
Corr: 0.055 **R2:** 0.003
Z-Score: 0.375 **P-Value:** 0.708

H8v2-Controls
AA (13) AG (32) GG (14)
Corr: -0.237 **R2:** 0.056
Z-Score: -1.806 **P-Value:** 0.071

H8v2-CeliacDisease
AA (1) AG (42) GG (68)
Corr: 0.037 **R2:** 0.001
Z-Score: 0.391 **P-Value:** 0.696

H8v2-ALS
AA (10) AG (28) GG (21)
Corr: -0.303 **R2:** 0.092
Z-Score: -2.333 **P-Value:** 0.02

SNP rs12485738 (chr. 3, 56840816 bp)
Probe 1410010 (chr. 10, 96986324 - 97041771 bp), PDLIM1
P-Value 1.26E-8

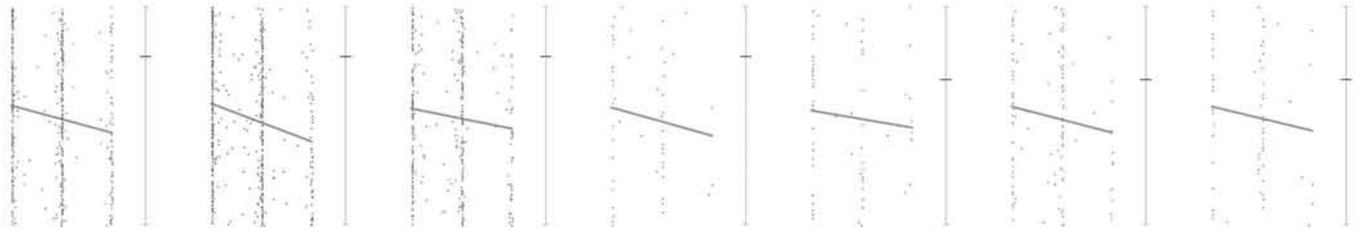

HT12-Controls
GG (153) GA (194) AA (67)
Corr: -0.151 **R2:** 0.023
Z-Score: -3.075 **P-Value:** 0.002

HT12-COPD
GG (202) GA (200) AA (51)
Corr: -0.196 **R2:** 0.038
Z-Score: -4.202 **P-Value:** 2.64E-5

HT12-ALS
GG (132) GA (148) AA (44)
Corr: -0.105 **R2:** 0.011
Z-Score: -1.885 **P-Value:** 0.059

HT12-UC
GG (24) GA (22) AA (3)
Corr: -0.126 **R2:** 0.016
Z-Score: -0.863 **P-Value:** 0.388

H8v2-Controls
GG (28) GA (21) AA (10)
Corr: -0.092 **R2:** 0.008
Z-Score: -0.692 **P-Value:** 0.489

H8v2-CeliacDisease
GG (41) GA (57) AA (13)
Corr: -0.128 **R2:** 0.016
Z-Score: -1.335 **P-Value:** 0.182

H8v2-ALS
GG (20) GA (32) AA (7)
Corr: -0.114 **R2:** 0.013
Z-Score: -0.861 **P-Value:** 0.389

SNP rs12485738 (chr. 3, 56840816 bp)
Probe 6770543 (chr. 16, 4947319 - 5010157 bp), SEC14L5
P-Value 1.19E-8

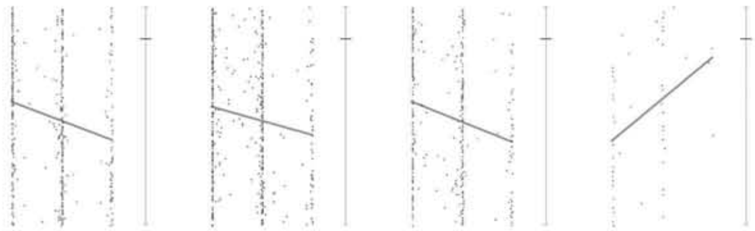

HT12-Controls
GG (153) GA (194) AA (67)
Corr: -0.207 **R2:** 0.043
Z-Score: -4.235 **P-Value:** 2.28E-5

HT12-COPD
GG (202) GA (200) AA (51)
Corr: -0.146 **R2:** 0.021
Z-Score: -3.118 **P-Value:** 0.002

HT12-ALS
GG (132) GA (148) AA (44)
Corr: -0.206 **R2:** 0.042
Z-Score: -3.725 **P-Value:** 1.95E-4

HT12-UC
GG (24) GA (22) AA (3)
Corr: 0.385 **R2:** 0.148
Z-Score: 2.734 **P-Value:** 0.006

H8v2-Controls
eQTL not available
Probe not present.

H8v2-CeliacDisease
eQTL not available
Probe not present.

H8v2-ALS
eQTL not available
Probe not present.

SNP rs12718597 (chr. 7, 50395922 bp)
Probe 7330379 (chr. 12, 6744802 - 6751379 bp), PTMS
P-Value 1.12E-8

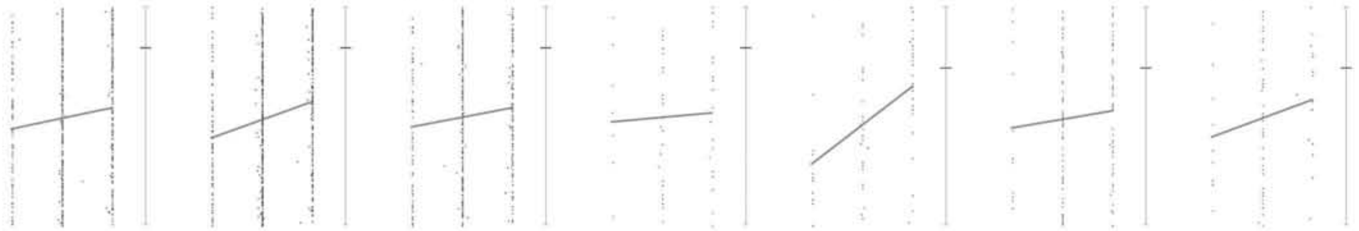

HT12-Controls
AA (64) AC (197) CC (153)
Corr: 0.111 **R2:** 0.012
Z-Score: 2.264 **P-Value:** 0.024

HT12-COPD
AA (68) AC (226) CC (159)
Corr: 0.197 **R2:** 0.039
Z-Score: 4.232 **P-Value:** 2.32E-5

HT12-ALS
AA (56) AC (163) CC (105)
Corr: 0.104 **R2:** 0.011
Z-Score: 1.864 **P-Value:** 0.062

HT12-UC
AA (7) AC (23) CC (19)
Corr: 0.046 **R2:** 0.002
Z-Score: 0.316 **P-Value:** 0.752

H8v2-Controls
AA (11) AC (23) CC (25)
Corr: 0.444 **R2:** 0.197
Z-Score: 3.525 **P-Value:** 4.24E-4

H8v2-CeliacDisease
AA (11) AC (53) CC (47)
Corr: 0.088 **R2:** 0.008
Z-Score: 0.919 **P-Value:** 0.358

H8v2-ALS
AA (9) AC (35) CC (15)
Corr: 0.18 **R2:** 0.032
Z-Score: 1.363 **P-Value:** 0.173

SNP rs2517713 (chr. 6, 30026078 bp)
Probe 7150170 (chr. 8, 6840527 - 6844510 bp), AF233439.5///DEFT1P///AF238378.4///DEFA3
P-Value 1.09E-8

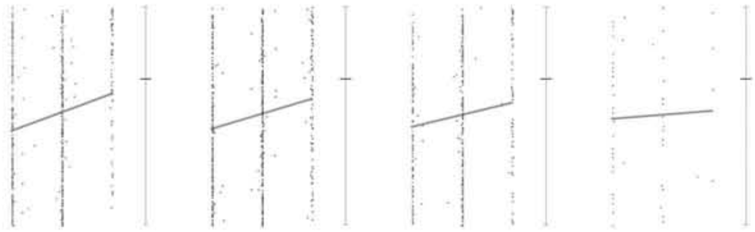

	HT12-Controls	HT12-COPD	HT12-ALS	HT12-UC	H8v2-Controls	H8v2-CeliacDisease	H8v2-ALS
Genotypes	TT (150) TG (205) GG (59)	TT (162) TG (209) GG (82)	TT (98) TG (170) GG (56)	TT (27) TG (17) GG (5)	eQTL not available	eQTL not available	eQTL not available
Corr:	0.197	0.166	0.129	0.039	Probe not present.	Probe not present.	Probe not present.
R2:	0.039	0.028	0.017	0.002			
Z-Score:	4.029	3.556	2.327	0.264			
P-Value:	5.6E-5	3.77E-4	0.02	0.791			

SNP rs2523393 (chr. 6, 29813638 bp)
Probe 7000274 (chr. 9, 118969171 - 118994970 bp), RPL10P3///SNORA70///ASTN2
P-Value 1.1E-8

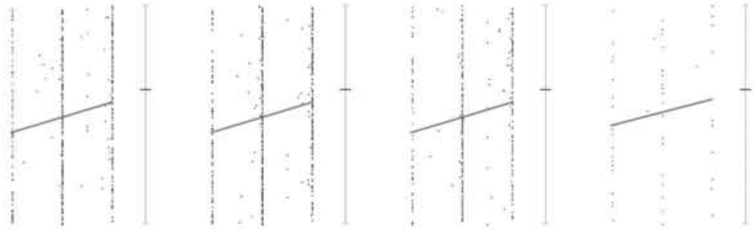

	HT12-Controls	HT12-COPD	HT12-ALS	HT12-UC	H8v2-Controls	H8v2-CeliacDisease	H8v2-ALS
Genotypes	GG (72) GA (196) AA (146)	GG (75) GA (221) AA (157)	GG (53) GA (158) AA (113)	GG (18) GA (20) AA (11)	eQTL not available	eQTL not available	eQTL not available
Corr:	0.164	0.161	0.162	0.151	Probe not present.	Probe not present.	Probe not present.
R2:	0.027	0.026	0.026	0.023			
Z-Score:	3.349	3.433	2.918	1.036			
P-Value:	8.12E-4	5.98E-4	0.004	0.3			

SNP rs2844479 (chr. 6, 31680935 bp)
Probe 1110300 (chr. 4, 153765727 - 153821641 bp), TMEM154
P-Value 1.09E-8

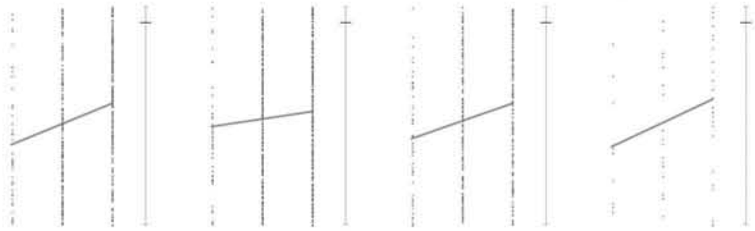

	HT12-Controls	HT12-COPD	HT12-ALS	HT12-UC	H8v2-Controls	H8v2-CeliacDisease	H8v2-ALS
Genotypes	CC (42) CA (175) AA (197)	CC (39) CA (203) AA (211)	CC (48) CA (139) AA (137)	CC (9) CA (17) AA (23)	eQTL not available	eQTL not available	eQTL not available
Corr:	0.215	0.076	0.195	0.279	Probe not present.	Probe not present.	Probe not present.
R2:	0.046	0.006	0.038	0.078			
Z-Score:	4.405	1.625	3.53	1.939			
P-Value:	1.06E-5	0.104	4.15E-4	0.052			

SNP rs9402686 (chr. 6, 135469510 bp)
Probe 6960615 (chr. 16, 1932977 - 1945672 bp), RPL3L
P-Value 1.09E-8

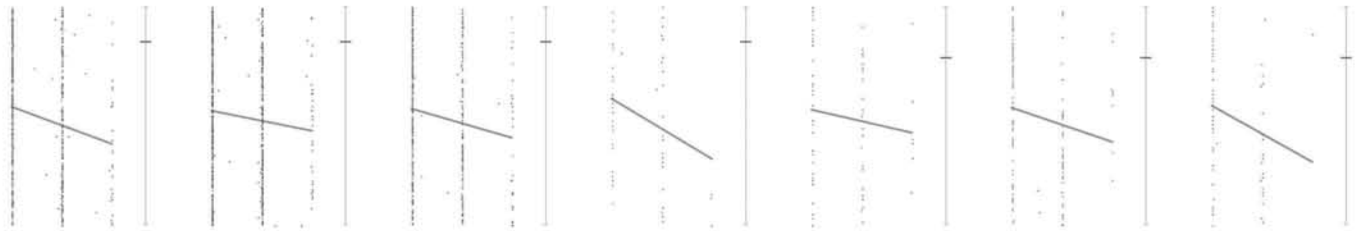

	HT12-Controls	HT12-COPD	HT12-ALS	HT12-UC	H8v2-Controls	H8v2-CeliacDisease	H8v2-ALS
Genotypes	GG (218) GA (169) AA (27)	GG (235) GA (188) AA (30)	GG (179) GA (117) AA (28)	GG (24) GA (22) AA (3)	GG (30) GA (23) AA (6)	GG (62) GA (40) AA (9)	GG (38) GA (20) AA (1)
Corr:	-0.179	-0.095	-0.151	-0.279	-0.118	-0.172	-0.225
R2:	0.032	0.009	0.023	0.078	0.014	0.029	0.051
Z-Score:	-3.664	-2.012	-2.715	-1.938	-0.893	-1.801	-1.713
P-Value:	2.48E-4	0.044	0.007	0.053	0.372	0.072	0.087

SNP rs1728785 (chr. 16, 67148731 bp)
Probe 7650747 (chr. 1, 159283573 - 159285545 bp), ARHGAP30
P-Value 1E-8

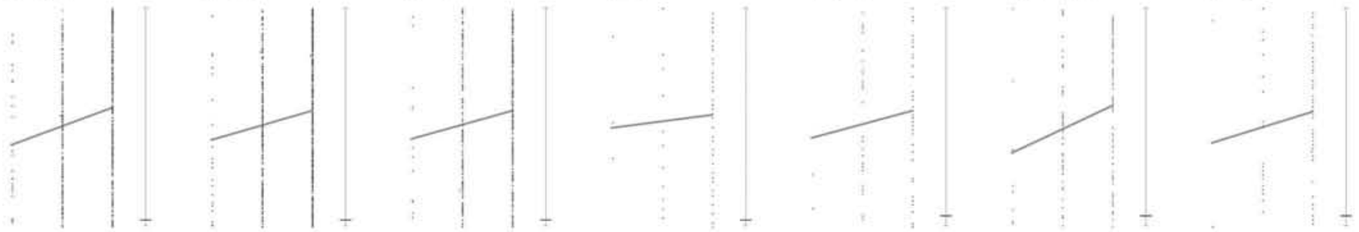

	HT12-Controls	HT12-COPD	HT12-ALS	HT12-UC	H8v2-Controls	H8v2-CeliacDisease	H8v2-ALS
Genotypes	AA (24) AC (154) CC (236)	AA (20) AC (167) CC (266)	AA (12) AC (130) CC (182)	AA (3) AC (11) CC (35)	AA (2) AC (24) CC (33)	AA (8) AC (40) CC (63)	AA (2) AC (18) CC (39)
Corr:	0.177	0.135	0.127	0.061	0.118	0.233	0.136
R2:	0.031	0.018	0.016	0.004	0.014	0.054	0.019
Z-Score:	3.626	2.886	2.294	0.417	0.893	2.46	1.028
P-Value:	2.88E-4	0.004	0.022	0.677	0.372	0.014	0.304

SNP rs4895441 (chr. 6, 135468266 bp)
Probe 6960615 (chr. 16, 1932977 - 1945672 bp), RPL3L
P-Value 9.91E-9

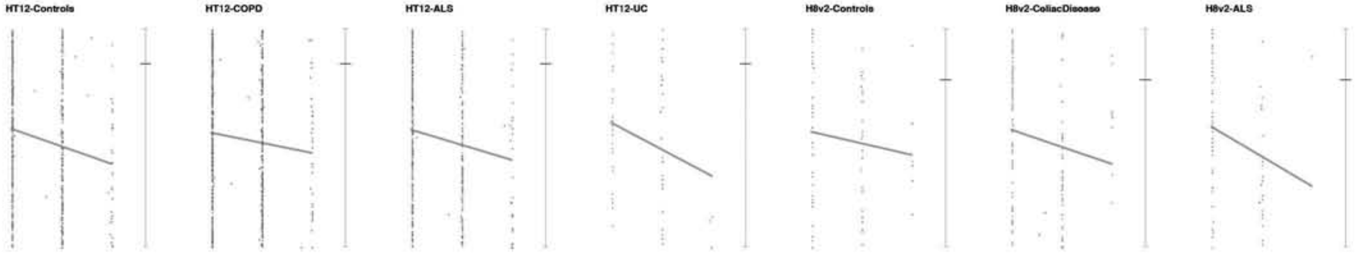

	HT12-Controls	HT12-COPD	HT12-ALS	HT12-UC	H8v2-Controls	H8v2-CeliacDisease	H8v2-ALS
Genotypes	AA (217) AG (167) GG (30)	AA (235) AG (167) GG (31)	AA (178) AG (118) GG (28)	AA (23) AG (23) GG (3)	AA (30) AG (23) GG (6)	AA (62) AG (40) GG (9)	AA (37) AG (21) GG (1)
Corr:	-0.174	-0.1	-0.154	-0.249	-0.119	-0.173	-0.238
R2:	0.03	0.01	0.024	0.062	0.014	0.03	0.057
Z-Score:	-3.563	-2.129	-2.769	-1.725	-0.9	-1.817	-1.817
P-Value:	3.67E-4	0.033	0.006	0.084	0.368	0.069	0.069

SNP rs9376092 (chr. 6, 135468837 bp)
Probe 6960615 (chr. 16, 1932977 - 1945672 bp), RPL3L
P-Value 9.94E-9

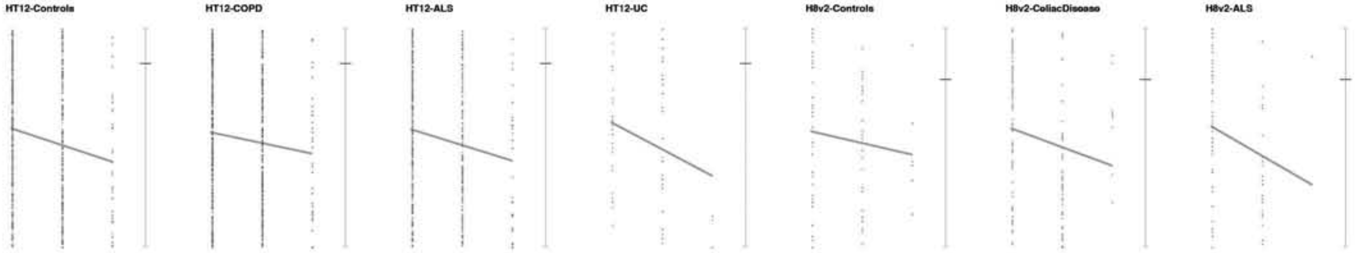

	HT12-Controls	HT12-COPD	HT12-ALS	HT12-UC	H8v2-Controls	H8v2-CeliacDisease	H8v2-ALS
Genotypes	CC (216) CA (167) AA (31)	CC (233) CA (189) AA (31)	CC (178) CA (118) AA (28)	CC (23) CA (23) AA (3)	CC (30) CA (23) AA (6)	CC (62) CA (40) AA (9)	CC (37) CA (21) AA (1)
Corr:	-0.165	-0.104	-0.155	-0.249	-0.119	-0.188	-0.237
R2:	0.027	0.011	0.024	0.062	0.014	0.035	0.056
Z-Score:	-3.365	-2.206	-2.791	-1.724	-0.901	-1.971	-1.805
P-Value:	7.66E-4	0.027	0.005	0.085	0.368	0.049	0.071

SNP rs9373124 (chr. 6, 135464902 bp)
Probe 6960615 (chr. 16, 1932977 - 1945672 bp), RPL3L
P-Value 9.56E-8

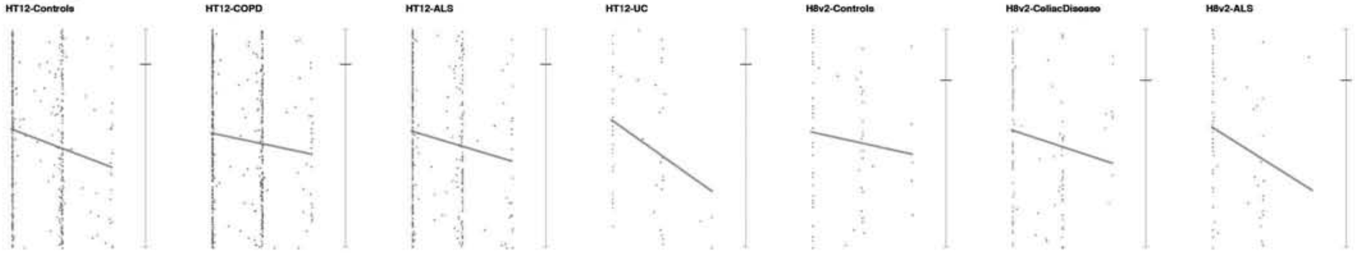

	HT12-Controls	HT12-COPD	HT12-ALS	HT12-UC	H8v2-Controls	H8v2-CeliacDisease	H8v2-ALS
Genotypes	TT (220) TC (169) CC (25)	TT (236) TC (188) CC (29)	TT (182) TC (116) CC (26)	TT (24) TC (22) CC (3)	TT (30) TC (24) CC (5)	TT (63) TC (39) CC (9)	TT (38) TC (20) CC (1)
Corr:	-0.179	-0.097	-0.149	-0.317	-0.11	-0.158	-0.236
R2:	0.032	0.009	0.022	0.101	0.012	0.025	0.056
Z-Score:	-3.656	-2.056	-2.689	-2.222	-0.826	-1.659	-1.798
P-Value:	2.57E-4	0.04	0.007	0.026	0.409	0.097	0.072

SNP rs11171739 (chr. 12, 54756892 bp)
Probe 3310592 (chr. 7, 98917628 - 98936260 bp), ZNF789///ZNF394
P-Value 9.22E-9

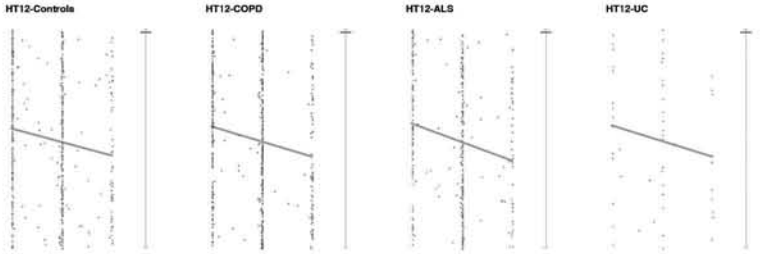

	HT12-Controls	HT12-COPD	HT12-ALS	HT12-UC	H8v2-Controls	H8v2-CeliacDisease	H8v2-ALS
Genotypes	TT (158) TC (196) CC (60)	TT (153) TC (225) CC (75)	TT (102) TC (174) CC (48)	TT (16) TC (24) CC (9)	eQTL not available	eQTL not available	eQTL not available
Corr:	-0.144	-0.159	-0.191	-0.167	Probe not present.	Probe not present.	Probe not present.
R2:	0.021	0.025	0.036	0.028			
Z-Score:	-2.928	-3.402	-3.454	-1.149			
P-Value:	0.003	6.69E-4	5.53E-4	0.25			

SNP rs602875 (chr. 6, 32681607 bp)
Probe 2940424 (chr. 7, 36518135 - 36731595 bp), AOAH
P-Value 8.85E-9

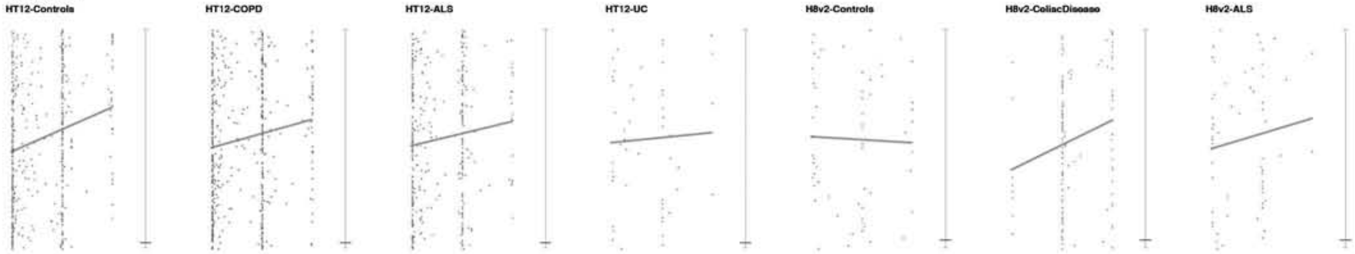

	HT12-Controls	HT12-COPD	HT12-ALS	HT12-UC	H8v2-Controls	H8v2-CeliacDisease	H8v2-ALS
Genotypes	AA (221) AG (165) GG (28)	AA (228) AG (179) GG (46)	AA (183) AG (121) GG (20)	AA (23) AG (22) GG (4)	AA (23) AG (26) GG (10)	AA (8) AG (69) GG (34)	AA (27) AG (30) GG (2)
Corr:	0.208	0.144	0.112	0.046	-0.028	0.217	0.126
R2:	0.043	0.021	0.013	0.002	0.001	0.047	0.016
Z-Score:	4.268	3.077	2.017	0.317	-0.211	2.287	0.949
P-Value:	1.97E-5	0.002	0.044	0.751	0.833	0.022	0.343

SNP rs615672 (chr. 6, 32682149 bp)
Probe 2940424 (chr. 7, 36518135 - 36731595 bp), AOAH
P-Value 8.95E-9

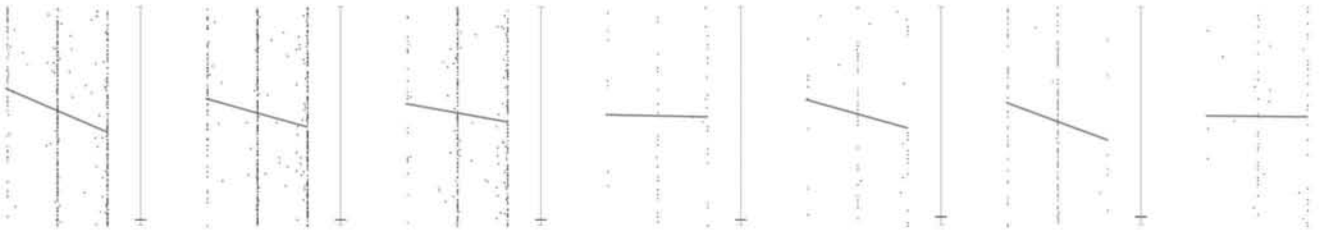

HT12-Controls
CC (56) CG (184) GG (174)
Corr: -0.232 **R2:** 0.054
Z-Score:-4.771 **P-Value:**1.83E-6

HT12-COPD
CC (66) CG (196) GG (191)
Corr: -0.15 **R2:** 0.023
Z-Score:-3.208 **P-Value:**0.001

HT12-ALS
CC (23) CG (159) GG (142)
Corr: -0.088 **R2:** 0.008
Z-Score:-1.579 **P-Value:**0.114

HT12-UC
CC (7) CG (21) GG (21)
Corr: -0.01 **R2:** 0
Z-Score:-0.071 **P-Value:**0.944

H8v2-Controls
CC (9) CG (30) GG (20)
Corr: -0.142 **R2:** 0.02
Z-Score:-1.073 **P-Value:**0.283

H8v2-CeliacDisease
CC (35) CG (69) GG (7)
Corr: -0.16 **R2:** 0.026
Z-Score:-1.683 **P-Value:**0.092

H8v2-ALS
CC (4) CG (30) GG (25)
Corr: -0.005 **R2:** 0
Z-Score:-0.034 **P-Value:**0.973

SNP rs12444268 (chr. 16, 20250073 bp)
Probe 1820086 (chr. 23, 144589943 - 144589943 bp), -
P-Value 8.87E-9

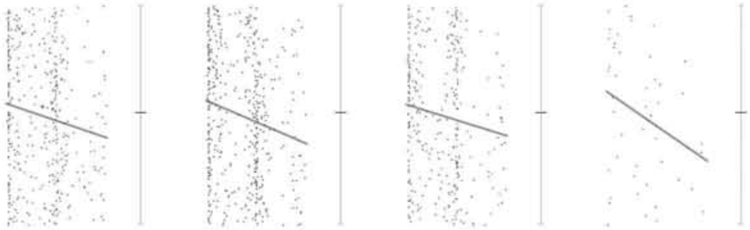

HT12-Controls
TT (175) TA (200) AA (39)
Corr: -0.149 **R2:** 0.022
Z-Score:-3.04 **P-Value:**0.002

HT12-COPD
TT (196) TA (216) AA (41)
Corr: -0.182 **R2:** 0.033
Z-Score:-3.9 **P-Value:**9.64E-5

HT12-ALS
TT (139) TA (157) AA (28)
Corr: -0.132 **R2:** 0.017
Z-Score:-2.369 **P-Value:**0.018

HT12-UC
TT (21) TA (21) AA (7)
Corr: -0.306 **R2:** 0.093
Z-Score:-2.136 **P-Value:**0.033

H8v2-Controls
eQTL not available
Probe not present.

H8v2-CeliacDisease
eQTL not available
Probe not present.

H8v2-ALS
eQTL not available
Probe not present.

SNP rs2517713 (chr. 6, 30026078 bp)
Probe 3290315 (chr. 1, 52026472 - 52117974 bp), NRD1
P-Value 8.32E-9

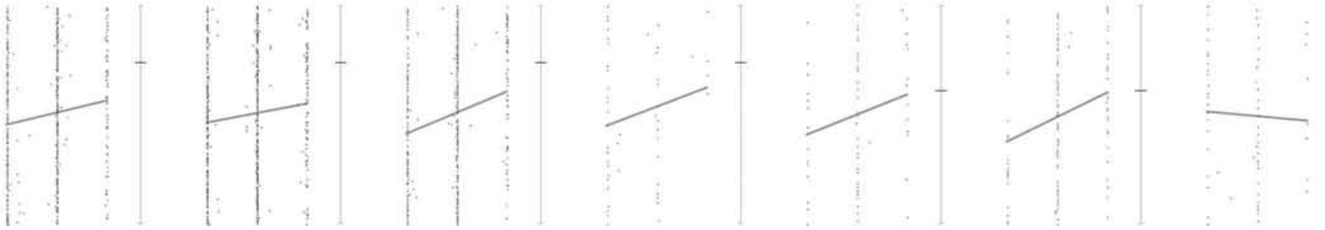

HT12-Controls
TT (150) TG (205) GG (59)
Corr: 0.125 **R2:** 0.016
Z-Score:2.543 **P-Value:**0.011

HT12-COPD
TT (162) TG (209) GG (82)
Corr: 0.108 **R2:** 0.012
Z-Score:2.29 **P-Value:**0.022

HT12-ALS
TT (98) TG (170) GG (56)
Corr: 0.223 **R2:** 0.05
Z-Score:4.052 **P-Value:**5.09E-5

HT12-UC
TT (27) TG (17) GG (5)
Corr: 0.19 **R2:** 0.036
Z-Score:1.306 **P-Value:**0.191

H8v2-Controls
TT (14) TG (32) GG (13)
Corr: 0.212 **R2:** 0.045
Z-Score:1.612 **P-Value:**0.107

H8v2-CeliacDisease
TT (20) TG (62) GG (29)
Corr: 0.256 **R2:** 0.066
Z-Score:2.711 **P-Value:**0.007

H8v2-ALS
TT (23) TG (28) GG (8)
Corr: -0.05 **R2:** 0.002
Z-Score:-0.372 **P-Value:**0.71

SNP rs1701704 (chr. 12, 54698754 bp)
Probe 3450750 (chr. 19, 62729505 - 62744936 bp), ZNF549
P-Value 8.11E-9

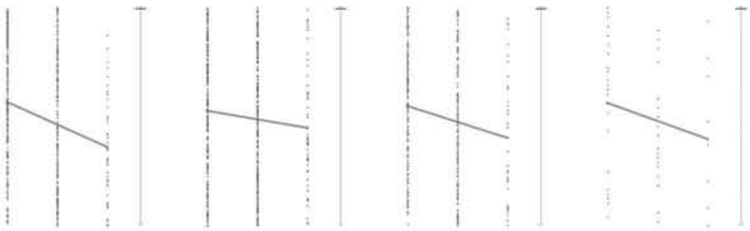

HT12-Controls
TT (193) TG (178) GG (43)
Corr: -0.232 **R2:** 0.054
Z-Score:-4.776 **P-Value:**1.79E-6

HT12-COPD
TT (196) TG (206) GG (51)
Corr: -0.092 **R2:** 0.008
Z-Score:-1.95 **P-Value:**0.051

HT12-ALS
TT (145) TG (146) GG (33)
Corr: -0.167 **R2:** 0.028
Z-Score:-3.019 **P-Value:**0.003

HT12-UC
TT (22) TG (18) GG (9)
Corr: -0.208 **R2:** 0.043
Z-Score:-1.433 **P-Value:**0.152

H8v2-Controls
eQTL not available
Probe not present.

H8v2-CeliacDisease
eQTL not available
Probe not present.

H8v2-ALS
eQTL not available
Probe not present.

SNP rs9268877 (chr. 6, 32539125 bp)
Probe 2120746 (chr. 7, 142003303 - 142005921 bp), ENST00000390392
P-Value 7.72E-9

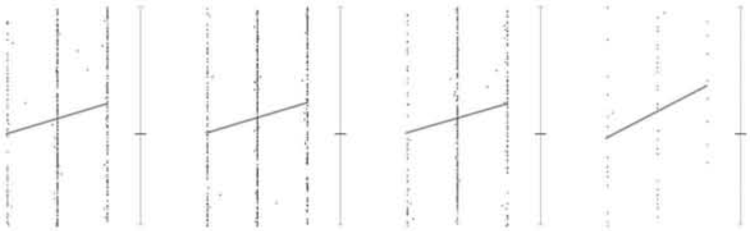

HT12-Controls
AA (61) AG (212) GG (141)
Corr: 0.158 **R2:** 0.025
Z-Score:3.234 **P-Value:**0.001

HT12-COPD
AA (91) AG (214) GG (148)
Corr: 0.169 **R2:** 0.028
Z-Score:3.607 **P-Value:**3.1E-4

HT12-ALS
AA (50) AG (179) GG (95)
Corr: 0.146 **R2:** 0.021
Z-Score:2.64 **P-Value:**0.008

HT12-UC
AA (16) AG (25) GG (8)
Corr: 0.276 **R2:** 0.076
Z-Score:1.917 **P-Value:**0.055

H8v2-Controls
eQTL not available
Probe not present.

H8v2-CeliacDisease
eQTL not available
Probe not present.

H8v2-ALS
eQTL not available
Probe not present.

SNP rs12485738 (chr. 3, 56840816 bp)
Probe 620750 (chr. 7, 134113711 - 134307010 bp), CALD1
P-Value 7.46E-9

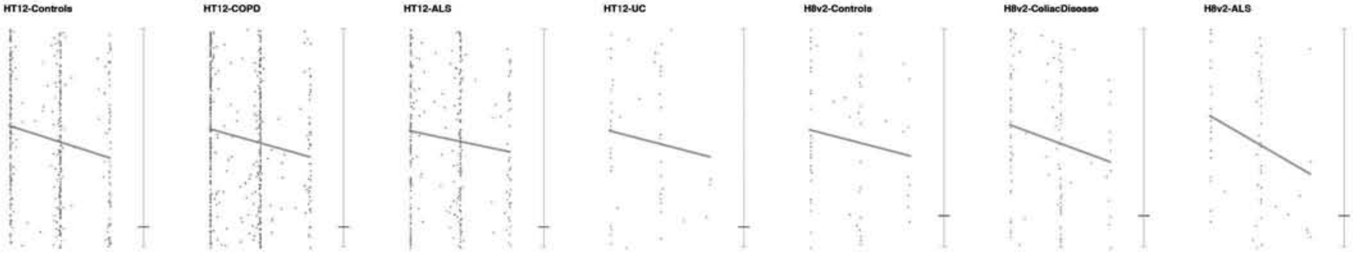

	HT12-Controls	HT12-COPD	HT12-ALS	HT12-UC	H8v2-Controls	H8v2-CeliacDisease	H8v2-ALS
Genotypes	GG (153) GA (194) AA (67)	GG (202) GA (200) AA (51)	GG (132) GA (148) AA (44)	GG (24) GA (22) AA (3)	GG (28) GA (21) AA (10)	GG (41) GA (57) AA (13)	GG (20) GA (32) AA (7)
Corr:	-0.171	-0.144	-0.106	-0.119	-0.144	-0.184	-0.277
R2:	0.029	0.021	0.011	0.014	0.021	0.034	0.077
Z-Score:	-3.493	-3.08	-1.912	-0.816	-1.089	-1.93	-2.127
P-Value:	4.77E-4	0.002	0.056	0.414	0.276	0.054	0.033

SNP rs7138803 (chr. 12, 48533735 bp)
Probe Human_RefSeq-8_v2-2260594 (chr. 4, 110955115 - 110966340 bp), GAR1
P-Value 6.75E-9

HT12-Controls
eQTL not available
Probe not present.

HT12-COPD
eQTL not available
Probe not present.

HT12-ALS
eQTL not available
Probe not present.

HT12-UC
eQTL not available
Probe not present.

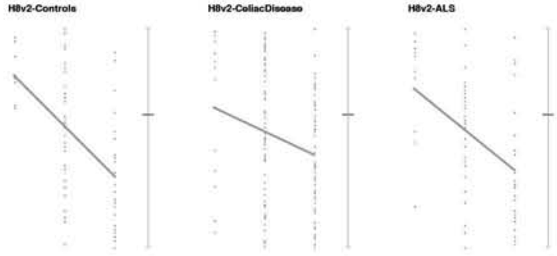

	H8v2-Controls	H8v2-CeliacDisease	H8v2-ALS
Genotypes	AA (8) AG (28) GG (23)	AA (13) AG (51) GG (47)	AA (10) AG (26) GG (23)
Corr:	-0.526	-0.244	-0.453
R2:	0.277	0.06	0.205
Z-Score:	-4.282	-2.585	-3.599
P-Value:	1.85E-5	0.01	3.19E-4

SNP rs11171739 (chr. 12, 54756892 bp)
Probe 3940500 (chr. 2, 19413728 - 19422865 bp), OSR1
P-Value 6.25E-9

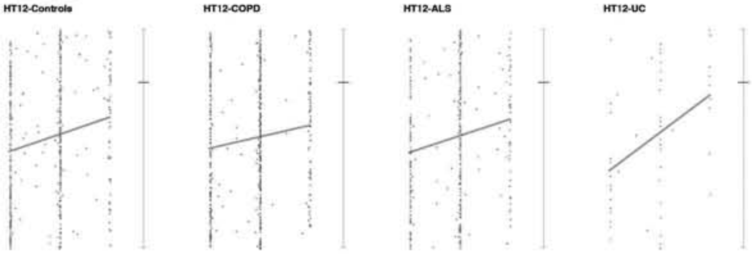

	HT12-Controls	HT12-COPD	HT12-ALS	HT12-UC
Genotypes	TT (158) TC (196) CC (60)	TT (153) TC (225) CC (75)	TT (102) TC (174) CC (48)	TT (16) TC (24) CC (9)
Corr:	0.179	0.12	0.171	0.403
R2:	0.032	0.014	0.029	0.162
Z-Score:	3.657	2.551	3.087	2.87
P-Value:	2.55E-4	0.011	0.002	0.004

H8v2-Controls
eQTL not available
Probe not present.

H8v2-CeliacDisease
eQTL not available
Probe not present.

H8v2-ALS
eQTL not available
Probe not present.

SNP rs12485738 (chr. 3, 56840816 bp)
Probe 2070369 (chr. 6, 41224061 - 41231053 bp), RP1-238O23.3
P-Value 6.19E-9

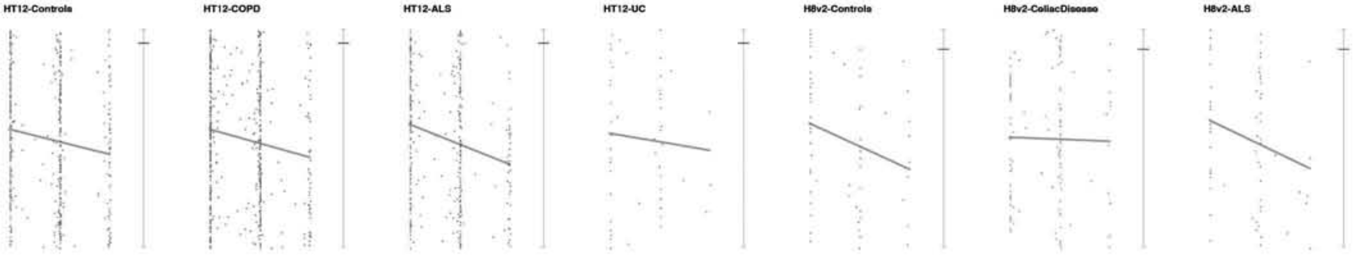

	HT12-Controls	HT12-COPD	HT12-ALS	HT12-UC	H8v2-Controls	H8v2-CeliacDisease	H8v2-ALS
Genotypes	GG (153) GA (194) AA (67)	GG (202) GA (200) AA (51)	GG (132) GA (148) AA (44)	GG (24) GA (22) AA (3)	GG (28) GA (21) AA (10)	GG (41) GA (57) AA (13)	GG (20) GA (32) AA (7)
Corr:	-0.137	-0.142	-0.206	-0.078	-0.256	-0.019	-0.229
R2:	0.019	0.02	0.042	0.006	0.066	0	0.052
Z-Score:	-2.794	-3.036	-3.734	-0.533	-1.961	-0.194	-1.745
P-Value:	0.005	0.002	1.89E-4	0.594	0.05	0.846	0.081

SNP rs11602954 (chr. 11, 192856 bp)
Probe 2230241 (chr. 6, 6068310 - 6266923 bp), F13A1
P-Value 5.68E-9

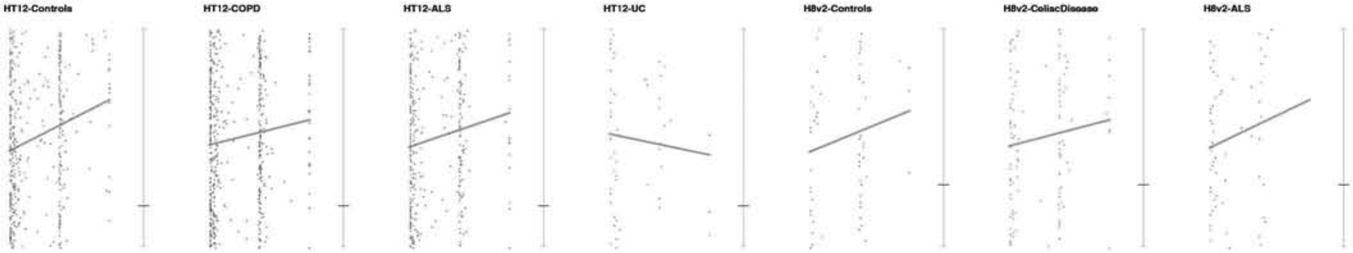

	HT12-Controls	HT12-COPD	HT12-ALS	HT12-UC	H8v2-Controls	H8v2-CeliacDisease	H8v2-ALS
Genotypes	GG (248) GA (143) AA (23)	GG (261) GA (165) AA (27)	GG (190) GA (116) AA (18)	GG (31) GA (15) AA (3)	GG (26) GA (29) AA (4)	GG (56) GA (47) AA (8)	GG (40) GA (19) AA (0)
Corr:	0.219	0.117	0.153	-0.095	0.183	0.123	0.162
R2:	0.048	0.014	0.024	0.009	0.033	0.015	0.026
Z-Score:	4.489	2.488	2.768	-0.65	1.385	1.282	1.226
P-Value:	7.16E-6	0.013	0.006	0.516	0.166	0.2	0.22

SNP rs643381 (chr. 6, 139881116 bp)
Probe 270133 (chr. 1, 23976470 - 23988309 bp), C1orf128
P-Value 4.9E-9

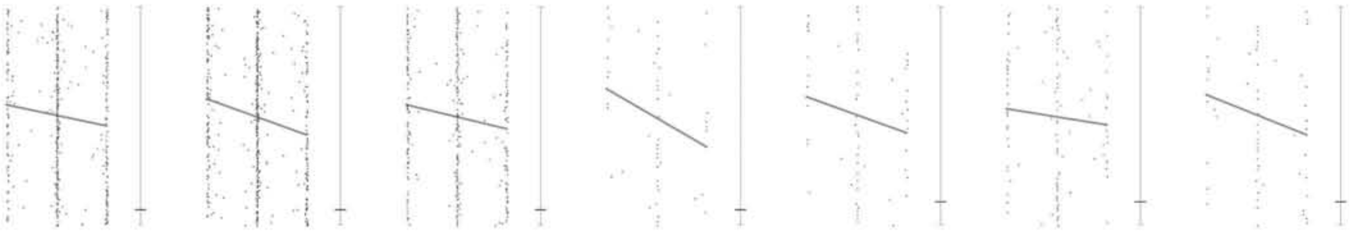

	HT12-Controls	HT12-COPD	HT12-ALS	HT12-UC	H8v2-Controls	H8v2-CeliacDisease	H8v2-ALS
Genotypes	AA (82) AC (216) CC (116)	AA (116) AC (236) CC (101)	AA (95) AC (149) CC (80)	AA (11) AC (29) CC (9)	AA (11) AC (31) CC (17)	AA (30) AC (57) CC (24)	AA (14) AC (28) CC (17)
Corr:	-0.112	-0.191	-0.139	-0.274	-0.188	-0.08	-0.216
R2:	0.012	0.037	0.019	0.075	0.036	0.006	0.047
Z-Score:	-2.274	-4.099	-2.501	-1.908	-1.429	-0.831	-1.645
P-Value:	0.023	4.16E-5	0.012	0.056	0.153	0.406	0.1

SNP rs7647305 (chr. 3, 187316984 bp)
Probe 540184 (chr. 4, 181855820 - 181855820 bp), -
P-Value 4.85E-9

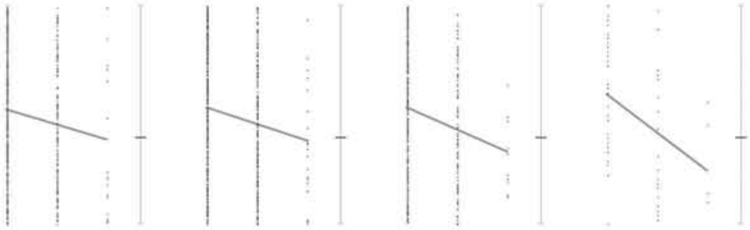

	HT12-Controls	HT12-COPD	HT12-ALS	HT12-UC	H8v2-Controls	H8v2-CeliacDisease	H8v2-ALS
Genotypes	CC (266) CT (132) TT (16)	CC (260) CT (173) TT (20)	CC (218) CT (96) TT (10)	CC (27) CT (18) TT (4)	eQTL not available	eQTL not available	eQTL not available
Corr:	-0.134	-0.155	-0.188	-0.38	Probe not present.	Probe not present.	Probe not present.
R2:	0.018	0.024	0.035	0.144			
Z-Score:	-2.725	-3.318	-3.402	-2.692			
P-Value:	0.006	9.06E-4	6.69E-4	0.007			

SNP rs628751 (chr. 6, 139880112 bp)
Probe 270133 (chr. 1, 23976470 - 23988309 bp), C1orf128
P-Value 4.75E-9

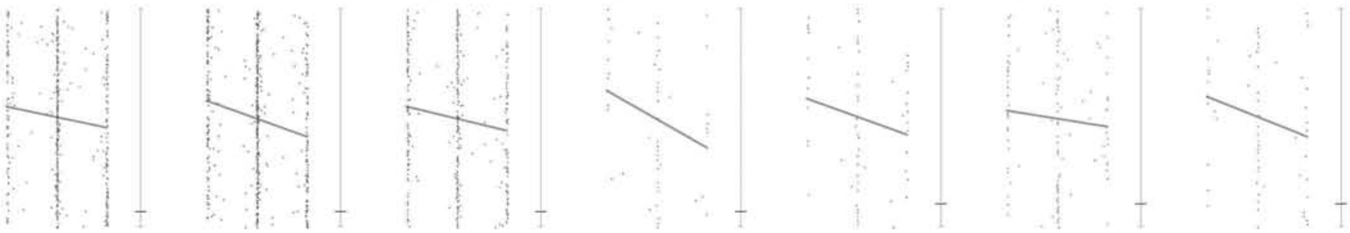

	HT12-Controls	HT12-COPD	HT12-ALS	HT12-UC	H8v2-Controls	H8v2-CeliacDisease	H8v2-ALS
Genotypes	AA (82) AC (216) CC (116)	AA (116) AC (236) CC (101)	AA (95) AC (149) CC (80)	AA (11) AC (29) CC (9)	AA (11) AC (31) CC (17)	AA (30) AC (57) CC (24)	AA (14) AC (28) CC (17)
Corr:	-0.112	-0.192	-0.138	-0.274	-0.188	-0.08	-0.217
R2:	0.013	0.037	0.019	0.075	0.035	0.006	0.047
Z-Score:	-2.28	-4.111	-2.494	-1.906	-1.426	-0.831	-1.648
P-Value:	0.023	3.95E-5	0.013	0.057	0.154	0.406	0.099

SNP rs12485738 (chr. 3, 56840816 bp)
Probe 4250753 (chr. 7, 128570946 - 128596907 bp), TSPAN33
P-Value 4.66E-9

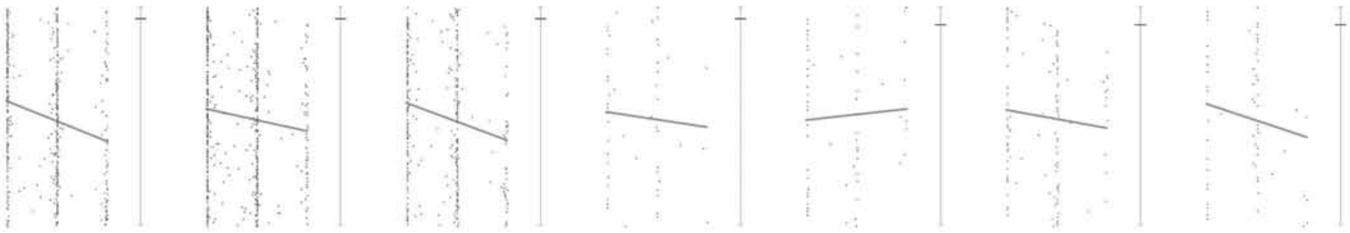

	HT12-Controls	HT12-COPD	HT12-ALS	HT12-UC	H8v2-Controls	H8v2-CeliacDisease	H8v2-ALS
Genotypes	GG (153) GA (194) AA (67)	GG (202) GA (200) AA (51)	GG (132) GA (148) AA (44)	GG (24) GA (22) AA (3)	GG (28) GA (21) AA (10)	GG (41) GA (57) AA (13)	GG (20) GA (32) AA (7)
Corr:	-0.219	-0.114	-0.192	-0.066	0.06	-0.089	-0.161
R2:	0.048	0.013	0.037	0.004	0.004	0.008	0.026
Z-Score:	-4.489	-2.425	-3.471	-0.452	0.45	-0.926	-1.215
P-Value:	7.14E-6	0.015	5.19E-4	0.651	0.653	0.354	0.224

SNP rs11171739 (chr. 12, 54756892 bp)
Probe 4760424 (chr. 19, 43800562 - 43820435 bp), EIF3K
P-Value 4.58E-9

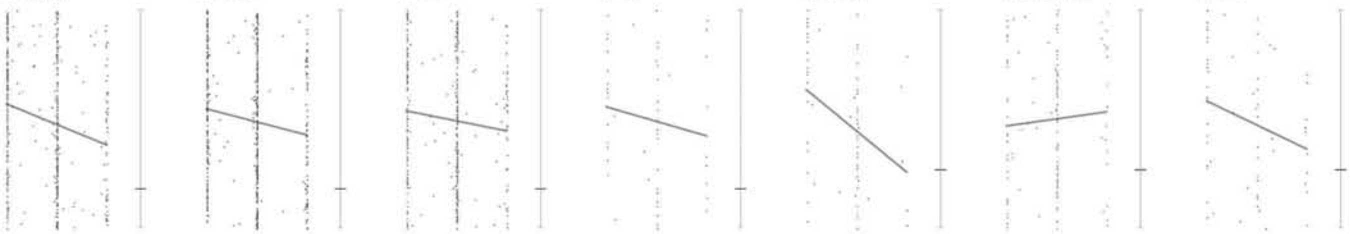

	HT12-Controls	HT12-COPD	HT12-ALS	HT12-UC	H8v2-Controls	H8v2-CeliacDisease	H8v2-ALS
Genotypes	TT (158) TC (196) CC (60)	TT (153) TC (225) CC (75)	TT (102) TC (174) CC (48)	TT (16) TC (24) CC (9)	TT (22) TC (32) CC (5)	TT (33) TC (59) CC (19)	TT (24) TC (25) CC (10)
Corr:	-0.216	-0.137	-0.105	-0.158	-0.379	0.071	-0.268
R2:	0.046	0.019	0.011	0.025	0.143	0.005	0.072
Z-Score:	-4.426	-2.919	-1.888	-1.085	-2.957	0.743	-2.048
P-Value:	9.61E-6	0.004	0.059	0.278	0.003	0.457	0.041

SNP rs2523393 (chr. 6, 29813638 bp)
Probe 4230441 (chr. 16, 56258658 - 56282476 bp), GPR97
P-Value 4.35E-9

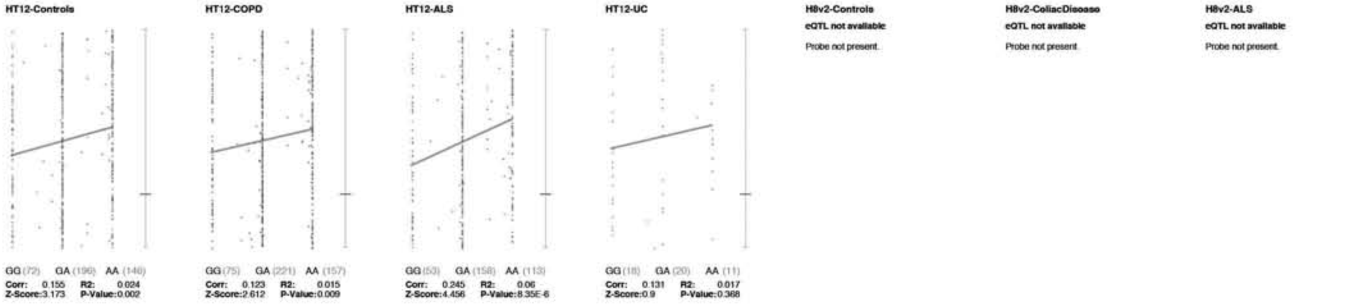

SNP rs2517713 (chr. 6, 30026078 bp)
Probe 6760209 (chr. 6, 136620419 - 136653682 bp), CTA-211L9.1
P-Value 4.27E-9

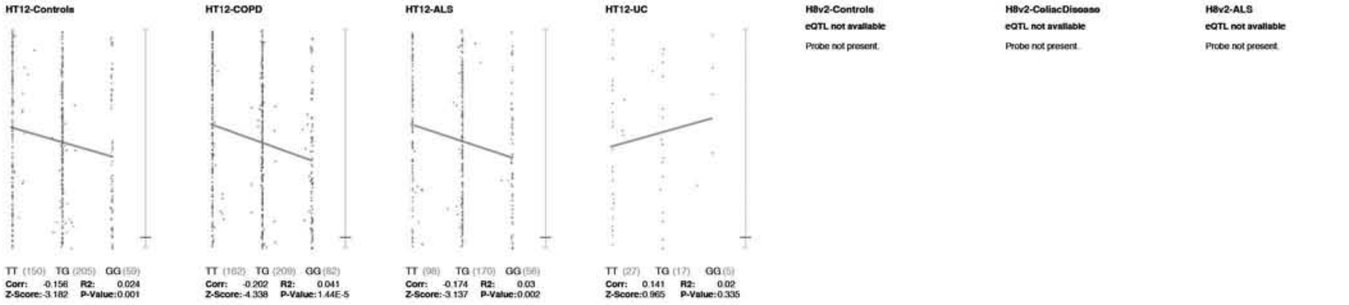

SNP rs2523393 (chr. 6, 29813638 bp)
Probe 5090044 (chr. 3, 101909850 - 101951500 bp), TFG
P-Value 4.29E-9

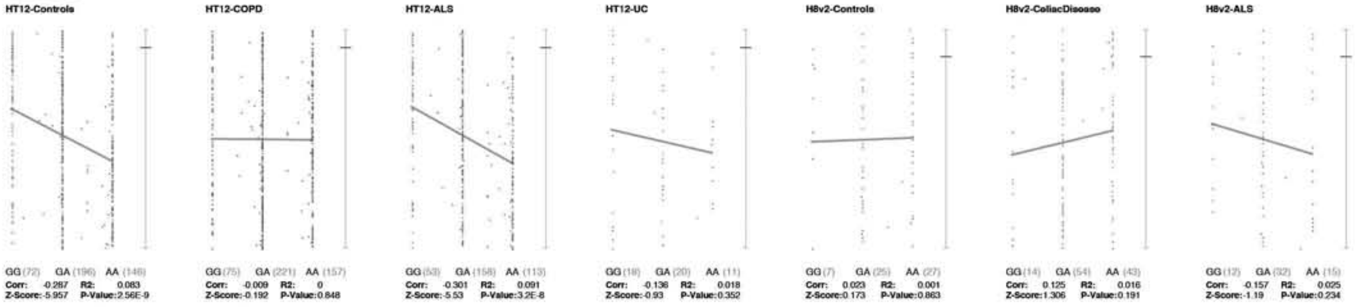

SNP rs1980493 (chr. 6, 32471193 bp)
Probe 6380347 (chr. 22, 40346241 - 40390998 bp), XRCC6
P-Value 3.67E-9

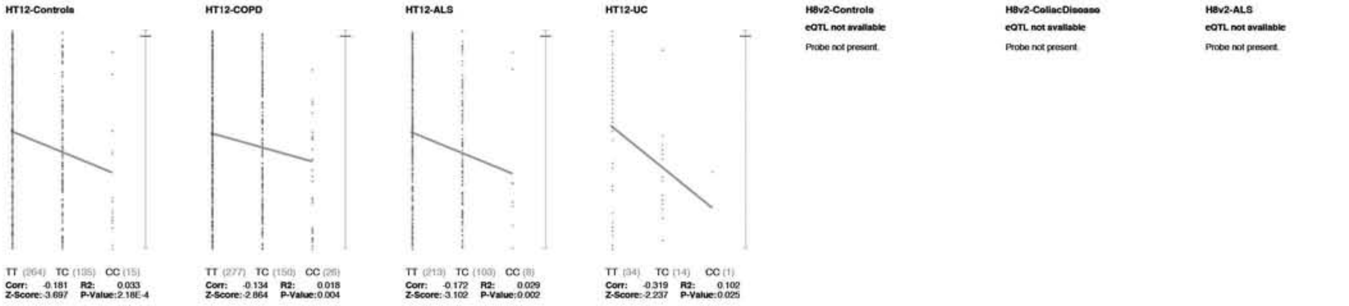

SNP rs628751 (chr. 6, 139880112 bp)
Probe 2450647 (chr. 12, 51353787 - 51361458 bp), KRT1
P-Value 3.09E-9

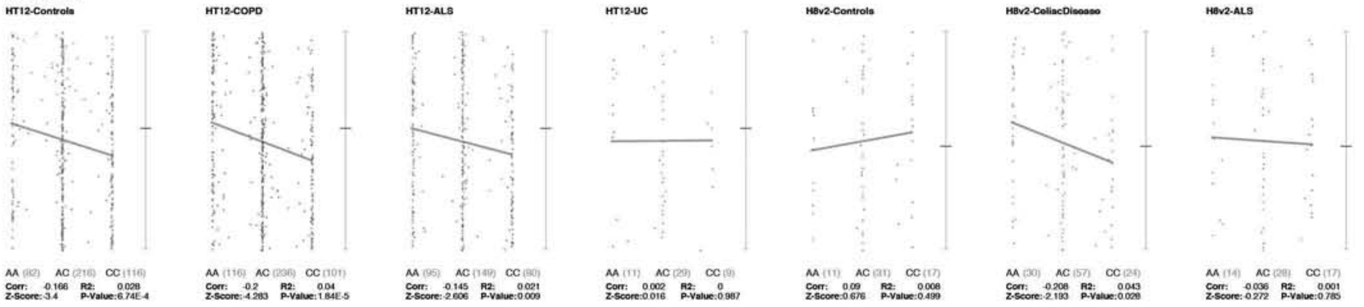

SNP rs643381 (chr. 6, 139881116 bp)
Probe 2450647 (chr. 12, 51353787 - 51361458 bp), KRT1
P-Value 3.03E-9

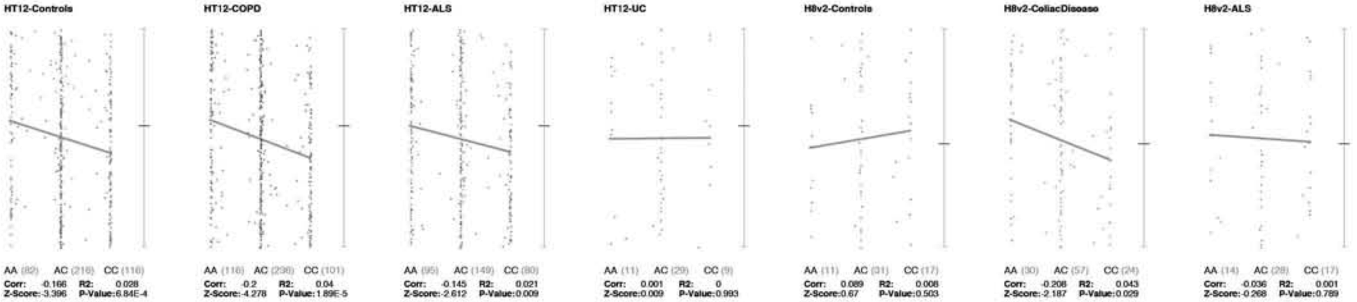

SNP rs628751 (chr. 6, 139880112 bp)
Probe 3170270 (chr. 1, 47420848 - 47430303 bp), PDZK1IP1
P-Value 2.86E-9

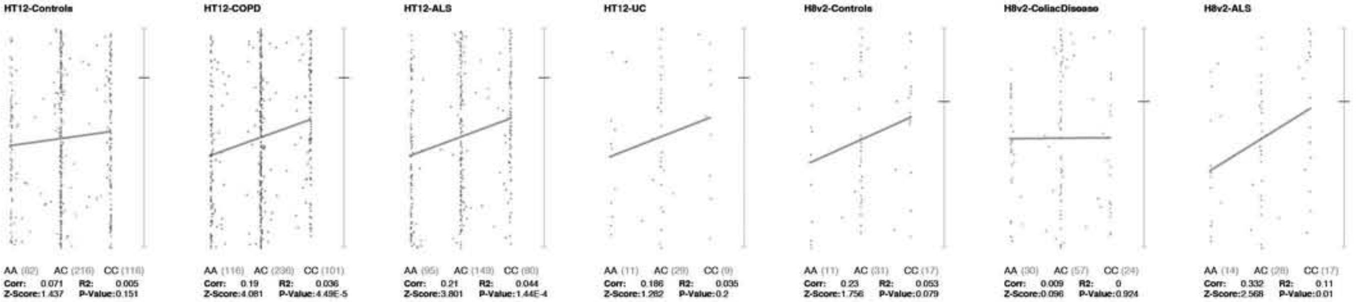

SNP rs2292239 (chr. 12, 54768447 bp)
Probe 2320494 (chr. 5, 177413996 - 177416873 bp), AC136632.3
P-Value 2.69E-9

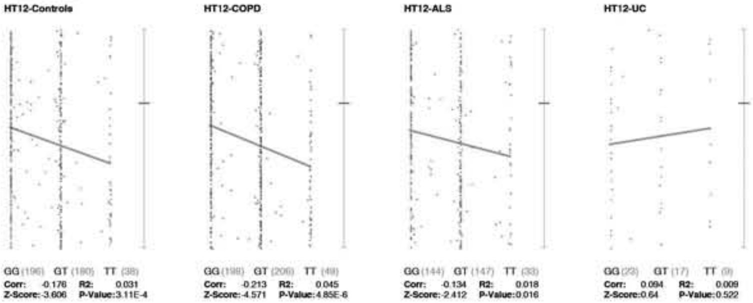

H8v2-Controls
eQTL not available
Probe not present.

H8v2-CeliacDisease
eQTL not available
Probe not present.

H8v2-ALS
eQTL not available
Probe not present.

SNP rs643381 (chr. 6, 139881116 bp)
Probe 3170270 (chr. 1, 47420848 - 47430303 bp), PDZK1IP1
P-Value 2.66E-9

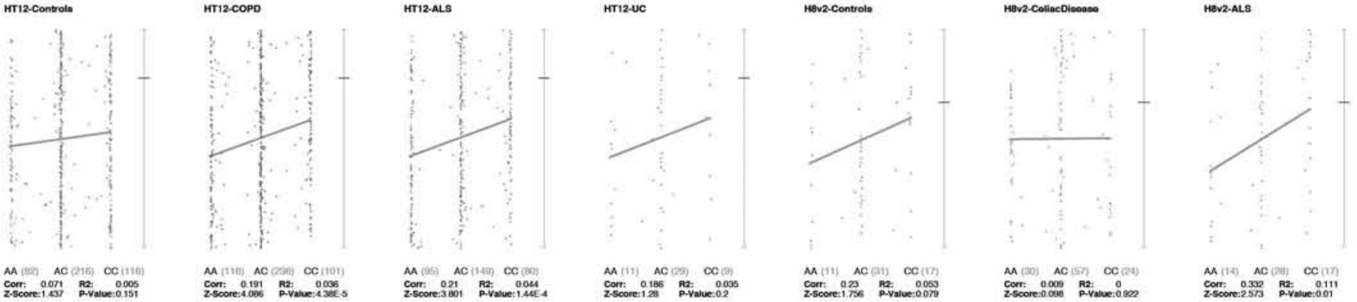

SNP rs8321 (chr. 6, 30140501 bp)
Probe 6380347 (chr. 22, 40346241 - 40390998 bp), XRCC6
P-Value 2.32E-9

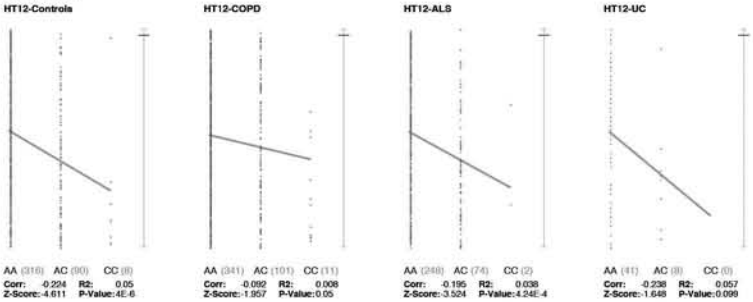

H8v2-Controls
eQTL not available
Probe not present.

H8v2-CeliacDisease
eQTL not available
Probe not present.

H8v2-ALS
eQTL not available
Probe not present.

SNP rs2517713 (chr. 6, 30026078 bp)
Probe 2970747 (chr. 8, 6839738 - 6844588 bp), AF233439.5///DEFT1P///AF238378.4///DEFA3
P-Value 1.98E-9

HT12-Controls | **HT12-COPD** | **HT12-ALS** | **HT12-UC** | **H8v2-Controls** | **H8v2-CeliacDisease** | **H8v2-ALS**

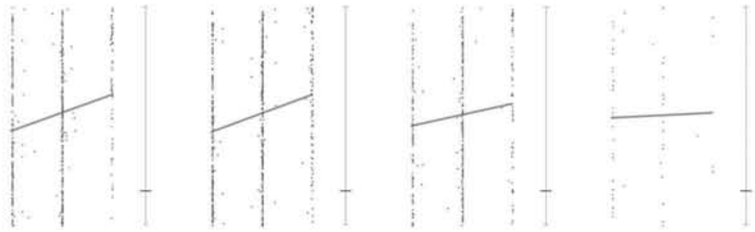

HT12-Controls: TT (150) TG (205) GG (59)
Corr: 0.189 **R2:** 0.036
Z-Score:3.868 **P-Value:**1.1E-4

HT12-COPD: TT (162) TG (209) GG (82)
Corr: 0.204 **R2:** 0.042
Z-Score:4.383 **P-Value:**1.17E-5

HT12-ALS: TT (98) TG (170) GG (56)
Corr: 0.118 **R2:** 0.014
Z-Score:2.125 **P-Value:**0.034

HT12-UC: TT (27) TG (17) GG (5)
Corr: 0.025 **R2:** 0.001
Z-Score:0.17 **P-Value:**0.865

H8v2-Controls: **eQTL not available** Probe not present.

H8v2-CeliacDisease: **eQTL not available** Probe not present.

H8v2-ALS: **eQTL not available** Probe not present.

SNP rs11171739 (chr. 12, 54756892 bp)
Probe 5960296 (chr. 3, 191505197 - 191523909 bp), CLDN1
P-Value 2E-9

HT12-Controls | **HT12-COPD** | **HT12-ALS** | **HT12-UC** | **H8v2-Controls** | **H8v2-CeliacDisease** | **H8v2-ALS**

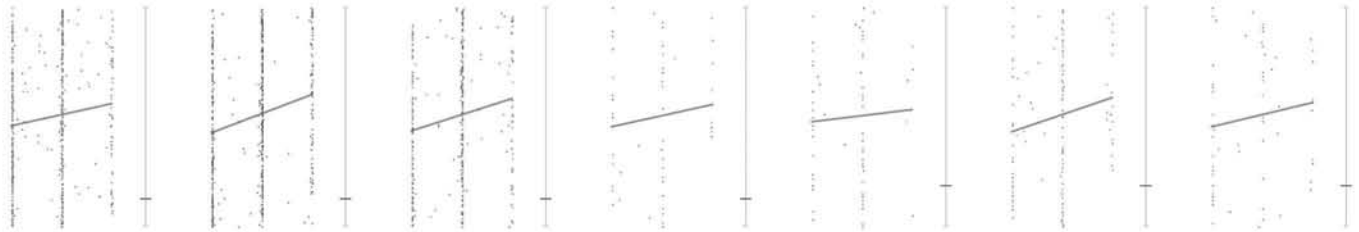

HT12-Controls: TT (158) TC (196) CC (60)
Corr: 0.114 **R2:** 0.013
Z-Score:2.327 **P-Value:**0.02

HT12-COPD: TT (153) TC (225) CC (75)
Corr: 0.203 **R2:** 0.041
Z-Score:4.357 **P-Value:**1.32E-5

HT12-ALS: TT (102) TC (174) CC (48)
Corr: 0.166 **R2:** 0.028
Z-Score:3.001 **P-Value:**0.003

HT12-UC: TT (16) TC (24) CC (9)
Corr: 0.115 **R2:** 0.013
Z-Score:0.788 **P-Value:**0.431

H8v2-Controls: TT (22) TC (32) CC (5)
Corr: 0.055 **R2:** 0.003
Z-Score:0.413 **P-Value:**0.68

H8v2-CeliacDisease: TT (33) TC (59) CC (19)
Corr: 0.175 **R2:** 0.031
Z-Score:1.839 **P-Value:**0.066

H8v2-ALS: TT (24) TC (25) CC (10)
Corr: 0.138 **R2:** 0.019
Z-Score:1.041 **P-Value:**0.298

SNP rs11171739 (chr. 12, 54756892 bp)
Probe 2320494 (chr. 5, 177413996 - 177416873 bp), AC136632.3
P-Value 1.95E-9

HT12-Controls | **HT12-COPD** | **HT12-ALS** | **HT12-UC** | **H8v2-Controls** | **H8v2-CeliacDisease** | **H8v2-ALS**

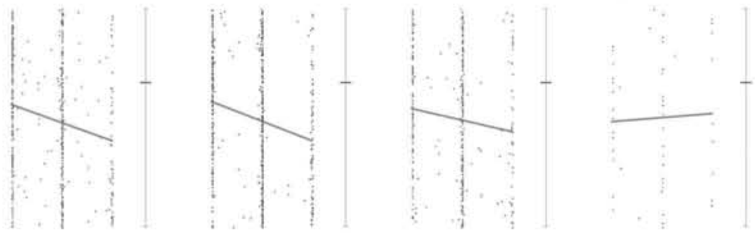

HT12-Controls: TT (158) TC (196) CC (60)
Corr: -0.194 **R2:** 0.038
Z-Score:-3.968 **P-Value:**7.25E-5

HT12-COPD: TT (153) TC (225) CC (75)
Corr: -0.209 **R2:** 0.043
Z-Score:-4.475 **P-Value:**7.65E-6

HT12-ALS: TT (102) TC (174) CC (48)
Corr: -0.114 **R2:** 0.013
Z-Score:-2.055 **P-Value:**0.04

HT12-UC: TT (16) TC (24) CC (9)
Corr: 0.039 **R2:** 0.002
Z-Score:0.268 **P-Value:**0.789

H8v2-Controls: **eQTL not available** Probe not present.

H8v2-CeliacDisease: **eQTL not available** Probe not present.

H8v2-ALS: **eQTL not available** Probe not present.

SNP rs12485738 (chr. 3, 56840816 bp)
Probe 2650114 (chr. 3, 125963488 - 126089834 bp), ITGB5
P-Value 1.89E-9

HT12-Controls | **HT12-COPD** | **HT12-ALS** | **HT12-UC** | **H8v2-Controls** | **H8v2-CeliacDisease** | **H8v2-ALS**

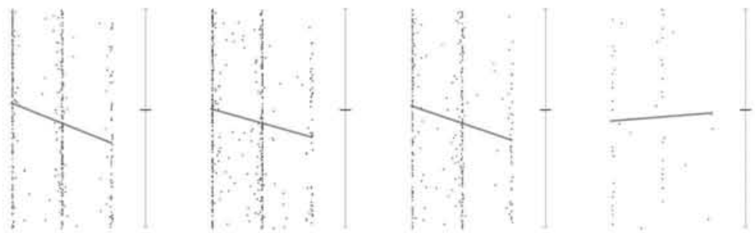

HT12-Controls: GG (153) GA (194) AA (67)
Corr: -0.22 **R2:** 0.048
Z-Score:-4.517 **P-Value:**6.27E-6

HT12-COPD: GG (202) GA (200) AA (51)
Corr: -0.141 **R2:** 0.02
Z-Score:-3.008 **P-Value:**0.003

HT12-ALS: GG (132) GA (148) AA (44)
Corr: -0.176 **R2:** 0.031
Z-Score:-3.173 **P-Value:**0.002

HT12-UC: GG (24) GA (22) AA (3)
Corr: 0.035 **R2:** 0.001
Z-Score:0.239 **P-Value:**0.811

H8v2-Controls: **eQTL not available** Probe not present.

H8v2-CeliacDisease: **eQTL not available** Probe not present.

H8v2-ALS: **eQTL not available** Probe not present.

SNP rs1701704 (chr. 12, 54698754 bp)
Probe 2320494 (chr. 5, 177413996 - 177416873 bp), AC136632.3
P-Value 1.83E-9

HT12-Controls | **HT12-COPD** | **HT12-ALS** | **HT12-UC** | **H8v2-Controls** | **H8v2-CeliacDisease** | **H8v2-ALS**

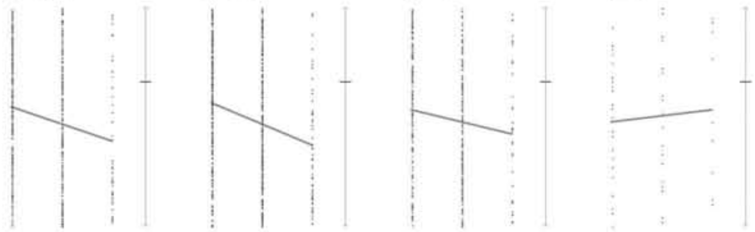

HT12-Controls: TT (193) TG (178) GG (43)
Corr: -0.18 **R2:** 0.032
Z-Score:-3.674 **P-Value:**2.39E-4

HT12-COPD: TT (196) TG (206) GG (51)
Corr: -0.218 **R2:** 0.048
Z-Score:-4.683 **P-Value:**2.83E-6

HT12-ALS: TT (145) TG (146) GG (33)
Corr: -0.125 **R2:** 0.016
Z-Score:-2.242 **P-Value:**0.025

HT12-UC: TT (22) TG (18) GG (9)
Corr: 0.071 **R2:** 0.005
Z-Score:0.486 **P-Value:**0.627

H8v2-Controls: **eQTL not available** Probe not present.

H8v2-CeliacDisease: **eQTL not available** Probe not present.

H8v2-ALS: **eQTL not available** Probe not present.

SNP rs4785763 (chr. 16, 88584437 bp)
Probe 4040541 (chr. 3, 115329247 - 115381589 bp), DRD3
P-Value 1.82E-9

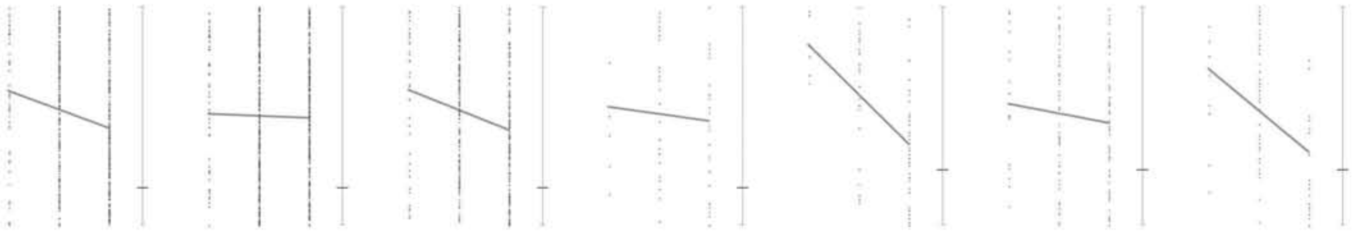

Dataset	Genotypes	Corr	R2	Z-Score	P-Value
HT12-Controls	AA (42) AC (174) CC (198)	-0.195	0.038	-3.997	6.41E-5
HT12-COPD	AA (45) AC (207) CC (201)	-0.017	0	-0.367	0.714
HT12-ALS	AA (35) AC (147) CC (142)	-0.211	0.044	-3.818	1.35E-4
HT12-UC	AA (5) AC (23) CC (21)	-0.071	0.005	-0.484	0.628
H8v2-Controls	AA (6) AC (21) CC (32)	-0.521	0.271	-4.229	2.35E-5
H8v2-CeliacDisease	AA (16) AC (43) CC (52)	-0.109	0.012	-1.138	0.255
H8v2-ALS	AA (8) AC (34) CC (17)	-0.41	0.169	-3.229	0.001

SNP rs12718597 (chr. 7, 50385922 bp)
Probe Human_RefSeq-8_v2-2340110 (chr. 2, 190709731 - 190777453 bp), AC010679.6
P-Value 1.74E-9

HT12-Controls: eQTL not available. Probe not present.

HT12-COPD: eQTL not available. Probe not present.

HT12-ALS: eQTL not available. Probe not present.

HT12-UC: eQTL not available. Probe not present.

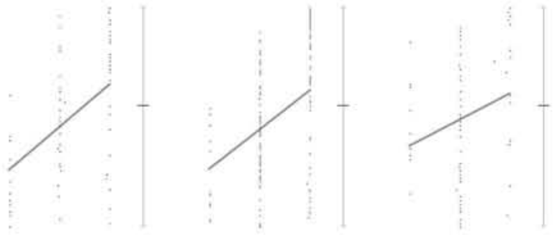

Dataset	Genotypes	Corr	R2	Z-Score	P-Value
H8v2-Controls	AA (11) AC (23) CC (25)	0.496	0.246	3.999	6.37E-5
H8v2-CeliacDisease	AA (11) AC (53) CC (47)	0.397	0.158	4.313	1.61E-5
H8v2-ALS	AA (9) AC (35) CC (15)	0.255	0.065	1.95	0.051

SNP rs2517713 (chr. 6, 30026078 bp)
Probe 3170403 (chr. 6, 118911755 - 118919501 bp), C6orf204
P-Value 1.68E-9

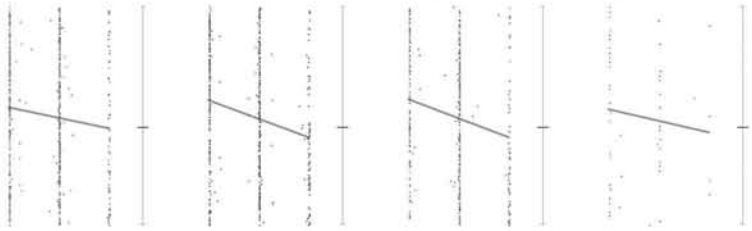

Dataset	Genotypes	Corr	R2	Z-Score	P-Value
HT12-Controls	TT (150) TG (205) GG (59)	-0.109	0.012	-2.218	0.027
HT12-COPD	TT (162) TG (209) GG (82)	-0.206	0.042	-4.42	9.88E-6
HT12-ALS	TT (98) TG (170) GG (56)	-0.207	0.043	-3.744	1.81E-4
HT12-UC	TT (27) TG (17) GG (5)	-0.114	0.013	-0.777	0.437

H8v2-Controls: eQTL not available. Probe not present.

H8v2-CeliacDisease: eQTL not available. Probe not present.

H8v2-ALS: eQTL not available. Probe not present.

SNP rs12485738 (chr. 3, 56840816 bp)
Probe 6860164 (chr. 12, 10030319 - 10051641 bp), CLEC1B///CLEC12B
P-Value 1.6E-9

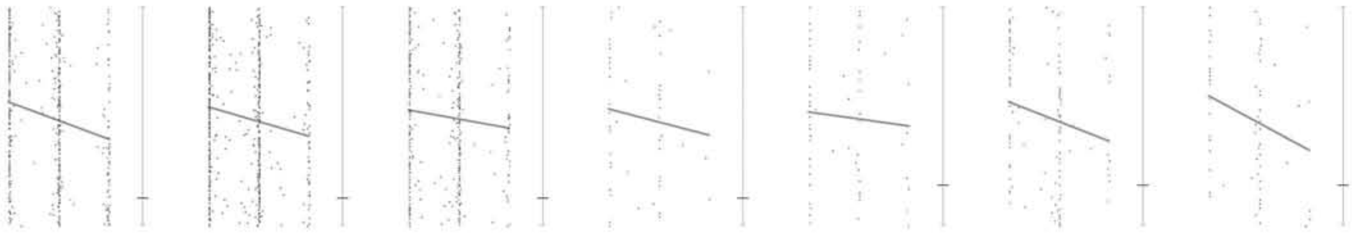

Dataset	Genotypes	Corr	R2	Z-Score	P-Value
HT12-Controls	GG (153) GA (194) AA (67)	-0.204	0.042	-4.19	2.79E-5
HT12-COPD	GG (202) GA (200) AA (51)	-0.152	0.023	-3.244	0.001
HT12-ALS	GG (132) GA (148) AA (44)	-0.095	0.009	-1.71	0.087
HT12-UC	GG (24) GA (22) AA (3)	-0.121	0.015	-0.826	0.409
H8v2-Controls	GG (28) GA (21) AA (10)	-0.079	0.006	-0.596	0.551
H8v2-CeliacDisease	GG (41) GA (57) AA (13)	-0.189	0.036	-1.986	0.047
H8v2-ALS	GG (20) GA (32) AA (7)	-0.257	0.066	-1.967	0.049

SNP rs12485738 (chr. 3, 56840816 bp)
Probe 6960022 (chr. 22, 18085526 - 18093297 bp), SEPT5///GP1BB
P-Value 1.47E-9

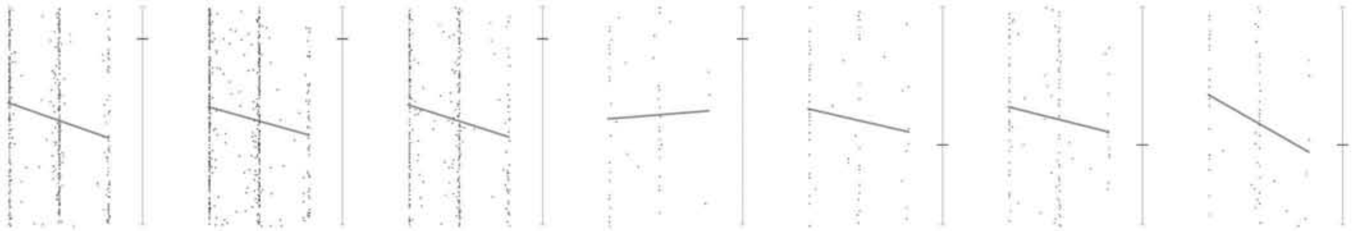

Dataset	Genotypes	Corr	R2	Z-Score	P-Value
HT12-Controls	GG (153) GA (194) AA (67)	-0.19	0.036	-3.898	9.68E-5
HT12-COPD	GG (202) GA (200) AA (51)	-0.142	0.02	-3.03	0.002
HT12-ALS	GG (132) GA (148) AA (44)	-0.164	0.027	-2.958	0.003
HT12-UC	GG (24) GA (22) AA (3)	0.038	0.001	0.258	0.796
H8v2-Controls	GG (28) GA (21) AA (10)	-0.128	0.016	-0.966	0.334
H8v2-CeliacDisease	GG (41) GA (57) AA (13)	-0.121	0.015	-1.263	0.207
H8v2-ALS	GG (20) GA (32) AA (7)	-0.271	0.073	-2.072	0.038

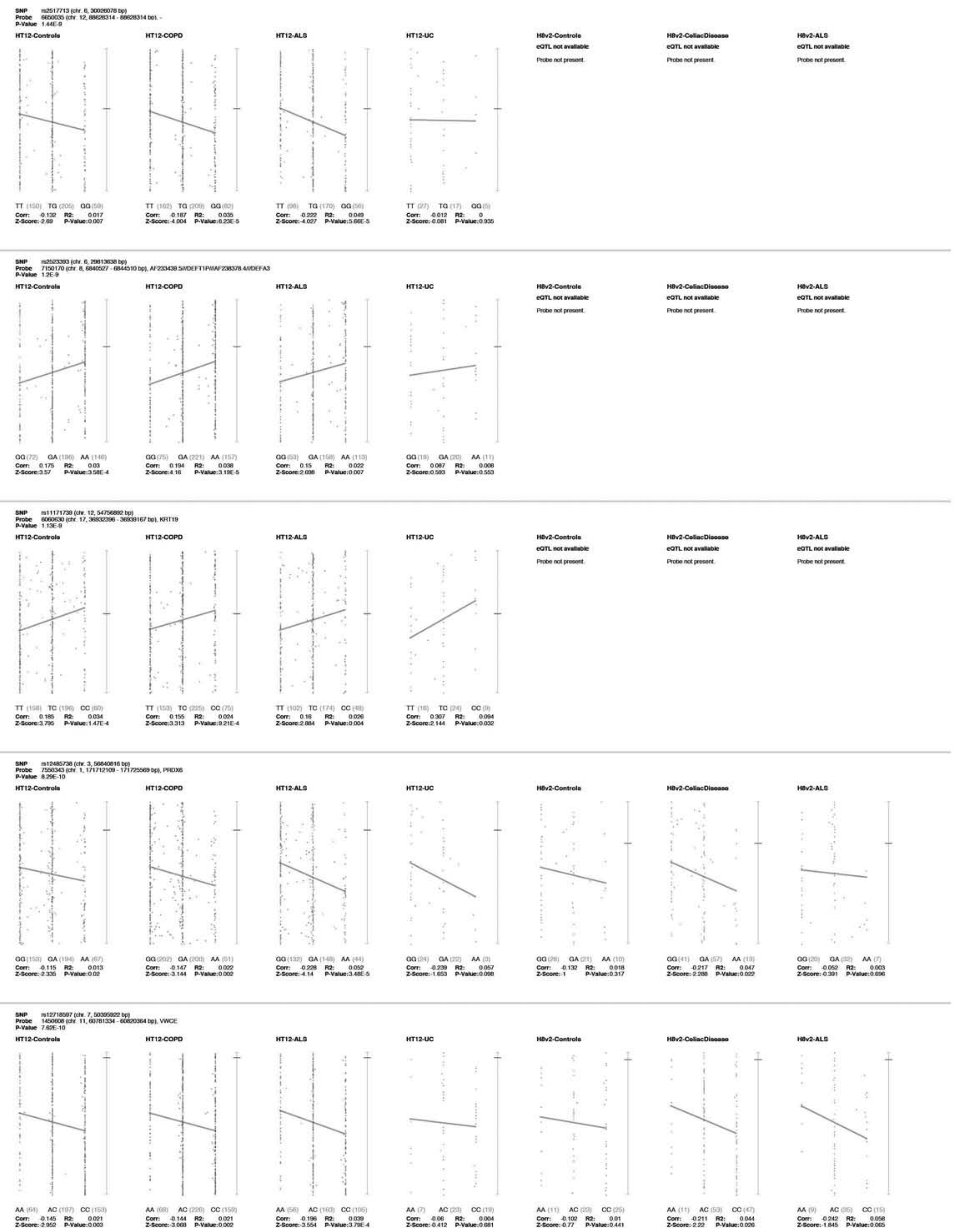
SNP rs2517713 (chr. 6, 30026078 bp)
Probe 6650035 (chr. 12, 88628314 - 88628314 bp), -
P-Value 1.44E-9
HT12-Controls
TT (150) TG (205) GG (59)
Corr: -0.132 R2: 0.017
Z-Score: -2.69 P-Value: 0.007
HT12-COPD
TT (162) TG (209) GG (82)
Corr: -0.187 R2: 0.035
Z-Score: -4.004 P-Value: 6.23E-5
HT12-ALS
TT (98) TG (170) GG (56)
Corr: -0.222 R2: 0.049
Z-Score: -4.027 P-Value: 5.66E-5
HT12-UC
TT (27) TG (17) GG (5)
Corr: -0.012 R2: 0
Z-Score: -0.081 P-Value: 0.935
H8v2-Controls
eQTL not available
Probe not present.
H8v2-CeliacDisease
eQTL not available
Probe not present.
H8v2-ALS
eQTL not available
Probe not present.
SNP rs2523393 (chr. 6, 29813638 bp)
Probe 7150170 (chr. 8, 6840527 - 6844510 bp), AF233439.5///DEFT1P///AF238378.4///DEFA3
P-Value 1.2E-9
HT12-Controls
GG (72) GA (196) AA (146)
Corr: 0.175 R2: 0.03
Z-Score: 3.57 P-Value: 3.58E-4
HT12-COPD
GG (75) GA (221) AA (157)
Corr: 0.194 R2: 0.038
Z-Score: 4.16 P-Value: 3.19E-5
HT12-ALS
GG (53) GA (158) AA (113)
Corr: 0.15 R2: 0.022
Z-Score: 2.698 P-Value: 0.007
HT12-UC
GG (18) GA (20) AA (11)
Corr: 0.087 R2: 0.008
Z-Score: 0.593 P-Value: 0.553
H8v2-Controls
eQTL not available
Probe not present.
H8v2-CeliacDisease
eQTL not available
Probe not present.
H8v2-ALS
eQTL not available
Probe not present.
SNP rs11171739 (chr. 12, 54756892 bp)
Probe 6060630 (chr. 17, 36932396 - 36939167 bp), KRT19
P-Value 1.13E-9
HT12-Controls
TT (158) TC (196) CC (60)
Corr: 0.185 R2: 0.034
Z-Score: 3.795 P-Value: 1.47E-4
HT12-COPD
TT (153) TC (225) CC (75)
Corr: 0.155 R2: 0.024
Z-Score: 3.313 P-Value: 9.21E-4
HT12-ALS
TT (102) TC (174) CC (48)
Corr: 0.16 R2: 0.026
Z-Score: 2.884 P-Value: 0.004
HT12-UC
TT (16) TC (24) CC (9)
Corr: 0.307 R2: 0.094
Z-Score: 2.144 P-Value: 0.032
H8v2-Controls
eQTL not available
Probe not present.
H8v2-CeliacDisease
eQTL not available
Probe not present.
H8v2-ALS
eQTL not available
Probe not present.
SNP rs12485738 (chr. 3, 56840816 bp)
Probe 7550343 (chr. 1, 171712109 - 171725569 bp), PRDX6
P-Value 8.29E-10
HT12-Controls
GG (153) GA (194) AA (67)
Corr: -0.115 R2: 0.013
Z-Score: -2.335 P-Value: 0.02
HT12-COPD
GG (202) GA (200) AA (51)
Corr: -0.147 R2: 0.022
Z-Score: -3.144 P-Value: 0.002
HT12-ALS
GG (132) GA (148) AA (44)
Corr: -0.228 R2: 0.052
Z-Score: -4.14 P-Value: 3.48E-5
HT12-UC
GG (24) GA (22) AA (3)
Corr: -0.239 R2: 0.057
Z-Score: -1.653 P-Value: 0.098
H8v2-Controls
GG (28) GA (21) AA (10)
Corr: -0.132 R2: 0.018
Z-Score: -1 P-Value: 0.317
H8v2-CeliacDisease
GG (41) GA (57) AA (13)
Corr: -0.217 R2: 0.047
Z-Score: -2.288 P-Value: 0.022
H8v2-ALS
GG (20) GA (32) AA (7)
Corr: -0.052 R2: 0.003
Z-Score: -0.391 P-Value: 0.696
SNP rs12718597 (chr. 7, 50395922 bp)
Probe 1450608 (chr. 11, 60781334 - 60820364 bp), VWCE
P-Value 7.62E-10
HT12-Controls
AA (64) AC (197) CC (153)
Corr: -0.145 R2: 0.021
Z-Score: -2.952 P-Value: 0.003
HT12-COPD
AA (68) AC (226) CC (159)
Corr: -0.144 R2: 0.021
Z-Score: -3.068 P-Value: 0.002
HT12-ALS
AA (56) AC (163) CC (105)
Corr: -0.196 R2: 0.039
Z-Score: -3.554 P-Value: 3.79E-4
HT12-UC
AA (7) AC (23) CC (19)
Corr: -0.06 R2: 0.004
Z-Score: -0.412 P-Value: 0.681
H8v2-Controls
AA (11) AC (23) CC (25)
Corr: -0.102 R2: 0.01
Z-Score: -0.77 P-Value: 0.441
H8v2-CeliacDisease
AA (11) AC (53) CC (47)
Corr: -0.211 R2: 0.044
Z-Score: -2.22 P-Value: 0.026
H8v2-ALS
AA (9) AC (35) CC (15)
Corr: -0.242 R2: 0.058
Z-Score: -1.845 P-Value: 0.065

SNP rs3131379 (chr. 6, 31829012 bp)
Probe 1110300 (chr. 4, 153765727 - 153821641 bp), TMEM154
P-Value 6.79E-10

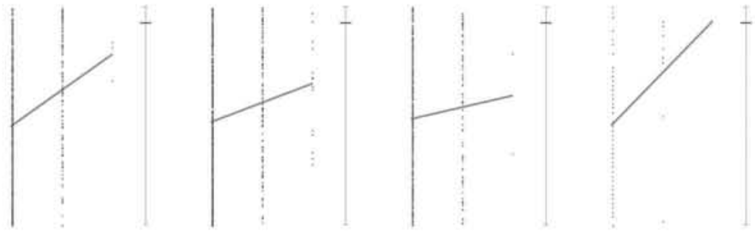

HT12-Controls	HT12-COPD	HT12-ALS	HT12-UC	H8v2-Controls	H8v2-CeliacDisease	H8v2-ALS
GG (310) GA (101) AA (3)	GG (334) GA (105) AA (14)	GG (248) GA (74) AA (2)	GG (41) GA (8) AA (0)	eQTL not available	eQTL not available	eQTL not available
Corr: 0.253 R2: 0.064	Corr: 0.155 R2: 0.024	Corr: 0.081 R2: 0.006	Corr: 0.297 R2: 0.088	Probe not present.	Probe not present.	Probe not present.
Z-Score:5.212 P-Value:1.87E-7	Z-Score:3.301 P-Value:9.62E-4	Z-Score:1.448 P-Value:0.148	Z-Score:2.07 P-Value:0.038			

SNP rs3117582 (chr. 6, 31728499 bp)
Probe 1110300 (chr. 4, 153765727 - 153821641 bp), TMEM154
P-Value 4.88E-10

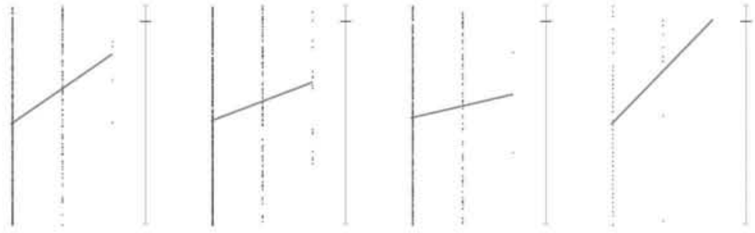

HT12-Controls	HT12-COPD	HT12-ALS	HT12-UC	H8v2-Controls	H8v2-CeliacDisease	H8v2-ALS
TT (311) TG (99) GG (4)	TT (336) TG (101) GG (16)	TT (248) TG (74) GG (2)	TT (41) TG (8) GG (0)	eQTL not available	eQTL not available	eQTL not available
Corr: 0.253 R2: 0.064	Corr: 0.159 R2: 0.025	Corr: 0.081 R2: 0.006	Corr: 0.297 R2: 0.088	Probe not present.	Probe not present.	Probe not present.
Z-Score:5.226 P-Value:1.74E-7	Z-Score:3.406 P-Value:6.58E-4	Z-Score:1.448 P-Value:0.148	Z-Score:2.07 P-Value:0.038			

SNP rs12485738 (chr. 3, 56840816 bp)
Probe 6290747 (chr. 11, 124117765 - 124125826 bp), NRGN///VSIG2
P-Value 4.85E-10

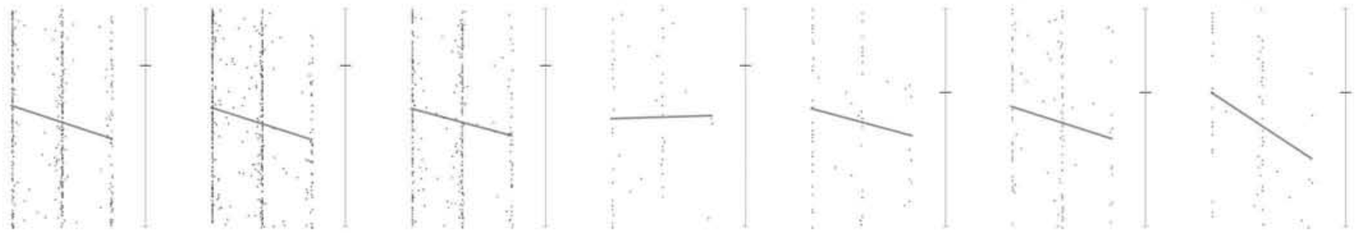

HT12-Controls	HT12-COPD	HT12-ALS	HT12-UC	H8v2-Controls	H8v2-CeliacDisease	H8v2-ALS
GG (153) GA (194) AA (67)	GG (202) GA (200) AA (51)	GG (132) GA (148) AA (44)	GG (24) GA (22) AA (3)	GG (28) GA (21) AA (10)	GG (41) GA (57) AA (13)	GG (20) GA (32) AA (7)
Corr: -0.179 R2: 0.032	Corr: -0.163 R2: 0.027	Corr: -0.142 R2: 0.02	Corr: 0.014 R2: 0	Corr: -0.148 R2: 0.022	Corr: -0.157 R2: 0.025	Corr: -0.314 R2: 0.099
Z-Score:-3.666 P-Value:2.47E-4	Z-Score:-3.481 P-Value:5E-4	Z-Score:-2.558 P-Value:0.011	Z-Score:0.094 P-Value:0.925	Z-Score:-1.121 P-Value:0.262	Z-Score:-1.65 P-Value:0.099	Z-Score:-2.424 P-Value:0.015

SNP rs6457620 (chr. 6, 32771977 bp)
Probe 2940424 (chr. 7, 36518135 - 36731595 bp), AOAH
P-Value 3.84E-10

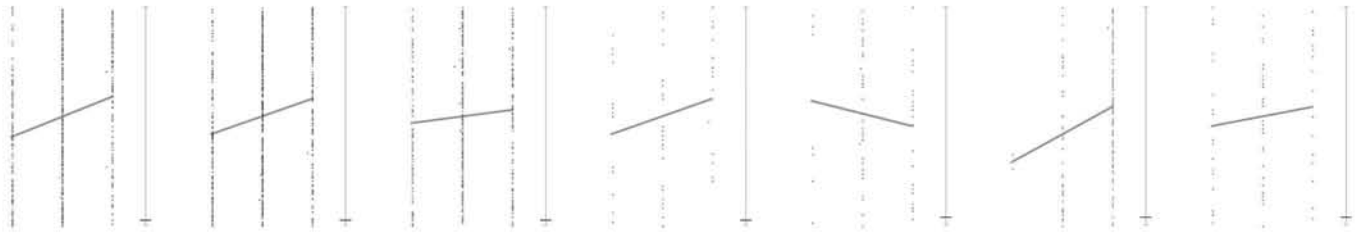

HT12-Controls	HT12-COPD	HT12-ALS	HT12-UC	H8v2-Controls	H8v2-CeliacDisease	H8v2-ALS
CC (99) CG (209) GG (106)	CC (110) CG (227) GG (118)	CC (75) CG (156) GG (93)	CC (13) CG (22) GG (14)	CC (7) CG (31) GG (21)	CC (2) CG (35) GG (74)	CC (17) CG (27) GG (15)
Corr: 0.225 R2: 0.051	Corr: 0.193 R2: 0.037	Corr: 0.074 R2: 0.005	Corr: 0.203 R2: 0.041	Corr: -0.123 R2: 0.015	Corr: 0.225 R2: 0.051	Corr: 0.108 R2: 0.012
Z-Score:4.621 P-Value:3.82E-6	Z-Score:4.139 P-Value:3.49E-5	Z-Score:1.331 P-Value:0.183	Z-Score:1.399 P-Value:0.162	Z-Score:-0.93 P-Value:0.352	Z-Score:2.379 P-Value:0.017	Z-Score:0.816 P-Value:0.415

SNP rs12718597 (chr. 7, 50395922 bp)
Probe 6270373 (chr. 6, 13898000 - 13923768 bp), CCDC90A
P-Value 3.33E-10

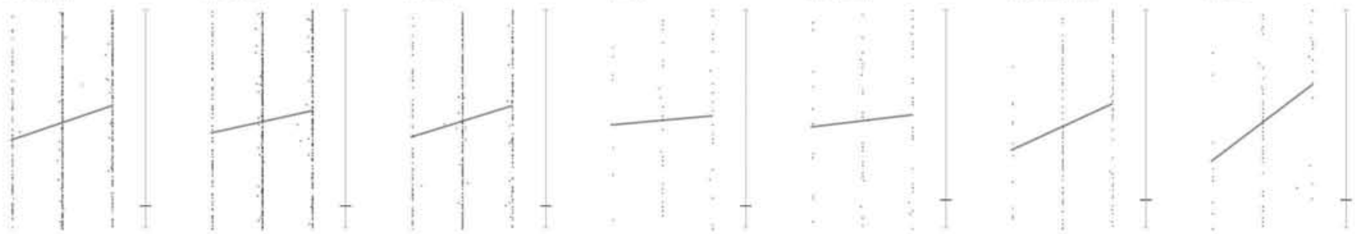

HT12-Controls	HT12-COPD	HT12-ALS	HT12-UC	H8v2-Controls	H8v2-CeliacDisease	H8v2-ALS
AA (64) AC (197) CC (153)	AA (68) AC (226) CC (159)	AA (56) AC (163) CC (105)	AA (7) AC (23) CC (19)	AA (11) AC (23) CC (25)	AA (11) AC (53) CC (47)	AA (9) AC (35) CC (15)
Corr: 0.189 R2: 0.036	Corr: 0.117 R2: 0.014	Corr: 0.17 R2: 0.029	Corr: 0.043 R2: 0.002	Corr: 0.069 R2: 0.005	Corr: 0.23 R2: 0.053	Corr: 0.37 R2: 0.137
Z-Score:3.864 P-Value:1.12E-4	Z-Score:2.499 P-Value:0.012	Z-Score:3.068 P-Value:0.002	Z-Score:0.294 P-Value:0.769	Z-Score:0.519 P-Value:0.604	Z-Score:2.432 P-Value:0.015	Z-Score:2.882 P-Value:0.004

SNP rs6457617 (chr. 6, 32771829 bp)
Probe 2940424 (chr. 7, 36518135 - 36731595 bp), AOAH
P-Value 3.02E-10

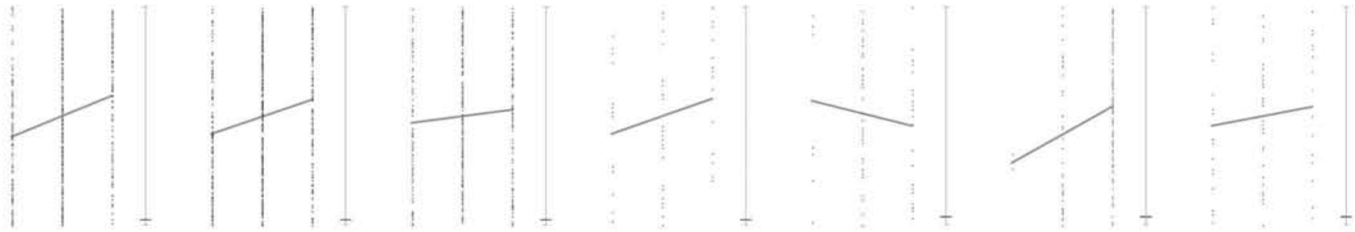

Dataset	Genotypes	Corr	R2	Z-Score	P-Value
HT12-Controls	TT (99) TC (208) CC (107)	0.229	0.053	4.716	2.4E-6
HT12-COPD	TT (110) TC (227) CC (118)	0.193	0.037	4.126	3.68E-5
HT12-ALS	TT (75) TC (155) CC (94)	0.074	0.005	1.322	0.186
HT12-UC	TT (13) TC (22) CC (14)	0.202	0.041	1.394	0.163
H8v2-Controls	TT (7) TC (31) CC (21)	-0.123	0.015	-0.929	0.353
H8v2-CeliacDisease	TT (2) TC (34) CC (75)	0.223	0.05	2.357	0.018
H8v2-ALS	TT (17) TC (27) CC (15)	0.108	0.012	0.816	0.415

SNP rs2517713 (chr. 6, 30026078 bp)
Probe 5560500 (chr. 13, 76352630 - 76358061 bp), KCTD12
P-Value 2.74E-10

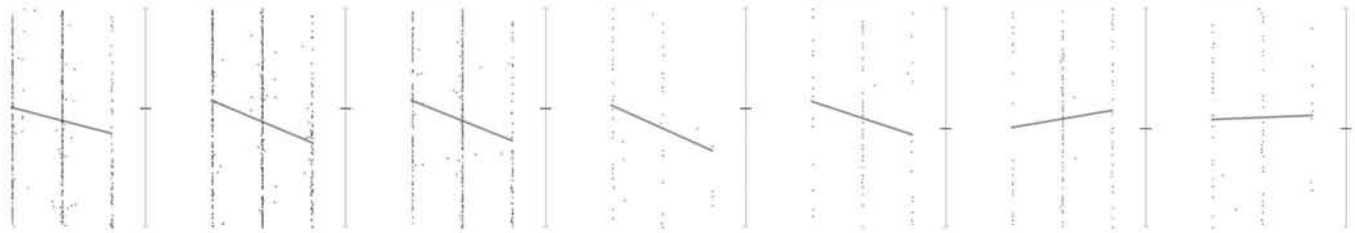

Dataset	Genotypes	Corr	R2	Z-Score	P-Value
HT12-Controls	TT (150) TG (205) GG (59)	-0.138	0.019	-2.815	0.005
HT12-COPD	TT (162) TG (209) GG (82)	-0.232	0.054	-5.003	5.64E-7
HT12-ALS	TT (98) TG (170) GG (56)	-0.209	0.044	-3.798	1.46E-4
HT12-UC	TT (27) TG (17) GG (5)	-0.229	0.052	-1.581	0.114
H8v2-Controls	TT (14) TG (32) GG (13)	-0.174	0.03	-1.317	0.188
H8v2-CeliacDisease	TT (20) TG (62) GG (29)	0.089	0.008	0.927	0.354
H8v2-ALS	TT (23) TG (28) GG (8)	0.02	0	0.152	0.879

SNP rs628751 (chr. 6, 139880112 bp)
Probe 5270148 (chr. 14, 66923814 - 66923814 bp), -
P-Value 1.94E-10

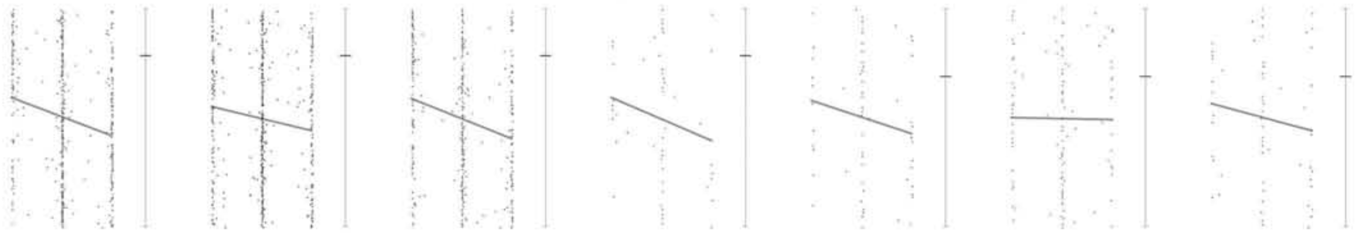

Dataset	Genotypes	Corr	R2	Z-Score	P-Value
HT12-Controls	AA (82) AC (216) CC (116)	-0.202	0.041	-4.148	3.35E-5
HT12-COPD	AA (116) AC (236) CC (101)	-0.125	0.016	-2.665	0.008
HT12-ALS	AA (95) AC (149) CC (80)	-0.225	0.051	-4.088	4.35E-5
HT12-UC	AA (11) AC (29) CC (9)	-0.208	0.043	-1.437	0.151
H8v2-Controls	AA (11) AC (31) CC (17)	-0.168	0.028	-1.273	0.203
H8v2-CeliacDisease	AA (30) AC (57) CC (24)	-0.01	0	-0.108	0.914
H8v2-ALS	AA (14) AC (28) CC (17)	-0.148	0.022	-1.118	0.263

SNP rs12485738 (chr. 3, 56840816 bp)
Probe 1050008 (chr. 4, 91018706 - 91095803 bp), MMRN1
P-Value 1.95E-10

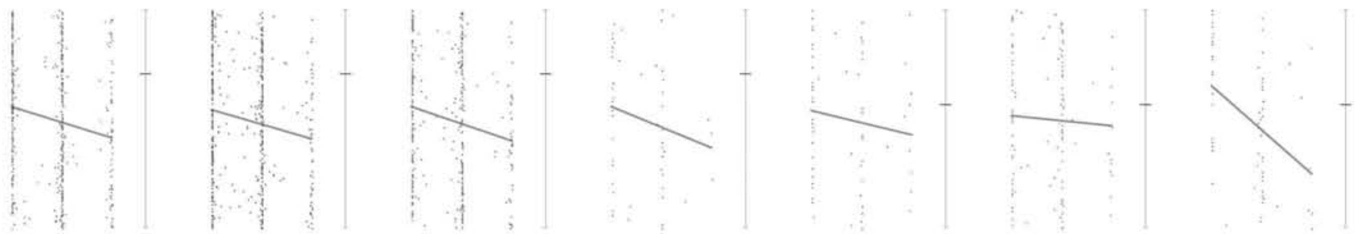

Dataset	Genotypes	Corr	R2	Z-Score	P-Value
HT12-Controls	GG (153) GA (194) AA (67)	-0.168	0.028	-3.434	5.94E-4
HT12-COPD	GG (202) GA (200) AA (51)	-0.151	0.023	-3.22	0.001
HT12-ALS	GG (132) GA (148) AA (44)	-0.178	0.032	-3.221	0.001
HT12-UC	GG (24) GA (22) AA (3)	-0.187	0.035	-1.285	0.199
H8v2-Controls	GG (28) GA (21) AA (10)	-0.131	0.017	-0.992	0.321
H8v2-CeliacDisease	GG (41) GA (57) AA (13)	-0.05	0.003	-0.521	0.602
H8v2-ALS	GG (20) GA (32) AA (7)	-0.421	0.177	-3.32	8.99E-4

SNP rs643381 (chr. 6, 139881116 bp)
Probe 5270148 (chr. 14, 66923814 - 66923814 bp), -
P-Value 1.77E-10

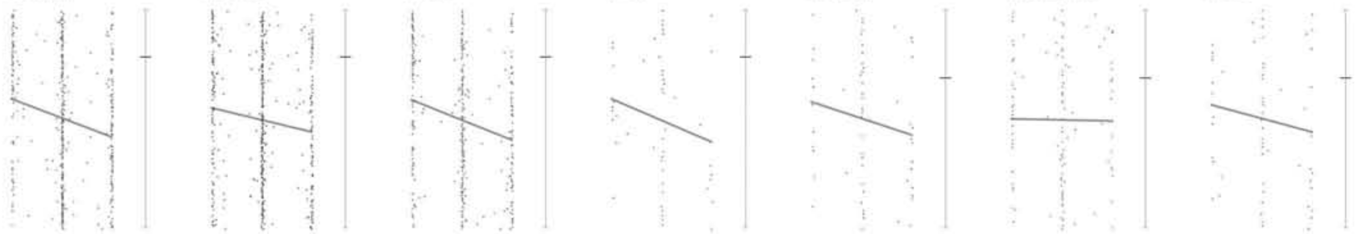

Dataset	Genotypes	Corr	R2	Z-Score	P-Value
HT12-Controls	AA (82) AC (216) CC (116)	-0.203	0.041	-4.158	3.21E-5
HT12-COPD	AA (116) AC (236) CC (101)	-0.125	0.016	-2.661	0.008
HT12-ALS	AA (95) AC (149) CC (80)	-0.225	0.051	-4.09	4.31E-5
HT12-UC	AA (11) AC (29) CC (9)	-0.209	0.044	-1.44	0.15
H8v2-Controls	AA (11) AC (31) CC (17)	-0.169	0.029	-1.282	0.2
H8v2-CeliacDisease	AA (30) AC (57) CC (24)	-0.01	0	-0.106	0.916
H8v2-ALS	AA (14) AC (28) CC (17)	-0.148	0.022	-1.118	0.264

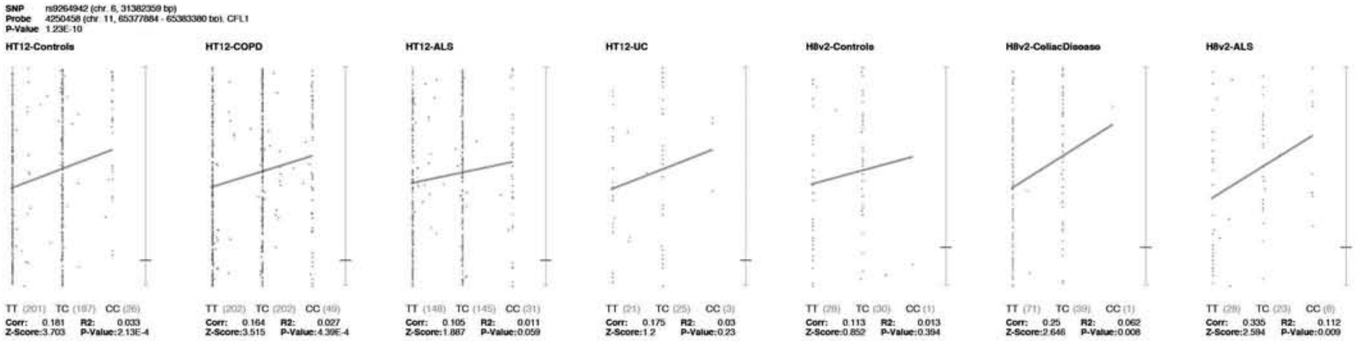
SNP rs9264942 (chr. 6, 31382359 bp)
Probe 4250458 (chr. 11, 65377884 - 65383380 bp), CFL1
P-Value 1.23E-10
HT12-Controls
TT (201) TC (187) CC (26)
Corr: 0.181 R2: 0.033
Z-Score:3.703 P-Value:2.13E-4
HT12-COPD
TT (202) TC (202) CC (49)
Corr: 0.164 R2: 0.027
Z-Score:3.515 P-Value:4.39E-4
HT12-ALS
TT (148) TC (145) CC (31)
Corr: 0.105 R2: 0.011
Z-Score:1.887 P-Value:0.059
HT12-UC
TT (21) TC (25) CC (3)
Corr: 0.175 R2: 0.03
Z-Score:1.2 P-Value:0.23
H8v2-Controls
TT (28) TC (30) CC (1)
Corr: 0.113 R2: 0.013
Z-Score:0.852 P-Value:0.394
H8v2-CeliacDisease
TT (71) TC (39) CC (1)
Corr: 0.25 R2: 0.062
Z-Score:2.646 P-Value:0.008
H8v2-ALS
TT (28) TC (23) CC (8)
Corr: 0.335 R2: 0.112
Z-Score:2.594 P-Value:0.009

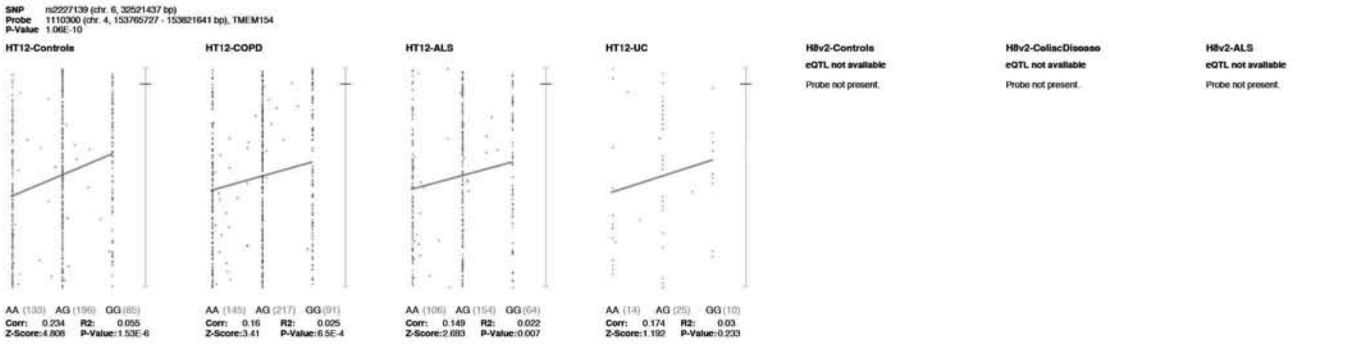
SNP rs2227139 (chr. 6, 32521437 bp)
Probe 1110300 (chr. 4, 153765727 - 153821641 bp), TMEM154
P-Value 1.06E-10
HT12-Controls
AA (133) AG (196) GG (85)
Corr: 0.234 R2: 0.055
Z-Score:4.808 P-Value:1.53E-6
HT12-COPD
AA (145) AG (217) GG (91)
Corr: 0.16 R2: 0.025
Z-Score:3.41 P-Value:6.5E-4
HT12-ALS
AA (106) AG (154) GG (64)
Corr: 0.149 R2: 0.022
Z-Score:2.693 P-Value:0.007
HT12-UC
AA (14) AG (25) GG (10)
Corr: 0.174 R2: 0.03
Z-Score:1.192 P-Value:0.233
H8v2-Controls
eQTL not available
Probe not present.
H8v2-CeliacDisease
eQTL not available
Probe not present.
H8v2-ALS
eQTL not available
Probe not present.

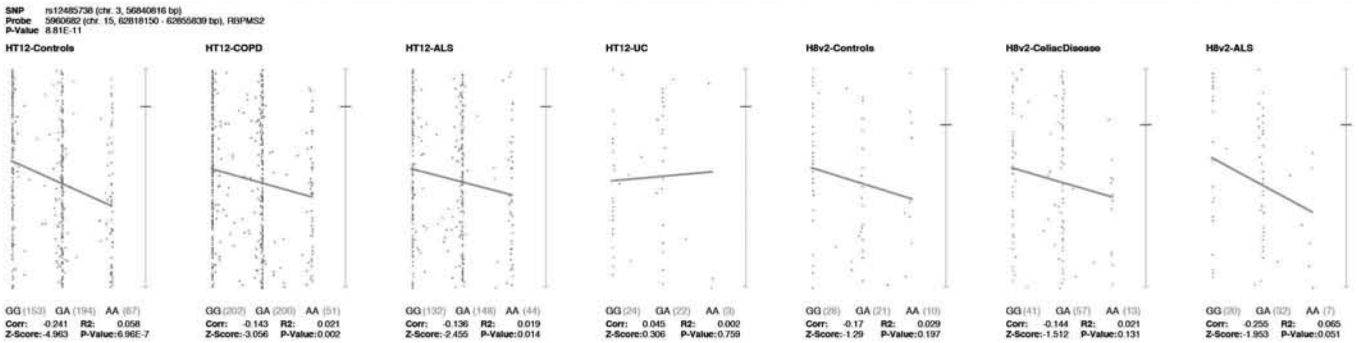
SNP rs12485738 (chr. 3, 56840816 bp)
Probe 5960682 (chr. 15, 62818150 - 62855839 bp), RBPMS2
P-Value 8.81E-11
HT12-Controls
GG (153) GA (194) AA (67)
Corr: -0.241 R2: 0.058
Z-Score:-4.963 P-Value:6.96E-7
HT12-COPD
GG (202) GA (200) AA (51)
Corr: -0.143 R2: 0.021
Z-Score:-3.056 P-Value:0.002
HT12-ALS
GG (132) GA (148) AA (44)
Corr: -0.136 R2: 0.019
Z-Score:-2.455 P-Value:0.014
HT12-UC
GG (24) GA (22) AA (3)
Corr: 0.045 R2: 0.002
Z-Score:0.306 P-Value:0.759
H8v2-Controls
GG (28) GA (21) AA (10)
Corr: -0.17 R2: 0.029
Z-Score:-1.29 P-Value:0.197
H8v2-CeliacDisease
GG (41) GA (57) AA (13)
Corr: -0.144 R2: 0.021
Z-Score:-1.512 P-Value:0.131
H8v2-ALS
GG (20) GA (32) AA (7)
Corr: -0.255 R2: 0.065
Z-Score:-1.953 P-Value:0.051

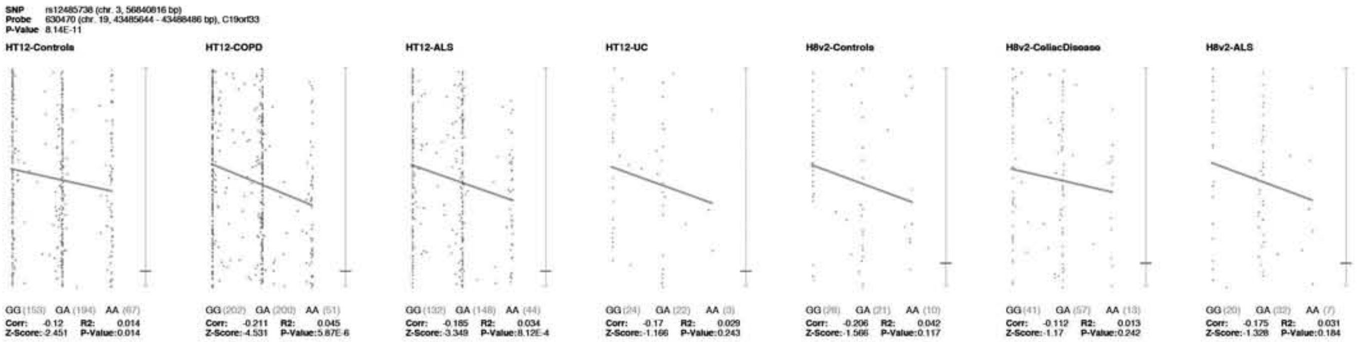
SNP rs12485738 (chr. 3, 56840816 bp)
Probe 630470 (chr. 19, 43485644 - 43488486 bp), C19orf33
P-Value 8.14E-11
HT12-Controls
GG (153) GA (194) AA (67)
Corr: -0.12 R2: 0.014
Z-Score:-2.451 P-Value:0.014
HT12-COPD
GG (202) GA (200) AA (51)
Corr: -0.211 R2: 0.045
Z-Score:-4.531 P-Value:5.87E-6
HT12-ALS
GG (132) GA (148) AA (44)
Corr: -0.185 R2: 0.034
Z-Score:-3.349 P-Value:8.12E-4
HT12-UC
GG (24) GA (22) AA (3)
Corr: -0.17 R2: 0.029
Z-Score:-1.166 P-Value:0.243
H8v2-Controls
GG (28) GA (21) AA (10)
Corr: -0.206 R2: 0.042
Z-Score:-1.566 P-Value:0.117
H8v2-CeliacDisease
GG (41) GA (57) AA (13)
Corr: -0.112 R2: 0.013
Z-Score:-1.17 P-Value:0.242
H8v2-ALS
GG (20) GA (32) AA (7)
Corr: -0.175 R2: 0.031
Z-Score:-1.328 P-Value:0.184

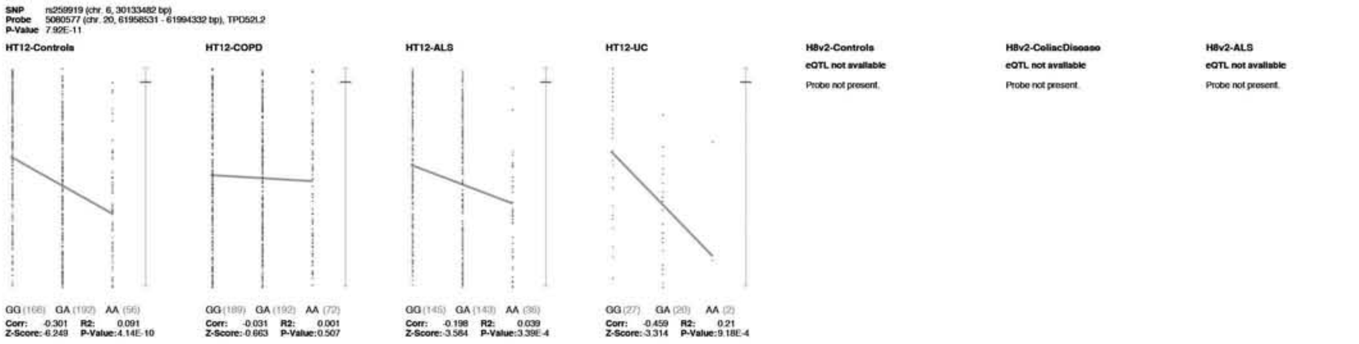
SNP rs259919 (chr. 6, 30133482 bp)
Probe 5080577 (chr. 20, 61958531 - 61994332 bp), TPD52L2
P-Value 7.92E-11
HT12-Controls
GG (166) GA (192) AA (56)
Corr: -0.301 R2: 0.091
Z-Score:-6.249 P-Value:4.14E-10
HT12-COPD
GG (189) GA (192) AA (72)
Corr: -0.031 R2: 0.001
Z-Score:-0.663 P-Value:0.507
HT12-ALS
GG (145) GA (143) AA (36)
Corr: -0.198 R2: 0.039
Z-Score:-3.584 P-Value:3.39E-4
HT12-UC
GG (27) GA (20) AA (2)
Corr: -0.459 R2: 0.21
Z-Score:-3.314 P-Value:9.18E-4
H8v2-Controls
eQTL not available
Probe not present.
H8v2-CeliacDisease
eQTL not available
Probe not present.
H8v2-ALS
eQTL not available
Probe not present.

SNP rs12485738 (chr. 3, 56840816 bp)
Probe 6330474 (chr. 11, 825298 - 831681 bp), CD151///POLR2L
P-Value 7.53E-11

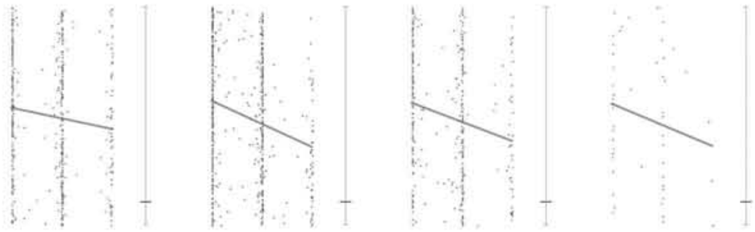

HT12-Controls
GG (153) GA (194) AA (67)
Corr: -0.115 R2: 0.013
Z-Score: -2.34 P-Value: 0.019

HT12-COPD
GG (202) GA (200) AA (51)
Corr: -0.234 R2: 0.055
Z-Score: -5.044 P-Value: 4.56E-7

HT12-ALS
GG (132) GA (148) AA (44)
Corr: -0.2 R2: 0.04
Z-Score: -3.626 P-Value: 2.88E-4

HT12-UC
GG (24) GA (22) AA (3)
Corr: -0.191 R2: 0.036
Z-Score: -1.314 P-Value: 0.189

H8v2-Controls
eQTL not available
Probe not present.

H8v2-CeliacDisease
eQTL not available
Probe not present.

H8v2-ALS
eQTL not available
Probe not present.

SNP rs9272346 (chr. 6, 32712350 bp)
Probe 2940424 (chr. 7, 36518135 - 36731595 bp), AOAH
P-Value 5.08E-11

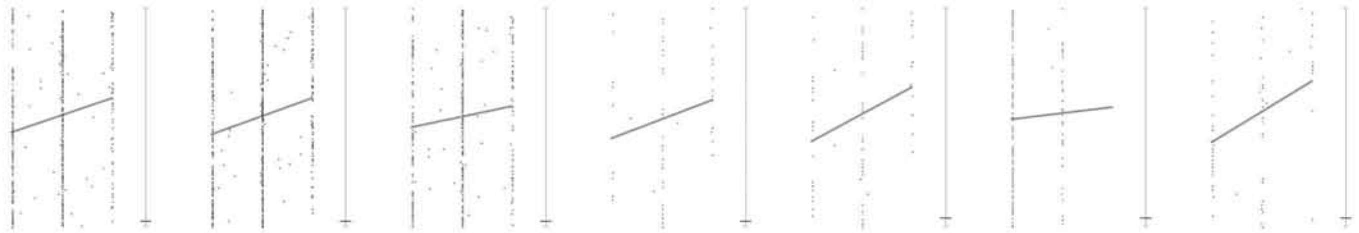

HT12-Controls
AA (130) AG (214) GG (70)
Corr: 0.182 R2: 0.033
Z-Score: 3.727 P-Value: 1.94E-4

HT12-COPD
AA (134) AG (235) GG (84)
Corr: 0.193 R2: 0.037
Z-Score: 4.133 P-Value: 3.58E-5

HT12-ALS
AA (97) AG (165) GG (62)
Corr: 0.111 R2: 0.012
Z-Score: 1.996 P-Value: 0.046

HT12-UC
AA (10) AG (26) GG (13)
Corr: 0.2 R2: 0.04
Z-Score: 1.376 P-Value: 0.169

H8v2-Controls
AA (20) AG (28) GG (11)
Corr: 0.288 R2: 0.083
Z-Score: 2.216 P-Value: 0.027

H8v2-CeliacDisease
AA (79) AG (32) GG (0)
Corr: 0.042 R2: 0.002
Z-Score: 0.434 P-Value: 0.664

H8v2-ALS
AA (24) AG (23) GG (12)
Corr: 0.349 R2: 0.122
Z-Score: 2.706 P-Value: 0.007

SNP rs2523393 (chr. 6, 29813638 bp)
Probe 5560500 (chr. 13, 76352630 - 76358061 bp), KCTD12
P-Value 4.7E-11

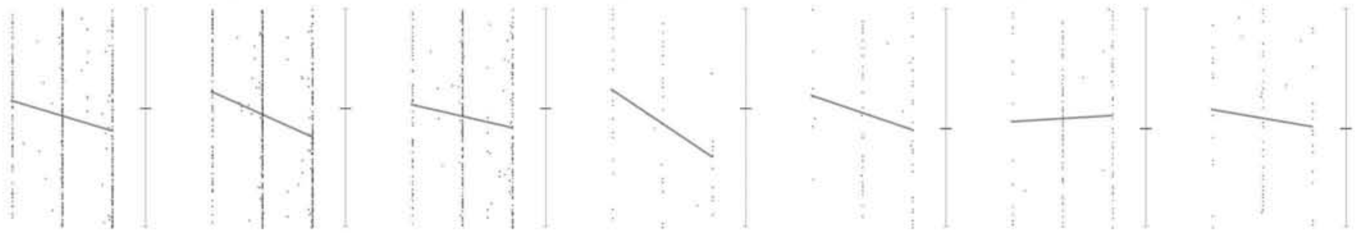

HT12-Controls
GG (72) GA (196) AA (146)
Corr: -0.164 R2: 0.027
Z-Score: -3.351 P-Value: 8.04E-4

HT12-COPD
GG (75) GA (221) AA (157)
Corr: -0.246 R2: 0.061
Z-Score: -5.311 P-Value: 1.09E-7

HT12-ALS
GG (53) GA (158) AA (113)
Corr: -0.123 R2: 0.015
Z-Score: -2.222 P-Value: 0.026

HT12-UC
GG (18) GA (20) AA (11)
Corr: -0.388 R2: 0.15
Z-Score: -2.754 P-Value: 0.006

H8v2-Controls
GG (7) GA (25) AA (27)
Corr: -0.177 R2: 0.031
Z-Score: -1.343 P-Value: 0.179

H8v2-CeliacDisease
GG (14) GA (54) AA (43)
Corr: 0.031 R2: 0.001
Z-Score: 0.321 P-Value: 0.748

H8v2-ALS
GG (12) GA (32) AA (15)
Corr: -0.089 R2: 0.008
Z-Score: -0.667 P-Value: 0.505

SNP rs9268645 (chr. 6, 32516505 bp)
Probe 2940424 (chr. 7, 36518135 - 36731595 bp), AOAH
P-Value 3.13E-11

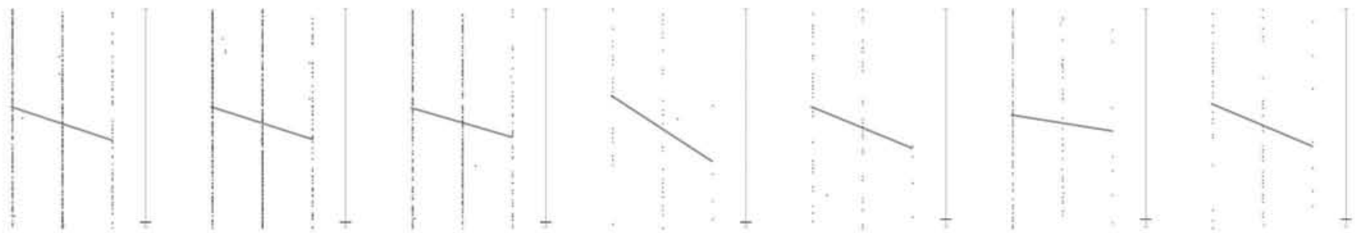

HT12-Controls
CC (172) CG (187) GG (55)
Corr: -0.178 R2: 0.032
Z-Score: -3.646 P-Value: 2.67E-4

HT12-COPD
CC (194) CG (197) GG (62)
Corr: -0.176 R2: 0.031
Z-Score: -3.768 P-Value: 1.65E-4

HT12-ALS
CC (139) CG (140) GG (45)
Corr: -0.161 R2: 0.026
Z-Score: -2.911 P-Value: 0.004

HT12-UC
CC (20) CG (25) GG (4)
Corr: -0.315 R2: 0.099
Z-Score: -2.202 P-Value: 0.028

H8v2-Controls
CC (29) CG (26) GG (4)
Corr: -0.197 R2: 0.039
Z-Score: -1.499 P-Value: 0.134

H8v2-CeliacDisease
CC (73) CG (31) GG (7)
Corr: -0.076 R2: 0.006
Z-Score: -0.792 P-Value: 0.428

H8v2-ALS
CC (24) CG (28) GG (7)
Corr: -0.218 R2: 0.048
Z-Score: -1.66 P-Value: 0.097

SNP rs3131379 (chr. 6, 31829012 bp)
Probe 6380347 (chr. 22, 40346241 - 40390998 bp), XRCC6
P-Value 2.72E-11

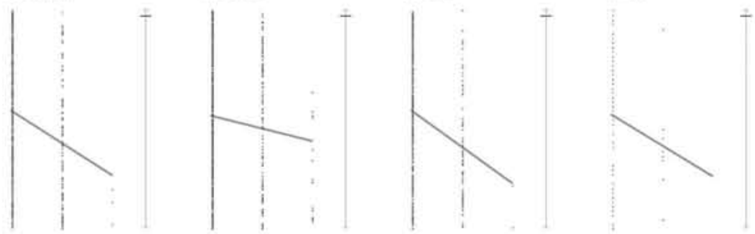

HT12-Controls
GG (310) GA (101) AA (3)
Corr: -0.231 R2: 0.053
Z-Score: -4.754 P-Value: 1.99E-6

HT12-COPD
GG (334) GA (105) AA (14)
Corr: -0.104 R2: 0.011
Z-Score: -2.214 P-Value: 0.027

HT12-ALS
GG (248) GA (74) AA (2)
Corr: -0.251 R2: 0.063
Z-Score: -4.577 P-Value: 4.72E-6

HT12-UC
GG (41) GA (8) AA (0)
Corr: -0.176 R2: 0.031
Z-Score: -1.208 P-Value: 0.227

H8v2-Controls
eQTL not available
Probe not present.

H8v2-CeliacDisease
eQTL not available
Probe not present.

H8v2-ALS
eQTL not available
Probe not present.

SNP rs9264942 (chr. 6, 31382359 bp)
Probe 1110300 (chr. 4, 153765727 - 153821641 bp), TMEM154
P-Value 2.59E-11

HT12-Controls | **HT12-COPD** | **HT12-ALS** | **HT12-UC**

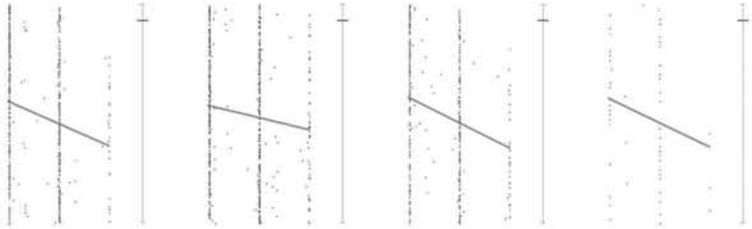

HT12-Controls	HT12-COPD	HT12-ALS	HT12-UC
TT (201) TC (197) CC (26)	TT (202) TC (202) CC (49)	TT (148) TC (145) CC (31)	TT (21) TC (25) CC (3)
Corr: -0.209 R2: 0.044	Corr: -0.124 R2: 0.015	Corr: -0.247 R2: 0.061	Corr: -0.215 R2: 0.046
Z-Score: -4.293 P-Value: 1.76E-5	Z-Score: -2.649 P-Value: 0.008	Z-Score: -4.504 P-Value: 6.66E-6	Z-Score: -1.487 P-Value: 0.137

H8v2-Controls
eQTL not available
Probe not present.

H8v2-CeliacDisease
eQTL not available
Probe not present.

H8v2-ALS
eQTL not available
Probe not present.

SNP rs12485738 (chr. 3, 56840816 bp)
Probe 6560398 (chr. 2, 73781171 - 73781171 bp), -
P-Value 2.54E-11

HT12-Controls | **HT12-COPD** | **HT12-ALS** | **HT12-UC** | **H8v2-Controls** | **H8v2-CeliacDisease** | **H8v2-ALS**

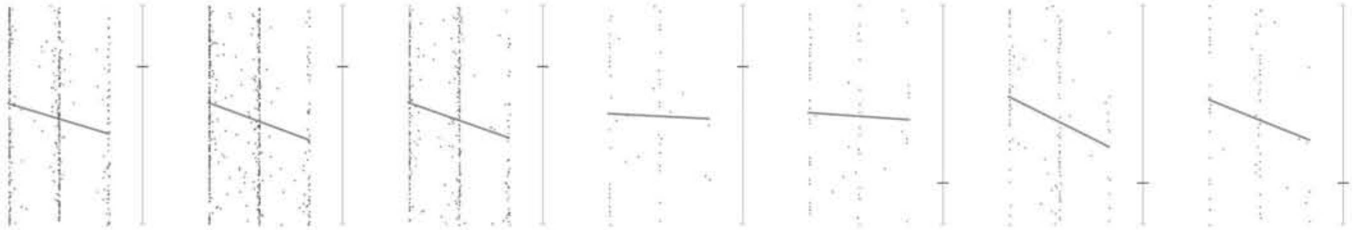

HT12-Controls	HT12-COPD	HT12-ALS	HT12-UC	H8v2-Controls	H8v2-CeliacDisease	H8v2-ALS
GG (153) GA (194) AA (67)	GG (202) GA (200) AA (51)	GG (132) GA (148) AA (44)	GG (24) GA (22) AA (3)	GG (28) GA (21) AA (10)	GG (41) GA (57) AA (13)	GG (20) GA (32) AA (7)
Corr: -0.164 R2: 0.027	Corr: -0.189 R2: 0.036	Corr: -0.183 R2: 0.033	Corr: -0.02 R2: 0	Corr: -0.036 R2: 0.001	Corr: -0.249 R2: 0.062	Corr: -0.189 R2: 0.036
Z-Score: -3.348 P-Value: 8.2E-4	Z-Score: -4.053 P-Value: 5.05E-5	Z-Score: -3.302 P-Value: 9.6E-4	Z-Score: -0.136 P-Value: 0.892	Z-Score: -0.274 P-Value: 0.784	Z-Score: -2.636 P-Value: 0.008	Z-Score: -1.437 P-Value: 0.151

SNP rs12485738 (chr. 3, 56840816 bp)
Probe 4880600 (chr. 6, 43001464 - 43001464 bp), -
P-Value 1.6E-11

HT12-Controls | **HT12-COPD** | **HT12-ALS** | **HT12-UC**

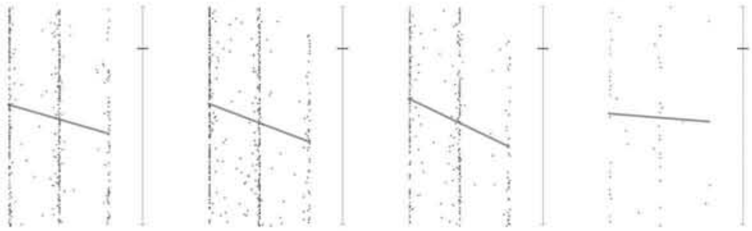

HT12-Controls	HT12-COPD	HT12-ALS	HT12-UC
GG (153) GA (194) AA (67)	GG (202) GA (200) AA (51)	GG (132) GA (148) AA (44)	GG (24) GA (22) AA (3)
Corr: -0.154 R2: 0.024	Corr: -0.195 R2: 0.038	Corr: -0.252 R2: 0.063	Corr: -0.036 R2: 0.001
Z-Score: -3.141 P-Value: 0.002	Z-Score: -4.184 P-Value: 2.86E-5	Z-Score: -4.587 P-Value: 4.49E-6	Z-Score: -0.247 P-Value: 0.805

H8v2-Controls
eQTL not available
Probe not present.

H8v2-CeliacDisease
eQTL not available
Probe not present.

H8v2-ALS
eQTL not available
Probe not present.

SNP rs3117582 (chr. 6, 31728499 bp)
Probe 6380347 (chr. 22, 40346241 - 40390998 bp), XRCC6
P-Value 1.52E-11

HT12-Controls | **HT12-COPD** | **HT12-ALS** | **HT12-UC**

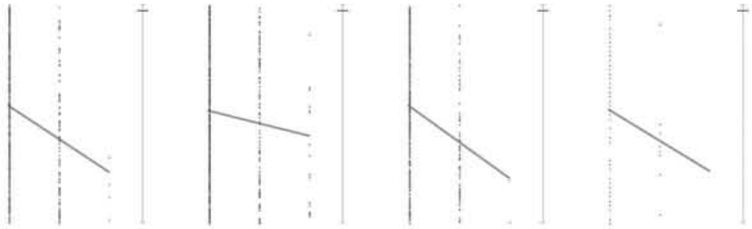

HT12-Controls	HT12-COPD	HT12-ALS	HT12-UC
TT (311) TG (99) GG (4)	TT (336) TG (101) GG (16)	TT (248) TG (74) GG (2)	TT (41) TG (8) GG (0)
Corr: -0.239 R2: 0.057	Corr: -0.103 R2: 0.011	Corr: -0.251 R2: 0.063	Corr: -0.176 R2: 0.031
Z-Score: -4.913 P-Value: 8.96E-7	Z-Score: -2.19 P-Value: 0.029	Z-Score: -4.577 P-Value: 4.72E-6	Z-Score: -1.208 P-Value: 0.227

H8v2-Controls
eQTL not available
Probe not present.

H8v2-CeliacDisease
eQTL not available
Probe not present.

H8v2-ALS
eQTL not available
Probe not present.

SNP rs2523393 (chr. 6, 29813638 bp)
Probe 6960017 (chr. 4, 109229903 - 109304132 bp), LEF1
P-Value 1.36E-11

HT12-Controls | **HT12-COPD** | **HT12-ALS** | **HT12-UC**

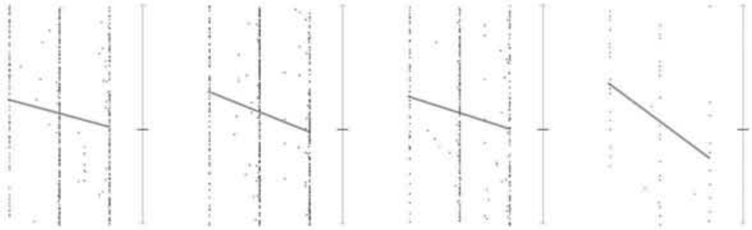

HT12-Controls	HT12-COPD	HT12-ALS	HT12-UC
GG (72) GA (196) AA (146)	GG (75) GA (221) AA (157)	GG (53) GA (158) AA (113)	GG (18) GA (20) AA (11)
Corr: -0.146 R2: 0.021	Corr: -0.218 R2: 0.048	Corr: -0.173 R2: 0.03	Corr: -0.43 R2: 0.185
Z-Score: -2.969 P-Value: 0.003	Z-Score: -4.692 P-Value: 2.71E-6	Z-Score: -3.126 P-Value: 0.002	Z-Score: -3.083 P-Value: 0.002

H8v2-Controls
eQTL not available
Probe not present.

H8v2-CeliacDisease
eQTL not available
Probe not present.

H8v2-ALS
eQTL not available
Probe not present.

SNP rs13437082 (chr. 6, 31462539 bp)
Probe 1110300 (chr. 4, 153765727 - 153821641 bp), TMEM154
P-Value 1.26E-11

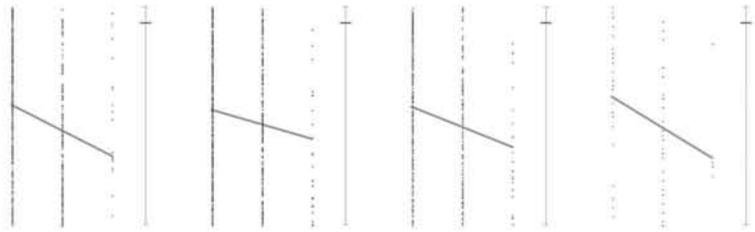

HT12-Controls	HT12-COPD	HT12-ALS	HT12-UC	H8v2-Controls	H8v2-CeliacDisease	H8v2-ALS
CC (248) CT (149) TT (17)	CC (260) CT (169) TT (24)	CC (188) CT (113) TT (23)	CC (22) CT (23) TT (4)	**eQTL not available**	**eQTL not available**	**eQTL not available**
**Corr:** -0.232 **R2:** 0.054	**Corr:** -0.137 **R2:** 0.019	**Corr:** -0.198 **R2:** 0.039	**Corr:** -0.301 **R2:** 0.09	Probe not present.	Probe not present.	Probe not present.
**Z-Score:** -4.772 **P-Value:** 1.82E-6	**Z-Score:** -2.926 **P-Value:** 0.003	**Z-Score:** -3.592 **P-Value:** 3.28E-4	**Z-Score:** -2.098 **P-Value:** 0.036			

SNP rs12485738 (chr. 3, 56840816 bp)
Probe 1580025 (chr. 7, 93387952 - 93394760 bp), GNG11
P-Value 1.15E-11

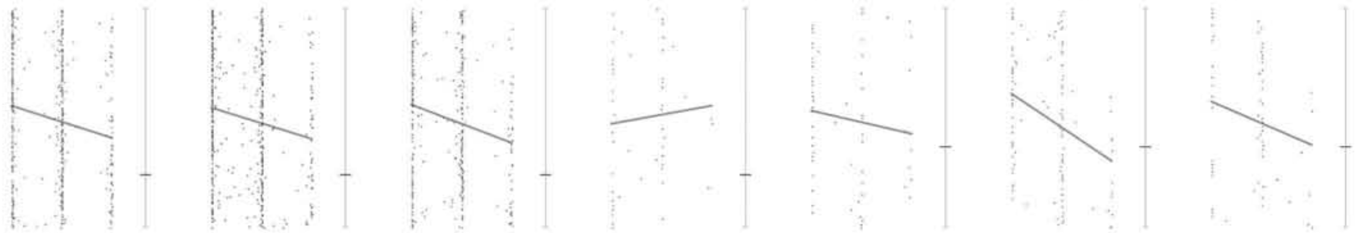

HT12-Controls	HT12-COPD	HT12-ALS	HT12-UC	H8v2-Controls	H8v2-CeliacDisease	H8v2-ALS
GG (153) GA (194) AA (67)	GG (202) GA (200) AA (51)	GG (132) GA (148) AA (44)	GG (24) GA (22) AA (3)	GG (28) GA (21) AA (10)	GG (41) GA (57) AA (13)	GG (20) GA (32) AA (7)
**Corr:** -0.175 **R2:** 0.03	**Corr:** -0.156 **R2:** 0.024	**Corr:** -0.196 **R2:** 0.039	**Corr:** 0.085 **R2:** 0.007	**Corr:** -0.128 **R2:** 0.016	**Corr:** -0.33 **R2:** 0.109	**Corr:** -0.204 **R2:** 0.042
**Z-Score:** -3.569 **P-Value:** 3.58E-4	**Z-Score:** -3.328 **P-Value:** 8.74E-4	**Z-Score:** -3.558 **P-Value:** 3.74E-4	**Z-Score:** 0.582 **P-Value:** 0.561	**Z-Score:** -0.965 **P-Value:** 0.335	**Z-Score:** -3.533 **P-Value:** 4.12E-4	**Z-Score:** -1.552 **P-Value:** 0.121

SNP rs9268877 (chr. 6, 32539125 bp)
Probe 1090739 (chr. 2, 108570199 - 108671134 bp), LIMS1
P-Value 1.02E-11

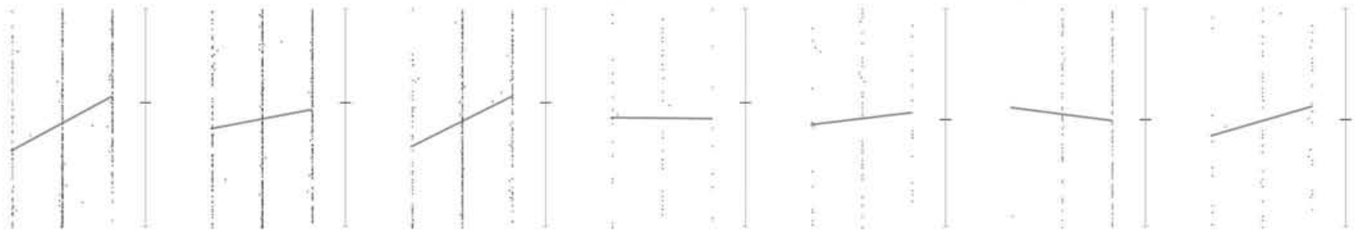

HT12-Controls	HT12-COPD	HT12-ALS	HT12-UC	H8v2-Controls	H8v2-CeliacDisease	H8v2-ALS
AA (61) AG (212) GG (141)	AA (91) AG (214) GG (148)	AA (50) AG (179) GG (95)	AA (16) AG (25) GG (8)	AA (13) AG (32) GG (14)	AA (1) AG (42) GG (68)	AA (10) AG (28) GG (21)
**Corr:** 0.287 **R2:** 0.082	**Corr:** 0.109 **R2:** 0.012	**Corr:** 0.256 **R2:** 0.066	**Corr:** -0.009 **R2:** 0	**Corr:** 0.064 **R2:** 0.004	**Corr:** -0.054 **R2:** 0.003	**Corr:** 0.155 **R2:** 0.024
**Z-Score:** 5.948 **P-Value:** 2.71E-9	**Z-Score:** 2.321 **P-Value:** 0.02	**Z-Score:** 4.668 **P-Value:** 3.04E-6	**Z-Score:** -0.061 **P-Value:** 0.951	**Z-Score:** 0.48 **P-Value:** 0.631	**Z-Score:** -0.563 **P-Value:** 0.573	**Z-Score:** 1.171 **P-Value:** 0.241

SNP rs9272346 (chr. 6, 32712350 bp)
Probe 1090739 (chr. 2, 108570199 - 108671134 bp), LIMS1
P-Value 1.01E-11

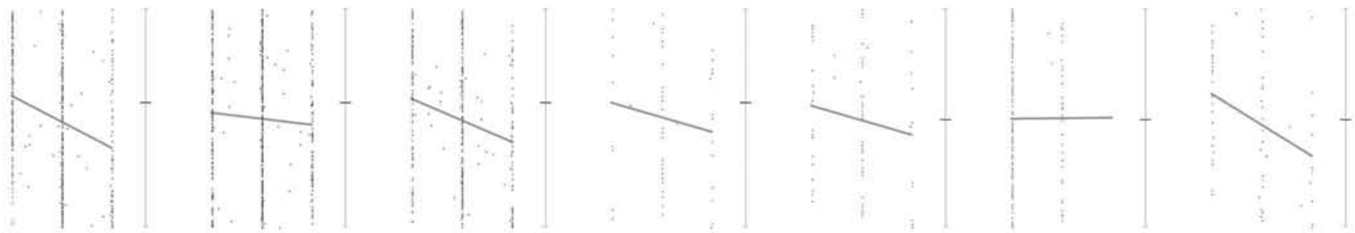

HT12-Controls	HT12-COPD	HT12-ALS	HT12-UC	H8v2-Controls	H8v2-CeliacDisease	H8v2-ALS
AA (130) AG (214) GG (70)	AA (134) AG (235) GG (84)	AA (97) AG (165) GG (62)	AA (10) AG (26) GG (13)	AA (20) AG (28) GG (11)	AA (79) AG (32) GG (0)	AA (24) AG (23) GG (12)
**Corr:** -0.278 **R2:** 0.078	**Corr:** -0.068 **R2:** 0.005	**Corr:** -0.232 **R2:** 0.054	**Corr:** -0.151 **R2:** 0.023	**Corr:** -0.159 **R2:** 0.025	**Corr:** 0.003 **R2:** 0	**Corr:** -0.351 **R2:** 0.123
**Z-Score:** -5.763 **P-Value:** 8.25E-9	**Z-Score:** -1.439 **P-Value:** 0.15	**Z-Score:** -4.225 **P-Value:** 2.39E-5	**Z-Score:** -1.033 **P-Value:** 0.301	**Z-Score:** -1.203 **P-Value:** 0.229	**Z-Score:** 0.028 **P-Value:** 0.978	**Z-Score:** -2.727 **P-Value:** 0.006

SNP rs12485738 (chr. 3, 56840816 bp)
Probe 2230563 (chr. 4, 75070622 - 75073764 bp), PPBP
P-Value 6.43E-12

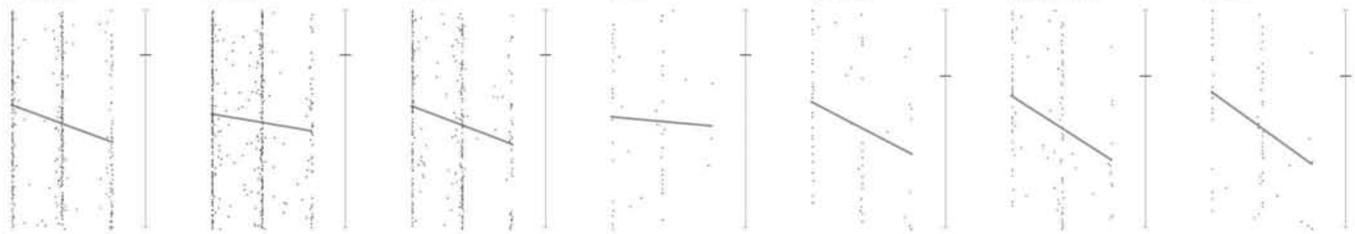

HT12-Controls	HT12-COPD	HT12-ALS	HT12-UC	H8v2-Controls	H8v2-CeliacDisease	H8v2-ALS
GG (153) GA (194) AA (67)	GG (202) GA (200) AA (51)	GG (132) GA (148) AA (44)	GG (24) GA (22) AA (3)	GG (29) GA (21) AA (10)	GG (41) GA (57) AA (13)	GG (20) GA (32) AA (7)
**Corr:** -0.202 **R2:** 0.041	**Corr:** -0.086 **R2:** 0.007	**Corr:** -0.2 **R2:** 0.04	**Corr:** -0.045 **R2:** 0.002	**Corr:** -0.285 **R2:** 0.081	**Corr:** -0.314 **R2:** 0.099	**Corr:** -0.346 **R2:** 0.12
**Z-Score:** -4.15 **P-Value:** 3.32E-5	**Z-Score:** -1.823 **P-Value:** 0.068	**Z-Score:** -3.614 **P-Value:** 3.01E-4	**Z-Score:** -0.306 **P-Value:** 0.76	**Z-Score:** -2.188 **P-Value:** 0.029	**Z-Score:** -3.356 **P-Value:** 7.91E-4	**Z-Score:** -2.688 **P-Value:** 0.007

SNP rs12485738 (chr. 3, 56840816 bp)
Probe 1770170 (chr. 3, 37877673 - 38001964 bp), CTDSPL
P-Value 5.51E-12

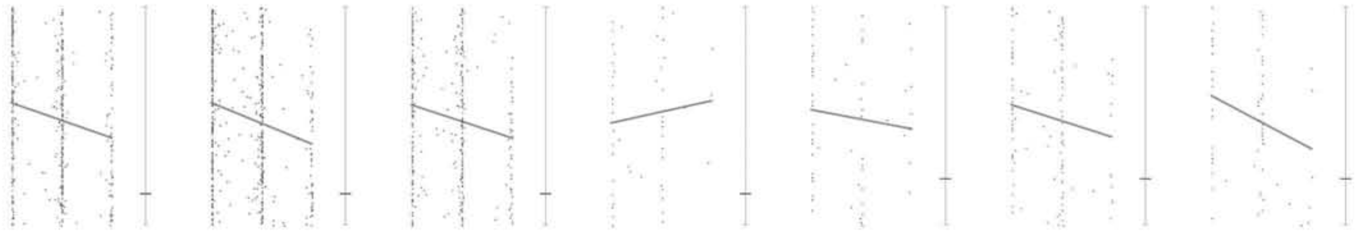

	HT12-Controls	HT12-COPD	HT12-ALS	HT12-UC	H8v2-Controls	H8v2-CeliacDisease	H8v2-ALS
Genotypes	GG (153) GA (194) AA (67)	GG (202) GA (200) AA (51)	GG (132) GA (148) AA (44)	GG (24) GA (22) AA (3)	GG (28) GA (21) AA (10)	GG (41) GA (57) AA (13)	GG (20) GA (32) AA (7)
Corr:	-0.188	-0.211	-0.172	0.099	-0.105	-0.158	-0.251
R2:	0.035	0.045	0.029	0.01	0.011	0.025	0.063
Z-Score:	-3.842	-4.534	-3.101	0.679	-0.789	-1.658	-1.916
P-Value:	1.22E-4	5.79E-6	0.002	0.497	0.43	0.097	0.055

SNP rs2227139 (chr. 6, 32521437 bp)
Probe 2940424 (chr. 7, 36518135 - 36731595 bp), AOAH
P-Value 5.08E-12

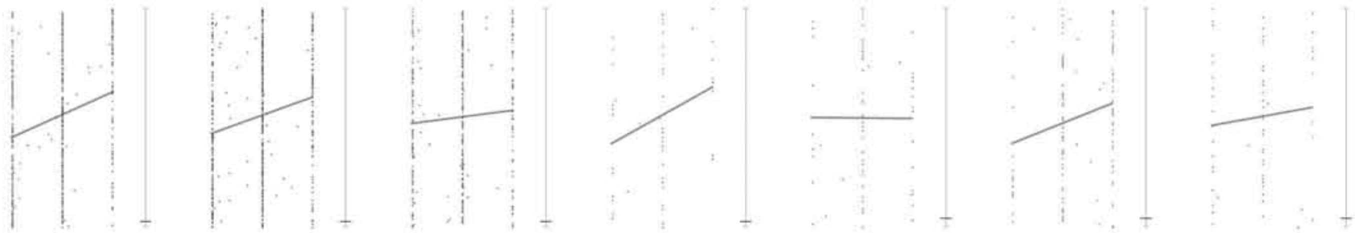

	HT12-Controls	HT12-COPD	HT12-ALS	HT12-UC	H8v2-Controls	H8v2-CeliacDisease	H8v2-ALS
Genotypes	AA (133) AG (196) GG (85)	AA (145) AG (217) GG (91)	AA (106) AG (154) GG (64)	AA (14) AG (25) GG (10)	AA (11) AG (33) GG (15)	AA (16) AG (49) GG (46)	AA (19) AG (29) GG (11)
Corr:	0.254	0.2	0.072	0.291	-0.004	0.216	0.095
R2:	0.065	0.04	0.005	0.085	0	0.047	0.009
Z-Score:	5.247	4.295	1.286	2.03	-0.027	2.279	0.717
P-Value:	1.55E-7	1.75E-5	0.198	0.042	0.978	0.023	0.473

SNP rs3134792 (chr. 6, 31420305 bp)
Probe 1110300 (chr. 4, 153765727 - 153821641 bp), TMEM154
P-Value 1.68E-12

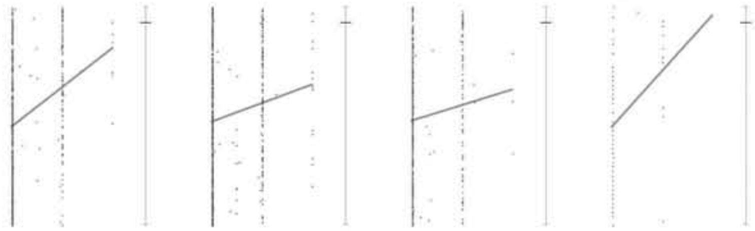

	HT12-Controls	HT12-COPD	HT12-ALS	HT12-UC	H8v2-Controls	H8v2-CeliacDisease	H8v2-ALS
Genotypes	TT (309) TG (98) GG (7)	TT (325) TG (115) GG (13)	TT (245) TG (76) GG (3)	TT (39) TG (10) GG (0)	eQTL not available	eQTL not available	eQTL not available
Corr:	0.299	0.153	0.112	0.335	Probe not present.	Probe not present.	Probe not present.
R2:	0.089	0.023	0.012	0.112			
Z-Score:	6.208	3.264	2.007	2.353			
P-Value:	5.36E-10	0.001	0.045	0.019			

SNP rs12485738 (chr. 3, 56840816 bp)
Probe 6040259 (chr. 17, 6848959 - 6856128 bp), ALOX12///AC027763.2
P-Value 1.37E-12

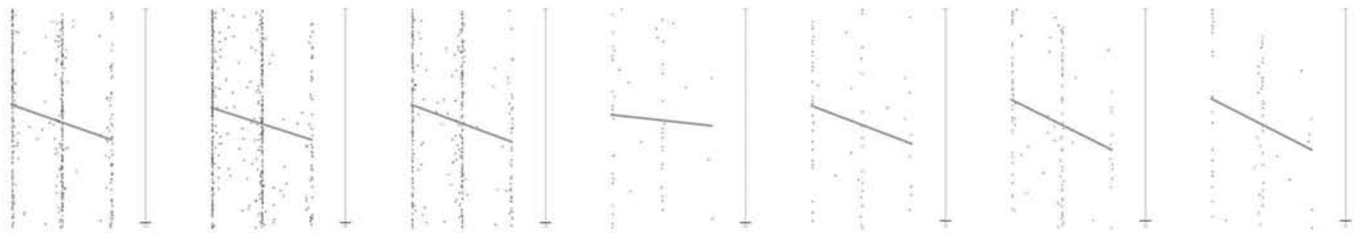

	HT12-Controls	HT12-COPD	HT12-ALS	HT12-UC	H8v2-Controls	H8v2-CeliacDisease	H8v2-ALS
Genotypes	GG (153) GA (194) AA (67)	GG (202) GA (200) AA (51)	GG (132) GA (148) AA (44)	GG (24) GA (22) AA (3)	GG (28) GA (21) AA (10)	GG (41) GA (57) AA (13)	GG (20) GA (32) AA (7)
Corr:	-0.189	-0.163	-0.192	-0.052	-0.209	-0.244	-0.245
R2:	0.036	0.027	0.037	0.003	0.044	0.059	0.06
Z-Score:	-3.879	-3.487	-3.474	-0.355	-1.589	-2.58	-1.872
P-Value:	1.05E-4	4.88E-4	5.13E-4	0.723	0.112	0.01	0.061

SNP rs2227139 (chr. 6, 32521437 bp)
Probe 2570703 (chr. 21, 38672819 - 38956488 bp), ERG
P-Value 1.08E-12

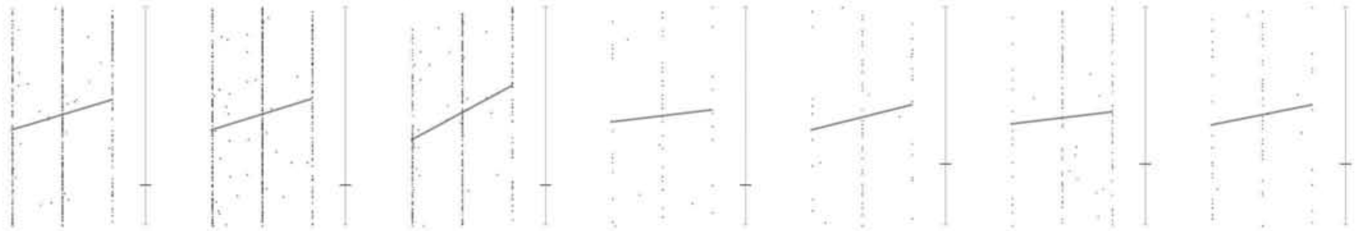

	HT12-Controls	HT12-COPD	HT12-ALS	HT12-UC	H8v2-Controls	H8v2-CeliacDisease	H8v2-ALS
Genotypes	AA (133) AG (196) GG (85)	AA (145) AG (217) GG (91)	AA (106) AG (154) GG (64)	AA (14) AG (25) GG (10)	AA (11) AG (33) GG (15)	AA (16) AG (49) GG (46)	AA (19) AG (29) GG (11)
Corr:	0.167	0.175	0.306	0.059	0.127	0.059	0.103
R2:	0.028	0.031	0.094	0.004	0.016	0.004	0.011
Z-Score:	3.402	3.75	5.631	0.405	0.961	0.619	0.779
P-Value:	6.69E-4	1.77E-4	1.8E-8	0.685	0.336	0.536	0.436

SNP rs12485738 (chr. 3, 56840816 bp)
Probe 7570196 (chr. 12, 3055818 - 3266990 bp), TSPAN9
P-Value 3.89E-13

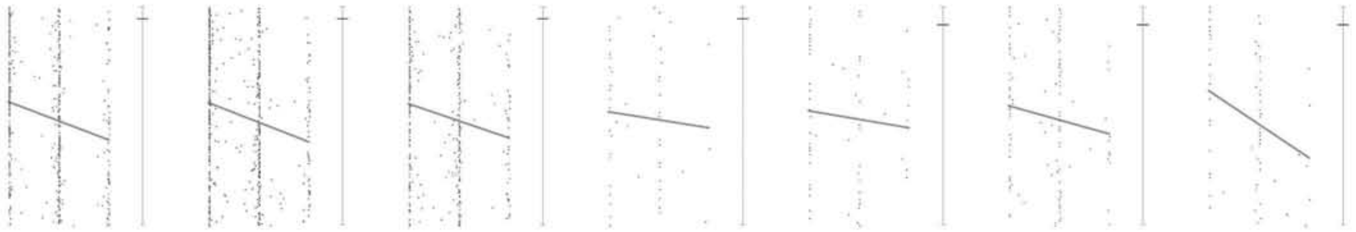

	HT12-Controls	HT12-COPD	HT12-ALS	HT12-UC	H8v2-Controls	H8v2-CeliacDisease	H8v2-ALS
Genotypes	GG (153) GA (194) AA (67)	GG (202) GA (200) AA (51)	GG (132) GA (148) AA (44)	GG (24) GA (22) AA (3)	GG (28) GA (21) AA (10)	GG (41) GA (57) AA (13)	GG (20) GA (32) AA (7)
Corr:	-0.205	-0.201	-0.176	-0.077	-0.097	-0.139	-0.322
R2:	0.042	0.04	0.031	0.006	0.009	0.019	0.104
Z-Score:	-4.197	-4.308	-3.178	-0.523	-0.729	-1.46	-2.485
P-Value:	2.71E-5	1.65E-5	0.001	0.601	0.466	0.144	0.013

SNP rs4963128 (chr. 11, 579564 bp)
Probe 2000670 (chr. 21, 43452479 - 43452479 bp), -
P-Value 3.83E-13

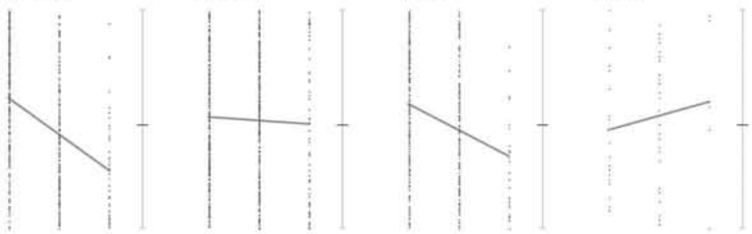

	HT12-Controls	HT12-COPD	HT12-ALS	HT12-UC	H8v2-Controls	H8v2-CeliacDisease	H8v2-ALS
Genotypes	CC (207) CT (166) TT (41)	CC (200) CT (199) TT (54)	CC (162) CT (135) TT (27)	CC (18) CT (25) TT (6)	**eQTL not available** Probe not present.	**eQTL not available** Probe not present.	**eQTL not available** Probe not present.
Corr:	-0.376	-0.033	-0.263	0.141			
R2:	0.141	0.001	0.069	0.02			
Z-Score:	-7.913	-0.708	-4.809	0.965			
P-Value:	2.51E-15	0.479	1.51E-6	0.334			

SNP rs12485738 (chr. 3, 56840816 bp)
Probe 130433 (chr. 11, 69959459 - 69960075 bp), CTTN
P-Value 3.31E-13

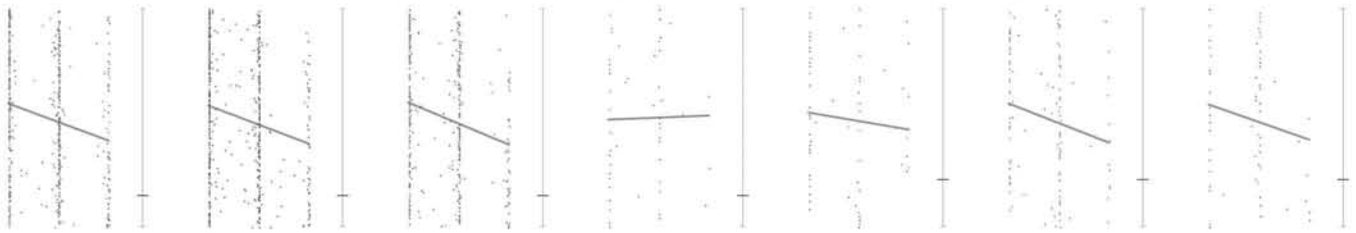

	HT12-Controls	HT12-COPD	HT12-ALS	HT12-UC	H8v2-Controls	H8v2-CeliacDisease	H8v2-ALS
Genotypes	GG (153) GA (194) AA (67)	GG (202) GA (200) AA (51)	GG (132) GA (148) AA (44)	GG (24) GA (22) AA (3)	GG (28) GA (21) AA (10)	GG (41) GA (57) AA (13)	GG (20) GA (32) AA (7)
Corr:	-0.2	-0.196	-0.217	0.016	-0.095	-0.188	-0.169
R2:	0.04	0.038	0.047	0	0.009	0.035	0.029
Z-Score:	-4.093	-4.193	-3.943	0.111	-0.718	-1.975	-1.279
P-Value:	4.26E-5	2.75E-5	8.03E-5	0.911	0.473	0.048	0.201

SNP rs12485738 (chr. 3, 56840816 bp)
Probe 50136 (chr. 14, 22914857 - 22919820 bp), CMTM5
P-Value 3.03E-13

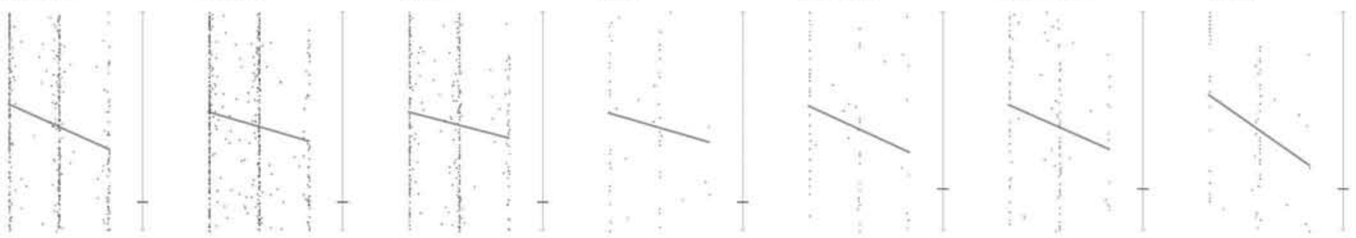

	HT12-Controls	HT12-COPD	HT12-ALS	HT12-UC	H8v2-Controls	H8v2-CeliacDisease	H8v2-ALS
Genotypes	GG (153) GA (194) AA (67)	GG (202) GA (200) AA (51)	GG (132) GA (148) AA (44)	GG (24) GA (22) AA (3)	GG (28) GA (21) AA (10)	GG (41) GA (57) AA (13)	GG (20) GA (32) AA (7)
Corr:	-0.243	-0.152	-0.136	-0.133	-0.25	-0.214	-0.332
R2:	0.059	0.023	0.019	0.018	0.062	0.046	0.11
Z-Score:	-5.002	-3.238	-2.458	-0.912	-1.907	-2.252	-2.567
P-Value:	5.69E-7	0.001	0.014	0.362	0.057	0.024	0.01

SNP rs12485738 (chr. 3, 56840816 bp)
Probe 1400689 (chr. 11, 133484788 - 133564241 bp), JAM3///NCAPD3
P-Value 2.2E-13

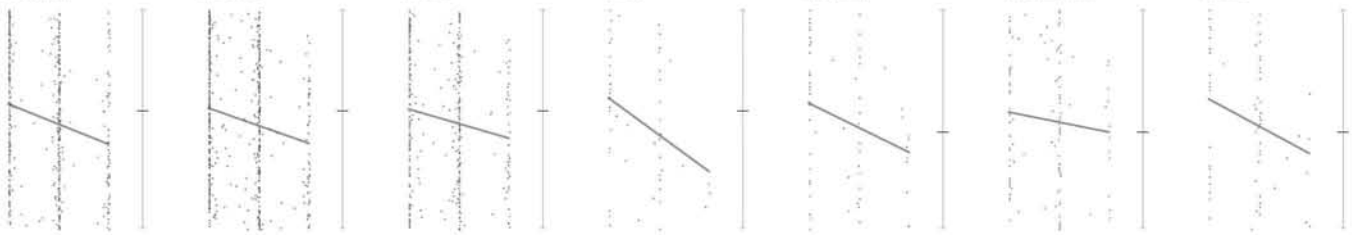

	HT12-Controls	HT12-COPD	HT12-ALS	HT12-UC	H8v2-Controls	H8v2-CeliacDisease	H8v2-ALS
Genotypes	GG (153) GA (194) AA (67)	GG (202) GA (200) AA (51)	GG (132) GA (148) AA (44)	GG (24) GA (22) AA (3)	GG (28) GA (21) AA (10)	GG (41) GA (57) AA (13)	GG (20) GA (32) AA (7)
Corr:	-0.221	-0.181	-0.149	-0.336	-0.272	-0.095	-0.256
R2:	0.049	0.033	0.022	0.113	0.074	0.009	0.065
Z-Score:	-4.536	-3.866	-2.679	-2.361	-2.084	-0.996	-1.954
P-Value:	5.74E-6	1.11E-4	0.007	0.018	0.037	0.319	0.051

SNP rs9483788 (chr. 6, 135477194 bp)
Probe 3130220 (chr. 3, 45240621 - 45243523 bp), AC010170.3
P-Value 7.83E-14

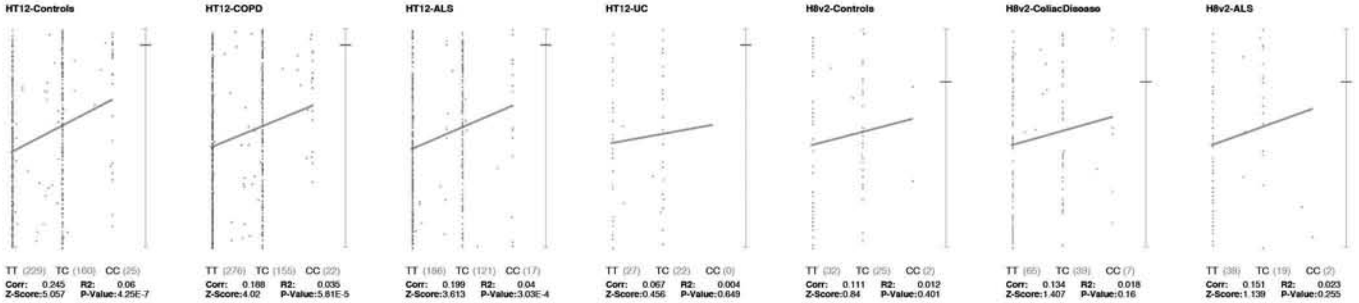

SNP rs2540917 (chr. 2, 60462263 bp)
Probe 3440630 (chr. 1, 6424616 - 6447223 bp), ESPN///TNFRSF25
P-Value 4.78E-14

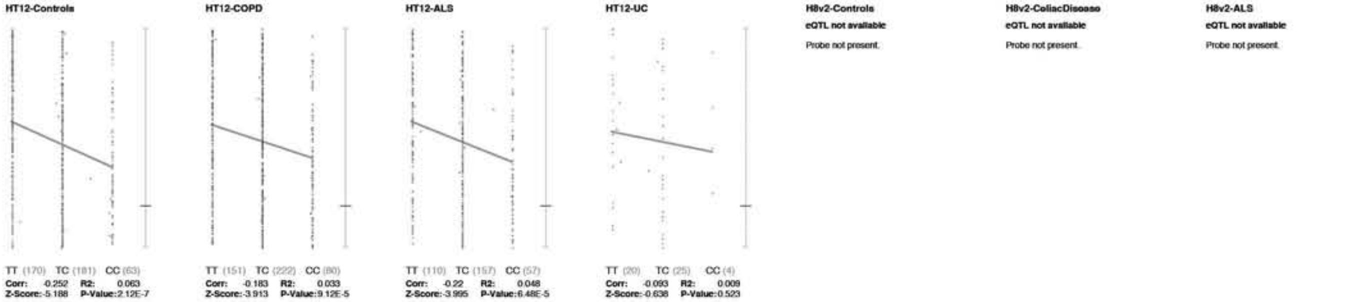

SNP rs7743761 (chr. 6, 31444079 bp)
Probe 1110300 (chr. 4, 153765727 - 153821641 bp), TMEM154
P-Value 9.26E-15

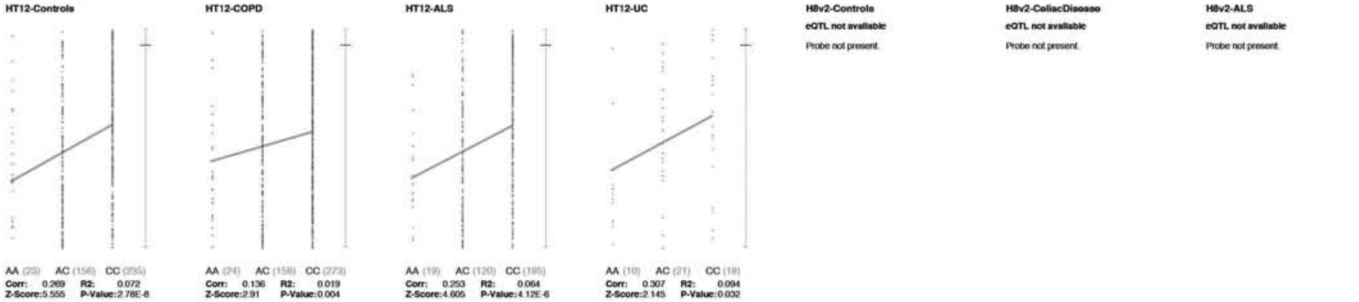

SNP rs3134792 (chr. 6, 31420305 bp)
Probe 6380347 (chr. 22, 40346241 - 40390998 bp), XRCC6
P-Value 1.4E-15

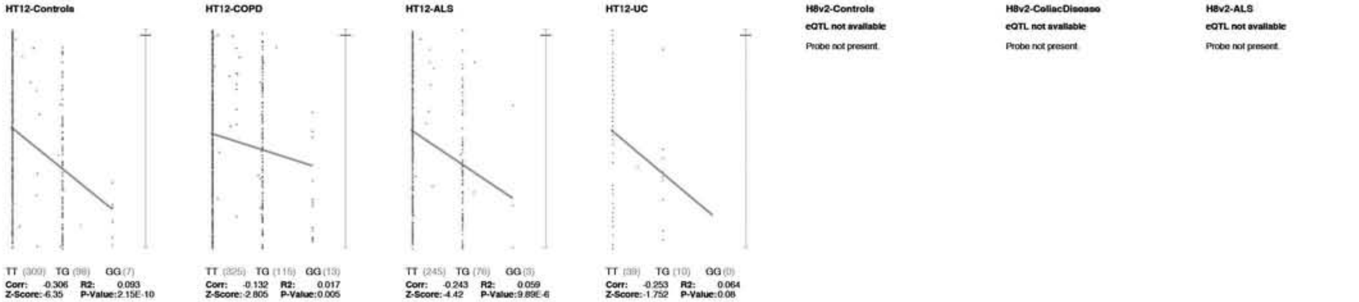

SNP rs2856683 (chr. 6, 32763196 bp)
Probe 2940424 (chr. 7, 36518135 - 36731595 bp), AOAH
P-Value 7.35E-16

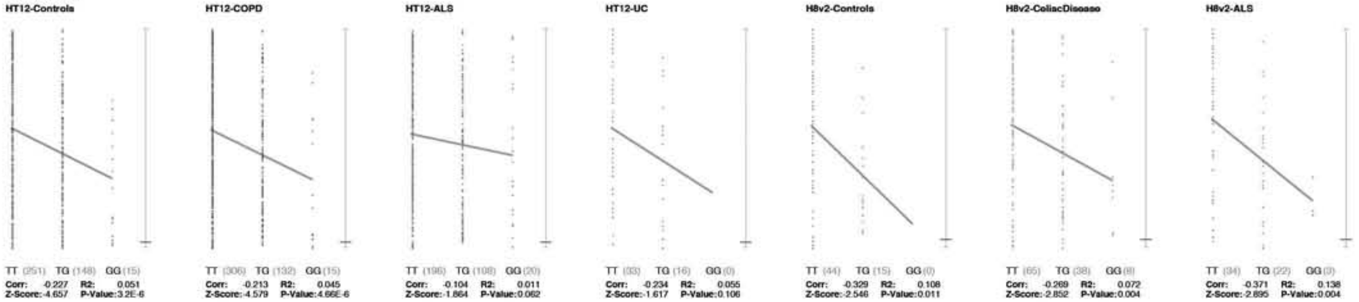

SNP rs12718597 (chr. 7, 50395922 bp)
Probe 3170017 (chr. 17, 21127561 - 21160118 bp), MAP2K3
P-Value 1.04E-16

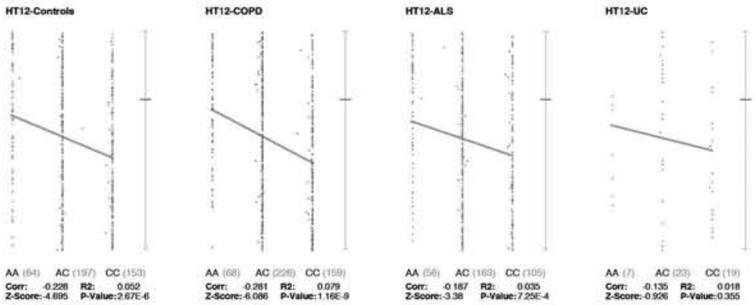

H8v2-Controls
eQTL not available
Probe not present.

H8v2-CeliacDisease
eQTL not available
Probe not present.

H8v2-ALS
eQTL not available
Probe not present.

SNP rs12485738 (chr. 3, 56840816 bp)
Probe 1050292 (chr. 3, 130261335 - 130264935 bp), GP9
P-Value 9.97E-17

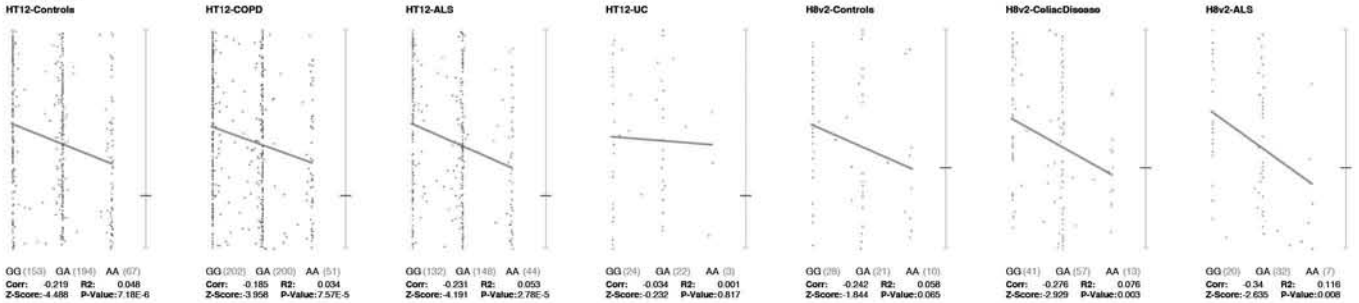

SNP rs12718597 (chr. 7, 50395922 bp)
Probe 4010433 (chr. 17, 21127561 - 21160118 bp), MAP2K3
P-Value 4.72E-17

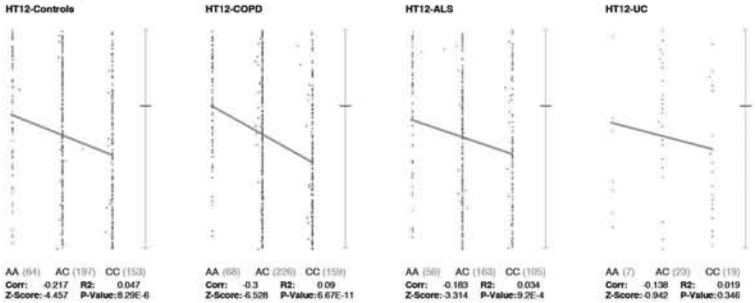

H8v2-Controls
eQTL not available
Probe not present.

H8v2-CeliacDisease
eQTL not available
Probe not present.

H8v2-ALS
eQTL not available
Probe not present.

SNP rs9402686 (chr. 6, 135469510 bp)
Probe 3130220 (chr. 3, 45240621 - 45243523 bp), AC010170.3
P-Value 3.83E-17

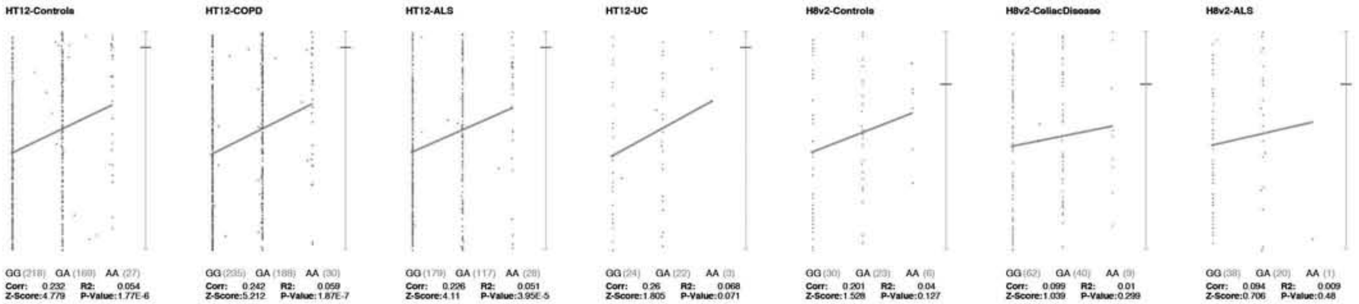

SNP rs4963128 (chr. 11, 579564 bp)
Probe 150328 (chr. 1, 14015613 - 14022280 bp), PRDM2
P-Value 1.9E-17

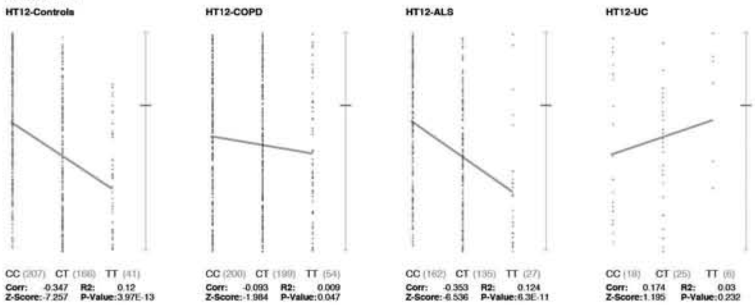

H8v2-Controls
eQTL not available
Probe not present.

H8v2-CeliacDisease
eQTL not available
Probe not present.

H8v2-ALS
eQTL not available
Probe not present.

SNP rs9373124 (chr. 6, 135464902 bp)
Probe 3130220 (chr. 3, 45240621 - 45243523 bp), AC010170.3
P-Value 1.71E-17

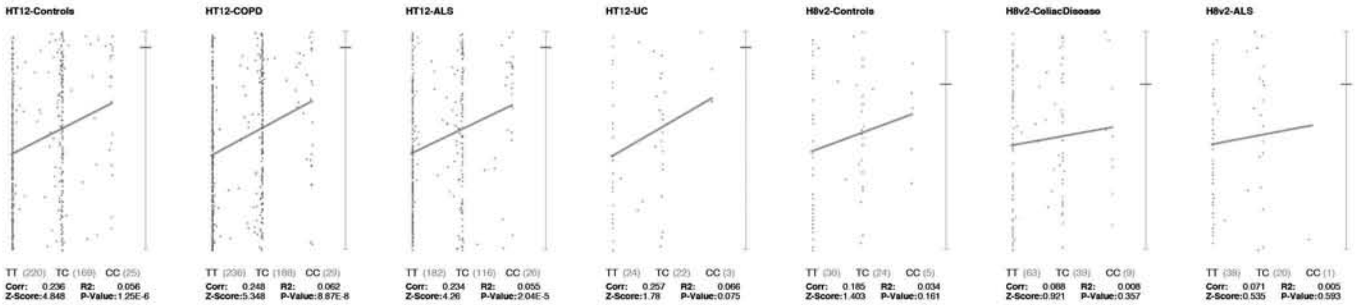

SNP rs9376092 (chr. 6, 135468837 bp)
Probe 3130220 (chr. 3, 45240621 - 45243523 bp), AC010170.3
P-Value 1.55E-17

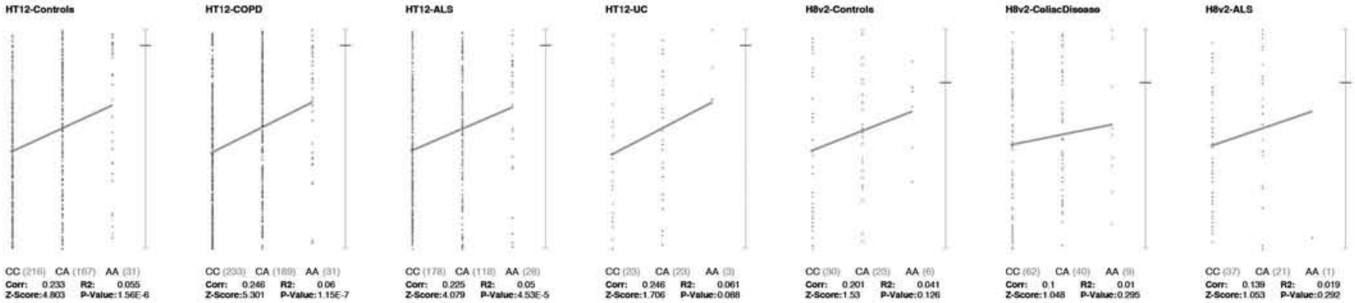

SNP rs4895441 (chr. 6, 135468266 bp)
Probe 3130220 (chr. 3, 45240621 - 45243523 bp), AC010170.3
P-Value 1.12E-17

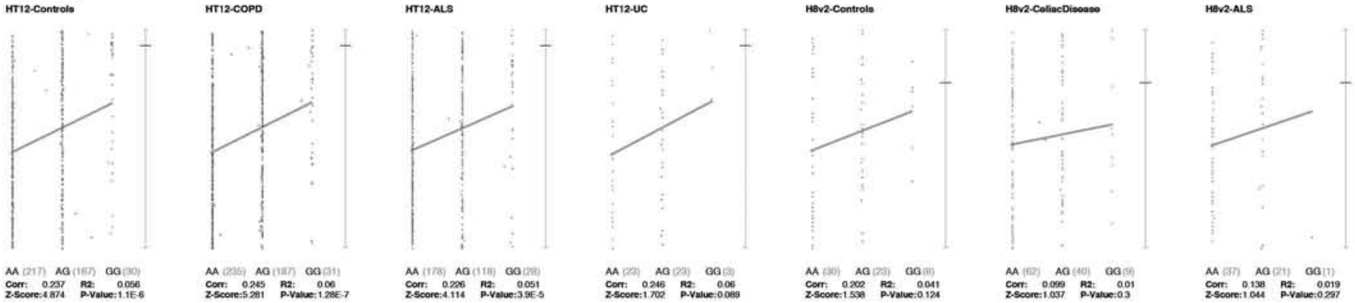

SNP rs7776054 (chr. 6, 135460609 bp)
Probe 3130220 (chr. 3, 45240621 - 45243523 bp), AC010170.3
P-Value 9.07E-18

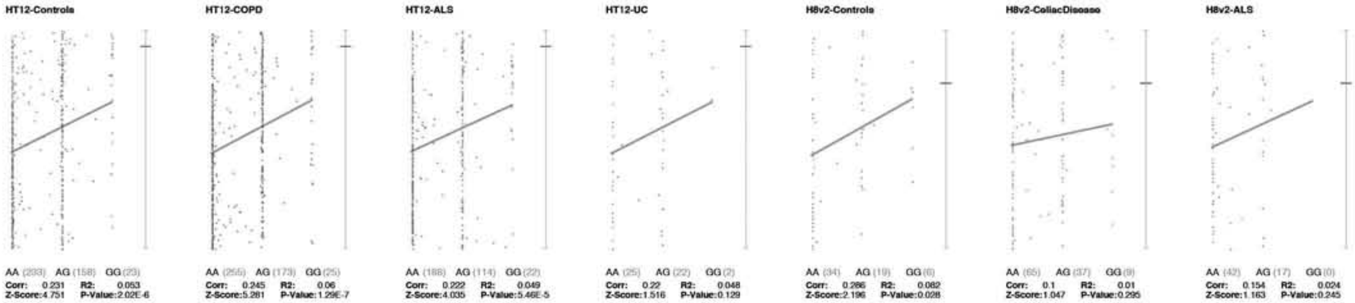

SNP rs9399137 (chr. 6, 135460711 bp)
Probe 3130220 (chr. 3, 45240621 - 45243523 bp), AC010170.3
P-Value 8.94E-18

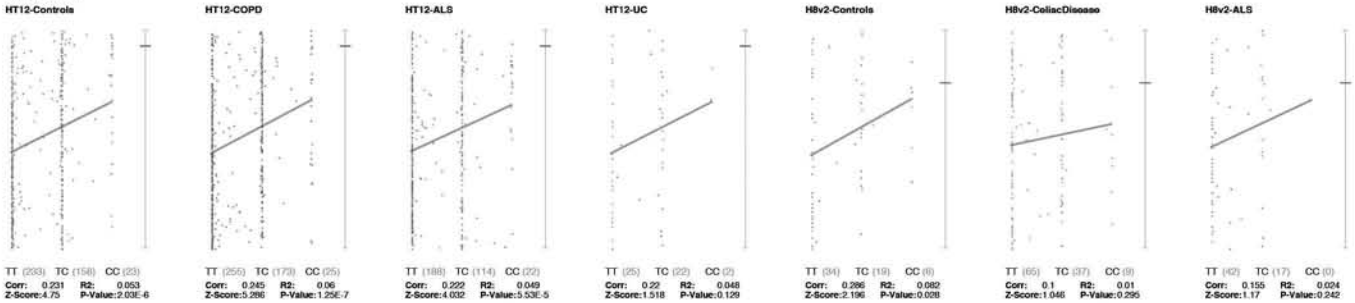

SNP rs2540917 (chr. 2, 60462263 bp)
Probe 450537 (chr. 11, 5229996 - 5429091 bp), HBG2///ENST00000399563
P-Value 7.04E-18

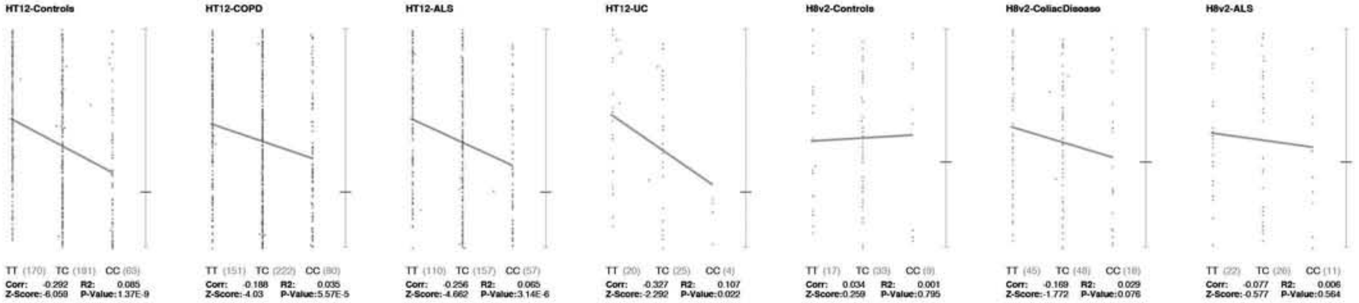

SNP rs12718597 (chr. 7, 50395922 bp)
Probe 3290386 (chr. 11, 64452638 - 64460229 bp), PPP2R5B///GPHA2
P-Value 6.67E-18

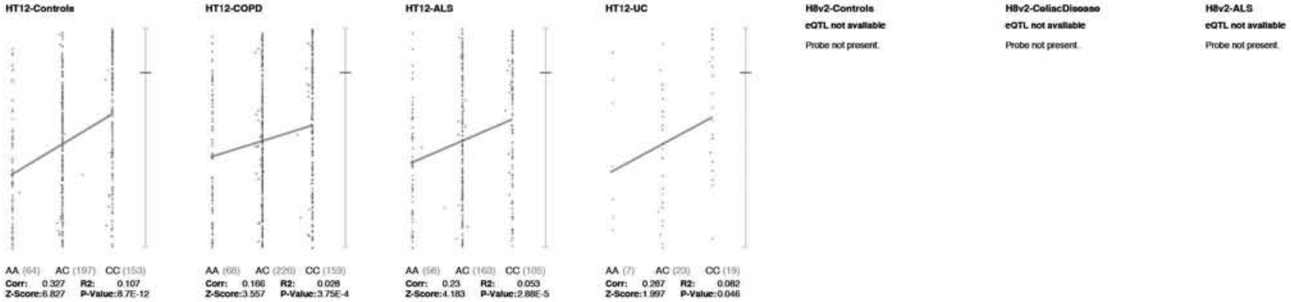

SNP rs12485738 (chr. 3, 56840816 bp)
Probe 5560280 (chr. 17, 45542679 - 45563176 bp), SAMD14
P-Value 3.74E-18

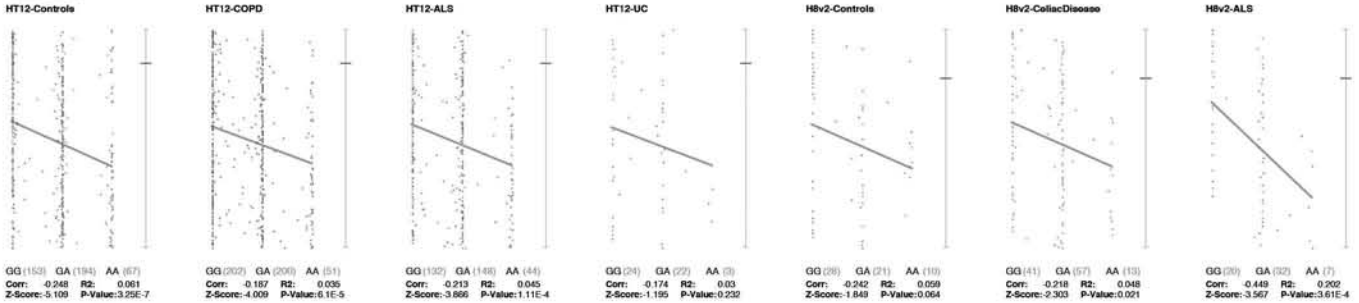

SNP rs2517713 (chr. 6, 30026078 bp)
Probe 5080687 (chr. 2, 206695080 - 206733432 bp), NDUFS1
P-Value 2.79E-18

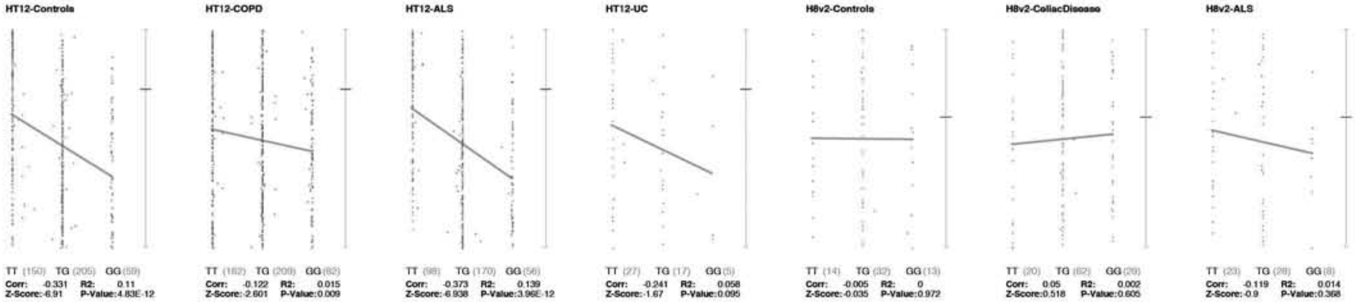

SNP rs12485738 (chr. 3, 56840816 bp)
Probe 7200044 (chr. 6, 80424595 - 80533976 bp), SH3BGRL2///C6orf152
P-Value 2.39E-18

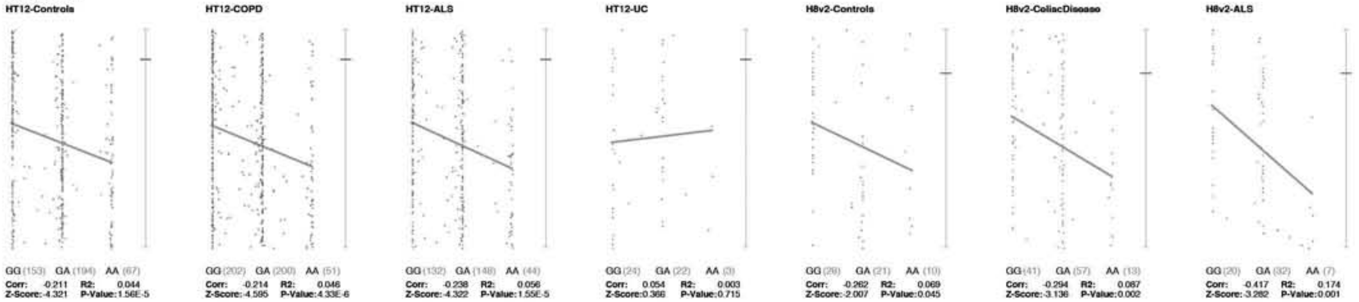

SNP rs9483786 (chr. 6, 135477194 bp)
Probe 4010040 (chr. 11, 5229996 - 5429091 bp), HBG2///ENST00000399563
P-Value 1.49E-18

HT12-Controls | **HT12-COPD** | **HT12-ALS** | **HT12-UC**

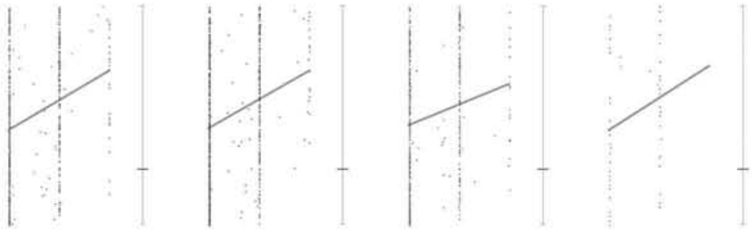

HT12-Controls	HT12-COPD	HT12-ALS	HT12-UC
TT (229) TC (160) CC (25)	TT (276) TC (155) CC (22)	TT (186) TC (121) CC (17)	TT (27) TC (22) CC (0)
**Corr:** 0.28 **R2:** 0.078	**Corr:** 0.256 **R2:** 0.066	**Corr:** 0.191 **R2:** 0.036	**Corr:** 0.243 **R2:** 0.059
**Z-Score:**5.791 **P-Value:**6.99E-9	**Z-Score:**5.53 **P-Value:**3.2E-8	**Z-Score:**3.457 **P-Value:**5.46E-4	**Z-Score:**1.681 **P-Value:**0.093

H8v2-Controls
eQTL not available
Probe not present.

H8v2-CeliacDisease
eQTL not available
Probe not present.

H8v2-ALS
eQTL not available
Probe not present.

SNP rs12485738 (chr. 3, 56840816 bp)
Probe 3850630 (chr. 15, 81314970 - 81314970 bp), -
P-Value 1.19E-18

HT12-Controls | **HT12-COPD** | **HT12-ALS** | **HT12-UC** | **H8v2-Controls** | **H8v2-CeliacDisease** | **H8v2-ALS**

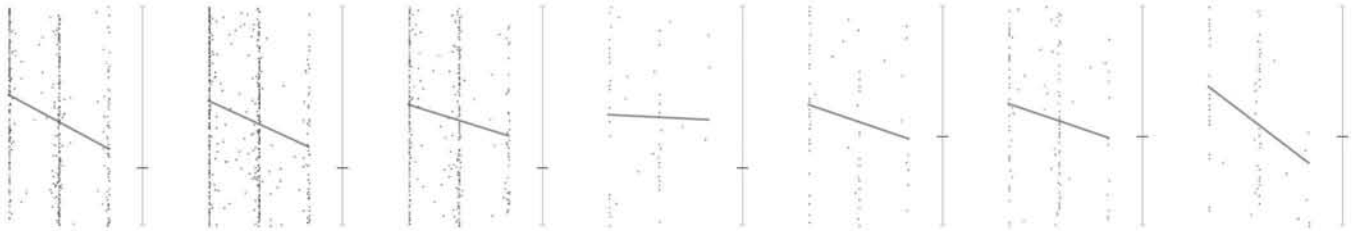

HT12-Controls	HT12-COPD	HT12-ALS	HT12-UC	H8v2-Controls	H8v2-CeliacDisease	H8v2-ALS
GG (153) GA (194) AA (67)	GG (202) GA (200) AA (51)	GG (132) GA (148) AA (44)	GG (24) GA (22) AA (3)	GG (28) GA (21) AA (10)	GG (41) GA (57) AA (13)	GG (20) GA (32) AA (7)
**Corr:** -0.293 **R2:** 0.086	**Corr:** -0.239 **R2:** 0.057	**Corr:** -0.162 **R2:** 0.026	**Corr:** -0.024 **R2:** 0.001	**Corr:** -0.188 **R2:** 0.035	**Corr:** -0.166 **R2:** 0.028	**Corr:** -0.361 **R2:** 0.13
**Z-Score:**-6.078 **P-Value:**1.22E-9	**Z-Score:**-5.157 **P-Value:**2.52E-7	**Z-Score:**-2.927 **P-Value:**0.003	**Z-Score:**-0.167 **P-Value:**0.867	**Z-Score:**-1.426 **P-Value:**0.154	**Z-Score:**-1.746 **P-Value:**0.081	**Z-Score:**-2.811 **P-Value:**0.005

SNP rs2540917 (chr. 2, 60462263 bp)
Probe 4010040 (chr. 11, 5229996 - 5429091 bp), HBG2///ENST00000399563
P-Value 1.17E-18

HT12-Controls | **HT12-COPD** | **HT12-ALS** | **HT12-UC**

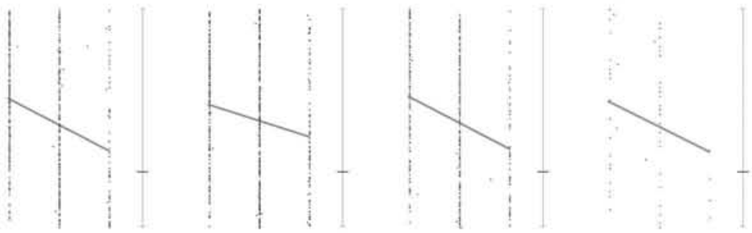

HT12-Controls	HT12-COPD	HT12-ALS	HT12-UC
TT (170) TC (181) CC (63)	TT (151) TC (222) CC (80)	TT (110) TC (157) CC (57)	TT (20) TC (25) CC (4)
**Corr:** -0.289 **R2:** 0.083	**Corr:** -0.181 **R2:** 0.033	**Corr:** -0.287 **R2:** 0.082	**Corr:** -0.238 **R2:** 0.057
**Z-Score:**-5.981 **P-Value:**2.21E-9	**Z-Score:**-3.865 **P-Value:**1.11E-4	**Z-Score:**-5.254 **P-Value:**1.49E-7	**Z-Score:**-1.648 **P-Value:**0.099

H8v2-Controls
eQTL not available
Probe not present.

H8v2-CeliacDisease
eQTL not available
Probe not present.

H8v2-ALS
eQTL not available
Probe not present.

SNP rs2517713 (chr. 6, 30026078 bp)
Probe 5080577 (chr. 20, 61958531 - 61994332 bp), TPD52L2
P-Value 2.03E-19

HT12-Controls | **HT12-COPD** | **HT12-ALS** | **HT12-UC**

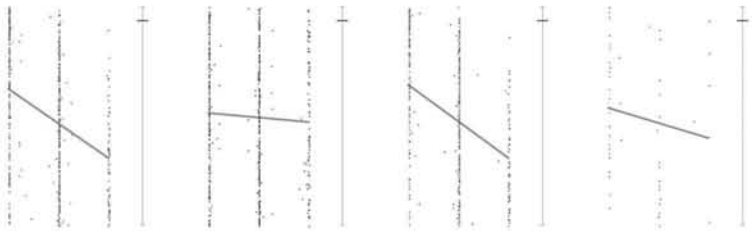

HT12-Controls	HT12-COPD	HT12-ALS	HT12-UC
TT (150) TG (205) GG (59)	TT (162) TG (209) GG (82)	TT (98) TG (170) GG (56)	TT (27) TG (17) GG (5)
**Corr:** -0.363 **R2:** 0.132	**Corr:** -0.054 **R2:** 0.003	**Corr:** -0.388 **R2:** 0.151	**Corr:** -0.154 **R2:** 0.024
**Z-Score:**-7.632 **P-Value:**2.31E-14	**Z-Score:**-1.14 **P-Value:**0.254	**Z-Score:**-7.244 **P-Value:**4.34E-13	**Z-Score:**-1.055 **P-Value:**0.292

H8v2-Controls
eQTL not available
Probe not present.

H8v2-CeliacDisease
eQTL not available
Probe not present.

H8v2-ALS
eQTL not available
Probe not present.

SNP rs4963128 (chr. 11, 579564 bp)
Probe 7650681 (chr. 1, 14015613 - 14022280 bp), PRDM2
P-Value 2.25E-20

HT12-Controls | **HT12-COPD** | **HT12-ALS** | **HT12-UC**

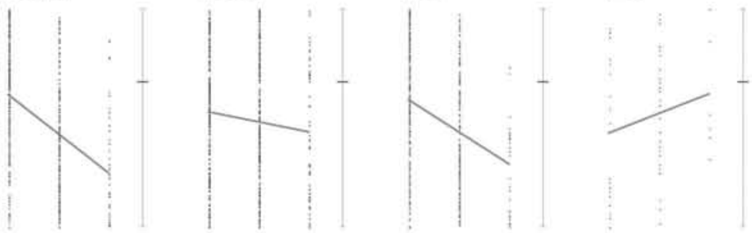

HT12-Controls	HT12-COPD	HT12-ALS	HT12-UC
CC (207) CT (166) TT (41)	CC (200) CT (199) TT (54)	CC (162) CT (135) TT (27)	CC (18) CT (25) TT (6)
**Corr:** -0.411 **R2:** 0.169	**Corr:** -0.107 **R2:** 0.011	**Corr:** -0.329 **R2:** 0.108	**Corr:** 0.198 **R2:** 0.039
**Z-Score:**-8.72 **P-Value:**2.78E-18	**Z-Score:**-2.28 **P-Value:**0.023	**Z-Score:**-6.07 **P-Value:**1.28E-9	**Z-Score:**1.364 **P-Value:**0.172

H8v2-Controls
eQTL not available
Probe not present.

H8v2-CeliacDisease
eQTL not available
Probe not present.

H8v2-ALS
eQTL not available
Probe not present.

SNP rs2523393 (chr. 6, 29813638 bp)
Probe 5080577 (chr. 20, 61958531 - 61994332 bp), TPD52L2
P-Value 6.89E-21

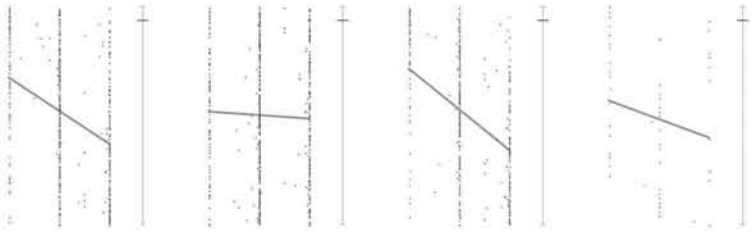

HT12-Controls
GG (72) GA (196) AA (146)
Corr: -0.362 **R2:** 0.131
Z-Score:-7.61 **P-Value:**2.74E-14

HT12-COPD
GG (75) GA (221) AA (157)
Corr: -0.039 **R2:** 0.002
Z-Score:-0.827 **P-Value:**0.408

HT12-ALS
GG (53) GA (158) AA (113)
Corr: -0.434 **R2:** 0.188
Z-Score:-8.192 **P-Value:**2.57E-16

HT12-UC
GG (18) GA (20) AA (11)
Corr: -0.215 **R2:** 0.046
Z-Score:-1.487 **P-Value:**0.137

H8v2-Controls
eQTL not available
Probe not present.

H8v2-CeliacDisease
eQTL not available
Probe not present.

H8v2-ALS
eQTL not available
Probe not present.

SNP rs2523393 (chr. 6, 29813638 bp)
Probe 5080576 (chr. 4, 55217852 - 55302636 bp), KIT
P-Value 4.99E-22

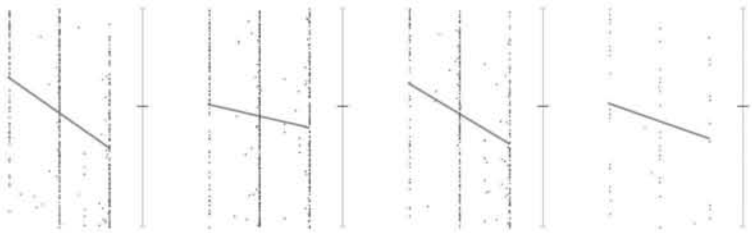

HT12-Controls
GG (72) GA (196) AA (146)
Corr: -0.384 **R2:** 0.148
Z-Score:-8.109 **P-Value:**5.12E-16

HT12-COPD
GG (75) GA (221) AA (157)
Corr: -0.13 **R2:** 0.017
Z-Score:-2.776 **P-Value:**0.005

HT12-ALS
GG (53) GA (158) AA (113)
Corr: -0.32 **R2:** 0.102
Z-Score:-5.886 **P-Value:**3.95E-9

HT12-UC
GG (18) GA (20) AA (11)
Corr: -0.204 **R2:** 0.042
Z-Score:-1.406 **P-Value:**0.16

H8v2-Controls
eQTL not available
Probe not present.

H8v2-CeliacDisease
eQTL not available
Probe not present.

H8v2-ALS
eQTL not available
Probe not present.

SNP rs2517713 (chr. 6, 30026078 bp)
Probe 5080576 (chr. 4, 55217852 - 55302636 bp), KIT
P-Value 2.63E-22

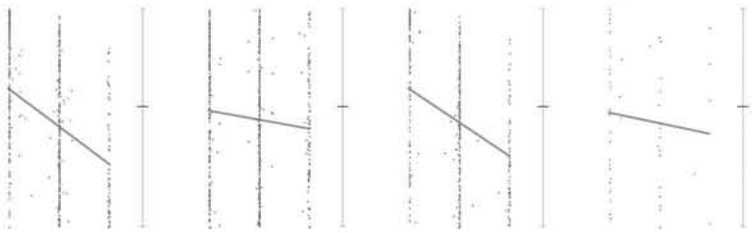

HT12-Controls
TT (150) TG (205) GG (59)
Corr: -0.402 **R2:** 0.162
Z-Score:-8.525 **P-Value:**1.53E-17

HT12-COPD
TT (162) TG (209) GG (82)
Corr: -0.1 **R2:** 0.01
Z-Score:-2.131 **P-Value:**0.033

HT12-ALS
TT (98) TG (170) GG (56)
Corr: -0.355 **R2:** 0.126
Z-Score:-6.587 **P-Value:**4.5E-11

HT12-UC
TT (27) TG (17) GG (5)
Corr: -0.105 **R2:** 0.011
Z-Score:-0.72 **P-Value:**0.472

H8v2-Controls
eQTL not available
Probe not present.

H8v2-CeliacDisease
eQTL not available
Probe not present.

H8v2-ALS
eQTL not available
Probe not present.

SNP rs9483788 (chr. 6, 135477194 bp)
Probe 450537 (chr. 11, 5229996 - 5429091 bp), HBG2///ENST00000399563
P-Value 1.36E-22

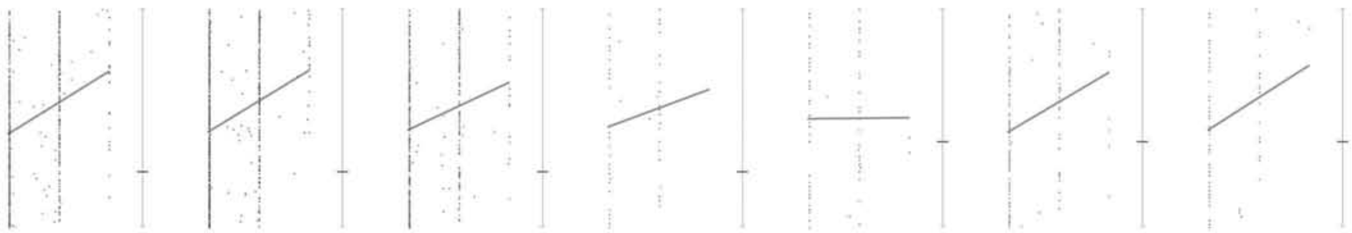

HT12-Controls
TT (229) TC (160) CC (25)
Corr: 0.293 **R2:** 0.086
Z-Score:6.078 **P-Value:**1.22E-9

HT12-COPD
TT (276) TC (155) CC (22)
Corr: 0.275 **R2:** 0.075
Z-Score:5.945 **P-Value:**2.76E-9

HT12-ALS
TT (186) TC (121) CC (17)
Corr: 0.218 **R2:** 0.048
Z-Score:3.957 **P-Value:**7.58E-5

HT12-UC
TT (27) TC (22) CC (0)
Corr: 0.142 **R2:** 0.02
Z-Score:0.972 **P-Value:**0.331

H8v2-Controls
TT (32) TC (25) CC (2)
Corr: 0.003 **R2:** 0
Z-Score:0.025 **P-Value:**0.98

H8v2-CeliacDisease
TT (65) TC (39) CC (7)
Corr: 0.276 **R2:** 0.076
Z-Score:2.934 **P-Value:**0.003

H8v2-ALS
TT (38) TC (19) CC (2)
Corr: 0.263 **R2:** 0.069
Z-Score:2.016 **P-Value:**0.044

SNP rs1427407 (chr. 2, 60571547 bp)
Probe 3440630 (chr. 1, 6424616 - 6447223 bp), ESPN///TNFRSF25
P-Value 2.91E-23

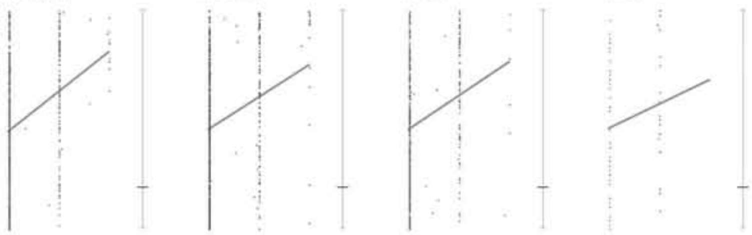

HT12-Controls
GG (301) GT (101) TT (12)
Corr: 0.321 **R2:** 0.103
Z-Score:6.69 **P-Value:**2.23E-11

HT12-COPD
GG (336) GT (102) TT (15)
Corr: 0.26 **R2:** 0.067
Z-Score:5.605 **P-Value:**2.08E-8

HT12-ALS
GG (234) GT (84) TT (6)
Corr: 0.261 **R2:** 0.068
Z-Score:4.757 **P-Value:**1.96E-6

HT12-UC
GG (32) GT (17) TT (0)
Corr: 0.177 **R2:** 0.031
Z-Score:1.217 **P-Value:**0.224

H8v2-Controls
eQTL not available
Probe not present.

H8v2-CeliacDisease
eQTL not available
Probe not present.

H8v2-ALS
eQTL not available
Probe not present.

SNP rs660895 (chr. 6, 32685358 bp)
Probe 2940424 (chr. 7, 36518135 - 36731595 bp), AOAH
P-Value 1.07E-23

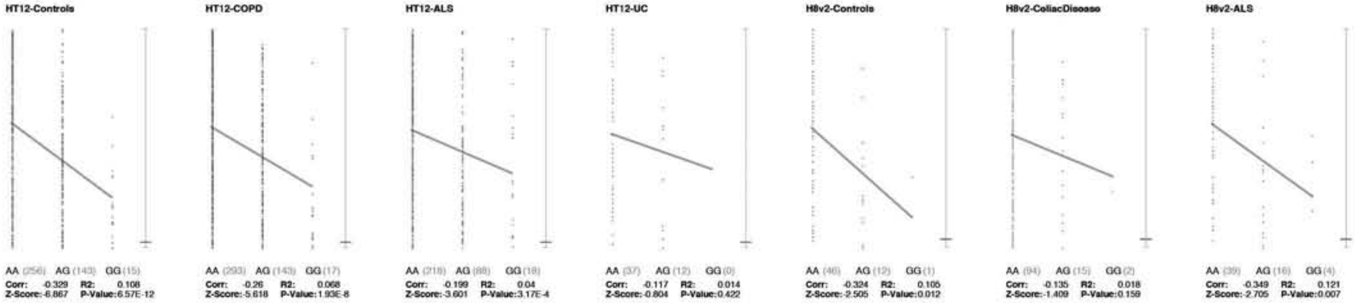

SNP rs12485738 (chr. 3, 56840816 bp)
Probe 1820386 (chr. 22, 42725424 - 42897434 bp), PARVB
P-Value 8.02E-24

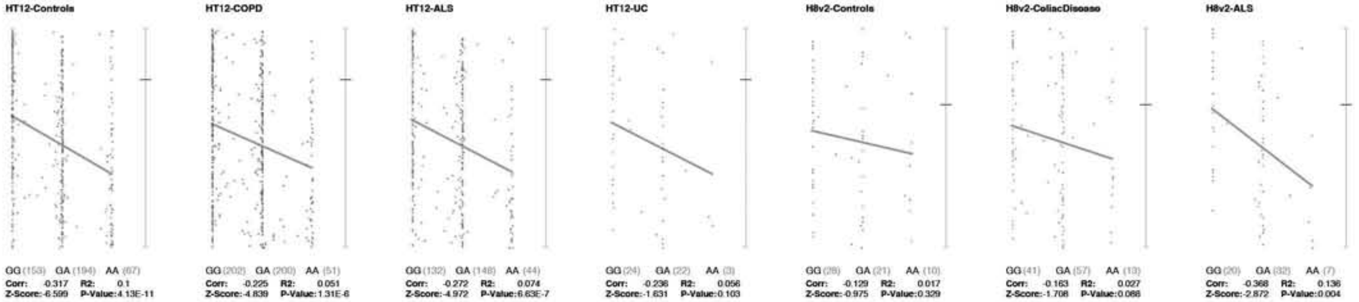

SNP rs766432 (chr. 2, 60573474 bp)
Probe 3440630 (chr. 1, 6424616 - 6447223 bp), ESPN///TNFRSF25
P-Value 2.97E-24

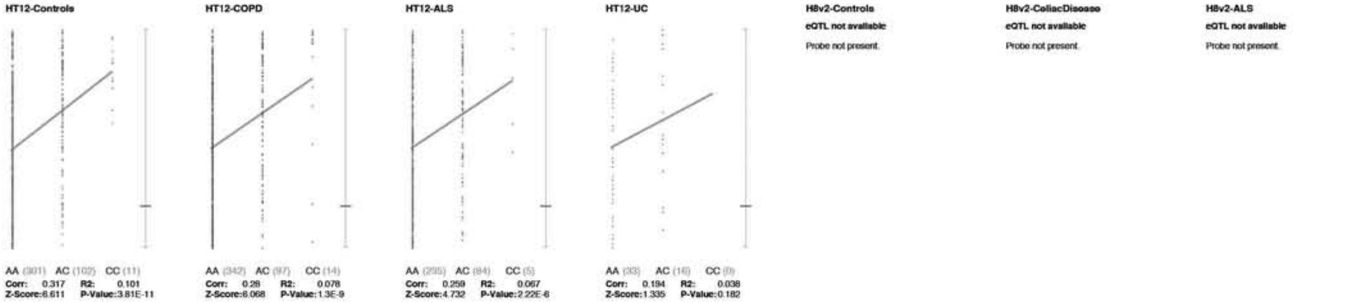

SNP rs2523393 (chr. 6, 29813638 bp)
Probe 5080687 (chr. 2, 206695080 - 206733432 bp), NDUFS1
P-Value 1.87E-25

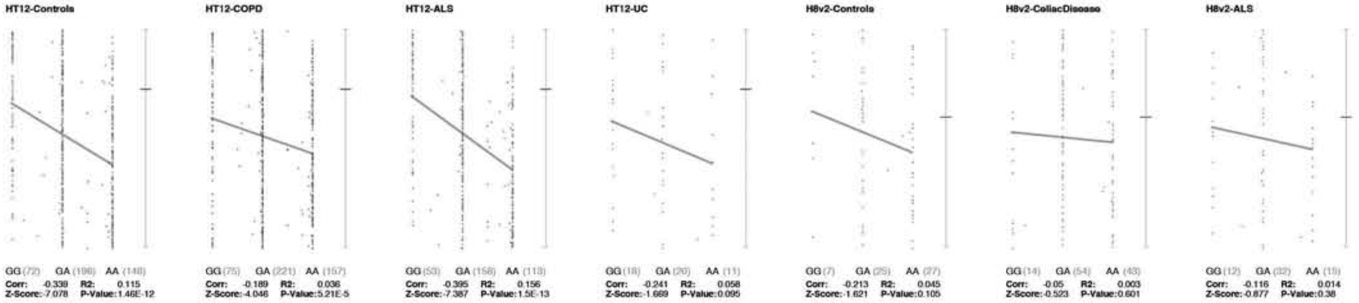

SNP rs7776054 (chr. 6, 135460609 bp)
Probe 4010040 (chr. 11, 5229996 - 5429091 bp), HBG2///ENST00000399563
P-Value 8.47E-27

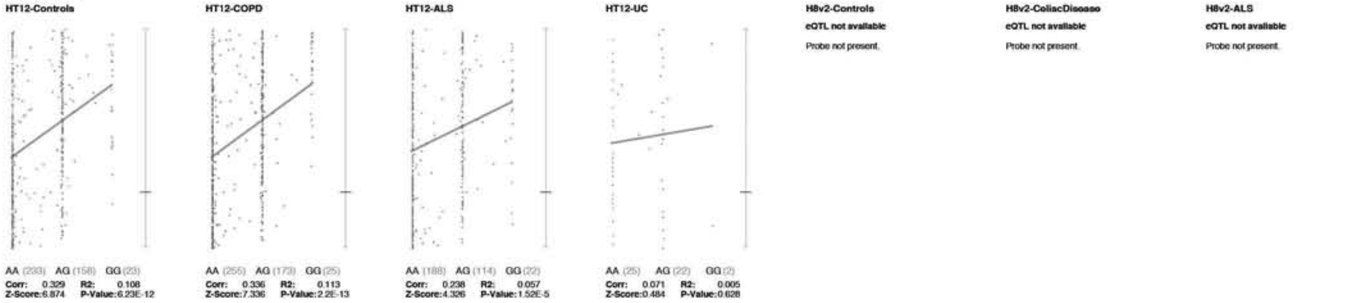

SNP rs9399137 (chr. 6, 135460711 bp)
Probe 4010040 (chr. 11, 5229996 - 5429091 bp), HBG2///ENST00000399563
P-Value 7.38E-27

HT12-Controls | **HT12-COPD** | **HT12-ALS** | **HT12-UC**

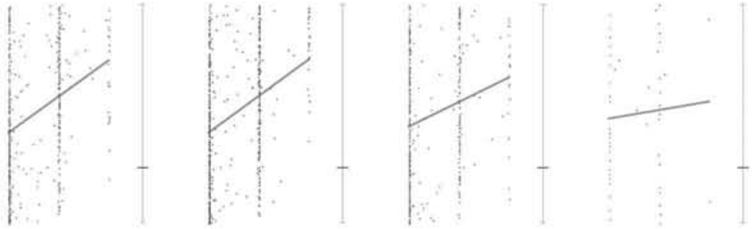

HT12-Controls	HT12-COPD	HT12-ALS	HT12-UC
TT (233) TC (158) CC (23)	TT (255) TC (173) CC (25)	TT (188) TC (114) CC (22)	TT (25) TC (22) CC (2)
Corr: 0.33 R2: 0.109	Corr: 0.336 R2: 0.113	Corr: 0.238 R2: 0.057	Corr: 0.071 R2: 0.005
Z-Score:6.878 P-Value:6.07E-12	Z-Score:7.343 P-Value:2.08E-13	Z-Score:4.328 P-Value:1.51E-5	Z-Score:0.484 P-Value:0.629

H8v2-Controls
eQTL not available
Probe not present.

H8v2-CeliacDisease
eQTL not available
Probe not present.

H8v2-ALS
eQTL not available
Probe not present.

SNP rs1427407 (chr. 2, 60571547 bp)
Probe 4010040 (chr. 11, 5229996 - 5429091 bp), HBG2///ENST00000399563
P-Value 2.26E-28

HT12-Controls | **HT12-COPD** | **HT12-ALS** | **HT12-UC**

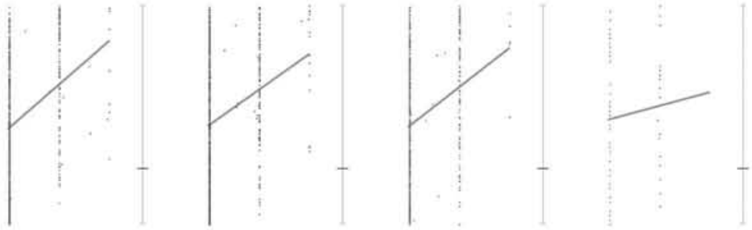

HT12-Controls	HT12-COPD	HT12-ALS	HT12-UC
GG (301) GT (101) TT (12)	GG (336) GT (102) TT (15)	GG (234) GT (84) TT (6)	GG (32) GT (17) TT (0)
Corr: 0.353 R2: 0.125	Corr: 0.289 R2: 0.084	Corr: 0.303 R2: 0.092	Corr: 0.102 R2: 0.01
Z-Score:7.406 P-Value:1.28E-13	Z-Score:6.272 P-Value:3.56E-10	Z-Score:5.566 P-Value:2.6E-8	Z-Score:0.694 P-Value:0.488

H8v2-Controls
eQTL not available
Probe not present.

H8v2-CeliacDisease
eQTL not available
Probe not present.

H8v2-ALS
eQTL not available
Probe not present.

SNP rs766432 (chr. 2, 60573474 bp)
Probe 4010040 (chr. 11, 5229996 - 5429091 bp), HBG2///ENST00000399563
P-Value 4.88E-29

HT12-Controls | **HT12-COPD** | **HT12-ALS** | **HT12-UC**

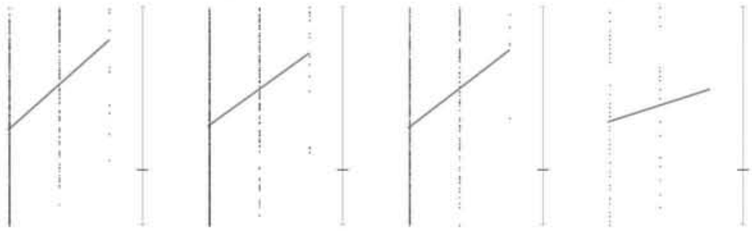

HT12-Controls	HT12-COPD	HT12-ALS	HT12-UC
AA (301) AC (102) CC (11)	AA (342) AC (97) CC (14)	AA (235) AC (84) CC (5)	AA (33) AC (16) CC (0)
Corr: 0.363 R2: 0.132	Corr: 0.293 R2: 0.086	Corr: 0.296 R2: 0.087	Corr: 0.117 R2: 0.014
Z-Score:7.62 P-Value:2.53E-14	Z-Score:6.359 P-Value:2.03E-10	Z-Score:5.423 P-Value:5.86E-8	Z-Score:0.8 P-Value:0.424

H8v2-Controls
eQTL not available
Probe not present.

H8v2-CeliacDisease
eQTL not available
Probe not present.

H8v2-ALS
eQTL not available
Probe not present.

SNP rs2292239 (chr. 12, 54768447 bp)
Probe 5890707 (chr. 4, 17410376 - 17422479 bp), AC110758.2
P-Value 1.89E-29

HT12-Controls | **HT12-COPD** | **HT12-ALS** | **HT12-UC** | **H8v2-Controls** | **H8v2-CeliacDisease** | **H8v2-ALS**

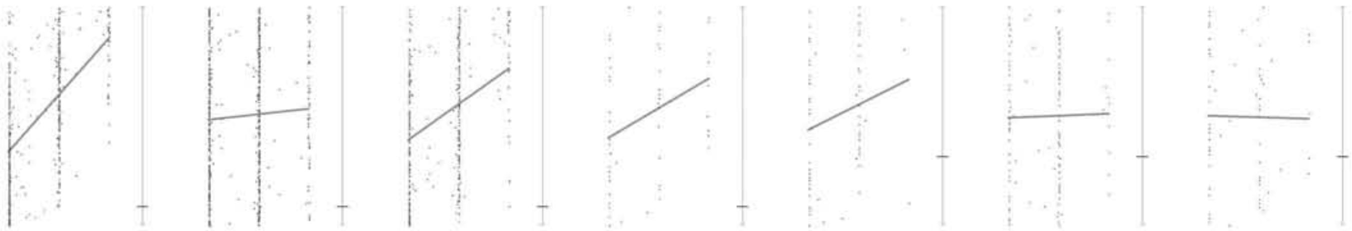

HT12-Controls	HT12-COPD	HT12-ALS	HT12-UC	H8v2-Controls	H8v2-CeliacDisease	H8v2-ALS
GG (196) GT (180) TT (38)	GG (198) GT (206) TT (48)	GG (144) GT (147) TT (33)	GG (23) GT (17) TT (9)	GG (30) GT (26) TT (3)	GG (44) GT (55) TT (12)	GG (28) GT (26) TT (5)
Corr: 0.566 R2: 0.32	Corr: 0.059 R2: 0.003	Corr: 0.353 R2: 0.125	Corr: 0.339 R2: 0.115	Corr: 0.225 R2: 0.05	Corr: 0.018 R2: 0	Corr: -0.015 R2: 0
Z-Score:12.608 P-Value:1.91E-36	Z-Score:1.248 P-Value:0.212	Z-Score:6.546 P-Value:5.93E-11	Z-Score:2.384 P-Value:0.017	Z-Score:1.711 P-Value:0.087	Z-Score:0.186 P-Value:0.852	Z-Score:-0.113 P-Value:0.91

SNP rs1701704 (chr. 12, 54698754 bp)
Probe 5890707 (chr. 4, 17410376 - 17422479 bp), AC110758.2
P-Value 1.68E-30

HT12-Controls | **HT12-COPD** | **HT12-ALS** | **HT12-UC** | **H8v2-Controls** | **H8v2-CeliacDisease** | **H8v2-ALS**

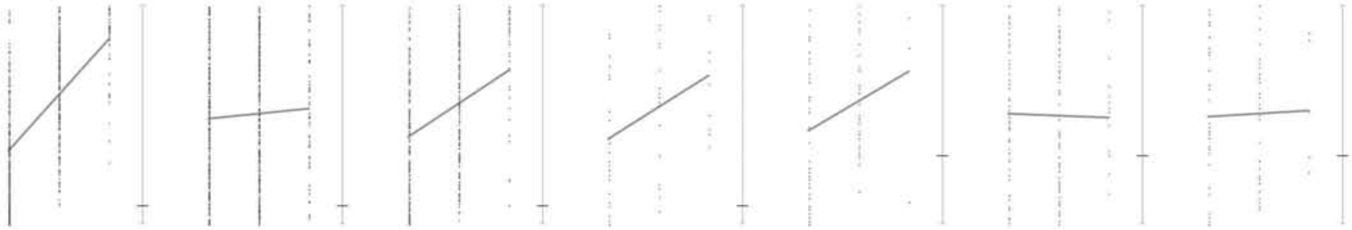

HT12-Controls	HT12-COPD	HT12-ALS	HT12-UC	H8v2-Controls	H8v2-CeliacDisease	H8v2-ALS
TT (193) TG (178) GG (43)	TT (196) TG (206) GG (51)	TT (145) TG (146) GG (33)	TT (22) TG (18) GG (9)	TT (31) TG (25) GG (3)	TT (44) TG (54) GG (13)	TT (27) TG (27) GG (5)
Corr: 0.582 R2: 0.338	Corr: 0.055 R2: 0.003	Corr: 0.347 R2: 0.12	Corr: 0.366 R2: 0.134	Corr: 0.274 R2: 0.075	Corr: -0.018 R2: 0	Corr: 0.031 R2: 0.001
Z-Score:13.038 P-Value:7.44E-39	Z-Score:1.164 P-Value:0.244	Z-Score:6.423 P-Value:1.34E-10	Z-Score:2.584 P-Value:0.01	Z-Score:2.098 P-Value:0.036	Z-Score:-0.186 P-Value:0.852	Z-Score:0.236 P-Value:0.814

SNP rs7776054 (chr. 6, 135460609 bp)
Probe 450537 (chr. 11, 5229996 - 5429091 bp), HBG2///ENST00000399563
P-Value 7.75E-31

HT12-Controls | HT12-COPD | HT12-ALS | HT12-UC | H8v2-Controls | H8v2-CeliacDisease | H8v2-ALS

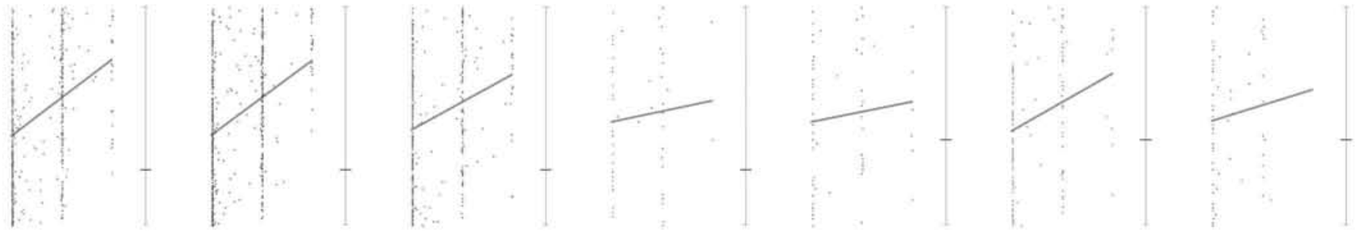

Dataset	Genotypes	Corr:	R2:	Z-Score:	P-Value:
HT12-Controls	AA (233) AG (158) GG (23)	0.35	0.122	7.323	2.42E-13
HT12-COPD	AA (255) AG (173) GG (25)	0.341	0.116	7.467	8.21E-14
HT12-ALS	AA (188) AG (114) GG (22)	0.266	0.071	4.857	1.19E-6
HT12-UC	AA (25) AG (22) GG (2)	0.091	0.008	0.625	0.532
H8v2-Controls	AA (34) AG (19) GG (6)	0.102	0.01	0.772	0.44
H8v2-CeliacDisease	AA (65) AG (37) GG (9)	0.275	0.076	2.922	0.003
H8v2-ALS	AA (42) AG (17) GG (0)	0.104	0.011	0.785	0.432

SNP rs9399137 (chr. 6, 135460711 bp)
Probe 450537 (chr. 11, 5229996 - 5429091 bp), HBG2///ENST00000399563
P-Value 6.57E-31

HT12-Controls | HT12-COPD | HT12-ALS | HT12-UC | H8v2-Controls | H8v2-CeliacDisease | H8v2-ALS

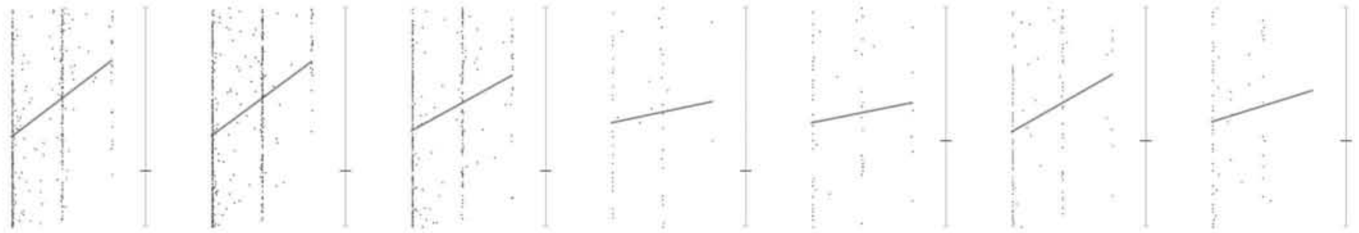

Dataset	Genotypes	Corr:	R2:	Z-Score:	P-Value:
HT12-Controls	TT (233) TC (158) CC (23)	0.35	0.122	7.327	2.35E-13
HT12-COPD	TT (255) TC (173) CC (25)	0.342	0.117	7.475	7.75E-14
HT12-ALS	TT (188) TC (114) CC (22)	0.266	0.071	4.858	1.19E-6
HT12-UC	TT (25) TC (22) CC (2)	0.092	0.008	0.626	0.531
H8v2-Controls	TT (34) TC (19) CC (6)	0.102	0.01	0.772	0.44
H8v2-CeliacDisease	TT (65) TC (37) CC (9)	0.275	0.076	2.923	0.003
H8v2-ALS	TT (42) TC (17) CC (0)	0.104	0.011	0.781	0.435

SNP rs12718597 (chr. 7, 50395922 bp)
Probe 4860181 (chr. 23, 48200866 - 48214509 bp), SLC38A5
P-Value 3.88E-31

HT12-Controls | HT12-COPD | HT12-ALS | HT12-UC | H8v2-Controls | H8v2-CeliacDisease | H8v2-ALS

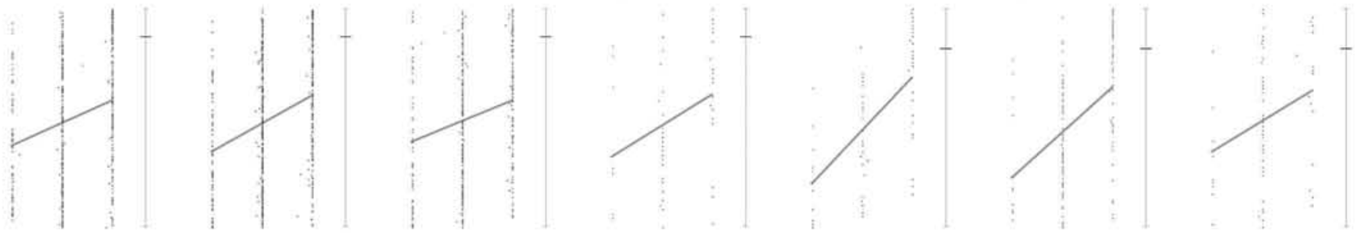

Dataset	Genotypes	Corr:	R2:	Z-Score:	P-Value:
HT12-Controls	AA (64) AC (197) CC (153)	0.249	0.062	5.125	2.97E-7
HT12-COPD	AA (68) AC (226) CC (159)	0.305	0.093	6.631	3.34E-11
HT12-ALS	AA (56) AC (163) CC (105)	0.224	0.05	4.067	4.77E-5
HT12-UC	AA (7) AC (23) CC (19)	0.329	0.108	2.31	0.021
H8v2-Controls	AA (11) AC (23) CC (25)	0.612	0.375	5.154	2.55E-7
H8v2-CeliacDisease	AA (11) AC (53) CC (47)	0.454	0.206	5.003	5.64E-7
H8v2-ALS	AA (9) AC (35) CC (15)	0.297	0.088	2.281	0.023

SNP rs9373124 (chr. 6, 135464902 bp)
Probe 4010040 (chr. 11, 5229996 - 5429091 bp), HBG2///ENST00000399563
P-Value 1.62E-31

HT12-Controls | HT12-COPD | HT12-ALS | HT12-UC

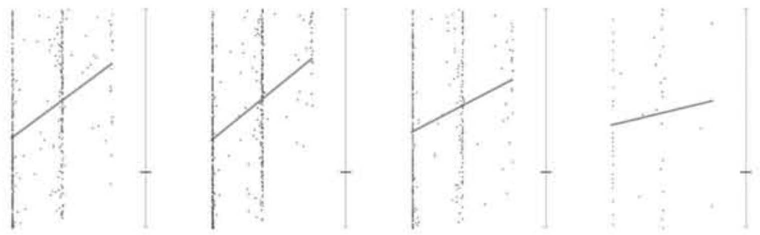

Dataset	Genotypes	Corr:	R2:	Z-Score:	P-Value:
HT12-Controls	TT (220) TC (169) CC (25)	0.343	0.117	7.167	7.65E-13
HT12-COPD	TT (236) TC (188) CC (29)	0.377	0.142	8.313	9.36E-17
HT12-ALS	TT (182) TC (116) CC (26)	0.254	0.064	4.629	3.68E-6
HT12-UC	TT (24) TC (22) CC (3)	0.105	0.011	0.715	0.475

H8v2-Controls
eQTL not available
Probe not present.

H8v2-CeliacDisease
eQTL not available
Probe not present.

H8v2-ALS
eQTL not available
Probe not present.

SNP rs9402686 (chr. 6, 135469510 bp)
Probe 4010040 (chr. 11, 5229996 - 5429091 bp), HBG2///ENST00000399563
P-Value 7.42E-32

HT12-Controls | HT12-COPD | HT12-ALS | HT12-UC

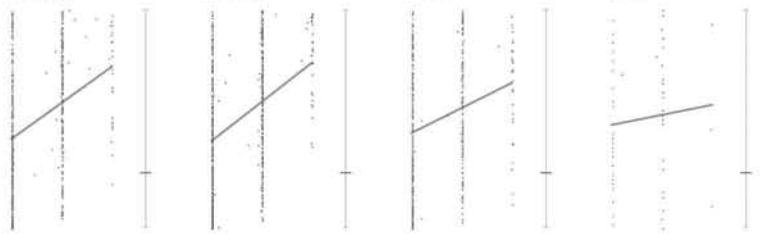

Dataset	Genotypes	Corr:	R2:	Z-Score:	P-Value:
HT12-Controls	GG (218) GA (169) AA (27)	0.35	0.123	7.338	2.17E-13
HT12-COPD	GG (235) GA (188) AA (30)	0.376	0.141	8.279	1.24E-16
HT12-ALS	GG (179) GA (117) AA (28)	0.255	0.065	4.65	3.32E-6
HT12-UC	GG (24) GA (22) AA (3)	0.091	0.008	0.624	0.533

H8v2-Controls
eQTL not available
Probe not present.

H8v2-CeliacDisease
eQTL not available
Probe not present.

H8v2-ALS
eQTL not available
Probe not present.

SNP rs4895441 (chr. 6, 135468266 bp)
Probe 4010040 (chr. 11, 5229996 - 5429091 bp), HBG2///ENST00000399563
P-Value 5.85E-33

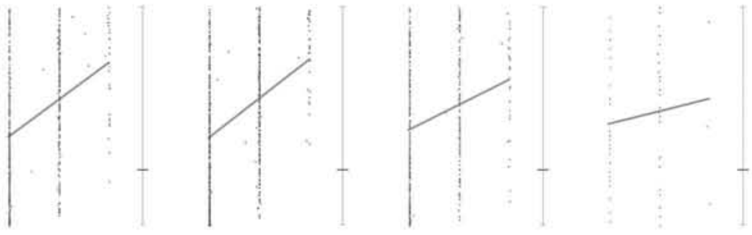

	HT12-Controls	HT12-COPD	HT12-ALS	HT12-UC	H8v2-Controls	H8v2-CeliacDisease	H8v2-ALS
Genotypes	AA (217) AG (167) GG (30)	AA (235) AG (167) GG (31)	AA (178) AG (118) GG (28)	AA (23) AG (23) GG (3)	eQTL not available	eQTL not available	eQTL not available
Corr:	0.36	0.379	0.256	0.113	Probe not present.	Probe not present.	Probe not present.
R2:	0.13	0.144	0.065	0.013			
Z-Score:	7.568	8.361	4.665	0.774			
P-Value:	3.8E-14	6.23E-17	3.09E-6	0.439			

SNP rs12718597 (chr. 7, 50385922 bp)
Probe 1510612 (chr. 17, 42640427 - 42657044 bp), MYL4
P-Value 5.91E-33

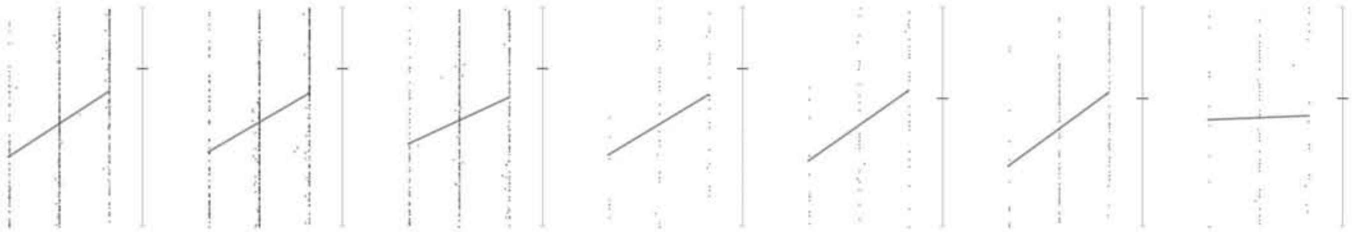

	HT12-Controls	HT12-COPD	HT12-ALS	HT12-UC	H8v2-Controls	H8v2-CeliacDisease	H8v2-ALS
Genotypes	AA (64) AC (197) CC (153)	AA (68) AC (226) CC (159)	AA (56) AC (163) CC (105)	AA (7) AC (23) CC (19)	AA (11) AC (23) CC (25)	AA (11) AC (53) CC (47)	AA (9) AC (35) CC (15)
Corr:	0.354	0.308	0.247	0.321	0.405	0.372	0.02
R2:	0.126	0.095	0.061	0.103	0.164	0.138	0
Z-Score:	7.434	6.693	4.497	2.251	3.18	4.018	0.148
P-Value:	1.05E-13	2.18E-11	6.89E-6	0.024	0.001	5.86E-5	0.883

SNP rs9376092 (chr. 6, 135468837 bp)
Probe 4010040 (chr. 11, 5229996 - 5429091 bp), HBG2///ENST00000399563
P-Value 4.53E-33

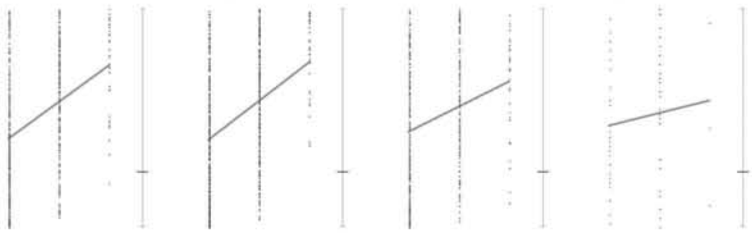

	HT12-Controls	HT12-COPD	HT12-ALS	HT12-UC	H8v2-Controls	H8v2-CeliacDisease	H8v2-ALS
Genotypes	CC (216) CA (167) AA (31)	CC (233) CA (189) AA (31)	CC (178) CA (118) AA (28)	CC (23) CA (23) AA (3)	eQTL not available	eQTL not available	eQTL not available
Corr:	0.364	0.376	0.257	0.115	Probe not present.	Probe not present.	Probe not present.
R2:	0.132	0.142	0.066	0.013			
Z-Score:	7.646	8.295	4.692	0.786			
P-Value:	2.07E-14	1.09E-16	2.71E-6	0.432			

SNP rs1427407 (chr. 2, 60571547 bp)
Probe 450537 (chr. 11, 5229996 - 5429091 bp), HBG2///ENST00000399563
P-Value 2E-36

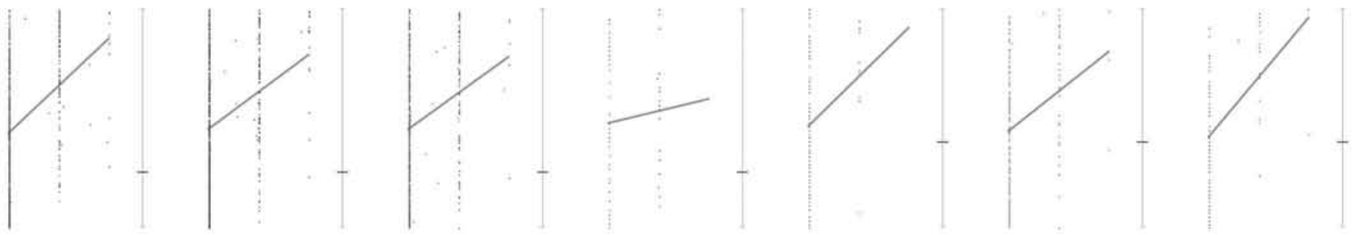

	HT12-Controls	HT12-COPD	HT12-ALS	HT12-UC	H8v2-Controls	H8v2-CeliacDisease	H8v2-ALS
Genotypes	GG (301) GT (101) TT (12)	GG (336) GT (102) TT (15)	GG (204) GT (84) TT (6)	GG (32) GT (17) TT (0)	GG (50) GT (9) TT (0)	GG (78) GT (30) TT (3)	GG (42) GT (15) TT (2)
Corr:	0.378	0.305	0.282	0.089	0.274	0.322	0.49
R2:	0.143	0.093	0.079	0.008	0.075	0.104	0.24
Z-Score:	7.966	6.642	5.158	0.605	2.103	3.445	3.941
P-Value:	1.64E-15	3.09E-11	2.49E-7	0.545	0.035	5.71E-4	8.13E-5

SNP rs2395185 (chr. 6, 32541145 bp)
Probe 2940424 (chr. 7, 36518135 - 36731595 bp), AOAH
P-Value 1.86E-36

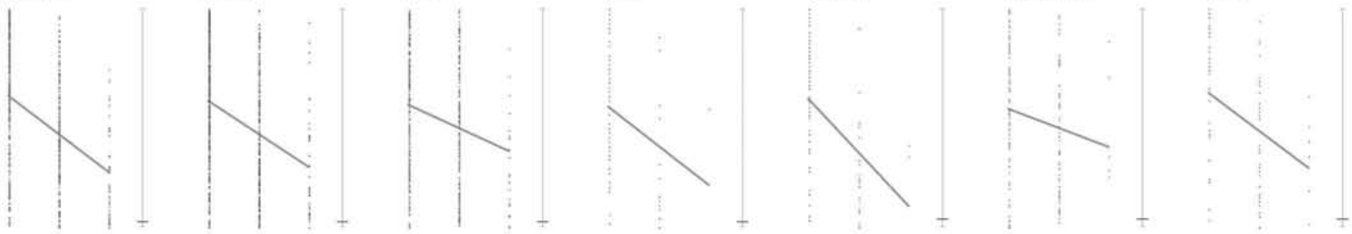

	HT12-Controls	HT12-COPD	HT12-ALS	HT12-UC	H8v2-Controls	H8v2-CeliacDisease	H8v2-ALS
Genotypes	GG (209) GT (167) TT (38)	GG (245) GT (173) TT (35)	GG (158) GT (145) TT (21)	GG (36) GT (12) TT (1)	GG (40) GT (17) TT (2)	GG (61) GT (45) TT (5)	GG (25) GT (28) TT (6)
Corr:	-0.394	-0.336	-0.222	-0.3	-0.455	-0.176	-0.382
R2:	0.155	0.113	0.049	0.09	0.207	0.031	0.146
Z-Score:	-8.332	-7.337	-4.035	-2.097	-3.618	-1.842	-2.982
P-Value:	7.97E-17	2.19E-13	5.46E-5	0.036	2.97E-4	0.065	0.003

SNP rs766432 (chr. 2, 60573474 bp)
Probe 450537 (chr. 11, 5229996 - 5429091 bp), HBG2///ENST00000399563
P-Value 3.49E-37

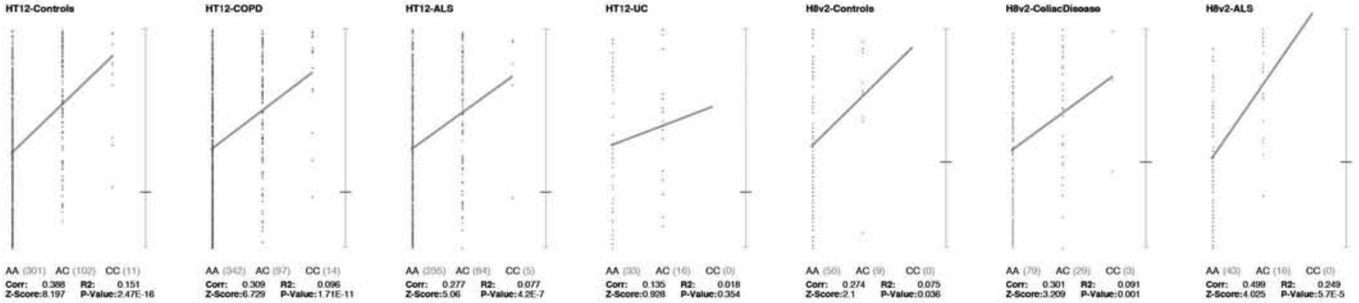

SNP rs9373124 (chr. 6, 135464902 bp)
Probe 450537 (chr. 11, 5229996 - 5429091 bp), HBG2///ENST00000399563
P-Value 5.86E-38

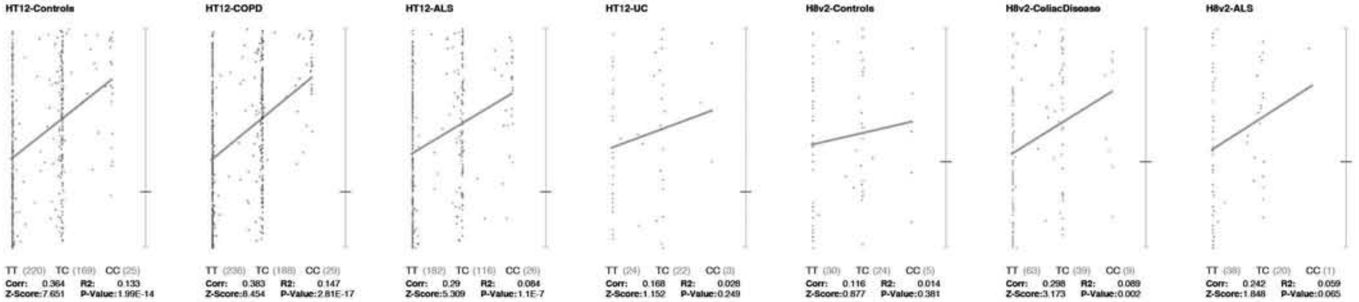

SNP rs9402686 (chr. 6, 135469510 bp)
Probe 450537 (chr. 11, 5229996 - 5429091 bp), HBG2///ENST00000399563
P-Value 2.55E-38

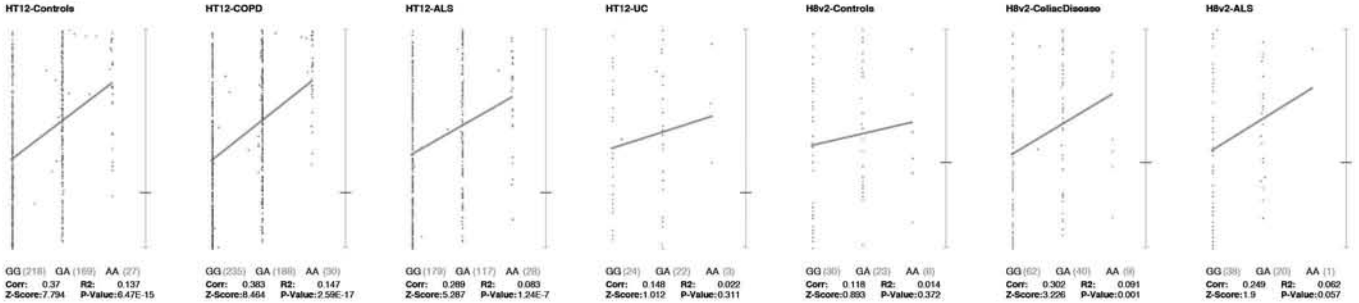

SNP rs2292239 (chr. 12, 54768447 bp)
Probe 5890554 (chr. 4, 41816859 - 41816859 bp), -
P-Value 7.23E-39

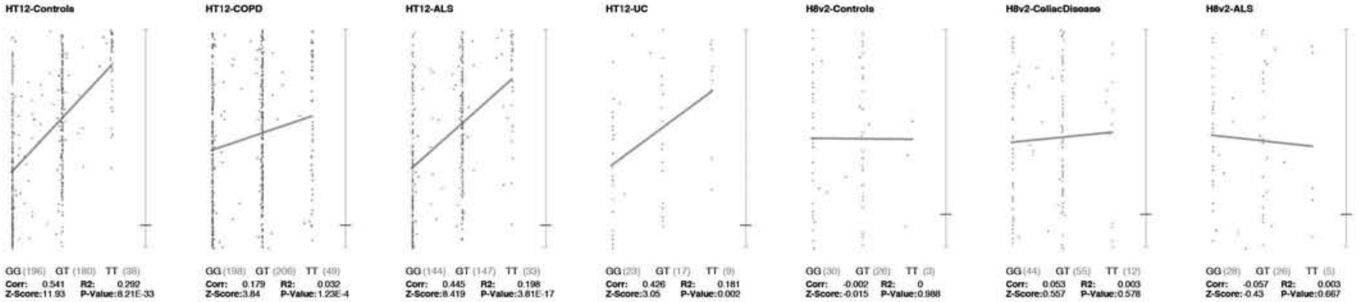

SNP rs1701704 (chr. 12, 54698754 bp)
Probe 5890554 (chr. 4, 41816859 - 41816859 bp), -
P-Value 3.64E-39

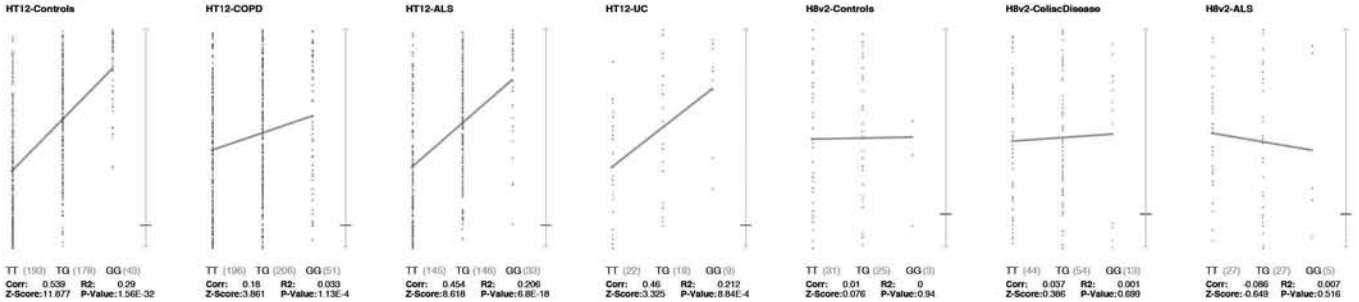

SNP rs4895441 (chr. 6, 135468266 bp)
Probe 450537 (chr. 11, 5229996 - 5429091 bp), HBG2///ENST00000399563
P-Value 5.76E-40

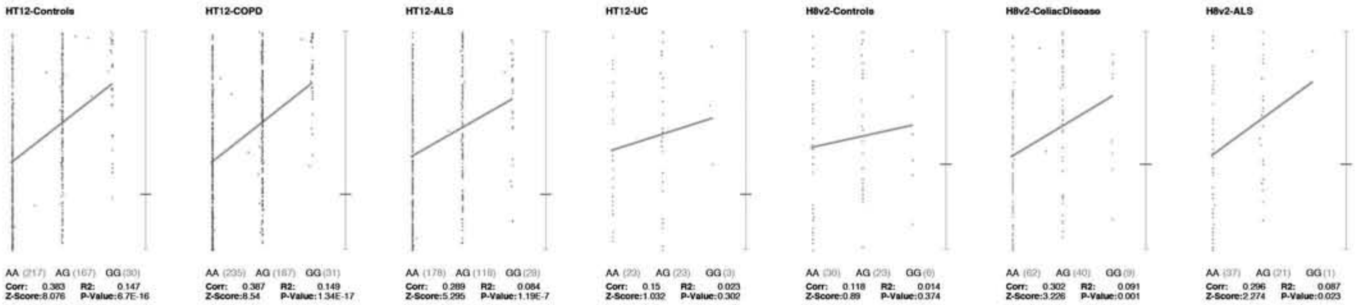

SNP rs9376092 (chr. 6, 135468837 bp)
Probe 450537 (chr. 11, 5229996 - 5429091 bp), HBG2///ENST00000399563
P-Value 3.8E-40

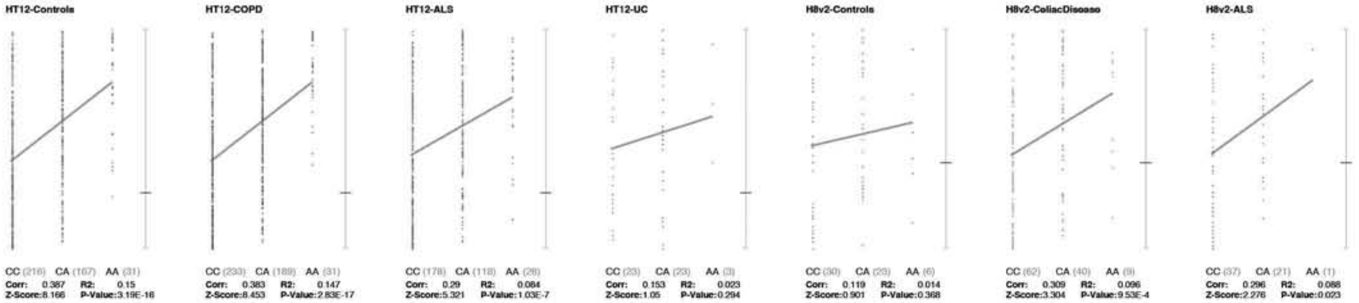

SNP rs11171739 (chr. 12, 54756892 bp)
Probe 5890707 (chr. 4, 17410376 - 17422479 bp), AC110758.2
P-Value 4.8E-44

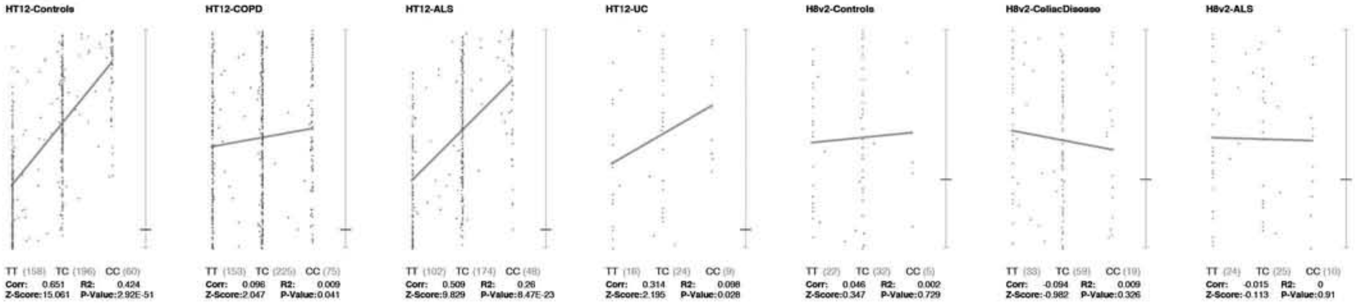

SNP rs12718597 (chr. 7, 50385922 bp)
Probe 4900309 (chr. 2, 190709731 - 190777453 bp), AC010679.6
P-Value 3.26E-51

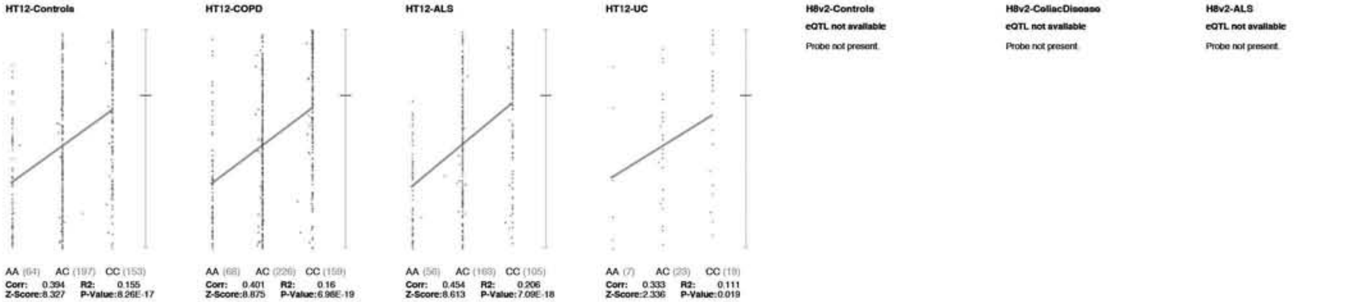

SNP rs11171739 (chr. 12, 54756892 bp)
Probe 5890554 (chr. 4, 41816859 - 41816859 bp), -
P-Value 1.82E-59

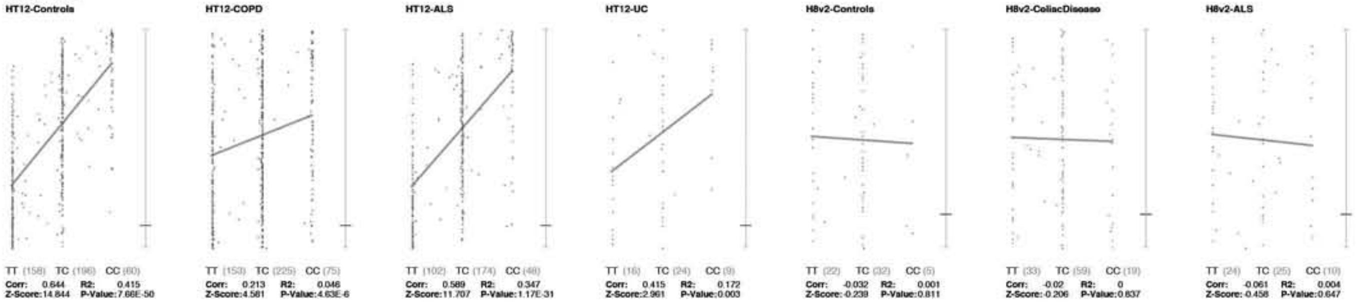

Supplement: Table S6 — Plots of detected trans-eQTLs for 1,167 trait-associated SNPs for each of the seven individual cohorts of samples that make up the total of 1,469 peripheral blood samples. (PDF) [file pgen.1002197.s015.pdf]
